# Genome-Wide Analysis of Members of the *WRKY* Gene Family and Their Cold Stress Response in *Prunus mume*

**DOI:** 10.3390/genes10110911

**Published:** 2019-11-08

**Authors:** Fei Bao, Anqi Ding, Tangren Cheng, Jia Wang, Qixiang Zhang

**Affiliations:** 1 Beijing Advanced Innovation Center for Tree Breeding by Molecular Design, Beijing Forestry University, Beijing 100083, China; baofei@bjfu.edu.cn (F.B.); angelddaq@163.com (A.D.); 2 Beijing Key Laboratory of Ornamental Plants Germplasm Innovation & Molecular Breeding, Beijing Forestry University, Beijing 100083, China; chengtangren@163.com (T.C.); 13910229248@163.com (J.W.); 3 National Engineering Research Center for Floriculture, Beijing Forestry University, Beijing 100083, China; 4 Beijing Laboratory of Urban and Rural Ecological Environment, Beijing Forestry University, Beijing 100083, China; 5 Key Laboratory of Genetics and Breeding in Forest Trees and Ornamental Plants of Ministry of Education, Beijing Forestry University, Beijing 100083, China; 6 School of Landscape Architecture, Beijing Forestry University, Beijing 100083, China

**Keywords:** *Prunus mume*, genome, *WRKY* genes, cold response

## Abstract

*Prunus mume*, which is a rosaceous arbor with very high ornamental, edible and medical values, has a distribution that is mainly restricted by low temperature. *WRKY* transcription factor genes play crucial roles in the growth, development, and stress responses of plants. However, the *WRKY* gene family has not been characterised in *P*. *mume*. There were 58 *PmWRKYs* identified from genome of *P. mume*. They were anchored onto eight link groups and categorised into three broad groups. The gene structure and motif composition were reasonably conservative in each group. Investigation of gene duplication indicated that nine and seven *PmWRKYs* were arranged in tandem and segmental duplications, respectively. *PmWRKYs* were discriminately expressed in different tissues (i.e., roots, stems, leaves, flowers and fruits) in *P. mume*. The 17 cold-related candidate genes were selected based on RNA-seq data. Further, to investigate the function of *PmWRKYs* in low temperatures, the expression patterns under artificial cold treatments were analysed. The results showed that the expression levels of the 12 *PmWRKYs* genes significantly and 5 genes slightly changed in stems. In particular, the expression level of *PmWRKY18* was up-regulated after ABA treatment. In addition, the spatiotemporal expression patterns of 17 *PmWRKYs* were analysed in winter. These results indicated that 17 PmWRKYs were potential transcription factors regulating cold resistance in *P. mume*.

## 1. Introduction

Low temperature is one of the most important abiotic factors affecting plant growth and development, and ultimately affecting the geographical distribution of plants. Chilling stress (0–15 °C) disrupts cellular homeostasis by altering the fatty acid composition of the membrane and accumulating the reactive oxygen species (ROS) in some organelles, which can deactivate proteins and interfere with normal physiological processes, especially for photosynthesis [1,2,3]. Freezing stress causes ice formation in intracellular and intercellular space, which can promote serious injuries and cell dehydration [4,5].

The stress response of plants is regulated by several transcription factors. The WRKYs transcription family was reported to participate in the regulation of plant growth, development, biotic, and abiotic stress responses [6,7]. The WRKY transcription factors have a unique WRKY domain of approximately 60 amino acid (AA) residues that are mainly composed of two parts: the highly conserved heptapeptide sequence WRKYGQK and a metal chelating zinc-finger motif [8]. According to the number of WRKY domains and the type of the zinc-finger motif (C_2_H_2_ or C_2_HC), WRKYs can be categorised into three groups: I, II and III [7]. Group I was composed of WRKYs with two WRKY domains. Group II proteins, which have only one WRKY domain and a C_2_H_2_ zinc-finger motif, can be further subdivided into five subgroups (i.e., II a–e) based on short conserved structural motifs [7]. In addition, the only difference in the structure between group III and II proteins is that the former contains another type of zinc-finger motif, which ends with HXC. WRKY transcription factors could regulate the expression of target genes by binding to the W-boxes in the target genes’ promoters. Moreover, clusters of W-boxes could strengthen the effects [9,10,11,12,13]. In addition, WRKYs, which could bind to SURE element, PRE4 element and the WK box, have also been reported [6].

Many *WRKY* genes have been found and cloned from different plants since *SPF1* was isolated from sweet potato in 1994 [9]. So far, only two *WRKY* homologues have been identified from non-plants, *Giardia lamblia* and *Dictyostelium discoideum* [14,15]. A total of 58 *PpWRKY* genes have been identified in the genome of *Prunus persica* [16]. WRKY proteins have been verified to be essential in the defense response to various biotic and abiotic stresses, including low or high temperature, mechanical damage, and pathogenic infection [17,18,19,20,21,22,23]. In addition, there is a great deal of definite evidence indicating that the regulation of plant development and metabolism also requires WRKYs.

*Prunus mume* Sieb. et Zucc. (Rosaceae, *Prunus*) is a traditional flower with high ornamental value, and has been widely cultivated in China for over three thousand years. *P. mume* originated from the Yangtze River Basin and was later introduced to northern China. The low-temperature was considered a key ecological factor that affected the distribution of *P*. *mume*. Stems and buds are the most important above ground organs of *P*. *mume* for safely overwintering. Transcription factors play an important role in regulating this natural abiotic stress response. In this study, 58 *WRKY* genes were identified in the *P*. *mume* genome, and systematic analysis of the phylogeny, gene duplication, genomic localization, gene structure, and tissue-specific expression was conducted. Moreover, RNA-seq data and real-time quantitative RT-PCR (qRT-PCR) were employed to determine the expression patterns of *PmWRKYs* under artificial low temperature and exogenous ABA treatments, and during winter time. In conclusion, this study improves our understanding of the evolution of *PmWRKYs* family and provides the candidate genes for cold resistance research in *P. mume.*

## 2. Materials and Methods

### 2.1. Identification of P. mume WRKY Genes

To identify the *WRKY* family in *P. mume*, BLASTP and BLASTX were performed in the *P*. *mume* genomic database with the *WRKY* sequences of *Arabidopsis thaliana* that were downloaded from Plant Transcription Factor Database (http://planttfdb.cbi.pku.edu.cn/index.php). The hidden Markov model (HMM) searches were conducted in *P*. *mume* protein sequences using the WRKY domain HMM profile (PF03106). Candidate protein sequences without a WRKY domain were abandoned by screening of the SWISS-MODEL (http://swissmodel.expasy.org/). The sequences of genomic DNA, coding sequences (CDS) and protein sequences of PmWRKYs were obtained from National Center for Biotechnology Information (NCBI) (gene ID were shown in Table 1). ExPASy (http://web.expasy.org/compute_pi) and Plant-mPLoc (http://www.csbio.sjtu.edu.cn/bioinf/plant-multi/) were used to separately predict the molecular weight (MW), isoelectric point (pI) and protein subcellular localization. 

### 2.2. Genomic Localization and Gene Family Expansion Pattern Analysis

Based on the positional information in the *P*. *mume* genome database, genomic localization of *PmWRKYs* was drawn by MapDraw2.1 and Photoshop CS6.0. Research on gene family expansion pattern focused on tandem and segmental duplications. The tandemly arrayed genes were characterised as tandem duplications when they exhibited close phylogenetic relationships and were located at the same chromosomal location (within 100 kb) according to the criteria reported by Kong [24]. Segmental duplication regions were identified by the method of the Plant Genome Duplication Database [25]. First, the search for potential anchors was conducted by BLASTP (*E* < e^−6^). Subsequently, MCscan was applied to identify the homologous regions. Finally, syntenic blocks were evaluated using ColinearScan, and alignments with an *E* value < e^−10^ were deemed to be significant matches.

### 2.3. Phylogenetic and Conserved Domains Analysis

A phylogenetic tree was constructed using Neighbour-Joining (NJ) methods (bootstrap replicates: 1,000) by MEGA5.1 with the default value. To categorise PmWRKYs, 12 referential *A. thaliana* WRKYs from diverse groups were used: AtWRKY11 (AEE85928.1), AtWRKY14 (AAP21276.1), AtWRKY18 (AAM78067.1), AtWRKY20 (ANM67410.1), AtWRKY21 (AAB63078.1), AtWRKY27 (ABH04558.1), AtWRKY28 (AEE84006.1), AtWRKY31 (AEE84546.1), AtWRKY41 (AEE82969.1), AtWRKY43 (AEC10646.1), AtWRKY45 (ABD57509.1), and AtWRKY49 (AAQ62425.1). To examine the domain organization of WRKY proteins in detail, multiple sequence alignments of the WRKY domain sequences were conducted by DNAMAN 7.

### 2.4. Gene Structure and Conserved Motif Analysis

The genomic sequences and CDS of *PmWRKYs* (Additional File 5) were obtained from the *P*. *mume* genome database. The exon-intron structures were identified with the Gene Structure Display Server2.0 (GSDS, http://gsds.cbi.pku.edu.cn/). It should be noted that since the database only contains the information of gene coding region, the untranslated region (5′ UTR and 3′ UTR) cannot be displayed by mapping the gene structure. PmWRKY protein sequences were submitted to the Multiple Em for the Motif Elucidation program (MEME) (http://meme.nbcr.net/meme/) to identify the conserved motif with the following parameters: any number of repetitions; the motifs number was set to 20; motif width was set to 6–200. SMART (http://smart.embl-heidelberg.de/) and Pfam were employed to annotate the identified motifs.

### 2.5. Heat Map Analysis by Transcriptome Data

The RNA-seq data of the five tissues (young roots, young stems, leaves, flowers, and immature fruits) (NCBI, Sequence Read Archive (SRA): SRP014885) and leaf buds collected before and after freezing stress in winter of *P. mume* cultivar ‘Zhusha’ (NCBI, SRA: SRP131731) have been used to draw the heatmaps of the *PmWRKY* genes. The ‘before freezing’ samples were collected when the lowest temperature of the day was higher than 5 °C, and the ‘after freezing’ samples were collected when the highest temperature of the day is lower than 0 °C in 2014. The time interval between the two samplings was one month. HemI (Heat map illustrator) was employed to draw heat maps with the default value [26].

### 2.6. Chilling and ABA Treatments

The annual branches were collected from the *P*. *mume* cultivar ‘Yudie’ for artificial treatments. Before the chilling treatment, the braches were exposed to 22 °C overnight by water culture and then transferred to 4 °C for 0, 0.5, 1, 2, 4, 8, 12, 24, 48, and 72 h in dark. For the ABA treatment, the branches were sprayed with 100 μM ABA for 0, 0.5, 2, 6, 12, 24, and 48 h. The stems were collected after the artificial treatments. The first time point (0 h) served as a control.

The stems and buds of the cultivar ‘Yudie’ were collected in winter from 5 November, 2017 to 4 March, 2018, which were planted in the open air. The daily land surface temperatures were recorded. Three replications of each sample were collected and all of the test samples were stored at −80 °C before total RNA isolation.

### 2.7. Gene Expression Analyses 

Total RNA of each sample was extracted using the EASYspin Plus Plant RNA Extraction Kit (Aidlab, Beijing, China). The first-strand cDNA was obtained using a TIANScript RT Kit (KR107, Tiangen, Beijing, China). The specific primers were designed by Beacon Designer 8 based on cDNA sequences (Additional File 1). The expression levels of *PmWRKYs*, *PmCBF1* (LOC103333423), *PmCBF5* (LOC103337424), *PmCBF6* (LOC103344251), *PmLEA10* (LOC103340137) and *PmLEA29* (LOC103321165) during the artificial low temperature and exogenous ABA treatments were examined using qRT-PCR with the following programme: 95 °C for 30 s, 40 cycles of 95 °C for 5 s and 60 °C for 30 s. Using the *Actin* gene of *P. mume* (ID: LOC103332029) as an internal control gene, the relative expression levels were calculated by the 2^−ΔΔCt^ method. Three independent experiments were performed with similar results.

## 3. Results

### 3.1. Identification and Classification of WRKY Genes in P. mume

Two strategies were applied to identify *PmWRKY* genes: an HMM search and BLAST using well-characterised *WRKY* sequences from *A*. *thaliana* as queries. After SWISS-MODEL analysis, 58 sequences, which contained at least one WRKY domain, were obtained (Table 1). The 58 genes were named *PmWRKY01*-*PmWRKY58* according to the order of the gene ID and their corresponding protein sequences varied in length, MW as well as pI, as shown in Table 1. The length ranged between 162 and 884 AA, the MW ranged from 18.46 to 98.18 kDa and the pI varied from 4.92 to 9.68. According to the results of Plant-mPLoc, almost all of the PmWRKYs were predicted to be localised in the nucleus with high reliability except PmWRKY51, which was predicted to be targeted to chloroplasts besides the nucleus. 

**Figure 1:**
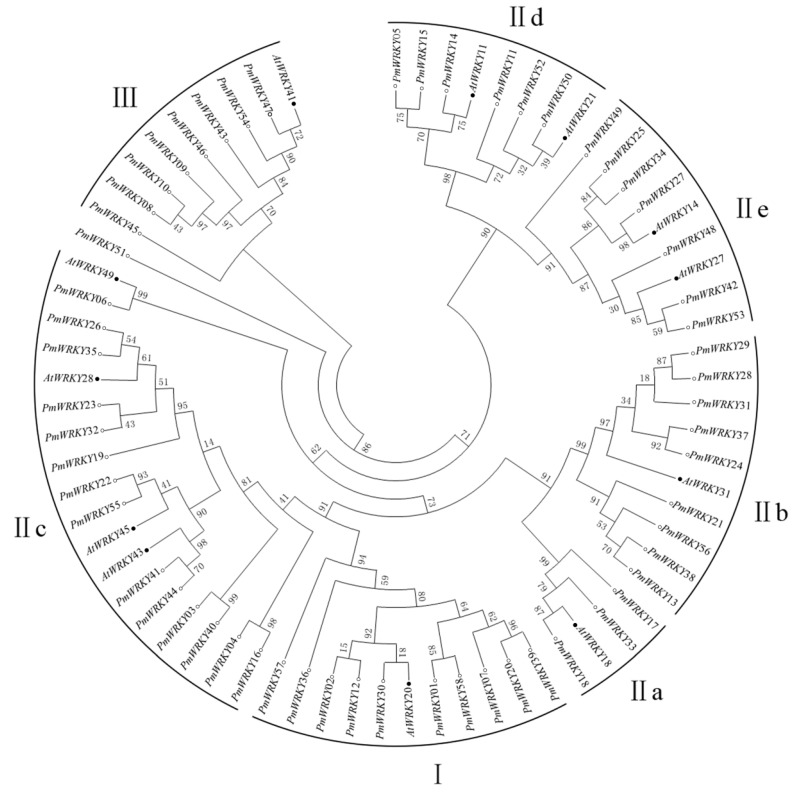
Tree of PmWRKYs. The unrooted phylogenetic tree of PmWRKY proteins was constructed using MEGA5.1 program by the neighbor-joining method with 1,000 bootstrap replicates. AtWRKYs were used as references to categorise PmWRKYs. The tree was divided into seven phylogenetic subgroups, designated as I, IIa-e, and III. The black solid points denote AtWRKYs, and the hollow points denote PmWRKYs.

The relationships among the 58 PmWRKYs are shown in the phylogenetic tree (Figure 1) produced by MEGA5.1; they were phylogenetically clustered into three main groups, which were similar to that of AtWRKYs [7]. Ten WRKYs, which contained WRKY domains and C_2_H_2_-type zinc-finger motifs in the N-terminal and the C-terminal were aggregated into group I. Group II consisted of 40 PmWRKYs, which were categorised into five subgroups using 12 diverse AtWRKY proteins, as references. Three members were clustered in subgroup II a, nine in II b, fourteen in II c, six in II d, and seven in II e. Furthermore, subgroup II c showed higher divergence than other subgroups, which was similar to those reported in the similar studies on other species [14,27,28,29]. It is worth mentioning that PmWRKY51 was not clustered into group II although it contained a WRKY domain and a C_2_H_2_-type zinc-finger motif. Since the protein length and domain locations were considerably different, PmWRKY51 was classified into another group. In addition, there were also eight WRKY proteins, which contained a C_2_HC-type zinc-finger motif in group III.

### 3.2. Genomic Localization and Duplication of the PmWRKY Genes

**Figure 2:**
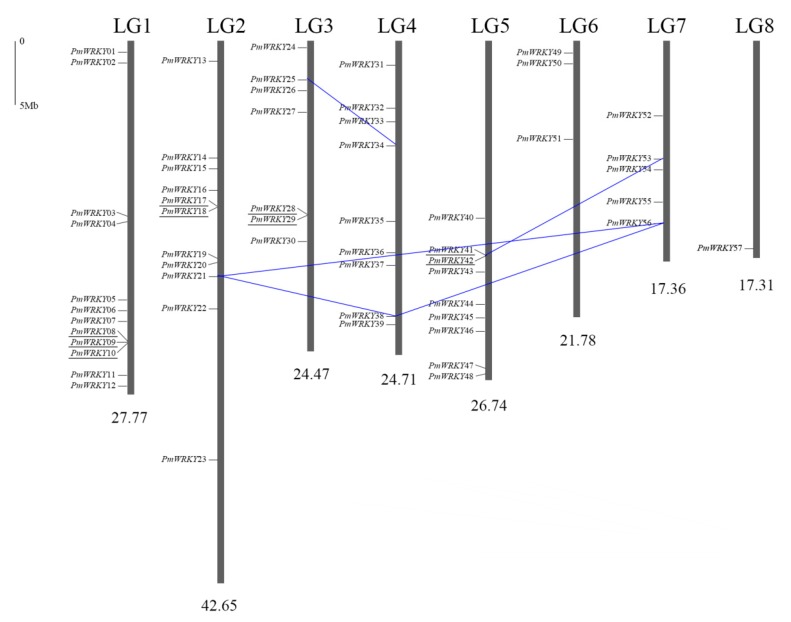
Localization and duplicated gene pairs of *PmWRKYs*. The 57 *PmWRKY* genes were mapped to the eight chromosomes. The chromosome number is indicated at the top. The scale refers to a 5 Mb chromosomal distance. Genes in tandem repeats are underlined in black. Segmental duplicate genes are linked by blue lines. There was a *WRKY* gene (*PmWRKY58*) that could not be clearly located on the chromosomes, but could be identified on scaffolds.

Based on the genomic database, 57 *PmWRKYs* were distributed on all eight link groups of *P*. *mume* randomly and unevenly (Figure 2), which leaves *PmWRKY58* on the scaffolds. There were twelve *PmWRKY* genes on LG 1 and eleven genes on LG 2. In contrast, LG 8 contained only one gene. LG 6, LG 7, LG 3, LG 4, and LG 5 contained 3, 5, 7, 9 and 9, respectively. It should be noted that group III genes were only located on LG 1, LG 5 and LG 7. Similar phenomenon occurred in *P*. *persica* [16], whereas the distribution of *WRKY* group III genes was even across all chromosomes in *Brassica rapa* ssp. *Pekinensis* [29]. The homologues of *PmWRKYs* in *P. persica* are shown in Table 1.

Gene duplication events fall into three categories: whole-genome duplication, tandem repeat and segmental duplication, which resulted in gene functional diversity, family evolution and plant adaptations [24]. Among the 58 *PmWRKY* genes, nine members were found in tandem repeats: *PmWRKY08* to *PmWRKY10*, *PmWRKY17* and *PmWRKY18*, *PmWRKY28* and *PmWRKY29*, and *PmWRKY41* and *PmWRKY42* (Figure 2). According to Holub’s definition, 3 or more genes within 200 kb are considered a gene cluster [30]. The only gene cluster, which consisted of three group III genes (*PmWRKY08*, *PmWRKY09*, and *PmWRKY10*), was on LG 1. Twenty-eight genes were located on duplicated segments; among them, three genes (*PmWRKY21*, *PmWRKY38* and *PmWRKY56*) were considered to be close relatives. *PmWRKY21*, *PmWRKY38* and *PmWRKY56* were located on LG 2, LG 4 and LG 7, respectively, and there was a triplet relationship among the three chromosomes [31]. Therefore, we surmised that these three *PmWRKYs* were generated along with a genome triplication process of *Prunus*. In addition, *PmWRKY25* and *PmWRKY34*, as well as *PmWRKY42* and *PmWRKY53*, were segmental duplicate pairs. The absence of the remaining 21 *PmWRKYs*’ corresponding relatives may be due to the gene evolution of the *P*. *mume WRKY* gene superfamily. No duplication event was observed in group I. Therefore, 15.5% of the *PmWRKY* genes can be explained by tandem duplication, 12.1% can be accounted for by segmental duplication, whereas 72.4% were monogenes, which indicated that the formation of *PmWRKY* genes may not rely primarily on gene duplication.

### 3.3. Gene Structure and Conserved Motif Analysis of PmWRKYs

**Figure 3:**
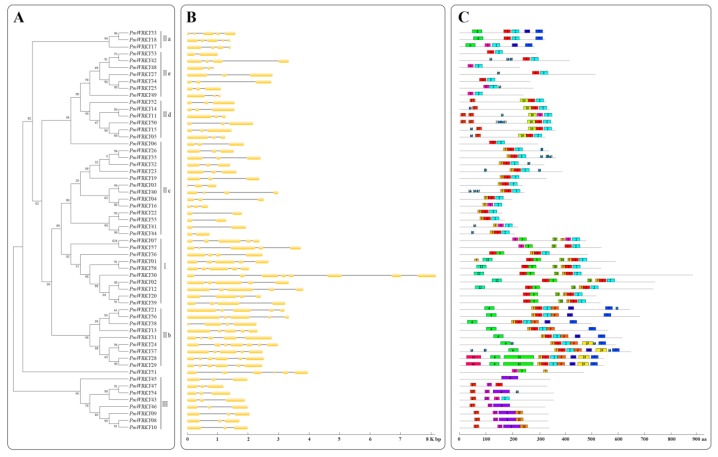
Relationships, gene structure and motif compositions of PmWRKYs. (**A**) The full-length PmWRKY protein sequences were aligned by ClustalW and the unrooted phylogenetic tree was constructed using MEGA5.1 program by the neighbor-joining method with 1,000 bootstrap replicates. (**B**) Exon/intron structures of the *PmWRKYs*. Yellow boxes represent exons and black lines represent introns. (**C**) Conserved motif analysis of PmWRKYs by MEME. Different motifs are represented by different colored boxes with numbers 1–20.

To make a thorough inquiry into the structural similarities and differences of *PmWRKYs*, we obtained the exon/intron structure diagrams and conserved motifs on the basis of the phylogenetic tree (Figure 3A). First, according to the genome sequences and CDS of the *PmWRKYs*, we found that all of the genes contained at least one intron (Figure 3B). Generally, the closest genes had similar structures, which only varied in the length of the intron and exon, whereas some genes exhibited different exon/intron arrangements. For instance, *PmWRKY03* contained three exons, whereas its nearby paralogous gene *PmWRKY40* had four exons and three introns even though their evolutionary relationships reached a 99% bootstrap value. Finally, all of the members of the subgroups II d and III were consistent with the number of exons. However, there was no significant consistency in the number of exons within other subgroups.

The MEME online tool predicted 20 individual motifs and revealed the specific regions of PmWRKYs (Figure 3C and Additional File 2). An analysis of the 20 motifs revealed that the lengths of PmWRKY motifs ranged from 7 to 113 amino acid residues and the number of motifs varied from 1 to 11 in each PmWRKY protein. As shown in Figure 3C, motif composition was similar among the same subgroup, which suggested functional similarities of these PmWRKYs, whereas those of different subgroups had no common conserved motifs except for the C-terminal conserved motifs. For instance, motifs 3, 12, and 20 were conserved in group I. Motifs 9, 11, 16, 19 were specific to subgroup II b. Motif 10 existed only in group II d. Motifs 1 and 2 were commonly shared by nearly all of the members of groups I and II, which were part of the WRKY domains. The results indicated that the conserved motifs mentioned above may bear special functions. According to the evolutionary analysis, II a and II b are two adjacent subgroups with near genetic distance, three unique motifs (motif 6, motif 7, and motif 14) nearly exist in all of the members of subgroups II a and II b, which supported the classification of PmWRKYs. In groups I and II, motif 1 and motif 8 both corresponded to the conserved heptapeptide domains, whereas motif 2 or motif 3 denoted the C_2_H_2_-type zinc-finger domain. However, in group I, motif 2 and motif 3 existed as a part of the N-terminal WRKY domain (NTWD) and the C-terminal WRKY domain (CTWD), respectively, which illustrated that the two WRKY domains belonging to family members of group I may be different in origin or function differentiation. It was also worth mentioning that there are three specific motifs (motif 4, motif 15, and motif 18) in PmWRKY08, PmWRKY09 and PmWRKY10 of group III. Although the function of the major motifs in the PmWRKYs was still indefinite; the PmWRKYs with the same conserved motifs may have similar functions.

### 3.4. Multiple Sequence Alignment of the WRKY Domains of PmWRKYs

**Figure 4:**
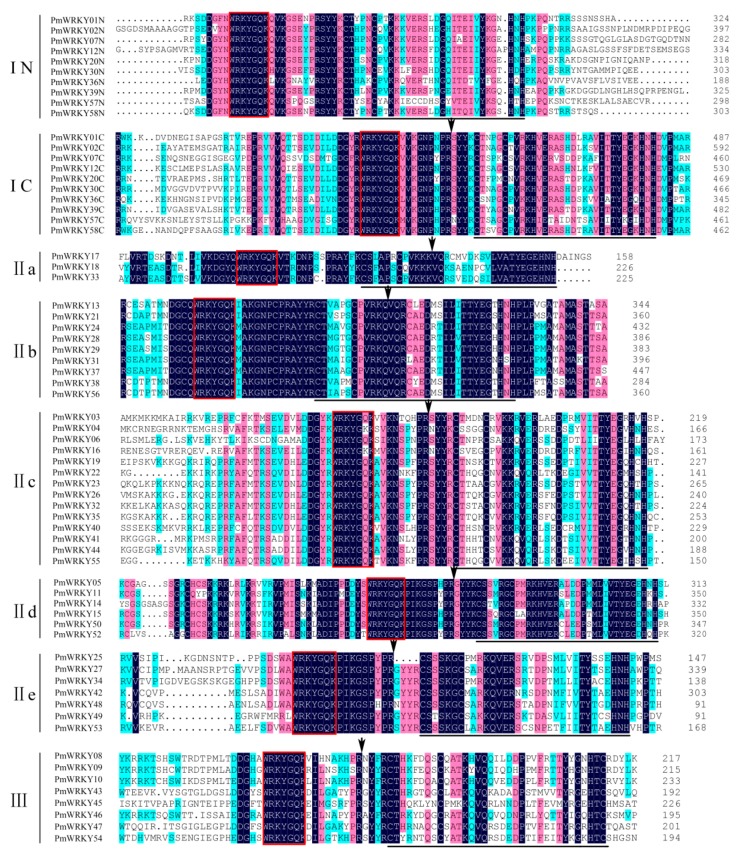
Acid residue alignment of WRKY domain. Alignment was performed using DNAMAN. The suffix ‘N’ and ‘C’ indicate the N-terminal WRKY domain and the C-terminal WRKY domain, respectively. The amino acids forming the zinc-finger motif are underlined in black. The conserved heptapeptide is surrounded by red box. The position of a conserved intron was indicated by an arrow head. The color shade of the amino acid residues highlighted the homology level: dark blue = 100%, pink ≥ 75%, and cambridge blue ≥ 50%.

The WRKY domain is an important functional and evolutionary unit of WRKY transcription factors and the conserved heptapeptide WRKYGQK near the N-terminal is regarded as the core sequence of genes; their variation often leads to a decline or loss of DNA binding activity, which means that *WRKY* gene mutations may no longer have the original biological function [6]. Multiple sequence alignment analysis using WRKY domains of PmWRKYs found that mutations only occurred in PmWRKY04 and PmWRKY16 of subgroup II c. The mutation sites were both changed from Q to K, which formed the WRKYGKK sequence (Figure 4). Interestingly, this type of mutation also occurred in the genome of *Populus trichocarpa*, *O. sativa* and *A*. *thaliana* [32,33,34]. We identified a CX_4_CX_22-23_HXH zinc finger motif in the N-terminal of subgroup I genes, a CX_4_CX_23_HXH motif in the C-terminal of subgroup I genes and II c genes, a CX_5_CX_23_HXH motif in II a, II b, II d and II e genes, and a CX_7_CX_23_HXC motif in subgroup III genes (Figure 4). From the multiple sequence alignment of the WRKY and zinc finger domains, we discovered that the homology of group I and II genes was higher than that of group III genes.

Through an analysis of the CDS and DNA sequences, we found that there were two types of introns in the WRKY domains. One is spliced exactly after the R position, similar to the splicing position found in *Arabidopsis* [7], and is designated as R-type intron. Another is spliced before the V position at the sixth AA after the second C residue of the C_2_H_2_-type zinc-finger motif [35]. We designate this type of intron as the V-type intron. Interestingly, the R-type intron is located before the zinc finger motif region in WRKY domains in subgroups I, II c, II d, II e and III, whereas the V-type intron is in subgroups II a and II b (Figure 4). 

### 3.5. Expression Profiles of PmWRKY Genes in Different Tissues

**Figure 5:**
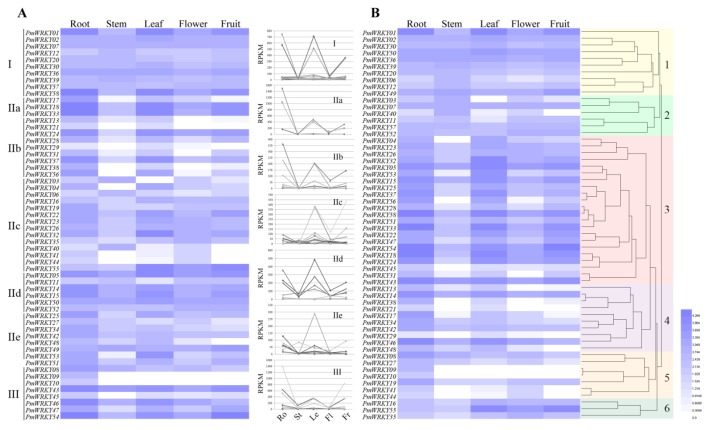
Expression patterns of *PmWRKYs* in five tissues. Transcriptome data was used to investigate expression profiles of *PmWRKY* genes. The colour scale represents RPKM expanded 25 times and then normalised log10 transformed counts. Light blue indicates low expression and dark blue indicate high expression. (**A**) Expression profiles related to subfamilies using heat map and line chart. (**B**) Hierarchical-clustering analysis of gene expression profiles.

According to the transcriptome data of five different organs (roots, stems, leaves, flowers and fruits) of *P. mume*, the heat map has been drawn by the RPKM values of *PmWRKY* genes (Figure 5 and Additional File 3). As shown in the expression patterns of *PmWRKYs* in different subgroups in Figure 5A, the expression of *WRKYs* exhibited a wide range of diversity in five tissues. Most *PmWRKYs* were expressed in the roots at a high expression level. The expression of *PmWRKY09* and *PmWRKY10* (which all belonged to group III) could not be detected in the stems, leaves, flowers or fruits, and 12 genes (*PmWRKY03*, *PmWRKY04*, *PmWRKY13*, *PmWRKY21*, *PmWRKY29*, *PmWRKY38*, *PmWRKY40*, *PmWRKY41*, *PmWRKY44*, *PmWRKY45*, *PmWRKY48* and *PmWRKY56*) lacked expression in one or two tissues. The expression levels of the other 44 genes were discovered in all of the detected tissues, which suggests that these *PmWRKYs* probably play vital roles in the developmental process of various tissues. We also hold the belief that the functions of duplicated genes diverged after the duplication event, which is supported by evidence that the expression patterns of several duplicated genes (*PmWRKY08*, *PmWRKY09* and *PmWRKY10*, *PmWRKY28* and *PmWRKY29*, *PmWRKY41* and *PmWRKY42*, and *PmWRKY21*, *PmWRKY38* and *PmWRKY56*) were quite different. In general, the group I genes had wider expression scopes than groups II and III genes. All of the genes in group I were expressed in all five tissues. However, a minority of groups II and III genes showed tissue-specific expression patterns. All of the subgroups II a and II b genes were hardly expressed in the stems, which meant that these genes might not participate in stem development. Furthermore, in the subgroups II d and II e, all of the members were expressed in five tissues, while *PmWRKY48* showed a tissue-specific expression pattern, which demonstrated the functional change of genes in the same subgroups during evolution. The similarity of the expression patterns of subgroup II a, II b and subgroup II d supported the phylogenetic analysis.

As shown in the hierarchical clustering in Figure 5B, 58 *PmWRKY* genes were classified into six clusters according to their expression patterns. It was found that the genes with a closer phylogenetic relationship were more likely to be clustered into the same group in the heat map. The finest examples are three subgroup II c genes (*PmWRKY16*, *PmWRKY35*, and *PmWRKY55*) in cluster 6 and four subgroup II c genes (*PmWRKY04*, *PmWRKY23*, *PmWRKY26*, and *PmWRKY32*) in a subcluster of cluster 3, which show similar expression patterns. Among these clusters, clusters 1 and 6 contained the genes whose transcripts were detected in every tissue. However, the genes in other clusters presented tissue-specific expression patterns: cluster 2 genes abundant in the stems and flowers, cluster 3 genes in the roots, leaves and fruits, cluster 4 genes in the roots and leaves, and cluster 5 genes in the roots.

### 3.6. Expression Analysis of PmWRKY Genes under Chilling Treatment

**Figure 6:**
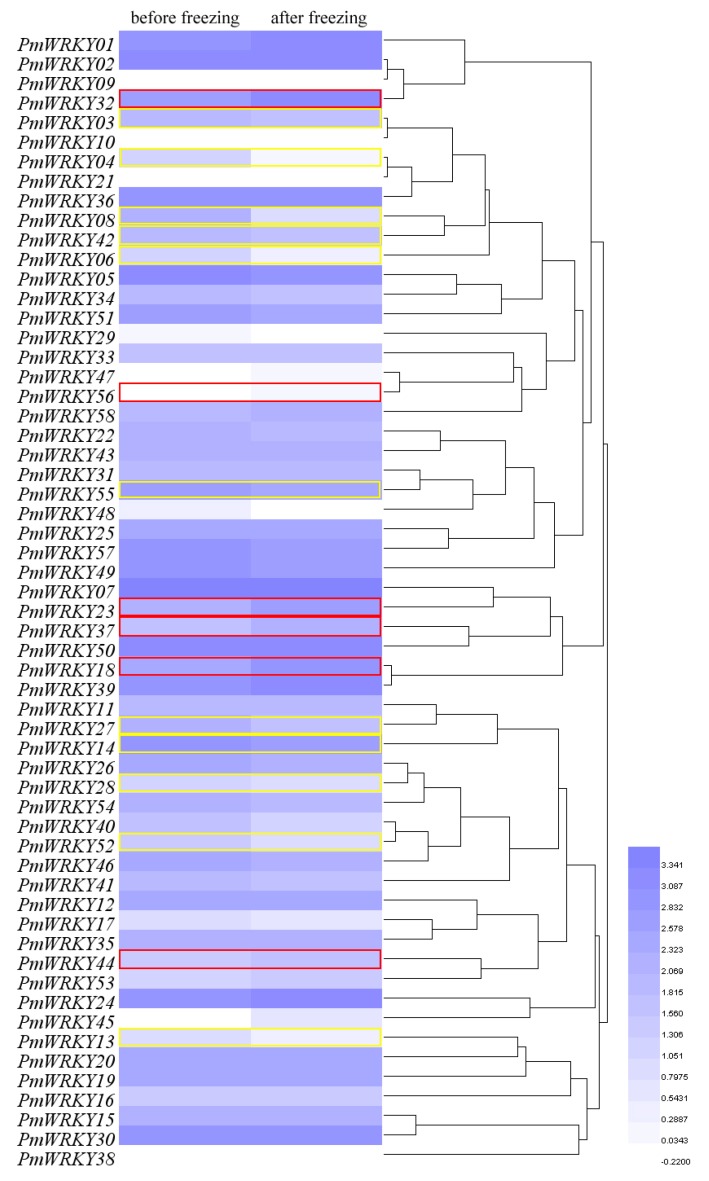
Hierarchical-clustering analysis of expression profiles of *PmWRKYs* in leaf buds of ‘Zhusha’ before and after freezing effect in winter. Transcriptome sequencing (RNA-seq) was performed to investigate expression profiles of *PmWRKY* genes. The colour scale represents RPKM expanded 25 times and then normalised log10 transformed counts. Light blue indicates low expression. Blue and dark blue indicate high expression. The genes which were up- or down-regulated over two fold were surrounded by red and yellow boxes, respectively.

To further examine the functioning of *PmWRKY* genes in cold tolerance, the expression patterns of *PmWRKY* genes before and after freezing stress in winter were analysed using transcriptome data. In Figure 6 and Additional File 4, after undergoing short freezing stress in *P*. *mume* ‘Zhusha’, 6 (*PmWRKY18*, *PmWRKY23*, *PmWRKY32*, *PmWRKY37*, *PmWRKY44* and *PmWRKY56*) and 11 (*PmWRKY03*, *PmWRKY04*, *PmWRKY06*, *PmWRKY08*, *PmWRKY13*, *PmWRKY14*, *PmWRKY27*, *PmWRKY28*, *PmWRKY42*, *PmWRKY52* and *PmWRKY55*) *PmWRKYs* were found up- or down-regulated over two-fold, respectively. Then, these 17 *PmWRKY* genes, which may play a role in the cold tolerance of *P. mume* in winter, were selected for further study.

**Figure 7:**
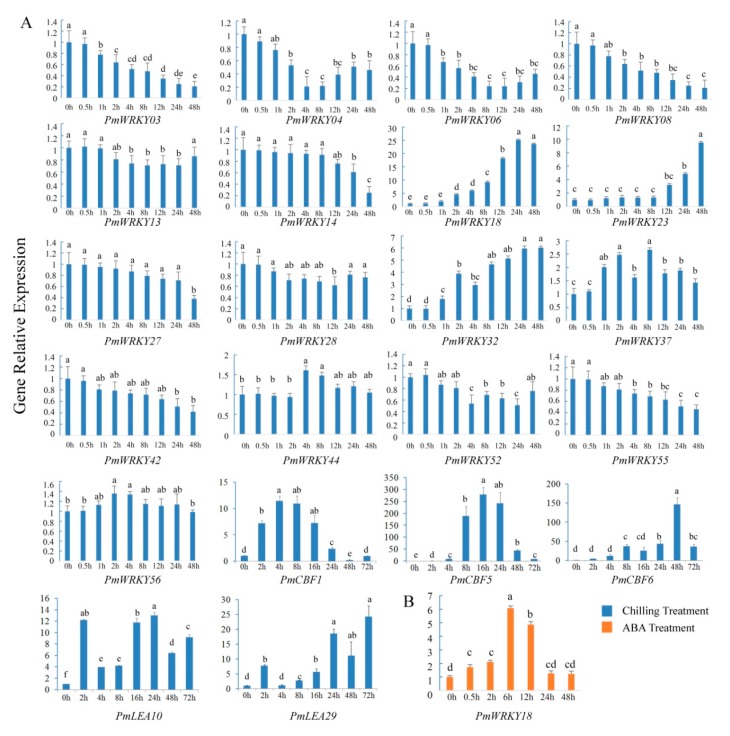
Expression patterns of 17 selected *PmWRKY* genes under artificial low temperature and exogenous ABA treatments. (**A**) The expression patterns of selected *PmWRKYs*, *PmCBFs* and *PmLEAs* under artificial low temperature treatments. (**B**) The expression pattern of *PmWRKY18* under exogenous ABA treatments. The transcript levels of the selected genes were assessed by qRT-PCR and normalised to *Actin* gene. Error bars are standard deviation of three technical replicates. Different letters indicate significant difference at level *p* = 0.05. Three independent experiments were performed with similar results.

To clarify the roles *WRKYs* played in cold resistance, the expression patterns of selected *PmWRKY* genes in different stages of artificial low temperature (4 °C) (0, 0.5, 1, 2, 4, 8, 12, 24 and 48 h) and exogenous ABA (100 μM) (0, 0.5, 2, 6, 12, 24 and 48 h) treatments were determined by qRT-PCR using cold resistant cultivar ‘Yudie’. As shown in Figure 7A, the expression of *PmCBF1*, *PmCBF5*, *PmCBF6* and dehydrin genes *PmLEA10* and *PmLEA29*, which were reported to be involved in cold resistance previously [36], was induced significantly after chilling treatment. The expression levels of 17 genes were changed with different patterns during artificial chilling treatment. The expression levels of *PmWRKY18*, *PmWRKY23* and *PmWRKY 32* were increased with prolonging of the treatment time in the cold. The largest increase of the expression level (approximately 25-fold) was detected in *PmWRKY18* after cold treatment for 24h. *PmWRKY23* and *PmWRKY32* also had the greatest up-regulation of more than 9.5- and 6-fold, respectively, at 48 h after being exposed to the cold condition. The three genes mentioned above were up-regulated continuously during low temperature treatment, whereas *PmWRKY37* showed an irregular pattern. The expression of *PmWRKY37* had fluctuations before and after it reached the maximum (nearly 2.7-fold) at 8 h. *PmWRKY04* and *PmWRKY06* had the greatest down-regulation of nearly 5- and 4-fold, respectively, and then slight up-regulation. The expression levels of six genes (*PmWRKY03*, *PmWRKY08*, *PmWRKY14*, *PmWRKY42* and *PmWRKY55*) gradually decreased over time and ultimately changed over 2-fold, but the expression levels of the other genes (*PmWRKY13*, *PmWRKY27*, *PmWRKY28*, *PmWRKY44,* and *PmWRKY56*) only changed slightly.

Based on previous research, the transcriptional regulation of plant cold resistance can usually be categorised into either ABA-independent or ABA-dependent signal pathways; the latter also response to dehydration stress [37]. To explain how the *PmWRKYs* respond to ABA and whether the *PmWRKYs* are involved in the ABA-dependent cold signaling pathway or not, annual branches of *P*. *mume* were treated with exogenous ABA. Among these 17 genes, only the expression level of *PmWRKY18* was continuously up-regulated, peaked (6-fold) at 6h and then gradually decreased during exogenous ABA treatment (Figure 7B), whereas that of the others were lower than 2-fold and had no remarkable changes throughout, which suggested that *PmWRKY18* may take part in cold adaptation in an ABA-dependent manner.

### 3.7. Expression Analysis of PmWRKY Genes in Winter

**Figure 8:**
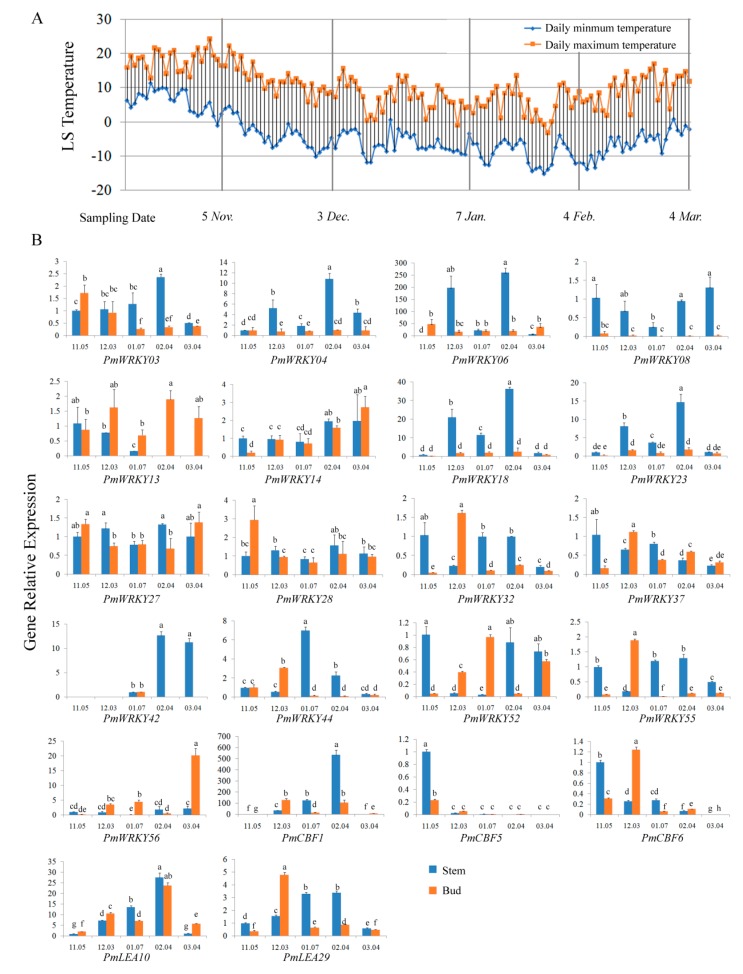
Expression patterns of 17 selected *PmWRKY* genes in winter. (**A**) The daily land surface temperature near the sampling sites in winter from the date 12 October, 2017 to 4 March, 2018. (**B**) The expression patterns of *PmWRKYs*, *PmCBFs* and *PmLEAs* in winter by qRT-PCR using *Actin* as reference gene. Different letters indicate significant difference at level *p* = 0.05. Three independent experiments were performed with similar results.

To verify the function of *PmWRKY* genes in cold tolerance, the expression patterns were analysed in winter using qRT-PCR. The daily land surface temperature was recorded from 12 Oct. to 4 Mar. As shown in Figure 8A, daily minimum and maximum temperatures below zero first appeared on 4 Nov. and 4 Jan., respectively, while the lowest temperature (−15.224 °C) was recorded on 26 January, after which the temperature began to increase with fluctuations. The stem and bud are the main aboveground organs of *P. mume* that overwinter. The expression of *PmCBFs* and *PmLEAs* was detected first (Figure 8B). The expression levels of *PmCBF1*, *PmLEA10* and *PmLEA29* in stems increased during winter and peaked on 4 February then decreased throughout the remaining winter, while the expression levels of *PmCBF5* and *PmCBF6* decreased during winter. The expression patterns differed in buds and stems. The expression patterns of *PmCBF1*, *PmCBF6*, *PmLEA10* and *PmLEA29* in buds were similar; they increased as the temperature decreased, peaking on 3 December or 4 February The expression pattern of *PmCBF5* in buds was similar in stems with both decreasing over winter. The expression patterns of *PmWRKY03* and *PmWRKY44* in stems were similar to those of *PmCBF1*, *PmLEA10* and *PmLEA29*, which increased over winter, peaked on 4 February and 7 January then decreased. In contrast, the expression levels of *PmWRKY08* and *PmWRKY52* decreased over winter, were at their lowest levels on 7 January, then increased. The expression levels of *PmWRKY32*, *PmWRKY37* and *PmWRKY55* first decreased then increased during winter, peaking on 7 January or 4 February, then decreasing. The expression patterns of *PmWRKY04*, *PmWRKY06*, *PmWRKY18*, *PmWRKY23*, *PmWRKY27* and *PmWRKY28* in stems were similar. Their expression levels increased as temperature decreased with two peaks on 3 December and 4 February The expression levels of *PmWRKY14* and *PmWRKY42* increased gradually while *PmWRKY13* decreased during winter. In addition, the expression of *PmWRKY56* in stems did not change significantly in winter. The expression of *PmWRKYs* in buds also had different patterns. The expression patterns of *PmWRKY23* and *PmWRKY37* were similar to *PmCBF1*, *PmCBF6*, *PmLEA10* and *PmLEA29* in buds, increasing during winter and peaking on 3 December and 4 February The expression pattern of *PmWRKY28* was similar to *PmCBF5* in buds, with the highest levels on 5 November then decreasing during winter. In contrast, the expression level of *PmWRKY14* increased during winter. The expression pattern of *PmWRKY32*, *PmWRKY42*, *PmWRKY44* and *PmWRKY55* were the same in buds. Their expression levels increased in winter time, and reached the highest peaks at 3 December or 7 January, and then decreased. On the contrary, the expression patterns of *PmWRKY03* and *PmWRKY27* decreased during winter and increased with increasing temperature. The expression patterns of *PmWRKY52* and *PmWRKY56* were the same, showing a fluctuating increase over winter. In addition, the expression levels of *PmWRKY04*, *PmWRKY06*, *PmWRKY08*, *PmWRKY13,* and *PmWRKY18* did not change significantly during winter. The results suggest that the transcription factor PmWRKYs may play an important role in overwintering survival of *P. mume* by different types of mechanisms. PmWRKY08 and PmWRKY28 may regulate negatively, while PmWRKY18, PmWRKY23 and PmWRKY44 may play a positively role in overwintering survival of *P. mume*.

## 4. Discussion

With the development of molecular biology, especially sequencing technology, the genomic sequencing of more and more plants has been completed, which provides convenient conditions for research from the genomic level in a systematic and global way. From the *P*. *mume* genome database-based analysis, 58 *PmWRKY* genes were identified (Table 1). Though 58 *PpWRKY* genes were reported in *P. persica* [16], these genes in two species do not exactly correspond one by one, *PmWRKY28* and *PmWRKY29* are the two closest homologues of *PpWRKY19*. The homologue of *PmWRKY51* was not described, and no homologous gene was found for *PmWRKY16* in *P. persica.* Gene duplication, which consists of whole-genome duplication, tandem repeat and segmental duplication, plays a vital role in the amplification and evolution of the plant gene family. The study on the *WRKY* gene family of *O*. *sativa* concluded that the production of 80% *OsWRKYs* resulted from gene duplication events [35]. However, as Figure 2 shows, there were only 16 *PmWRKYs* (27.6%) involved in seven gene duplication events, which is much lower than the rate of duplication genes in *O*. *sativa*. We speculated that the *PmWRKY* gene family may mainly originate from two reasons: One is that the formation of *PmWRKY* genes may not depend on gene duplication. Another, which occurs in the evolution process, is the loss-function or redundant genes in the *PmWRKY* gene superfamily may be lost in the genome to reduce energy consumption. It was shown that the gene duplication of *WRKYs* in *A*. *thaliana* and *O*. *sativa* mainly occurred in group III [35]; in contrast, most of the *PmWRKY* gene duplication events were found in group II (13/16). In addition, genomic localization indicated that 58 *PmWRKY* genes were unevenly mapped to all eight chromosomes (Figure 2). This indicated that the *WRKY* gene families in different species originated from different evolution patterns. 

The analysis of PmWRKY domain structure showed that there were two kinds of conserved introns in PmWRKY domains: R-type intron and V-type intron. The R-type introns only existed in the PmWRKY domains of subgroups I, II c, II d, II e and III, whereas the V-type introns were only detected in subgroups II a and II b and this was consistent with previous research on *O*. *sativa* [35]. In addition, another form of intron, which was located at the fourth AA residue (K) after the second C residue of the zinc-finger motif, was found in subgroups II a and II b of *A*. *thaliana* and *C*. *sativus* [7,38]. It is not clear whether or not the insertion of conserved introns in different loci affect the function of *WRKYs*. The heptapeptide sequence WRKYGQK is the conserved structure of *WRKY* genes. It can be found in multiple alignment analysis of WRKY domains in *P*. *mume*. Some mutations occurred in subgroup II c (Figure 4), which indicated that the selection pressure and evolution pattern of *PmWRKY* genes vary between subgroups. As is well known, theWRKY domain contains a zinc-finger motif C_2_H_2_ or C_2_HC besides the conserved heptapeptide WRKYGQK [7]. Our study demonstrated that the zinc-finger motif C_2_HC only existed in the PmWRKY domains of group III whereas C_2_H_2_ was owned by PmWRKY domains of other groups. We found that this result is slightly different from that of previous research on *O*. *sativa* [35], which announced that zinc-finger motif C_2_H_2_ and C_2_HC simultaneously occurred in the N-termial of OsWRKYs of subgroup I.

RNA-seq data and qRT-PCR analysis provide a good clue to identify the important *PmWRKY* genes in cold resistance of *P. mume*. In our study, the expression of some *PmWRKYs* was changed under cold stress in *P. mume* cultivar ‘Zhusha’ based on the RNA-Seq (Figure 6). There were 6 and 11 *PmWRKYs* that were found to be up- or down-regulated over two-fold, respectively, after being subjected to cold stress in winter. Since some factors were uncontrolled during winter time, artificial low temperature treatment was performed to verify the cold response of *PmWRKYs*. Based on the qRT-PCR results, the expression levels of the 12 selected *PmWRKYs* were significantly different and 5 slightly changed under the artificial chilling treatment (Figure 7A). In addition, spatiotemporal expression patterns of the 17 *PmWRKYs* candidate genes varied during winter. The expression patterns of most *PmWRKYs* differed in stems and buds, which may result from the asynchronous icing of the organs (Figure 8B). However some factors, for example developmental stage, air humidity and day length, which were uncontrollable in winter time, may also affected gene expression. The results suggest that these genes potentially took part in the cold resistance of *P. mume*. Bud dormancy is the important way plants in *Prunus* genus respond to the cold winter. It was reported that PpWRKY11 (Prupe.1G071400), PpWRKY18 (Prupe.1G393000), PpWRKY33 (Prupe.6G286000), PpWRKY46 (Prupe.2G185100), PpWRKY48 (Prupe.1G114800) and PpWRKY53 (Prupe.2G307400) may play a role in bud dormancy in *P. persica* (Table 1) [16]. *PmWRKY18*, *PmWRKY55* and *PmWRKY27*, the homologous genes of *PpWRKY11*, *PpWRKY33* and *PpWRKY48* in *P. mume*, showed low temperature response in different degrees. These genes may enhance cold tolerance of *P. mume* by regulating bud dormancy in a low temperature induction pattern.

Low temperature signal transduction pathways are mainly categorized into either ABA-dependent or ABA-independent manners [37], both of which ultimately regulate the expression of functional genes, *COR47*, *RD29A* and *KIN1*, and enhance the chilling tolerance of plants. WRKY transcription factors have been reported to be key nodes in ABA signalling [39]. It is reported that *AtWRKY18*, *AtWRKY40* and *AtWRKY60* genes in *A*. *thaliana* can be induced by ABA and act as negative regulation factors in the ABA signaling pathway [40,41]. *AtWRKY6*, the homologous gene of *PmWRKY37*, functioned as a positive regulator of ABA signalling during the germination process [42]. In this work, we detected the response of 17 selected genes to exogenous ABA treatment. The results showed that only *PmWRKY18* was significantly up-regulated after ABA treatment (Figure 7B); whereas other genes had no significant changes compared with the control (data were not shown). We speculated that *PmWRKY18* may take a part in an ABA-dependent cold signaling pathway.

## 5. Conclusions

Although cold tolerance related *WRKY* genes were studied in various species of plants, the cold signal response mechanism of *WRKY* genes and the down-stream target genes are mostly unclear. Research on the molecular biological function of *PmWRKY* genes in response to low temperature needs to be conducted. In this study, the characterisation and the expression analysis of *PmWRKYs* can create a foundation for further gene isolation and function analysis to clarify the role of *WRKY* genes in the cold resistance of *P. mume*.

## Figures and Tables

**Table 1:** Characteristics of the *WRKY* genes identified in *Prunus mume*.

Name	Gene ID	Locus	Protein Length (aa)	MW (kDa)	pI	Localization	EST Number	WRKY Domain			Subgroup	Homolog in *P. persica*
								Conserved Heptapeptide	Zinc Finger Motif	Domain Number		
*PmWRKY01*	LOC103330550	Pa1:1070280:1072953	590	64.75	6.98	Nucleus	1	WRKYGQK	C2H2	2	I	*PpWRKY6*
*PmWRKY02*	LOC103344588	Pa1:1909602:1912937	740	80.02	5.65	Nucleus	0	WRKYGQK	C2H2	2	I	*PpWRKY5*
*PmWRKY03*	LOC103331503	Pa1:13787725:13788689	239	27.20	9.03	Nucleus	0	WRKYGQK	C2H2	1	IIc	*PpWRKY34*
*PmWRKY04*	LOC103331584	Pa1:13972916:13975438	197	22.17	6.20	Nucleus	0	WRKYGKK	C2H2	1	IIc	*PpWRKY35*
*PmWRKY05*	LOC103337527	Pa1:19841802:19843058	326	35.77	9.60	Nucleus	1	WRKYGQK	C2H2	1	IId	*PpWRKY41*
*PmWRKY06*	LOC103339250	Pa1:20889375:20891243	297	33.36	5.03	Nucleus	0	WRKYGQK	C2H2	1	IIc	*PpWRKY36*
*PmWRKY07*	LOC103340389	Pa1:21713743:21716122	479	52.28	8.91	Nucleus	0	WRKYGQK	C2H2	2	I	*PpWRKY7*
*PmWRKY08*	LOC103342893	Pa1:22703478:22705201	335	37.41	5.76	Nucleus	0	WRKYGQK	C2HC	1	III	*PpWRKY58*
*PmWRKY09*	LOC103343421	Pa1:22717809:22719867	337	38.22	5.50	Nucleus	0	WRKYGQK	C2HC	1	III	*PpWRKY57*
*PmWRKY10*	LOC103343430	Pa1:22722643:22724633	340	37.98	5.67	Nucleus	0	WRKYGQK	C2HC	1	III	*PpWRKY56*
*PmWRKY11*	LOC103318654	Pa1:25540026:25541291	354	40.21	9.68	Nucleus	0	WRKYGQK	C2H2	1	IId	*PpWRKY42*
*PmWRKY12*	LOC103318792	Pa1:26409186:26413001	733	80.43	5.88	Nucleus	0	WRKYGQK	C2H2	2	I	*PpWRKY9*
*PmWRKY13*	LOC103319105	Pa2:1797770:1800086	562	62.09	5.17	Nucleus	0	WRKYGQK	C2H2	1	IIb	*PpWRKY15*
*PmWRKY14*	LOC103320106	Pa2:8913661:8915220	342	37.88	9.48	Nucleus	0	WRKYGQK	C2H2	1	IId	*PpWRKY39*
*PmWRKY15*	LOC103320368	Pa2:10410393:10411854	364	39.89	9.29	Nucleus	1	WRKYGQK	C2H2	1	IId	*PpWRKY38*
*PmWRKY16*	LOC103320597	Pa2:11611164:11611850	162	18.82	5.35	Nucleus	0	WRKYGKK	C2H2	1	IIc	*-*
*PmWRKY17*	LOC103320740	Pa2:12418544:12419976	285	31.68	8.33	Nucleus	0	WRKYGQK	C2H2	1	IIa	*PpWRKY12*
*PmWRKY18*	LOC103320741	Pa2:12424777:12426189	323	36.40	7.62	Nucleus	1	WRKYGQK	C2H2	1	IIa	*PpWRKY11/* *Prupe.1G071400*
*PmWRKY19*	LOC103321497	Pa2:16703139:16705510	330	36.38	6.00	Nucleus	0	WRKYGQK	C2H2	1	IIc	*PpWRKY24*
*PmWRKY20*	LOC103321524	Pa2:16839818:16842349	517	56.52	6.74	Nucleus	0	WRKYGQK	C2H2	2	I	*PpWRKY1*
*PmWRKY21*	LOC103321616	Pa2:17544191:17547389	643	70.06	6.43	Nucleus	0	WRKYGQK	C2H2	1	IIb	*PpWRKY14*
*PmWRKY22*	LOC103322097	Pa2:20999398:21001199	162	18.46	9.67	Nucleus	0	WRKYGQK	C2H2	1	IIc	*PpWRKY23*
*PmWRKY23*	LOC103323186	Pa2:32805980:32807604	390	43.02	5.82	Nucleus	0	WRKYGQK	C2H2	1	IIc	*PpWRKY22*
*PmWRKY24*	LOC103324471	Pa3:408956:411939	591	64.44	7.06	Nucleus	1	WRKYGQK	C2H2	1	IIb	*PpWRKY20*
*PmWRKY25*	LOC103324889	Pa3:2860917:2862029	280	30.55	5.45	Nucleus	0	WRKYGQK	C2H2	1	IIe	*PpWRKY47*
*PmWRKY26*	LOC103325015	Pa3:3723897:3725432	340	37.69	6.55	Nucleus	0	WRKYGQK	C2H2	1	IIc	*PpWRKY32*
*PmWRKY27*	LOC103325306	Pa3:5613186:5615990	515	55.88	6.07	Nucleus	0	WRKYGQK	C2H2	1	IIe	*PpWRKY48/* *Prupe.1G114800*
*PmWRKY28*	LOC103326420	Pa3:13640842:13643362	547	59.91	6.13	Nucleus	0	WRKYGQK	C2H2	1	IIb	*PpWRKY19*
*PmWRKY29*	LOC103326493	Pa3:13656379:13658843	544	59.51	6.02	Nucleus	0	WRKYGQK	C2H2	1	IIb	*PpWRKY19*
*PmWRKY30*	LOC103326638	Pa3:15399255:15407416	884	98.18	6.98	Nucleus	0	WRKYGQK	C2H2	2	I	*PpWRKY4*
*PmWRKY31*	LOC103327251	Pa4:195608:198391	616	67.23	6.42	Nucleus	0	WRKYGQK	C2H2	1	IIb	*PpWRKY16*
*PmWRKY32*	LOC103327303	Pa4:564486:565902	321	35.77	6.51	Nucleus	0	WRKYGQK	C2H2	1	IIc	*PpWRKY29*
*PmWRKY33*	LOC103328175	Pa4:6725543:6727134	320	35.25	8.71	Nucleus	1	WRKYGQK	C2H2	1	IIa	*PpWRKY13*
*PmWRKY34*	LOC103328332	Pa4:8491309:8494074	268	29.42	5.37	Nucleus	0	WRKYGQK	C2H2	1	IIe	*PpWRKY46/* *Purpe.2G185100*
*PmWRKY35*	LOC103328791	Pa4:14169139:14171554	367	41.67	7.10	Nucleus	1	WRKYGQK	C2H2	1	IIc	*PpWRKY30*
*PmWRKY36*	LOC103329228	Pa4:16910810:16913287	486	53.04	5.90	Nucleus	0	WRKYGQK	C2H2	2	I	*PpWRKY2*
*PmWRKY37*	LOC103329304	Pa4:17519417:17521899	649	70.74	6.11	Nucleus	0	WRKYGQK	C2H2	1	IIb	*PpWRKY17*
*PmWRKY38*	LOC103329978	Pa4:21736698:21738977	499	54.64	6.71	Nucleus	0	WRKYGQK	C2H2	1	IIb	*PpWRKY18/* *Prupe.1G393000*
*PmWRKY39*	LOC103330053	Pa4:22207825:22211049	533	58.44	8.45	Nucleus	0	WRKYGQK	C2H2	2	I	*PpWRKY3*
*PmWRKY40*	LOC103331676	Pa5:13956787:13959777	244	27.79	7.29	Nucleus	0	WRKYGQK	C2H2	1	IIc	*PpWRKY26*
*PmWRKY41*	LOC103332060	Pa5:16864359:16866293	221	24.84	9.24	Nucleus	0	WRKYGQK	C2H2	1	IIc	*PpWRKY27*
*PmWRKY42*	LOC103332064	Pa5:16887019:16890355	417	46.73	7.72	Nucleus	0	WRKYGQK	C2H2	1	IIe	*PpWRKY44*
*PmWRKY43*	LOC103332261	Pa5:18120250:18122148	357	39.80	4.90	Nucleus	1	WRKYGQK	C2HC	1	III	*PpWRKY51*
*PmWRKY44*	LOC103332696	Pa5:20443902:20444645	209	24.04	9.05	Nucleus	0	WRKYGQK	C2H2	1	IIc	*PpWRKY28*
*PmWRKY45*	LOC103333076	Pa5:21989132:21991115	344	37.86	5.78	Nucleus	0	WRKYGQK	C2HC	1	III	*PpWRKY52*
*PmWRKY46*	LOC103333154	Pa5:23138449:23140440	323	36.46	5.70	Nucleus	0	WRKYGQK	C2HC	1	III	*PpWRKY53/* *Purpe.2G307400*
*PmWRKY47*	LOC103333772	Pa5:25721161:25722357	332	36.12	5.32	Nucleus	0	WRKYGQK	C2HC	1	III	*PpWRKY54*
*PmWRKY48*	LOC103333707	Pa5:25986620:25987499	228	24.63	4.92	Nucleus	0	WRKYGQK	C2H2	1	IIe	*PpWRKY45*
*PmWRKY49*	LOC103334065	Pa6:994260:995360	242	28.23	6.02	Nucleus	0	WRKYGQK	C2H2	1	IIe	*PpWRKY50*
*PmWRKY50*	LOC103334153	Pa6:1797284:1799454	355	40.24	9.61	Nucleus	0	WRKYGQK	C2H2	1	IId	*PpWRKY43*
*PmWRKY51*	LOC103335291	Pa6:8182262:8186226	471	52.51	7.88	Chlo, Nucl	0	WRKYGQK	C2H2	1	IIx	*ppa024204*
*PmWRKY52*	LOC103337200	Pa7:5945463:5947021	330	37.05	9.68	Nucleus	0	WRKYGQK	C2H2	1	IId	*PpWRKY40*
*PmWRKY53*	LOC103337694	Pa7:9472026:9473037	291	32.89	5.23	Nucleus	0	WRKYGQK	C2H2	1	IIe	*PpWRKY49*
*PmWRKY54*	LOC103337660	Pa7:10206830:10208250	356	40.00	5.41	Nucleus	0	WRKYGQK	C2HC	1	III	*PpWRKY55*
*PmWRKY55*	LOC103338090	Pa7:12882417:12883702	170	19.52	9.35	Nucleus	0	WRKYGQK	C2H2	1	IIc	*PpWRKY33/* *Prupe.6G286000*
*PmWRKY56*	LOC103338408	Pa7:14366251:14369593	683	73.58	6.45	Nucleus	0	WRKYGQK	C2H2	1	IIb	*PpWRKY21*
*PmWRKY57*	LOC103341266	Pa8:16767252:16770984	537	59.39	5.47	Nucleus	0	WRKYGQK	C2H2	2	I	*PpWRKY10*
*PmWRKY58*	LOC103342913	scaffold22:94704:96740	536	59.89	6.82	Nucleus	1	WRKYGQK	C2H2	2	I	*PpWRKY8*

## Supplementary Materials

The following are available online at https://www.mdpi.com/2073-4425/10/11/911/s1, Additional File 1: Primers used for qRT-PCR, Additional File 2: Conserved motifs predicted by MEME program in *Prunus mume* WRKY proteins, Additional File 3: The RPKM values of *PmWRKY* genes in different tissues, Additional File 4: The RPKM values of *PmWRKY* genes in leaf buds of ‘Zhusha’ before and after freezing effect in winter, Additional File 5: Supplementary Data 1: The CDS sequences of 58 *P*. *mume WRKY* genes, Supplementary Data 2: The genomic sequences of 58 *P*. *mume WRKY* genes, Supplementary Data 3: The protein sequences of 58 *P*. *mume WRKY* genes and 12 *Arabidopsis thaliana WRKY* genes.
