# Supplementary material for: Genome-Wide Analysis of Members of the WRKY Gene Family and Their Cold Stress Response in Prunus mume
Source: Genes (Basel). 2019 Nov 8;10(11):911. doi: 10.3390/genes10110911 (PMC6896039; doi:10.3390/genes10110911)
Supplement: Supplementary file 1 [file genes-10-00911-s001.pdf]

Supplementary Materials

Additional file 1

Primers used for qRT-PCR

Gene	Forward primer	Reverse primer
PmWRKY03	5'-ACCCTACTCCCATCACTCACT-3'	5'-TCTCCCCATGCCCAGAGATT-3'
PmWRKY04	5'-ATCGGAGCTGGAGGTTATGG-3'	5'-TTGCACCCTCCACTTGAACA-3'
PmWRKY06	5'-CCCAAGGTGCAGTGCTAAGA-3'	5'-GGCCTGATTGTTGAGTGGGA-3'
PmWRKY08	5'-GACACTCCGGCTTTGACTGA-3'	5'-CCTAAAGTAGTTCCTTGGGTGCT-3'
PmWRKY13	5'-ACGGATCAAGAGCTATGCGT-3'	5'-GGTTGCTTTGGTGAAGAGCC-3'
PmWRKY14	5'-CTCACCCTACCCAAGGGGATA-3'	5'-GATCAGCATCGCCGGATCAT-3'
PmWRKY18	5'-CCCAAGAGAACATCGCCAGT-3'	5'-ACTGTTGAAAAGCCGGTGGA-3'
PmWRKY23	5'-ACAACATACGAAGGCCAGCA-3'	5'-GATGAGGGGAAGGCACAAGG-3'
PmWRKY27	5'-CATCAAGCGAAGGAAGAGCC-3'	5'-TGCCCACAGATCAGATGGAAC-3'
PmWRKY28	5'-CCACAACCATCCACTCCCTC-3'	5'-GCACTAGGCATTGAGCCTGA-3'
PmWRKY32	5'-GAAGCGAGTGGAGCGATCTT-3'	5'-CGAGGTATGAGTGGGCTTGG-3'
PmWRKY37	5'-AAGGCAATCACAACCACCCA-3'	5'-GACCCCGAGAGTAGCATTCG-3'
PmWRKY42	5'-ATGAAGAAAGTGGGGACAGGG-3'	5'-TGTAGGCCAGGTTGTGCATT-3'
PmWRKY44	5'-TTGATGATGGCTACCGCTGG-3'	5'-AACGACGATGCTTGTGTCCT-3'
PmWRKY52	5'-GCCCTGCAAGAAAGCATGTC-3'	5'-TCTTGGGGTGTTGATGGTCG-3'
PmWRKY55	5'-TGATGATGGGTACCGATGGC-3'	5'-GCTTTTTGACAACGCACCCT-3'
PmWRKY56	5'-GGCTGGTGGTGGTGACAATA-3'	5'-CCCTATCTGCGTTTGGAGGT-3'
PmCBF1	5'-CAACAAGAAGACCAGGATT-3'	5'-AAGTTCAAGCAAGCAGAC-3'
PmCBF5	5'-CTCCATTGCCTCTTATCCA-3'	5'-TTCTCTTGCTTCTTCTGACT-3'
PmCBF6	5'-GTGGAAGAGAAGAAGAAGAAG-3'	5'-GTCAATCAACCTTGGCATAT-3'
PmLEA10	5'-TACAACTGCCACCACCACACCTTA-3'	5'-AGTGTTGTCCGGGAAGTTTCTCCT-3'
PmLEA29	5'-ATGCTGAGCCTGCAGTAGTAGGTT-3'	5'-TCCTTCTCCTTGATGGCCTCCTTT-3'
PmACTIN	5'-CCCTAAGGCTAACAGAGAAAAGA-3'	5'-CAGCAAGGTCCAGACGAAGAAT-3'

Additional file 2

Conserved motifs predicted by MEME program in *P. mume* WRKY proteins

Motif	Width	Best possible match
1	26	DDGYRWRKYGQKVVKGSPYPRSYYRC
2	29	GCPVRKQVZRSSEDPSILITTYEGEHNHP
3	29	HPNCPVKKKVERSLDGQITEIIYKGEHNH
4	63	MLTEDGHAWRKYGQKEILNAKHPRNYYRCTHKFDQSCQATKQVQQIZDDPPJFRTTYYGNHTC
5	15	EPRVAVQTRSEVDIL
6	37	ELAVLQAELGRVNEENQRLKEMLSQVTKNYQALQMQL
7	27	VEAATAAITADPNFTAALAAAITSIIG
8	21	YSWRKYGQKPIKGSPYPRSYY
9	29	LPPMATAMASTTSAAASMLLSGSSTSADG
10	30	GRCHCSKKRKLRVKRSIRVPMISNKLADIP
11	113	TPEQHKMNNNGSVVVEEKQMMNGFNHIVPRQFMDMGRAEKDERSQCPLEGCRSQDCSGSPPRNDIVESMECCKSTSHVLHRDLSGRISTTNGGEDSPDQE
12	37	RSPYLTIPPGLSPTELLDSPVLLSSSNALPSPTTGSF
13	7	HHQQQHQ
14	21	LATLSASAPFPTITLDLTRSP
15	18	DKDRRGSYKKRKTSHSWT
16	55	TAVDHDHHGKRIVDELDFFADNKGRLMEMRDQTVEVKEEGAHDHHGVGQEKQLPD
17	20	EELVDKAVSSFKKVLSLLNG
18	29	RDYLKASELILDCTSPRESSNFIRFGDTK
19	41	PNQLPPSFPGNMMPLPQIFGQALYNQSKFSGLZSFQGLDGA
20	17	EEGDEDEPESKRRKIEV

Additional file 3

The RPKM values of *PmWRKY* genes in different tissues

Gene	Root_RPKM	Stem_RPKM	Leaf_RPKM	Flower_RPKM	Fruit_RPKM
PmWRKY01	571.3452853	16.21669951	712.7492726	63.01261693	362.7986276
PmWRKY02	40.85797138	36.33093366	65.78550055	27.83845093	42.02976848
PmWRKY03	5.072884776	34.0938205	0	4.256376479	0.353619091
PmWRKY04	30.71470125	0	82.14500298	4.456217575	1.608550141
PmWRKY05	230.6023513	65.35487562	485.298879	106.627799	206.6934226
PmWRKY06	2.148184687	14.78460072	2.290604061	0.612588114	1.908514668
PmWRKY07	32.16485355	37.72833294	23.34178196	37.20749596	26.77805123
PmWRKY08	131.6293397	23.58552974	9.877524779	10.95669752	3.791582475
PmWRKY09	11.19665562	0	0	0	0
PmWRKY10	3.257666307	0	0	0	0
PmWRKY11	4.879422648	7.978329802	0.596736289	5.827927482	6.728723828
PmWRKY12	3.899029234	11.14743931	4.810203962	3.440452862	5.547901332
PmWRKY13	13.31017117	0.508155638	5.100581935	0	0.121222886
PmWRKY14	356.0891503	37.11684338	171.7651378	36.72313934	86.48787546
PmWRKY15	197.7219651	32.4498445	277.7475904	37.59388918	128.7683736
PmWRKY16	6.353075968	1.579647005	34.80148138	13.99932959	16.46895946
PmWRKY17	164.6480616	0.100032036	10.5344588	1.489345927	0.159087377
PmWRKY18	1039.619205	6.357591649	400.1448336	89.96095939	192.5983468
PmWRKY19	50.79634083	2.333677902	2.204458831	5.607062805	7.079147944
PmWRKY20	17.26524769	11.26692883	7.952003847	2.936796298	9.047096407
PmWRKY21	2.309671351	0	1.315784984	0	0.070650605
PmWRKY22	89.75163685	6.845137021	111.0470505	12.13275231	157.0133847
PmWRKY23	56.19569698	4.682829655	45.26988339	22.95508145	11.28747315
PmWRKY24	363.2698985	2.223009242	206.0761797	64.34520689	145.9887347
PmWRKY25	127.8441953	1.832615387	60.98083292	8.553706627	19.26825542
PmWRKY26	25.73004235	3.691504829	35.75556733	29.88986872	11.20796227
PmWRKY27	17.44163358	4.768193737	0.912322922	4.717086767	0.52905802
PmWRKY28	30.71608945	0.417652006	20.23059431	1.054888656	18.93023659
PmWRKY29	0.483660362	0	0.777399569	0.111652145	0
PmWRKY30	21.35990936	21.33564655	36.30426706	10.10735778	18.35382974
PmWRKY31	15.0137404	0.18547269	3.242664778	0.049311523	4.055825662
PmWRKY32	88.35223824	4.264719864	378.5805797	42.42521469	68.31913515
PmWRKY33	1503.14785	6.238758161	484.3974291	39.14520743	325.79261
PmWRKY34	61.24416762	3.296966673	9.362671593	2.37520224	1.099417967
PmWRKY35	3.22330717	1.088392049	6.779947628	22.81890724	10.07654256
PmWRKY36	46.5872472	55.39720773	82.11693107	24.05278381	62.03558349
PmWRKY37	231.0945156	1.936620226	203.5487882	7.255242301	40.73909539
PmWRKY38	31.89498259	0	1.883034511	0	0.272993938
PmWRKY39	14.8087021	23.57309263	21.28992671	8.717335348	22.11046409
PmWRKY40	1.306447014	27.5582136	0.288219568	1.986952467	0

PmWRKY41	4.579834125	0	0.212053436	2.055757409	0
PmWRKY42	64.41228696	6.981183175	20.72238936	8.297784449	6.258832315
PmWRKY43	641.5264483	122.0284665	357.0796239	47.59250671	343.2758971
PmWRKY44	2.600085723	0	0.112085388	2.318111211	0
PmWRKY45	2.947023698	0	0.750484769	0.529134081	1.120989601
PmWRKY46	510.8603931	42.29564445	341.2273572	55.12221624	16.92169216
PmWRKY47	72.37268741	0.945047407	39.86604596	4.202881813	76.4465007
PmWRKY48	14.47058701	1.998893444	18.39864506	0.53144471	0
PmWRKY49	32.6975446	83.70829005	43.49189792	23.9144652	93.15122376
PmWRKY50	50.45536359	77.14830316	125.6237911	47.34709591	73.16902135
PmWRKY51	59.59606186	3.636757935	43.98401586	4.898974435	32.48550747
PmWRKY52	20.81910498	9.161846576	5.831149166	40.62822557	25.01757137
PmWRKY53	80.40676054	0.195953167	290.1130653	2.083918469	7.635104435
PmWRKY54	1384.532462	18.91249953	184.9408895	49.25986324	833.829524
PmWRKY55	9.57926319	3.513405912	385.5540691	117.6085589	435.3002757
PmWRKY56	106.4454332	0	61.70103944	0.133443902	3.059873576
PmWRKY57	14.55861356	19.09050058	8.618907962	18.32298871	20.80437075
PmWRKY58	744.7134373	17.74087726	515.029225	50.08544754	352.5536241

Additional file 4

The RPKM values of *PmWRKY* genes in leaf buds of 'Zhusha' before and after freezing effect in winter

Name	'Zhusha'		
	ZS_bef_RPKM	ZS_aft_RPKM	log2(aft/bef)
PmWRKY01	35.08319782	68.06779948	0.956192216
PmWRKY02	50.65246531	84.19881327	0.733167411
PmWRKY03	4.292187655	1.832817527	-1.227649992
PmWRKY04	0.492211656	0.06160463	-2.998168146
PmWRKY05	64.18959303	44.24003651	-0.536986838
PmWRKY06	0.613199924	0.08186387	-2.905058742
PmWRKY07	116.010442	156.410304	0.431080892
PmWRKY08	4.749622745	0.326724553	-3.861666146
PmWRKY09	0	0	-
PmWRKY10	0	0	-
PmWRKY11	4.152255936	3.332897224	-0.317118541
PmWRKY12	12.99549419	9.007033271	-0.528887602
PmWRKY13	0.259656948	0.108327857	-1.261202552
PmWRKY14	35.58776376	17.63868063	-1.012638629
PmWRKY15	5.807423281	7.38546679	0.346790917
PmWRKY16	1.42001553	0.823158792	-0.786664041
PmWRKY17	0.255571437	0.213246794	-0.261202552
PmWRKY18	12.520634	43.06848099	1.782324824
PmWRKY19	11.44615157	9.876096859	-0.212849726
PmWRKY20	12.95832704	12.38609837	-0.065157665
PmWRKY21	0	0	-
PmWRKY22	5.082160843	4.040961342	-0.330743485
PmWRKY23	6.480576998	21.18222404	1.7086599
PmWRKY24	40.3330869	64.12042938	0.66882026
PmWRKY25	14.30654342	14.19449588	-0.011343535
PmWRKY26	9.574310603	5.615957516	-0.769636588
PmWRKY27	5.453676534	2.151147703	-1.342122547
PmWRKY28	0.755832315	0.311620498	-1.278276065
PmWRKY29	0.044705462	0	-
PmWRKY30	36.39529782	39.91478802	0.133171279
PmWRKY31	4.580679627	3.341027735	-0.455269705
PmWRKY32	22.51065808	54.28362717	1.26990889
PmWRKY33	2.504759316	2.127950567	-0.235207344
PmWRKY34	3.622970555	1.859131533	-0.962544236
PmWRKY35	6.951820878	6.993799489	0.008685525
PmWRKY36	37.72241405	29.75541555	-0.342269739
PmWRKY37	2.380221982	6.098858322	1.357443075
PmWRKY38	0	0	-

PmWRKY39	38.69115446	54.04456475	0.482145758
PmWRKY40	1.44197925	0.746798978	-0.949258546
PmWRKY41	3.29249689	1.868118765	-0.817595901
PmWRKY42	4.313328461	1.92595526	-1.163227392
PmWRKY43	5.989033448	5.928499151	-0.014656269
PmWRKY44	1.10220253	2.381458964	1.111456345
PmWRKY45	0	0.141422802	-
PmWRKY46	12.25743749	7.416512897	-0.724844481
PmWRKY47	0	0.073259559	-
PmWRKY48	0.106395096	0	-
PmWRKY49	41.91091102	26.30289515	-0.672104277
PmWRKY50	62.75906009	72.60382463	0.210221803
PmWRKY51	15.74399466	9.199972723	-0.775100148
PmWRKY52	1.030521685	0.331659969	-1.635598067
PmWRKY53	0.500639938	0.960779051	0.940431309
PmWRKY54	5.39157894	3.655898266	-0.560481908
PmWRKY55	24.1507535	11.34173653	-1.090426654
PmWRKY56	0.04062058	0.071331676	1.001831854
PmWRKY57	28.05537824	26.57197752	-0.078371764
PmWRKY58	3.176002586	4.906344125	0.627436339

Additional file 5

Supplementary Data 1. The CDS sequences of 58 *P.mume WRKY* genes

>PmWRKY01

ATGGCCTCCTCTTCTGGGAGCTTAGACACCTCTGCAAATTCACACCCAACCTTCACTTTCTCGACGCAC
CCTTTCATGACCACCTCCTTCTCTGACCTCTTAGCCTCTGGCACAGATGAAGACCCCAGCACCAACAC
CACAGTTCAACATGTTGGCCATGGCGGGCTAGCAGATCGAATAGCAGAACGTACTGGGTCTGGTGTGC
CCAAATTCAAGTCTCTTCCTCCTCCTTCGCTGCCCATTTCTCCTCCCTCCGTTTCTCCCTCTTCTTACTTC
GCAATCCCAGCTGGGTTGAGCCCAGCTGAGCTTCTCGACTCTCCTGTCCTCCTAAGCACTTCAAACAT
TCTTCCATCTCCAACTACCGGAAGTTTTGCAGCTCAGGCCTTTTGGAAGGCCAATTCTGGAAACAACC
AGCAGATTGTTAAACACGAAAGCAAGAACTACTCAGATTTCTCTTTTCAAACACAAACAAGACCCTTC
ACTTCATCATCAACAATGTTTCAATCTTCTAACGGCACAATTCAAACTGCACAAAAACAGGGATGGAG
TAACAGTTACTTTCAGGCACAAGAACCCCAAAAGCAAGATGATTTTTCCTCAGGGAAGAGTATGGTGA
AACCTGAATATGGTTCTGTGCAGAGCTTCTCATCTGGAATGGCCACCAATATCCAAAATAATAGTCAGG
CCAATGGTGGTTTCCAATCTGAGTATAGCAACTACAACCACCAAACATCTCAGACCTTGAGCAGGAAG
TCAGATGATGGGTTCAATTGGAGAAAATATGGTCAAAAACAAGTGAAGGGAAGTGAAAATCCAAGAA
GCTATTACAAGTGTACTTACCCCAATTGCCCAACTAAGAAGAAAGTAGAGAGGTCTTTGGATGGGCAA
ATCACTGAGATAGTTTACAAGGGCAACCATAACCATCCCAAGCCACAGAATACTAGAAGATCATCATCA
AATTCTTCGCATGCAATTCAGGCTTCTAATCCCAACACCAATGAAATCCCAGATCAGTCTTTTGCCAAT
CATGGTAATTCACAAATGGATTCCATTGGAACTCCAGAAAATTCATCCATTTCAATGGGAGATGATGATT
TTGAACAGAGTTCTCAGAAAAGCAAGTCAGGAGGAGGGGATGAATTTGATGAAGATGAACCTAATGC
CAAAAGATGGAAAAAGGATGTTGATAATGAAGGTATTTCAGCTCCTGGGAGCAGAACAGTGAGAGAG
CCTAGAGTTGTAGTTCAAACAACCAGTGATATTGATATTCTAGATGATGGGTACAGATGGAGGAAGTAC
GGGCAGAAGGTGGTAAAGGGCAATCCAAATCCAAGGAGCTACTACAAGTGCACAAATCCAGGATGTC
CAGTGAGAAAGCATGTTGAGAGAGCTTCTCATGATCTTAGAGCTGTGATCACAACCTATGAAGGAAAG
CACAACCATGATGTTCCGGCAGCTCGTGGCAGCGGCAGCCATGCTTCTGTCAATAGGGCTCTACCAAA
CAACAACAACATTAACAACAGCAATAACAATGTAGCCACAGCAATGAGGCCTGTGGCCCATCAAACTA
ACAACCTAAGGCAACAAACATCAGAAGGGCAACAAGCACCCTTTACCCTAGAGATGTTGCAGAGCCC
CGGGAGTTTCGGATTTGCAGGGTTTGATAACTCTATGGGGTCATACATGAACCAAGCGCAGCTCAATG
AGAACATGTTTTCTAAAACCAAGGAAGAGCCAAGAGATGATGCCTTTTTTGAATCATTGCTATGCTGA

>PmWRKY02

ATGGCTGGCATCGATGATAATGTTGCTATAATTGGAGATTGGGTGCCTCCAAGTACAAGCCCAAGAGCC
TTCTTCTCTTCAATGTTAGTTGACGACATAGGCTCAAGATCAATGCTGGAACCTCCCAGCAGTAATAAA
ACTGCGGAGTTCTTTTTGGGGTCTCAAGAAGAAGATAACAATGGAAAAAATTTGTCACAAGGCAATGC
TTCTGGTGAGGAACTAAATGAAGTGGGTTCATTTTCGGAGTACAAGTCAAACTCACGCGGAGGGCTTG
TGGAAAGGATTGCAGCCAGAGCTGGGTTTAATGCTCCGCGGCTGAATACAGAAAGCATTAGATCTTCT
GACCTTTCACTTAATTCGGACATTCGGTCCCCTTATCTGACAATACCTCCTGGTCTCAGTCCAACCATAC
TTCTAGACTCTCCTGTTTTCCTTTCAAGTTCATTGGCACAGCCATCTCCAACAACTGGAAAATTTCCATT
TGTCTCAAATGGTCATAGCAGGAGTTCCACATTAATGACAGAGGGCCCCGATAAAACTAATTTCTTTG
AGGACATAAATACTTCATTCGCTTTCAAGCCTATTGCGGAATCAGGCTCCTTCTTTCTTGGTCCAACTAG
CAAAATGGGTTCAACTAGTTTTCCACAGCAATCTTTTGCCAGCATCGAGGTGTCAGTCCAGTCAGAAA
ATTCTTCTCAAAGTATAGAACCAACCAAAGTTCAAAACCAGAATACAAACAATCTTCAGCTCCAGGCA
GACTTCTCTCGCACATCTACTGAAAAAGATAATGGAGCTAACTCAGCAGATCCAAGGGCTTTTGATACT
GTTGGTGGCAGTACTGAGCATTCTCCACCCCTTGATGAGCAACCAGATGAAGAAGGAGATCAAAGAG

GTAGTGGAGATTCCATGGCTGCTGCTGCTGGTGGTACACCATCCGAAGATGTATATAATTGGAGAAAAT
ATGGACAGAAACAAGTAAAAGGTAGTGAGTATCCACGAAGTTATTACAAGTGCACGCATCCAAATTGT
CAGGTTAAGAAAAAGGTTGAACGATCTCATGAGGGTCATATAACAGAGATCATCTACAAAGGGGCCCA
TAACCACCCTAAACCTCCTCCCAATCGTCGATCAGCCGCCATTGGATCATCTAACCCACTTAATGACAT
GCGACCAGACATCCCTGAACAAGGTGGACCACAGAGTGGTGCTGATGGTGATTTAGTTTGGGCAAGT
ACACAAAAGGCAAATGTTGGAGCTCCTGATTGGAAGCATGAAAACCTTGAGGTGACTTCATCAGCAT
CTGTGGGCCCTGACTACTGCAACCAATCCTCTTCTATGCAGGCTCAGAATGGTACGCACCTTGAATCAG
GTGATGTGGTGGACGCGTCATCTACCTTTTCTAATGATGAAGATGAAGATGATCGAGGGACACATGGCA
GTGTTTCATTGGCTTATGATGGTGAAGGAGATGAATCAGAGTCGAAAAGAAGGAAAATTGAAGCCTAT
GCAACAGAAATGAGTGGAGCTACCAGAGCCATTCGTGAGCCTAGAGTCGTTGTCCAGACAACCAGTG
AAGTAGATATCCTTGATGATGGATATCGTTGGCGCAAGTATGGGCAGAAGGTTGTGAAAGGCAATCCA
AATCCAAGGAGTTACTACAAGTGCACCAATGCTGGCTGCACAGTGAGGAAGCATGTGGAAAGAGCAT
CCCATGACCTTAAGTCAGTGATCACCACATATGAGGGAAAGCACAATCATGATGTTCCCGCTGCTCGAA
ATAGCAGCCATGTCAACTCTGGCCCGTCCAACACCATGTCTGGCCAAGCTTCCTCTGCTGGCATTCAA
ACCCATCCGCATAGACCGGAGCCATCACAAGTTCACAACAGCATGGCAAGATTTGAAAGGCCTTCATC
ACTGGGTTCATTCAGCCTACCTGGAAGGCAGCAGCTGGGGCCATCCCATGGCTTCTCTTTCGGAATGA
ACCAACCCGGCCTGGCCAATCTGGCAATGGCTGGGTTGGGTCCAGGCCAACCCAAACTCCCTGTTATG
CCTGTTCATCATCCGTACTTTGCACAACAGCGCCAGGTCAATGAAATGGGCTTCATGTTACCAAAAGG
AGAACCAAAAGTGGAGCCTATGTCAGAATCTGGTCTAAACATGTCCAATGGTTCATCTGTATACCAACA
GCTAATGAGTAGGCTTCCTCTTGGACCACAGATGTAG
>PmWRKY03
ATGGCATCTACGACGTCCCAGGCCATGCTCAGCCAGCAGCAGGGCTTGTTTGAGAATGAAGAGGGTTC
TAATGCACAGATGGGTTTCTTTTCAATCCCACCAAACTTGAACTTTTCTTCATTGGGATCTCTCAAAGC
CTTCGGTTCTTCTAGCATACCTTCAAGCACCAATCTCTCTGAAACCCTACTCCCATCACTCACTCCTCTA
AAGCATAGAGAAGATCATAACATCACTACTAGTTCTGATTTTGGAGGATCCCAACTCCTTTCCTTGCAA
AGATCCACTGCAAATCTCTGGGCATGGGGAGAGGTGAGCGATGATCATGAGTGCCTAAGCAGCAAGA
GATCAAACGGTGGAGATGATCGCCATCATCACCATATGGGAGTTTCAGCAATGAAGATGAAGAAGATG
AAGGCAATTAGGAGGAAGGTGAGGGAGCCTAGGTTTTGCTTCAAAACCATGAGTGAGGTTGATGTGC
TTGATGATGGATACAAGTGGAGGAAGTACGGACAGAAGGTGGTAAAGAACACGCAGCATCCCAGGCT
TGGCGCTGGAGATTTGACGGGTAGATGGGTCTGA
>PmWRKY04
ATGGACTGTCTTCAGAATCCAAACCCTAGCTCTGCCGGCCCTTATCACTTCGGAGAGAGCATCGATCCT
TCCATCGATTTTGATGAGTTTTCAGACTGTTTCATGCTTGATTATGGTGTTGATGATCATCAAGATTCTTC
GTCTTTAAGTACCGTGTCGCCGGAGAAGTTCATGGCTGACTGCTTTACTGGATCCAGTGGTGGTGCAA
CATCAAGAAATAGCAACAATAATATGAAATGCAGAAATGAGGGGAGGAGAAACAAGACAGAAATGGG
TCATAGTAGGGTTGCTTTCAGAACTAAATCGGAGCTGGAGGTTATGGATGATGGATTCAAGTGGAGGA
AGTACGGAAAGAAGTCAGTCAAGAACAGCCCATATCCAAGAAATTACTACAAATGTTCAAGTGGAGG
GTGCAATGTAAAGAAGAGGGTGGAGAGGGACCGAGAAGACTCGAGCTATGTGATAACCACATATGAT
GGAGTGCACAATCATGAGAGCCCTTGTGTGGTATATTACAATCAGATGCCTCCTCCTGTGGATCCCAAC
AACATCTGGACTTTGCGAGCTTCTTCACAGTCTTCTGCTTCTTCATAG
>PmWRKY05
ATGGCTGTAGATTTTATGGGTTATAGGAACAGCAGCTTCTCTGCGAAATTGGAAGAGAACGCGGTGCA
AGAAGCAGCTTCTGGGCTCGAGAGCGTTGAGAAGCTCATTAGACTCTTGTCTCAGGCTCAGCAGAAC
CAGCACCAAGATAAGTACCCTTCCATGGTTATGGACATGGATTGCAGAGCCGTCGCGGACGTCGCCGT

TTCCAAGTTCAAGAAGGTCATTTCTCTTCTGGGTCGTACCCGGACCGGCCATGCCCGCTTCCGGCGAG
CACCTTTGACTTTGACTTCTGGGTCTTCTTCTTCTTCTCAAAACCAAGCCCAAACCCAAGAGACCTTTG
TCAAGCAAGCTCCTTTAGAGTCTACCAAGGTTTACCATGCGACGCCGATCCAGCAGATCCCGCCTCCT
CTGCATCATCACAGTACTGTGCTCGAGAGCACCAAGGACTCCTCCACCACTATAAATTTCTCCTATTCA
GCTACGACGTCGTTTATGTCGTCGTTGACTGGAGACTCCGACAGCAAGCAGCCATTGTCGTCTTCGGC
TTTTCAAATTACCAACATGTCCCAGGTGTCTTCAGCTGGAAAGCCACCGCTTTCGTCAGCTTCGTTGAA
GAGGAAGTGCAGTTCTGATAACTTGGGCTCTGGCAAGTGCGGTGCTGGGTCCTCCGGCCGCTGCCATT
GCTCTAAGAAGAGAAAGCTGAGATTGAAAAGGGTTGTGAGAGTTCCAGCTATAAGCTTAAAGATGGC
TGATATTCCACCTGATGATTACTCTTGGAGAAAGTATGGACAGAAACCCATTAAGGGATCTCCACATCC
AAGGGGATACTATAAGTGCAGCAGTGTCAGAGGGTGCCCAGCTCGAAAACATGTAGAGAGAGCTCTC
GATGATCCAGCAATGCTTGTAGTGACCTATGAAGGCGAGCACAATCACTCTCTCTCAGTTGCAGAGAC
CTCCAATCTCATTCTAGAATCGTCTTAG
>PmWRKY06
ATGGAGGACGCAATGGGTGCCACGTGGTCCGACTGGTCGGAGGAAGAGCTTGTGAGAGAACTTCTGG
ACAATGAGACACCATTCTTTGTGCTACCAGAGGAGGCTCTACAATCCCAGATGAGTGTTTCAAATGAA
GATAGTGTTGTTAATCGGTTCATTCCGACCGTCTATTCTGGTCCAACAATCAAAGATATCGAGACCGCTT
TGTCTGTCACCACTGGAGCAATTCAACCCCAAGAACTTTCACCGGCTAGGCTCTCAATGCTGGAAAGG
GGTTTGAGCAAGGTTGAGCATAAGTACACTCTGAAGATTAAAAGCTGTGACAATGGAGCAATGGCTGA
TGATGGCTATAAATGGAGGAAGTATGGGCAGAAATCCATTAAGAATAGCCCAAATCCTAGGAGCTACTA
CAGGTGCACAAACCCAAGGTGCAGTGCTAAGAAGCAAGTTGAGAGGTCCAGTGACGACCCAGACAC
ACTCATCATCACCTATGAAGGGCTCCACTTACACTTTGCCTACCCATTTTTCCCACTCAACAATCAGGC
CCAGAACACCAGCCCACCTATGAAGAAGCCCAGAAACAAAACCTCACAGCCACAAGCAGAAGACCG
TGAACATGAAGCCCAAGAAAGCCCGCGAAGTATTACCCCTGAACCTGACCCGGATCTGCAGCCCGGC
CCATTCCCCGACCCACATGAAGAATTTGTTGAAAAAGAGCCAAGCGCACAAGGGTTGCTTCAAGATGT
GGTGCCATTTATGATTAGGAACCCTAATCCATCGAGTGCTTCTTCAAATTCTTCTTGTTCCTCCTACCGG
TCGTCTCCAACATCTCCTTCTTCTCTGTCATGGGCTACTTCGTATTTCGATGTTGGTTTTAATCATAGCTT
TGGATGA
>PmWRKY07
ATGGATATTAAGGAGGCAGAGCGGGTAGTTATAGCTAAACCAGTTGCTTCAAGGCCTACTTGTTCAAGC
TTTAGGTCCTTCACGGAGCTTCTTGCAGGAGCTATCGATGCCTCTCCTTCTAATATATCTTCTGAAACTG
CTGTTCCTGCCATCAGACCAAAGACTGTGAGGTTCAAGCCAACGGTGAATCATGCTGTCTCTGGATTG
GTTTCTTCCCAGGCAGAGATGTCTGGTACTGCACATAGTAATTCATCAGAGAAGATCTCAAAATCAGAC
AGCAGAGCAAGTGTGGTATACAAACCATTGGCAAAAGTTGTATCAAGGGCAACTGTTTCTGTCTTGGC
AAATATGGTCAGTAGCAAGGGAAACTTCAATACCAGCCACCAATCTACACAATCATCAGTCGAGGCTG
GTATTCTACATCAAAATCAAGATAAATGCTTTAGATCCCAACTTAGCCCAAATCTGCGCCATAATAATCC
ATCATGTGCTGAGACAAATCAGACAATAGAGCCTTTGAAGATAGCATCACAAAATGTGGAAGAGGATC
CAAAACATATACCGTCCACAGCCAATACTGATAGGCCTTCTTATGATGGGTATAATTGGAGAAAATATGG
ACAGAAGCAAGTCAAAGGAAGTGAGTACCCGCGAAGTTACTATAAGTGCACACATCCAAATTGTCCT
GTGAAAAAGAAGGTTGAGAGATCTTTGGATGGACAGATTGCTGAGATTGTCTACAAGGGTGAA
CACAACCACTCAAAGCCTCAACCTCCAAAGAAAAGCTCATCGGGGACGCAAGGGTTAGGCTTAGCAT
CTGATGGAACTGGTCAAGATACCAACAACCGGTTATGGAATAGTCAACTTAATGAAAGGAATGAAGGT
TCTGAAGGTAGAGTAGAGGATCAGAATGAAGTTGGACTACCTGCGCATTCTTATCAAAGTAAAGCTCC
ACTACTTTATGATCCTCTTGCAAGTGGAGGAATAAATGCTGGTGGTGGAACTCCTGATAATTCTTGTGG
TCTTAGTGGAGAATGTGAGGAAGGAAGCAAGGGACCTGAAGCAGAGGATTATGAACCTAGAAGTAAA

AGAAGAAAAAGTGAGAATCAATCGAATGAAGGGGGCATATCAGGAGAAGGTGTGCCAGATCCCCGTG
TTGTGGTGCAAAGTTCTGTAGATTCTGATATGACGGGTGATGGCTTCCGTTGGAGGAAATATGGGCAGA
AGGTTGTTAAGGGAAATCCATATCCCAGAAGTTACTATAGATGCACCAGTCCAAAATGCAGTGTGCGTA
AGCATGTCGAAAGAGTCTCAGACGATCCCAAAGCTTTTATTACAACATACGAGGGAAAGCACAACCAT
GACATGCCACTGAGGAACACAAACCCAGGAGCATCCGAAAAGGATGCACAGGCCCCAACAACCAAA
GAAAAGCCATGA
>PmWRKY08
ATGGAGTTGTCTTGGCCGGAGAGTGTGCTATCAAATAGGGAAAGGGTGATGGAGGAGTTGATCCAAG
GGCGTGAGCTTGCAAGTCAACTTTGTAAGGTTCTTGATGATCACAAGTCAACACTTGTTAGTGGTTGT
GGTGGTGGTGGTGGAGGAGATGTTGGGTCGGCAGAGGGTCTTGTTAATAAGATTTTGGGGTCATTTAC
AAATACCCTTTTGATCTTAAATGGGAAGGAGGCTGACGGGGAGATTGTTTCTGGTGATCAGATTCAAG
GGAATAGTAGTGGTATTGGGTCTGCAGATTCATCATCTTGGGATGCCAATCATGCTATTATTAAGTCTGA
AGATTTTCATGAGGAGATCAGTTGTAAGAGTGCTTCAACCTTCAAGGATCGTAGGGGTTCTTACAAGA
GAAGGAAAACTTCACACTCTTGGACTAGAGACACTCCGGCTTTGACTGACGACGGTCATGCATGGAG
GAAGTACGGACAAAAAGTCATCCATAATGCCAAGCACCCAAGGAACTACTTTAGGTGCACCCACAAAT
TCGATCAATCGTGCCAAGCAACCAAACACGTGCAACAAATTCTAGATGATCCACCAGTTTTTCGGACC
ACATATTATGGCAACCACACTTGCAGAGACTACTTGAAAGCTTCTGAGTTGATCTTGGATTGCACAAGC
CCTAGAGAGTCTTCAAGTTTCATCAGATTTGGCGACATTAAGCAAGACCACCCCTTTTTCTCATCTTTC
ACATCAGTGAAGAAAGAGGTCGTCATCAAGGAAGAGAAACCACAACCAAGTGATGATATGATGGCAA
GCCACCACCACAACCATTCATCATCCGGTGATTATAATGTGTCACCTCATCCGACGGAGTTCAAGTCGT
CTTGTCCCCTGAGCGGGCTTTCGTCAACCATTGATCCGTATGATCATGAGGGCGATGTGATGTCTGGTTT
AATTGTGGGGCCTTATTACTTTGATGATGAAGTTTTGCAATATG
AATTTTGA
>PmWRKY09
ATGGAGTGGTCTTGGCCGGAGAGTGTGTTATCAAATGGGGAAAGGGTGAAGGAAGAGTTGATGCAAG
GGCGTGAGCTTGCAGGTAAACTTTGTAGGGTTTTTGATAATCACAAGTCAACACTTGTTAGTGGTTTTG
GTGGTGGTGGTGGTGGTTCGGCTGAGGATCTTGTTGATAAGATTTTGGGATCATTTGCAAATACCCTTT
TGATCTTAAATGGAAAAGAGTCTGACGGGGAGATTGTTTCTGGTGATCAGATTCAAGGGATCAGTAGT
GGTGGTGGCTCTGCAGATTCATCATCTTGGGATGCTAATCATACGGCGGCTGTTATTAAGTCTGAAGATT
TTTATGAGGAGAGTTGTAAGACTACTTCAACCTTCAAGGATCGTAGAGGTTCCTACAAGAGAAGAAAG
ACTTCACACTGTTGGACTAGAGACACTCCTGCTTTGGCTGACGACGGTCATGGATGGAGAAAATACGG
ACAAAAACGCATCCTTAATTCCAAGCACTCAAGGAATTACTATAGGTGCACTCACAAGTTCGAACAAT
CTTGCAAAGCAACCAAATACGTGCAACAAATTCAAGATCATCCACCAATGTTTCGGACCACATATCAC
GGCAACCACACATGCAGAGACTACCTAAAAGCTTCTGAGTTGCTCTTGGATTGTACAAGTCCTAGAGA
GTCTTCAAATTTCATCAGATTTGGCGACACCAAACAAGATCACCCCTTTTTCTCATTCTTCACATCAGTT
AGAAAAGAAGAATTGGTCTTCAAGGAAGAATCGATCATCAAGGAAGAGATACCACCAACAAGTGATG
ATCGTGTGATGACAAGCCACGACAACTATTCATGGTTGTGTGATTATTTTGTGTCACCCGATCTGACGA
CGTTCGAGTCCTCTGGGCCCCCGAGTGGGTTCTCATCAACCCTCGATGAGTTTGATCATGAGGATGTGA
TTTCTAGGTTAATGATGGGGTCTTTTGACTTGGATGAAGTTTTGCAATATATTTGA
>PmWRKY10
ATGGAGGGAGTGTCGTCAAATCGGCAAAGTGAGATGGAGGAGCTGATCCAAGGTGGTGAAGTTGCCA
GTGGTCAACTTAGTAGGGTAATTAATCTTCATAATAATAGGTCACTTACTGTTAATGGTGATCATAATCAT
GATTTGGGATCGGGCGATGATGAAGGTTTTGTTAATAAGATGTTGGGGTCATTTTCGAATACTCTTTTCA
TCGCAAATGGGAAGGAGATGAAGGAGTTTCATCATCATGATGATCAGGAGCTGATTTCTGATCAGATTA

TTCAAGTGAATACTGGTGGTGGTGGTGGTGGTGGTGGTGGTGGTGGTGGCGGTGGTGCAGGTGCATCA
TCTTGGGATGTTGATCATCTACTTGATCATGCTATTAAGTGCGAAGCTTCTCATGAGGAGAGTTGTAAG
AGTGCTTCGACCTTCTCCACGGATCGCAGAGGCTCTTACAAGAAAAGGAAGACTTGTCACTCTTGGAT
TAAAGACTCTCCTGCTTTGACAGAAGATGGTCATGCATGGAGGAAGTACGGACAAAAACTCATCCTTA
ATGCCAAGCACCCAAGGAACTACTTTAGGTGCACCCACAAATTCGATCAAGCATGCCAAGCAACCAA
GCACGTACAACAAGTTGAAGATGATCCACCATTGTTTCGTACCACATATTATGGCAACCACACATGCAG
AGTCTACTTGAAAGCTTCTGAGCTGATCTTGGATTGTACAAGCCCTAGAGAGTCTTCAAAGTTCATCAG
ATTTGACGACACCAACCCCTCAAGCAAACAAGAGCACCCCTTTTTCTCGTCGTTTAAATCGCTAAAAG
GAGAAGAATGTTTCAAGGAAGAGACGATGCCAAGTGATTATATGACAACCCAGCACCACAACCAATTA
GCATTGAGTGATTATCTTGTATCACCTCCTGATCTTCCGGCATTCACGTCCCCTGGGCTCATGAGCGGGT
TTGCATCAGCCAATTATGATGACTTTGCATTTGATGAGTTTCTGCAGTATGAGTTGAGGTAG
>PmWRKY11
ATGGATGAGGTTGAAGAAGCTAACAAAGCAGCTGTTGAGAGCTGCCACGGAGTTCTCAGCCATTTGT
GTCAACCTAAGGGCCAGGTTCATTGTAAAAATTTACTGGCGGAAACTGAGGAGGCTGTGTTCAAGTTC
AAGAGAGTTGTGTCTCTTCTGGGCGGTGGCTTAGGCCATGGAAGAGTGAGAAAGTTGAAGAAGTTCA
AGCCGCCACCTTTGCCTCAGAACATCTTCCTAGATGGCCCTAATTACAGATTAGATATTTCAACAAAAC
CCCTCCAATTACTCCCACCTAATTCTCTTGAAAACCGACGCGCAGAAAAGGATTCTAAGTATGGAAGTT
CTCTGCAGCACACACACACAGCCAGAAAGTGTTTCTTGGAAACCCAGTAGTGGATTTGGAAATGAAAAT
CAAGCTCCCTCTCCAAATTCCCAAAACAAAACCATTGCAACAGTATCATTTCCTCGAACAGAAGCATG
ACCATCAGCAAGAGATACAAAGATTGCAGCTTCAGCCCCAACAGTTGAAATATCAGGCTGATACAATG
TACTCTGGGAGAAACCGTGGCATCAACCTTGCATTTGATAGGTCCACTACCCGCACTCCCTCCATGTCG
TCAGCCAGATCATTTGTATCATGTTTGAGCATGGATGGTGGTGTGGCTAACACTCACTGCGATTCGTTC
CAACTAATCCGCGGTGTGCCTCAGCCGCCTGATCGGATCTCACAACAACAAAGGAGGTGCAATGGTGC
CGGGGAAAACGGAACTGTAAAATGCGGCAGTAGTGGTAAATGCCAGTATCCAAAGAAGAGGAAAGTG
AGGGTGAAAAGATCGTTTAAGGTTCCTGCCATTAGTAACAAGATTGCAGACATTCCAGCTGATGAATAT
TCATGGAGGAAATATGGGCAGAAGCCAATTAAGGGTTCCCCCTATCCTAGGGGATACTATAAGTGCAGC
AGCATGAGAGGGTGTCCCGCGAGGAAACATGTTGAGAGAAGCCTTGAAGACCCCGCTATGCTTATCGT
CACCTATGAAGGCGAGCACAAGCATTCACCATCTGACCAGTGA
>PmWRKY12
ATGGACCAAGAAGACGACGCCGTTTCGGAGAACAAAACCAAAGGTTGTGGACCTGGTGCTAACACCA
TTAGTATAGCAGAGAGGAGGGCAGCCAAGTGTGGATTCAATGCGGAGAGGATCAATACGGCGCGTTTT
AGGACAACAAGCCCTTTGCCTTCGCCGGCCGCGGCTGCCAGGTCCCCCTGCCTCACTATTCCACCTGG
TATCAGCCCCACGGCTTTGCTCGACTCCCCCATGATGCTCCCCAATTCTCAGGCGCTCCCATCTCCAAC
CACCGGAACTTTTCCGTTGCTTCCTCCCAATGATGACAGTTCAGTGCTGAAATCTGGAACTCATGAAGA
TGGTCACAGGGGGGAGTGATTTGGGTTCATCCTTCACATTCAAGAGACAGGGCGATCCCAAATATCTGC
CAGGCTATTCTAGTTTCGAAAATCAGGGATCTACTGTTGACTACCAATCTCTGGTTTTGGAGCAACAAC
CAATAGATTTTGAATTCCCAATGGAGTTTCCGGAAGAAGCTAATGCAAAGAACTATGCTGTGGATCCAT
CCACCCATGTTAAAAACTTAAATAGTGGGATTCTGAATGCCAACTGCGTAGACTTGCAAAGGTCTAATT
CAAGCGCAGCTAGTGAACAAAACTCTCTTCCCAAAGAACCGATTCATGGAGAGGACGTTGGGAGCCA
CCCATTTTTGGAAGGAGAGCATAGAGGGTCATACCCATCTGCGGGAATGGTCAGGACTTCAGAAGATG
GATATAATTGGAGGAAGTATGGGCAGAAACAGGTCAAAGGCAGTGAATATCCAAGGAGCTATTATAAA
TGCACCCATCCAAATTGTCAGGTGAAGAAAAAGGTGGAACGATCCTTTGATGGTCAAATAACAGAAAT
CATCTACAAGGGAGCTCCTCATAATCATGCAATGCCTCAGCCTAATCGTCGAGCTGGAGCATCACTTGG
ATCATCATTTTCATTTGATGAGACGTCAGAGATGAGTGAAGGAAGTAGGGCCTCCGTCAAAGTTGAAG

GTGGGTTAGTTTGGACAAATATTCAGTCTGGTAAGGATATTAAAACTGGTTATGATGGGAGGGCTGAAG
GTCTGGAAAGGACATCCTCAACATCTGTTGTCACCGACCTTTCTGATCCATTATCAACTACCCAAGGGA
AATCCATGAGTATCTTTGAATCAGCAGAAACTCCAGAGTTTTCATCCACACTTGCTAGTCATGATGATG
ATGATCGGGGCACCCAAGGAAGCATATCACTTGGAGATGATGCAGATGATGAAGAATCTGAGTCAAAA
AGAAGGAAGAAAGAGAGCTGCTTGATGGAACCAAGTTTAGCATCCAGGGCTGTCCGTGAACCAAGA
GTAGTTGTCCAAATTGAGAGTGAAGTCGACATACTTGATGATGGCTATCGTTGGCGGAAGTATGGGCA
AAAAGTTGTCAAAGGAAATCCAAACCCTAGGAGCTACTACAAATGTACAAGTGCGGGCTGTTCAGTA
AGGAAGCATGTGGAAAGGGCCTCCCACAATCTGAAATTCGTAATTACAACATACGAGGGAAAACACA
ACCATGAAGTGCCCGCAGCGAGAAACAGTAATCACATAAATTCAAACGGTGGCAATGCACATCCATCT
ACTGCCAATGCTCAGCCAGCTCTTGCATTACCCAGGAGTAGCAATAATCCAAAGCCTGAGACACAAGT
ACAAGATCTTGCACCTCATTTTGATAGAAAACCGGAGTTCCATGAAGAATACCTGAGATCTAGTTTTCT
TGGGAATTTCAATAATGAATTGAAATTTGGAGTTCCCTCCATTTACCAAATGAAATATCCTCCTTTGCAA
AATACCATGCCATATAGCTCGTTTGGACTACATGCTAATCACGGTGTGACCCATCAGGCTGGTTCTGTTG
TCCCAGACTTCCCAATTTCACTGCCTTTAAACCTTCCTCCATCTGGGAATCTCCCTCTCTCTGGTTTTGA
TTTCAACAATGGAAAATCAGTTTGTCCAGTACAGCCTTATTTTTCAGGACAGCAGCTGCAGGAGGATG
ATATGAGGTTCCTAAAGCCTAAACAAGAGCAAAAGGATGATAACCTTTATGATGCCTGTTTGCCTATCA
TTGACCAGGCAAATGCATCATTGTCGTCATCATCATCATCATCGCTGTTATATCAAAGGATCATGGGAGG
TTATCCATCATAA
>PmWRKY13
ATGGGGAGAGAGAAGGAGATAGGCTTGGATATTGATCTCTCACTCAAGATTGATTCTAAACAAGAAAA
GCAAGGAGAAGACGGAGACCAAGGCGAAGGCGAAGAGGAAGAGAGAGAGGAAGAGGAAGATCGC
AACGACGATGATGATATTGAACAAGGAGCAAAACAAGAGGAGGAGGATGAGCAAGCACCAAGAGCA
CAGAATAACAACCAAAAAGCAGCAGAAGCCGCTGCTGGGGAAGTTGAAGATGATGCCTCAGTAGTAG
AAACATCTTTGGAGGAGAATAACATGAAGACGGATCAAGAGCTATGCGTTTTACAAATGGAGATGAGC
CGCATAAAAGAAGAGAACAAAGTTTTGAGGAAGGGCGTGCAGCAAACCATGAAAGATTATTATGATCT
CCAAATGAAATTTACGGCTCTTCACCAAAGCAACCAAAACAAGGATCCTCAAACCTTTCTATCACTTG
ATGGTAATGATCCCGATGCTGTTCAAGGACCCAAGACGATTCCAAAGAGATCTCCATTATCACCAACAC
GTGAAGATATGATGAAAGAGAGTCAACTAGGGTTGTCACTGAGGCTACAAACCAGCCCTACTACCAAT
ATTCAATCACACGGAAGAGAAGAAGATGATGATGATAAAGAAGAGAACAAGAAGAAGGAAGACTTG
ACAAGTTCAAGTCTTGCATCATCACTACTGCAGAACAAGCTTCAGCGAACCGAATTGGCCGGAATTTC
GAGTCACGTTACTTCCCAACCCAACAGAAAAGCTAGGGTTTCGGTTAGAGCTAGATGTGAATCCGCCA
CAATGAATGATGGGTGTCAATGGAGGAAATACGGACAGAAAATTGCAAAAGGAAACCCTTGCCCACG
AGCTTATTATCGTTGCACAGTAGCTCCTGGATGCCCAGTTAGGAAACAGGTCCAAAGATGCCTAGAGG
ACATGTCCATACTCATAACAACATATGAAGGAACACATAACCATCCACTTCCTGTTGGTGCAACAGCCA
TGGCTTCAACAGCTTCTGCAGCAGCTTCATTCATGTTATTAGATTCAAGTAACCATCATCTTTTAGATGG
AACACCCTCTAATTACACCCAAGCATCCCTCCCTAATTACTATAATGTCCCTGATCACAGGATGAACCCT
TCATCTCATTCCTCATCAAACTTTAGAACCATCAATCCTCATGACCCTTCCAAAGGAATTGTTCTTGACC
TCACAAACAACTTTTATGATCACAACAATACACCACAAAACATTCCCATGGGCAGCTCCTCATCACACT
CATCAGTACCACCAGGTTTTAGTAACTGGATGATGAGCACAAGCAGATCTGCAAGTAGTTATCAAAAC
CCTAATTCTATAACCAGCCATCTTTTGGCTAACAGCACTATTTCCAGATCATCAGGAGTTGGAGTGGAA
GATCAGAGAAGTTGGAGAGGGGAAGAAAATAATATGTCATTGGCTGAGAATGTGACTGCCATTGCTTC
AGATCCTAAATTTAGGGTTGCTGTTGCAGCTGCTATCACATCCCTCATTAATAAAGAGAATCATACAGCT
AATCATCCTTTTGGTCCAAGGGATGGTGGAGAGAATGGTAGTTCTACTAGCAACAATTGGGCCCTTGAT
CACCAGTCCTTCTCAGCCAATGGTAAGCCCATTCGCAAAAGCAGCTTAGAATAA

>PmWRKY14
ATGGCGGTGGACCTGATGAACTTTCCGAAGATGGAAGATCAGAAGGCTATACAGGAAGCTGCATCGCA
AGGCTTGAAGAGCATGGAGCACCTGATCCGCTTTCTATCGCACCAGCAGCAAACTAATCAGTCGTCTC
GCTTGGACTGTACGGACATCACCGACCACACCGTCTCCAAGTTCAAAAAGGTCATTTCGCTCCTGAAC
CGGACCGGCCACGCCCGGTTCAGACGCGGACCTGTTCAACCGGATCAACCGGTTCAGTTCCCCTCTTC
TTCTTCTCATCCCTCGTATTCACAAACATTGAGCTTGGCTCCGGCTCTGAATCCGAGGCCCTCTCCTGC
GCCGGCTCCGGTCACTACACTACCGATTGTCCCGCCGGCTCCGATCGAGTCGAGCTATGTCCAATCTCA
GCCGCACAGCATGACGCTAGACTTCACGAGGCCTAATGTCTTCGCATCAAATCCTAAGAGCACGGAGA
TCGAGTTCGCGAAGGAGAGCTTCAGCGTCTCGTCGAGCTCGTCGTTCATGTCGTCGGCGATCACCGGA
GACGGCAGCGTTTCGAACGGCAAACAAGGATCGTCGATCTTCCTGGCACCCGCGCCGGCCGTCTCCG
GCCCAAAGCCTCCGCTCTCTACGGCGCCGTTTAAGAAGAGGTGCCACGAGCACGACCACCACTCCGA
CGACGCGTCGTGCAAGTATTCCGGCTCGGGTTCTGCTTCTGGCTCCGGCAAGTGCCATTGCTCCAAGA
GAAGGAAAAATCGGGTGAAGAAAACCATTCGGGTCCCAGCGATTAGTTCAAAAATCGCCGATATTCCA
CCGGACGAGTACTCGTGGAGGAAGTACGGTCAAAAGCCGATCAAGGGCTCACCCTACCCAAGGGGAT
ATTACAAGTGCAGTACGGTTAGGGGGTGCCCAGCAAGGAAACACGTGGAGCGTGCCCCGGATGATCC
GGCGATGCTGATCGTAACATACGAAGGGGAACACCGTCATGCGCCGGAGAACGTGGGGTTGGTGTTC
GAGTCAACGTGA
>PmWRKY15
ATGGCTGTGGAGCTCATGATGGGATTTATTGACAGTTTTGCAGCGCAAATGGAAGAGAATGCTGTGAA
GGAAGCGGCCTCAGCTGGAATTCAAAGCGTCGAGAAGTTCATAAGCTTGATTTCGCAACACCACCAC
CAATTCCAAGACTCTTGTTTTTCTTCCTCATCGAACCCTGAAGCTGCTACAGAGTACAAAGCTGTTGCA
GACATGGCCGTGACCAAGTTCAGGAAGGTCATTTCATTACTGGACAATGGAAGAACCGGCCACGCTC
GGTTCAGAAGAGCTCCTGTCACTCCATCTCCGCCTCCACCGCCACCGCCGCTTCAGGAAACAGAGAC
CCAAATCCCAAGGCCTTCAATTCAAGAACCCCATAACCCAAAAACAGATCAACCCCCTGGTTTCAAAA
CAGAGCAATCCTCTGCTTTCAAGGTTTACTGCCCCACACCGAGTGTGCGTTTACCGCCTCTGCCTCACA
ATCCCCATCTGAAAACCACGCCTGTTGTGTTGACAAAGAGTGTTTTGTGCGCGGAGAGAAGGCTGGAT
GCTCCTACCACCATCAATTTCTCACCCTCACCTTCGATTTCCGCTGCCAATTCATTTATGTCTTCTTTAAC
CACTGGAGAGGCTGAGGGTTCTGTGCAGCATTCAATGTCTTCTGGGTTTCAGTTCACAAACATGTCAC
AGTCGTCCTCGGGCAAGCCGCCTCTTTCTTCTTCTTCGTTGAAGAGGAAGTGCAATTCCATGGACGAT
GTTGCAGCTCTCAGGTGCGGCTCATCTTCTGGGCGATGCCACTGTTCCAAGAAAAGGAAATCAAAAGT
GAAAAGAGTGGTCAGAGTCCCTGCAATTAGTATGAAAATGGCTGATATTCCACCGGATGATTATTCATG
GAGAAAGTACGGTCAAAAACCCATCAAGGGTTCCCCTCATCCAAGAGGATATTACAAGTGTAGTAGCC
AGAGAGGCTGTCTTGCACGCAAGCACGTGGAGCGGGCTCTAGACGATCCAACCATGCTGATTGTGAC
CTACGAAGGTGATCACAATCACTCCCACAGTGTCACGGATCCAACGCCTGCTCTCGTCCTCGAATCATC
TTAA
>PmWRKY16
ATGTCTAACAGCAACAACTTTAGAGCACAAGAGTCACCTGAGAATGACTTTTCCGACCAGTCCAATTT
CGAGTTTTCCGAGTACTTGATGATTGGTGAGTGGCTAGATGAAGATCATCGGACTTCCATGGCTTTGGA
GACCGTCCAGAATTCGGGGTATCAAGCAAATGAAGTTGATGAGTCTCGTGGAAGTAGCAGCCAACTTG
GAGGGTCTAATAGCAGAGAGAATGAAAGCGGCACCGTACGGGAGAGGCAGGAAGTTAGAGAAAGAG
TTGCATTCAAAACAAAATCAGAGGTTGAAATATTGGATGATGGTTTCAAGTGGAGGAAGTATGGTAAA
AAGATGGTGAAGAACAGCCCAAATCCAAGGAATTACTACAAGTGTTCAGTTGAAGGCTGCCCTGTGA
AAAAGAGAGTAGAAAGAGATAGAGATGATCCAAGGTTTGTAATTACAACGTATGAGGGCATTCATAAC
CATCAGAGCCTCTAA

>PmWRKY17
ATGATTTCATATTGTCCTGTGGCTCTTGAAATCTTGATGAACATGGATGCTCACACCCCTACCAAAAGA
AAGGTGGATGATCTCCAACAAGACTTGCTGCGTCTGCGAAAAGAGAATGAAGCTCTGAGATTCTTGCT
TCAAGCTATGACCACCAAATGCAACACACTTGAGCAGCTTATCCGAGAGAAAAACATTGAACGACATG
ATCAGTTTCCGGTTGCCCAGAAGACAACACAGTTTCTTGTGAGAACCGACTCCAAAGACAACACCCT
AATTGTGAAAGATGGATATCAATGGAGGAAATACGGACAGAAGGTTACGAAAGACAACCCATCATCCC
CTCGAGCTTATTTCAAGTGTTCCTTGGCTCCTCGATGTCCGGTCAAGAAGAAGGTGCAGAGATGCATG
GTGGATAAGTCTGTTCTTGTGGCAACATATGAAGGAGAGCACAACCATGACGCCATCAATGGCTCACC
ACTTGGACAATTTTGCTGTTCATCATCCACCGCTGATCATAATAATAATATTATTTTTAATCCTATTAATTT
TCCTTCGCATGGTATCTCAGCAACTATTAATAATAATGATGTCATAAATATTGCTAGCCCTTCGAATTCTT
CTCGGCCGGTGCCTATCACCCTTGATCTGACACTCTCGGGATCCATGAGCAATCAAGAAAATAATGGAT
CAGCTGGAAGCCCTCAAAACTCATCATCATGTGCCCCGAACATCAACTGTGAAAGTAGAATTGAAGAT
TACGTTGCTTATTTAACCAAAGATCATAATTTCACACAAGCTTTGGCTGCTGCGGTTGCAAGCTCCATTA
CAAGACCTCCTGCAGGCACAGAAAACCAGTGA
>PmWRKY18
ATGGACTCAGAATGGGTGAACACTTCTCTCGGCCTCAATGTTATTCCTTTCCACCCTGCTAGTTATGATC
AAGCTCCTGTGAAGAAGCAACTAGAGCTTGAAGGGGATTATACTAAAGTATTTGAAGGCCATGCATCG
GTGAAACAAGAGGCAGCAGCTAGCCATGTATTGACTGAAGAGTTGAATAGGATAAGTTTGGAGAACA
AGAAGCTGACTGAGATGCTTGCCTCTCTCTGTGAAAACTACACCAATTTGCAATCCCATGTGAAGGAG
TTGATGATCACCAAGCAGCAAAGTTCTGATCAAAACGATTTGGCTACAAACTTCAACAAGAAGCGAA
AACCGGACAGCGAAGACTACAGCAACATGATTGGATTAACTAGTACTGAGACCAGCTCCATCAGTGAT
GAAGAGTACGGATGTAAGAGGCCGAAGGAGAACATGAACTTGAAGATTTCTAGGGTTTATGTACGCAC
TGAAGCCTCTGATACACGCCTGATTGTGAAGGATGGATATCAATGGAGAAAATATGGTCAAAAGGTCA
CGAGAGATAACCCATCTCCTAGGGCTTACTACAAGTGCTCCTTTGCCCCAAGTTGCCAAGTTAAAAAG
AAGGTGCAAAAAAGTGCGGAAAATCCATGTGTGTTGGTGGCTACATATGAAGGAGAACATAACCACAT
GCACCCTGAAACCAGAGCTGAAGTAACATTGATAGGCTCAATATCTCCAAACCAGCAGCTTAGTCCGC
TTTCTCCATCCATGCCCAAGAGAACATCGCCAGTTCCAACTTTCTCATGTGACAAAAATAATAATCTGA
GCCCCCGAGAAATCGAAGGCGCTCCACCGGCTTTTCAACAGTTTCTGGTCCAACAAATGGCTTCTTCC
TTGACCAAAGATCCCAATTTCGCATCTGCACTTGCTGCTGCCATGTCAGGAAGATTTTCAGACCATTCT
CGGATGGGAAATTGGTGA
>PmWRKY19
ATGGACGACGACAAATACAACCGCGATCCAGCTAGTACGACCGAGTTCACAACTCAGTCCACCTGGCC
ACTCGACCCGGACAGCGCCTACTTCTTCGCCAGCCACGACGTCAGAGACAACACACTCCTCACCGAA
TTCGGCTGGAACTTCCACCCGGACGGCTCCAGACCCGACGGCTTTTCAGAACTTGACCCGATCGGAA
CCCGCGACATGTCCGATTTGGCGGCAACTAGTAGCCAGCTTGTTGCGGACTGCTTACGACCAGCCGAC
AGCAGCAGCAGCAGCACCGCGGCGTTTCGGAGCTCCGACCCGGCCCCCGCAGTCGGATCCGCGTCGA
CGAACCCGTCCGGTTCTTCGACCTCCAGCGAGGATCCGCCGGAGAAATCTACGGGGTCCGGCGGCAA
ACCACCGCCCGAGATACCGAGTAAGGTTAAAAAGAAGGGGCAGAAGAGAATCCGGCAGCCACGATTT
GCATTTATGACTAAGAGTGAAGTTGATCATCTTGAGGATGGCTACCGCTGGCGGAAATATGGACAGAA
AGCTGTCAAAAACAGCCCCTTTCCAAGGAGCTACTATCGCTGCACAAATAGCAAATGCACAGTGAAG
AAAAGGGTTGAACGTTCCTCTGAAGATCCCACAATTGTAATTACCACGTACGAAGGCCAACACTGTCA
TCACACTGTTGCATTCCCCCGGGGCGGAGTGATTGCTCAAGAATCTGGCTTTGCAGGCCACCATTTGG
CGCAGCTGCCTCCAGTCAGCTCACACTTCATGTATTATCCTGCAATTCAACCCAGAGAGCGAGTTAGTC
CTGTTAACTTGACACGAACGACTTCGCACCAATTAGCATCCCCACGTGGTGATGATGATGATGATGATG

ATCAAGCTGCAGGAGCAGGATCATCCCACTGCCTTAATCCACAGACAACTTCAGTTCCTGCAGATGAA
GGGCTTCTTGGGGACATTGTACCTCCTGGGATGCGTAATCGATGA
>PmWRKY20
ATGGCTGAGAACCAAGAGCAGCCACCGGGCCCACCGCCGTCCACTTCATCGTCACCCTTGAAATCAG
CTGCTCCTCCTCCTCCTCCTCCGCAGCGCCCTACGATCACCCTCCCTCCGCGCTCCTCTTTCGAGATGC
TCTTCAACTCCGGCGGCGCCAATAGTGGGCCGGGTTTCGGCCTCGGCTTCAGCCCAGGGCCCATGACG
CTCGTCTCCAGCTTCTTGTCCGATGGCGAAGACTGCAAGTCCTTCTCTCAGCTGCTCGCAGGAGCCAT
GTCTCCGGCGGCCAGGCCGCCTGGCTTCCCTCAGCTCGAAGACCGAGGCTCCGGCGACGGCGACGAC
AACTCCGATTTCCGGTTCAAGCAGAACAGACCGTCGCCGATGTTTTCGGTCCCGTCTCCGGGCTTGCT
CGACTCGCCGGGGCTCTTCTCGCCTGGTCAGGGACCCTTTGGAATGACACACCAGCAGGCCCTAGCA
CAGGTCACAGCTCAGGCTGCACAATCCTCTTCCTATTTCCATATCCCAACTGAATACTCATCTTCTTTAT
CGACAGTGCCCGCCACATCCTTGACACAGCTTCCAGCCTTTACTTCCGATTCAACAGCACCCCAGGAG
ATGCCATCTGGAGCAGCTGACTCTGGGGTGGCTATAAAAGAATCATCTGACATCTCCCATTCTGATCAG
AGATCCCAACCGTCTTCGTTTACTGTTGATAAGCCTAATGATGATGGATACAATTGGCGTAAATATGGGC
AGAAGCAGGTGAAGGGCAGTGAGTTTCCTCGAAGTTATTACAAATGCACGCATTCAAATTGTCCTGTT
AAGAAGAAGGTCGAGCGCTCAATTGATGGACAAATAACTGAGATTATTTACAAGGGTGAGCACAACCA
TGAACGTCCTCAGTCCAAGCGTGCAAAGGATTCTGGAAATCCGATTGGAAACATTCAGGCGAATCCTG
ACTTAGCTTCCCAAGTTCATGGTGGGCATTTGAACAAATCTAAGAAGGGTCAGGAATCTAGCCAAGCA
ACACACGATCATTTATCTGGGACTAGTGACAGTGAGGAAGTAGGAGATGCTGAAACCAGAGTAGATGA
AAAAGATGAAGATCAACCTGACCCAAAGAGACGAAATACAGAGGTTAGGGCAGAGCCAGCTTCTTCA
CATCGGACTCTGACAGAGCCTAGAATCGTAGTGCAGACAACCAGTGAAGTCGATCTTTTAGACGATGG
CTATAGGTGGCGCAAGTATGGGCAGAAAGTCGTCAAAGGCAACCCTTATCCCCGGAGCTATTACAAAT
GCACGTTCCCTGCCTGCAATGTCCGTAAGCATGTAGAGAGAGCCTCGACAGATCCTAAAGCTGTCATA
ACAACGTATGAGGGAAAACATAATCATGATGTACCAGCTTCTAAAACTGGCAGCCACTACGCAGCCAA
CAACAATGCCTCAAATCTAAGAGCAGTCAACGCAGGAACTGAGAAGATTAACAAGATGGATCTTAGA
AACAATGACCAACAGCCTATAGCACGTTTACGCTTAAAAGAAGAACAAATAACATGA
>PmWRKY21
ATGGAAATGCCGAAGAGGCGTTCAAAAGCAATGGAGAACAACAATACTGAGGAAAATTCTTTCCTTG
GAAATACACCAGTGGAAGACAAAACTAAGAAATCTGATGGTCATGATGAAGAGGGTACTTCCATGGA
GATGGAAATAAGCAAGTCTTCCTCATCCATGGATATCACAATTCAGGGAGGACGTGGGTGGCTTGCTG
AACCAGAGCAAACAAACACTTTGATGCCGTCTTCTTCTGCAGGAAAAGATAGAGCCCTTATCAAGCAC
CCACAGGAGGATGAGCTCCAATCAGCTAAAGCTGAAATGGGGGAGGTTAAAGAAGAAAATGAAAGGT
TAAAGCTGTTACTATCGCGAATCGTGAGGAATTACCAGTCTCTTCAGATGCATTTTCATGGCCTTCTTCA
AAAAGATGAAGAAGCTAAGAAATCTATGGATCCTAGTAGTTCTGCTCGTGATCAGTCGAACGAACAAG
ATGAAGAAGCTGATGAACTTGTGTCCCTTAGCCTTGGAAGAAGCTCAAGCATTGATCAGCCCAGAAAG
GATGAACAGATGAAAAAGACTAGCCATTTCAGTAAAAATGGGAAAGGTGACGATGAAGAAGGGCTCA
ACGGAGCTGGACTTGCACTGGAGTTGGGCTGCAGATTTGAGCCAGCTGCTGATCAATCAACTGAAGTT
GTTATGAAGAATTCAAGCTCTGATAATAGCTGCGGAGACCCCAAGGAAGACGACCCGACTGAGATATG
GCCACCTGGCAAGACTTCGAAGACAACAAGAAGTGGGGATGATGAGGTTTCACAACAAACCCATTTA
AAGAAAGCTAGGGTTTCTATCAGAGCTAGATGCGATGCTCCAACGATGAATGATGGATGCCAATGGAG
AAAATATGGACAGAAAATAGCAAAAGGAAATCCATGCCCTCGAGCGTACTATCGATGCACTGTCTCAC
CATCCTGCCCCGTGAGAAAACAGGTGCAAAGATGTGCTGATGACATGTCCATACTAATCACAACCTAC
GAAGGAAGCCACAACCATCCACTTCCTATGTCAGCCACAGCCATGGCCTCCACCACCTCCGCCGCGGC
TTCCATGCTTCTGTCTCACTCCTCCACCTCCCAACAAGGCCACACCGCCACGGCACCCATCTCTGCCTC

CACAAACCTCCAAGGACTAAACTTCAGTACAAGTACTCTCTCCCAAAATTCAAGACTACCACAACACT
TCTATTTCCCCAACAGTTCAATCTCAACCACAAATTCCCACCCAACCATCACTCTTGATCTTACTGCTCC
ATCTCCCTCTCACTTTGGAAGATTTCCTGCAGCTGTCTTCTCTTCCAACCCAAGATATCCCTCCACATGT
CTCAATTTTTCCTCATCTCCCTCCTCTTCTTTAGACCACAACAACCACACATTGCAGTTGCAAGCACCT
TGGAACAACAATAATCACACAGCAGCTGGCTATTTGAACTACGGGAATAGAGTACTTAACCAAGTTGG
GTCAGCTCTAAGCATGGGAAAGCAATCAATATTTCAAGAACCTAATAATTTGTACCAATCTTACATACA
AAATCAGAAGCCTCCTCCTCCTCCTCCTCCTCCTTCTCCTCCTCCTCTTCCTCCCCCTCCTCATCAGCAG
TTGATGCTAACAGAGACAATAGCCTCTGCAACAAAAGCAATCACATCAAACCCTAAATTCCAATCAGC
ATTAGCAGCTGCACTCACATCGTTTGTTGGTACTAATAATGGGGGTAGTGCTACTACTGGGGTCAGAGA
AAATCATCATCATCATCAGCATCAAAGTAGCACTTCTACAGAAAGTGCATCTGGGCTGAAATTGAAGTG
GGCTGAATCTTTGACAACAAATCCAATCTACCCACCAAGCCCAAATGGAATAGGGTGCGCATCAAGCT
ACTTGAACAAATCTTCGTGA
>PmWRKY22
ATGGGGAACCCTCAGGGCGCTTACAGTACTACTGATCATCTTCAATTCCAAAATAACAAGTCACCACC
AGCAGCAAATGGGTTCTTGGGGCTGATGTCAGATATGGAGGTTTCAAACAATATTAATTCTTCTCAGAG
CAAAAGCTTTGGGGGGCCTGAAGGTGCTGTGAGGTTAGGGACGAAGAAGGGGGAGAAGAAGATAAG
AAAACCCAGATATGCTTTTCAAACGAGGAGCCAAGTTGATATCCTTGATGATGGATATCGATGGAGGAA
GTATGGTCAAAAAGCGGTGAAGAACAACAAGTTTCCAAGAAGCTACTACCGGTGCACGCATCAGGGG
TGCAATGTGAAGAAGCAAGTTCAGAGGCTAACCAAAGACGAAGGCATTGTTGTGACAACTTATGAAG
GCATGCACTCTCATCCCATCGAGAAATCTACCGATAACTTTGAGCATATTTTGAGCCAGATGCAAATCTA
CACTTCAATTTAA
>PmWRKY23
ATGGATGGTAGAGAAACAGGAGAGCTAAAGACGACCATCGATCAAATTAGCATGGCGAATTCCACCGT
TTTTTCGGATGAGATTATTCCAGGCAGCAGCTTTGCTTCTTTTGCATCATCAGGCGGCAACATATTTGAC
ATGTCTGATAATAATCAGAGAGGCTCTGGGTTCATGGACTTGCTTGGATTTCAAGACTTCAACATGCCC
CCTTCTTTATTCGATTTCTCCTCTTCCCAAACGACGTCGTCCTCCTCGTCGATGATGATGGTGATGATGC
CACCTCACCACCAACAGCAACAACAACCACCACTTGCCTCTCCGGCCTCCACCCCTACTGCAGTTGCT
CTGCCGGAGAGGGAGAGTACGACGTCGGAGGTGCTGAACGGTAGTACGGCTCCAACGACCCCCAACT
CCTCTTCAATCTCTTCGTCGTCCAATGAAGCTGCTGCCAATAAGAATGAGGACCAGACGACGACCAAG
GCTCAGGATGAGGAAGCTGATGAGCAGAACCAAGATCCCGAAAAAACCCAGAAACAATTGAAGCCC
AAAAAGAAGAACCAAAAAAGGCAAAGAGAGCCGAGATTTGCGTTCATGACCAAGAGTGAGGTTGAT
AACCTAGATGATGGCTACCGATGGCGCAAGTACGGCCAAAAAGCTGTCAAAAACAGCCCTTATCCCAG
GAGCTATTATCGTTGCACCACTGCGGCCTGTGGTGTGAAGAAGAGGGTGGAGAGGTCCTCCGACGAC
CCTTCCACTGTGGTCACAACATACGAAGGCCAGCACACCCATCCAAGCCCCATTACGCCACGTGGCAC
CATGGGGATTGCACCGTTGCCTGACCAGCCTTGTGCCTTCCCCTCATCTCCTTTTGGTGTTCAACAGTT
ATTACCTCATCATCACTATCAACAACAACAACAACAGCAGCAGCAGCCGCAGTATTCGTATATTTATAG
CTCAGCCCCTTCTTTGAATATTAGCTCACCTGCTTATGGGGGTGGTGCTTTTAACCCCTCCTCATTTTCT
ACGGGTTTACTTCAAGAGAGATATAATTTTGGATCACCTTCTTCTTCTTCCTCTGCGGCTTCTAATTTGC
TTAGAGACCATGGACTTCTTCAGGACATCGTGCCACAGATTCGAAAGGAGGCCAAAGAAGAAGATCA
TCTTCATCAATAG
>PmWRKY24
ATGGACAAAGGATGGGGGCTCACCCTTGATTCTGACTCCTTCGGCTTCTTCCTCAACAAGCCTCCGGC
TGCCGTTAAGCTCGATCATCATCAAAACAACAGCAAGATAAGCAATTTTTTTGGTGGAGAAAGGATGT
TTCCGGGTATTGAATTCCCCGTCAAACTTGGCGGCAGGGAAGACCAGCTGGCTGCTCCACAGCCATCC

ATTCATGACAACAATAATCGTGTGGTTGTGGATGAGGTCGACTTCTTTTCTGATCGTAAGAATAAGCAC
AATACAACCACCACTGATGATCATCATCAGGATATGAAATCCAAAGGTACAATCAGCGTCAAGAAGGA
GAATTGGACTGGCTTGGATGTAAATACTGGCCTGCACCTTGTTACTGCTAACACCGGAAGTGATCAGTC
AATGGTTGATGATGGGATTTCATCCGATGTGGATAACAAAAGAGCAAAGAATCATGAGCTGGCACAGT
TGCAAGTGGAGCTCCAACGTATGAATTCTGAAAATCTGAGGCTGAAAGAAATGCTTGGTCAGGTGACC
AACAACTACAGTGCTCTGCAGATGCATGTTGCGGCTGTAATGCAACAACAGCAGCAGCAGCAAAATC
ACACAGCCGCGGCTGATCAAAGCTCTCAATTAAAACATGATCAGAATGTTGAAGCAAAGGCTGATCAA
GAGAAGAAACAAGGGTTGGTGCCAAGGCAGTTTCTAGACTTGGGCCCCAGAGCCACAGCCGAGACC
GATGACCAAGTTTCCAATTCTTCATCAGAAGCCAGAACCCGATCGGCTTCACCTCAGAACATTAATGA
AGCTGCATCATCAAAAGATCATCACCTGAAGAAAAATGATCATCCGATTGGTCCATTGGATCCCGAAAA
CTCTAACAATTTCAGGGATGGTAAAAGGGTTGGAAGGGAAGAGAGTCCTGAATCTGAATCACAAGGC
TGGGTGCCCAACAAGGCTCCTAAGCTCAACAACTCTGCAGCTAATAAGCCAATTGATCAATCCACCGA
GGCCACCATGAGGAAAGCCCGCGTATCGGTTCGAGCCAGATCCGAAGCTCCCATGATTACTGATGGAT
GTCAATGGCGAAAATACGGACAAAAGATGGCAAAAGGAAACCCATGTCCTCGTGCATATTATAGGTGT
ACCATGGCTGTTGGTTGTCCTGTTCGCAAACAAGTACAACGTTGTGCTGAGGACAGAACCATATTGAT
CACAACATATGAAGGCAACCATAACCACCCTCTGCCTCCAGCTGCAATGGCCATGGCATCAACCACAA
CAGCAGCAGCAAGCATGTTGCTTTCGGGCTCCATGTCAAGCGCAGATGGGATAATGAACCCAAACAAT
TTGCTAGCCAGAGCAATTCTTCCCTGCTCATCAAGTTCCCAATTTCAAGTCCCTTTCCCAGGGCAACAG
CCACAACTGCCTCAAGTTTTTGGACAGGCCCTTTATAACCAATCCAAGTTTTCTGGGCTTCAGCTGTCC
CAGGATTTAATGGGGTCTAATTCCCAACAGCAGCAGCAACATTTGCCGCATCAAACTCAGTCTGCCTC
GTTTGCTGACACAGTGAGTGCCGCTACAGCTGCCATAACTGCCGACCCAACTTTCACTGCTGCTCTTG
CGGCCGCCATCACCTCCATCATCGGTGGAGGTCATCCGAACAACAACAACAACAACAACAACAGCAC
CTCGACGACCTCCAACAACAGCAATGGAGGCAACAACAACAGCAACAGCAAAATGAGCGGCTTCCC
AGGACATTAA
>PmWRKY25
ATGGACTCTACATTCTTCAACAGCAGCAACAGCAACAGCAACAGCAGAAGCAAGCAGTTTATGAGCG
ATCAAGAAGAGAACGACAATACTACCAGCTCCACACCAGAAAATAGCTCAGGCTCTCCTCCTCCACCT
TCTACCAATTTCAGTGACTTCTCCAAGATCACTTCCACCTCCTCCCCCAAGAAAAGTGCTCGGCGAGC
CATACAGAAACGAGTGGTGTCGATACCCATCAAAGGCGACAACAGCAACACTCCGCCTCCATCCGATT
CTTGGGCTTGGAGAAAGTACGGCCAAAAACCCATCAAGGGCTCGCCTTACCCCAGGTGCAGTAGCTC
AAAGGGCTGCCCGGCAAGGAAGCAAGTAGAGAGAAGCCGCGTGGACCCCTCCATGCTCGTCATCACC
TACTCCTCCGAACACAACCACCCCTGGCCCGCTTCTCGCAACCACCACAACCATCACCAGAGCAACA
GCTCGAGCTCCGCCGCAGCCGCCGCCACCACAACCAAACCCGGCTCCAACAAGACCGAAGCCCCGG
AGGCCCAGCAGCCCGAGCACCAAGACCACGACCCGACATTCGCGGATCTCAACGACGAGTCGCTCCT
CACACACGACGAGTTCGGCTGGTTCGCCGACATGGAAACGACGTCGTCGACGGTGCTCGAGAGCCCA
ATTTTCGCCGAGAGCGGCTGCGCTGGTGGGGCCGACTCCGCTGACATGGCAGCGATGGTTTTCCCGAT
GGGGGAAGAGGACGAGTCTCTCTTCGCTGATCTGGGTGAGCTGCCGGAGTGCTCTCTCGTTTTTCGGC
ACCGGGGAGTGGGACCACAAGTTCAGATCTGTTGA
>PmWRKY26
ATGTCTGATCATGAACATAAAGACCTTTACTACCACGACCTATTTCAGTATGAAGATCATGAACACCTC
AATGGTGGGATGATAAGCATCAATCACAACCAAAACTTGCAAGGGATTGAAGTTCCTTCATACATGAA
CAACACCAGCTTCGCAGAGTGTTTGAAAGGACCTATGGATTACAATACGCTCGCAACTGCTTTTGGCC
TGGCTTCTTCAACGTCTGATGAAGTTATATTTTCCTCCGCTAATGAAGGAGATCAAAAGCCTGCTGAGC
ATATTCGATATTCAGGTGGTGGTGGCGATGGCGATGGTGGAGGTGGAGAAACTCCTTTGACACCAAAC

TCTTCGGTGTCGTCTTCTTCAGCTGAGGCCGGTGCCGAAGAGGACTCTGGTAAGAGTAAGAAAGACA
GGCAGCCAAAAGGGTCATCAGAAGATGGAGGAGATAGCTCGAAGAAAGTGATGAGCAAAGCAAAGA
AGAAAGGAGAGAAAAAGCAAAGAGAGCCACGATTTGCCTTCATGACCAAGAGTGAGGTTGATCATCT
AGAAGATGGATACAGATGGAGAAAATATGGGCAGAAGGCTGTGAAAAATAGTCCATATCCAAGAAGCT
ACTATCGATGTACGACCCAAAAATGCGGGGTCAAGAAACGTGTAGAGAGGTCGTTCGAGGACCCATC
CACTGTGATTACGACGTATGAAGGCCAACACAACCACCCACTCCCTGCAACCCTCAGAGGAAATGCTG
CTGCTTCTTCTGCATTCTTCCCACATGCTGCTGCTGCCTTCAACTATGCGCCCATCACAGGATCTGCAG
GTCCAAGCTCCAACTTTCCTCAAGAACTCTTGTTTCAAATGCCCCACCACTACATGATCAATAATAACA
ATCAAGGGTCATCAAGAAGTGTTGTTGTTAACCCTCATCATCCTCATCATCAGCAGCAGCATCAACTAC
CTCGTGCTGATCAGCATGGACTTCTGCAGGACTTGTTGTTTCCCTCCATGTTCCTCAAACAAGAGCCAT
GA
>PmWRKY27
ATGGAGAATTACCAAGGTGATTTAACTGATATTCTTAGAGCTAGCAGTGCTGCTACTTCCGCCGCAGGA
GCTTACCATTCTGATCATCTTCTTCATCATCATCATCAAGAACCTGAAGCCGCCGCCGACAGCTGGCAC
TTCCCATCTGACCATCCTCCTCCCATGAAATTTTCCTCAGCGTCCTCAGTCATGGAGGAGGAGGAAGA
AGAAGATTCCAAGGACAATTTTGGCGATCCCTTTTCATCCTACATGCGCGATCCACTTCTTCATGAACT
GGACATATCGTCCAGCTCCAGCTTTTTCAGCAGCCCCAATTCCTCATCAGATATGAATATTAACATCACT
TCTGTGGATGAAGCTGGCGGTACAAGTAGCACTTTTGGCGGTGCACATATAGTTCTTGCTCCTCCTCCT
CCTCCACCTCATCACCACCATCTTCATCATCATCAACAAGGTGGTGTTGTTGATGGGGATCCAATAAAG
AGGCCTTGCAACATATTCTCGCGGATGCTTCAGATCTCTCCGAGTGCAAAGGTCCCTGTGACGGCATGT
GACTCGCCGTTGCTGGGTGCAGCAACAGCAGTGTCGTCTCCACGGGGAATTAGTAAGGCGGTGTCTG
GTTCGATATCTGGTGCCATGGTTGGAAGCGACATGATTATTAATGGGAACAGCTCATCAGGAAAAGGTT
GCTTGCTCGAGAACGCCGGGGTGCAGATCTCATCTCCACGGAATCCGGGCATCAAGCGAAGGAAGAG
CCAGGCAAAGAAGGTTGTGTGCATTCCGGCGCCTGCGGCGGCAAACAGCAGGCCAACTGGAGAAGT
AGTTCCATCTGATCTGTGGGCATGGAGAAAGTACGGTCAGAAACCCATCAAAGGTTCACCTTATCCAA
GGGGTTACTATCGATGCAGCAGCTCAAAGGGTTGCTCAGCAAGGAAACAAGTAGAGCGTAGCCGGAC
TGATCCAAACATGTTGGTCATCACTTACACTTCTGAACACAACCATGCCTGGCCAACTCAGAGAAATG
CTCTAGCTGGCTCAACGAGATCCCAGCCATCCAAAAACGGTGCCGGTTCGAAGACCTCTTCCAATAGT
TCTCATCAGCCTCATCAGAAGCCAGCAAGCCCTACAAAGGAAGAACAAAATGAGACCATGAATGATAA
TAATCATGTGTCTCCAGTTATCACTGCTGGGAGTGCTTCAACAGTGAAAGAAGAATTTGAGGACATTG
AGAAGCAATTGAAGCTTGATCATCATCAATTCAACGGTGATCATCAAGCAGGGTTTCCGTATAGACCAT
CAATGCCTGATGATCAGTCCAACCAGTCTCATCACAATGAGGACTTCTTTGCTGATTTAGGAGAAATTG
AGGCTGATCCACTAAATCTCTTGTTTTCACAATGTTTCACAGCAGATGAGCAGCAAAAGGGAAGCAAG
GCTGCAGCCTTGGATCCATTCAATCTCTTTGATTGGTCTGGGGATCAAAACACCAACAACAACAACAA
CAACAACGCCACCTCATTTGGTGAAGCAGCTAAGAGGCGCTTATAA
>PmWRKY28
ATGGAATCTGCAGCTAGTGATCATTCGATTCACAACTGCAGATCAATCGAGCAGCCTCTACTTTCTCCC
GAATTCACAGCCGTTGATCACGATCATCACGGGAAGCGGGTTGTTGATGAGTTGGACTTCTTTGCTGAT
AACAAGGGTCGTCTGATGGAAATGAGAGATCAGACGGTTGAGGTTAAAGAAGAAGGCGCACATGATC
ATCACGGCGTTGGTCAAGAAAAGCAACTACCAGACGTAAATACTGGTTTAAATCTTCTCACAACATAC
ACCAGTAGCGACAAATCATCCATGGATGATGGAACTTCATCGTCCCATAATATGGAAGACAAGCATAGA
ACAAATGAGTTGGCAGTTCTTCAGGCTGAATTAGGCCGTATGAACGTAGAAAATCAGCGATTGAGAGT
TATGATTAGCCAGGTGAACAACAATTACCAGGCATTACAGGTGCAAATTGTGACACTGATGCAACGCC
AGCAAAATCAGAAGGCTGATCATCAAACTCCAGAACAACACAAGATGATCAATAATGGATCAGTCGTG

GTAGAAGAGAAGCAGATGATGAACGGGTTCAACCATATTGTTCCAAGGCAATTTATGGATATGGGCAG
GGCTGAGAAGGATGAGCGTTCACAGTGTCCTTTGGAAGGCTGCAGATCCCAAGATTGTTCCGGGTCGC
CTCCACGAAACGACATCGTTGAGTCAATGGAGTGCTGCAAGAGTACTAGTCATGTACTTCACAGGGAT
CTTAGTGGAAGAATTAGTACTACTAATGGGGGAGAAGATAGCCCTGACCAAGAGTTTCAAGGGTGGGT
TCCTAAGAAAGTCTCCAAGATGATGAGTCCCAGGGACGTGGATCAAGCCTCATCAGAAACTATGTCCA
TGATTAAGAAAGCCCGCGTTTCCGTTCGGGCACGATCTGAAGCGTCCATGATATCTGATGGATGCCAAT
GGAGAAAGTATGGTCAAAAAATGGCTAAAGGAAACCCATGCCCTCGAGCTTATTATCGTTGTACCATG
GGAACTGGTTGCGCAGTTCGCAAACAGGTACAAAGATGTGCAGAAGACCGAACGATACTGGTAACGA
CCTACGAAGGCCACCACAACCATCCACTCCCTCCTGCGGCAATGGCAATGGCGTCTACAACATCAGCA
GCAGCATCAATGCTACTATCAGGCTCAATGCCTAGTGCTGATGGCCTAATAAGCTCAAACAGCTTCCTG
GCAAGAAGTGCCCTACCAAACTGCCCACCAAGTTTGGCAACACTCTCAGCTTCAGCCCCATTTCCCAC
TGTCACATTGGACCTCACTCGAACTCCAACTTCCTCAGAGATGCCACTTGGTCAGCCAAACCAGCTGC
CTCCAAGTTTTCCTCAAAACATGATGTCTGTGCCACAAATTCTAGGTCAAGCCCTTTCCAGCCAATCGA
AATTCTCGGTTCTCGATAGCTTTCAGGGATTGGATAGCGCTACACACTCATTGGCTGACAAAGTCAATG
CAGCAACAGCGGCCATCACAGCTGACCCTAACTTCACAGCGGCTCTGGTAGCAGCCATCACCTCTATC
GTTGGCAATGTTCATTCAAACAATAATACCAACAACAATATTACAACAAGAAACAATAGTGATAGCAAC
ACATGA
>PmWRKY29
ATGGAATCTGCAGCTAGTGATCATTCGATTCACAACTGCAGATCAATCGAGCAGCCTCTACTTTCTCCG
GAAACAGCCGTTGATCACGATCATCACGGGAAGCGGATTGTTGATGAGTTGGACTTCTTTGCTGATAA
CAAGGGTCGTCTGATGGAAATGAGAGATCAGACGGTTGAGGTTCAAGAAGAAGGCGCACATGATCAT
CACGGCGTTGGTCAAGAAAAGCAACTCCCAGACACTGGTTTAAATCTTCTCACAACATACACCAGTAG
CGACAAATCATCCATGGATGATGGAACTTCATCGTCCCATAATATGGAAGACAAGCATAGAACAAATGA
GTTGGCAGTTCTTCAGGCTGAATTAGGCCGTATGAACGTAGAAAATCAGCGATTGAGAGTTATGATTAG
CCAGGTGAACAACAATTACCAGGCATTACAGGTGCAAATTGTGACACTGATGCAACGCCAGCAAAATC
AAAAGGTTGATCATCAAACTCCAGAACAACATAAGATGAACAATAATGGATCAGTCGTGGTAGAAGAG
AAGCAGATGATGAACGGGTTCAACCATATTGTTCCAAGGCAATTTATGGATATGGGCAGGGCTGAGAA
AGATGAGCTTTCACAGTGTTCTTTGGAAGGCTGCAGATCCCAAGATTGTTCCGGGTCGTCTCCACGAA
ATGACATCGTTGAGTCAATGGAGTGCTGCAAGAGTACTAGTCATGTACTTCACAGGGATCTTAGCGGA
AGAATTAGTACTACTAATGGGGGAGAAGACAGCCCTGACCAAGAGTTTCAAGGGTGGGTTCCTAAGA
AAGTGTCCAAGATGATGAGTCCCAGGCACGTGGATCAAGCCTCATCAGAAACCATGTCCATGATTAAG
AAAGCCCGCGTTTCTGTTCGGGCAAGATCTGAAGCGTCCATGATATCTGATGGATGCCAATGGAGAAA
GTATGGTCAGAAAATGGCTAAAGGAAACCCATGCCCTCGAGCTTATTATCGTTGTACCATGGGAACTGG
TTGCCCAGTTCGCAAACAGGTACAAAGATGTGCAGAAGACCGAACGATACTGGTAACGACCTACGAA
GGCCACCACAACCATCCACTCCCTCCTGCTGCAATGGCAATGGCGTCTACAACATCAGCAGCAGCATC
AATGCTACTATCAGGATCAATGCCTAGTGCTGATGGCCTAATAAGCTCAAACAGCTTCCTGGCAAGAAG
TGCCCTACAAAATTGCCCACCAAGTTTGGCAACACTCTCAGCTTCAGCCCCATTTCCCACTGTCACATT
GGACCTCACTCGAACTCCAACTTCCTCAGAGATGCCACTTGGCCAGCCAAACCAGCTGCCTCCAAGTT
TTCCTCAAAACATGATGCCTGTGCCACAAATTCTAGGTCAAGCCCTTTCCAGCCAATCGACATTCTCGG
TTCTCGAGAGCTTTCCGGGATTGGATAGCGCTACACACTCATTGGCTGACAAAGTCAATGCAGCAACA
GCGGCCATCACAGCTGACCCTAACTTCACAGCGGCTCTGGTAGCAGCCATCACCTCTATCGTTGGCAAT
GTTCATTCAAACAATAATACCAACGGCAATATTACAACAAGAAACAATAGTGATAGCAATACATGA
>PmWRKY30
ATGGACGCGACCACACTCGACCACCCCTCTGGACCCTCCGATGACTTCGATCCGGGTCTAACCGACTT

CAATCCCGGGTCTGACCCGACCCTTTTTTCTTCTACTGGTGGTGGTGGTGGTGCAAAGTACAAGCTCAT
GTCCCCGGCCAAGCTTCCGATCTCGAGGTCACCCTGCCTCACTATCCCTCCCGGCCTCAGTCCGACGT
CGTTTCTCGAGTCCCCCGTCCTCCTTTCCAACATGAAGGCAGAACCTTCCCCAACTACTGGGTCCTTTT
TGAAGCCTCAAATGGTGTACGGTTCTCTGAGTTCTACTACATATTCAGCAAACACAATGTGCTCTGATT
TCGATACCTTTGATGAAAGAAATTCTGGAAGTTTCGAGTTTAAGCCCCATGCTGGATCAAATATGGTTA
CTACAGATTATAACCACCAGAGAAATGACCAGTTAGTCCAAGGTCAAGCTCAGCCTCAATCACTCGTG
TCTCCACCTTTGGTTAAAAGTGAGATGGCGGTCTCCTCAAATGAATTGAGTTTGTCAGCACCTGTTCAC
ATGGTCACTTCAGGAGCTAGTGCACCTGCTGAAGGTGATTCAGATGACTTAAGTCAGAGGGGACATCC
AAATCCTGGGGTCCAAACATCACAGTTTGATCATAAAGGAAGTGGGCCTTCAGTCATATCTTCTGATGA
TGGTTATAACTGGAGAAAATATGGACAAAAACACGTTAAAGGAAGTGAATTTCCTCGCAGTTATTATAA
ATGTACCCATCCTAATTGTGAAGTGAAGAAGCTGTTTGAGCGATCTCACGATGGACAGATAACAGAGA
TTATCTACAAGGGTACACATGATCATCCTAAGCCTCAACCTAGTCGACGATATAATACTGGTGCTATGAT
GCCTATCCAGGAAGAAAGATCTGAAAAGGCTTCCTCTTTGATCGGCCGTGATGACAAGCCATCCAGCA
TATACGGGCAAATGTCGAGTACTAATGAGCCAAATAGTACTCCTGAACGATCTCCTGTCACAGGAAATG
ATGATAGTGTAGAAGGCACAGGTTCACTATCTAATAGGATGGCCGAGGAGATTGATGATGATGACCCAT
TCTCAAAAAGGAGGAGGATGGATGTTGGTGGGGTTGATGTCACACCAGTTGTTAAACCCATCCGTGAA
CCACGAGTTGTTGTTCAGACTCTGAGTGAGGTTGATATATTGGATGATGGATATCGCTGGCGGAAATAT
GGTCAGAAAGTGGTGAGGGGAAATCCTAATCCAAGGAGCTATTACAAGTGCACCAATGCTGGATGCCC
TGTTAGAAAACATGTGGAGAGGGCATCCCATGATCCAAAAGCGGTTATAACTACATATGAGGGGAAAC
ATAACCATGACGTCCCAACTGCAAGGAATAGTAGCCATGACACTTCAGGACCAACAACTGTGAATGTC
CCATCGAGGATTAGATCAGAAGAAAGTGACACCATAAGCCTCGATCTTGGTGTTGGAATCAACTCTGC
TGCTGAAAATAAATCCAACGAGCACCTGCAACTGCACTCTGAACTAATGGAACGCCAATCTCACACTA
GTTCCAATTTTAAGGCCATTCAAACTACCCCTGTTTCAACATACTACGGTGTTTTAAATAGTGGCATGAA
TCAGTACGGATCTAGAGAAAATCCAAGTGAATCACGTAGCATCGAGATTCCACCTTTAAATCATTCCTC
TTATCCATATCCACAGAACATGGGAAGGAAAGATCTGCAACTTTGCAAAGATAAAATACTGCACCAGT
ACGAGTTAACTAGAATTCACACAAACACACTCCCACTCAGTCACTCGGTCAATCAAACAGAGATGCAT
GCTTTGTGGGTGTCTTTGAAGGACAATGTCAGGTGCAATGGGAACAAGGTGGCAGATGTAGTTGGCC
GGGCATCTGAAATATGTAACAGAAGAAAAAGCAGTTCAGGAAAGGCAAATTCTGGTGGTGAAATTGT
TCATGGCAAAGAGACTCCGGCCCGAGAATTGATGCACCGGGGAAGCTACACACAAGCACAGCTATAT
GAGCTAGACATTGGAGACCCATCAAGGAATGTCATTGAGATGATTGTCCGAAGAGCAACGGTAAATCC
ATCAGAGCCATCAAACAGGATTAAAAGGGTCCTGAGAGTGCAGAACTCCATAGAAACGCTTGAAAGG
TTTGAGAAGTACAGAGAGATGGTAAAGAAGATGGCCAAAGAGAGATATATGAGGCATCCAAGAAGCA
CAGTAGATGGAAACGAGCTGTTGAGGTTCTATGGCACAACGGTAGCCTGCTGCAGTGGCGAGTCAAA
GCGAGTCTCTGAGCTTTGTAAGGCTCCAAATTGTCAAGTTTGCATGATAATTCAATCAGATTTTTACAC
AGAATATAGCGTGAGCAATGAGATTCAACTGAGTACAAGTAGCGAAAAATTCGGTGAAAACAGCATTA
CCATCACAAGGAGGAACAAGATAAGAAGGGCTGTAATAGTTTGCAGGACAATTGCAGGGAGCATGGT
TAATATGACAGACAAGGAATATGAAGAGTCTGATTCAATTGAAAGCCAAAAGCTGCTTTCTACTTCAG
AATATTTGGTAGTGAGGAATCCCTCTGCTGTACTTCCCTGTTTTGTCATAGCTTTCACCTGA
>PmWRKY31
ATGGAAAAACAAGGCCGTCGTCATCACATAATCCCCAAACTCTTCCCCCACTCCGTCTCCGATTGCTCA
TTTAATGCTTCCGATGATGGTCTTGTTGACGCTACCAACAACACTAGTAGCAGCAACCCCTCAATCCCA
ATCCCAATCCCAATCCCAATCAAACAACTTTTCAGACAACCAAAAGAAATCAAAGTAATGGATTTCTTT
TCCGATAATAATATCATGAATGATGATGCTGATGATGATGAGGAGCCCCGTCGTCAACCCCAACAAGAC
TGCAACAATAATCCTCCGGCTGTTAACACAGGATTGAATCTCCTCACTCTAAATTCCGGAATTTCATCA

ACATCTGCCAGTGATATTCATCAAAATTCCAATAACAAGCTGATGACTAGTCTTCAAGTTGAGTTAGAG
CGGCTACATGAAGAAAATGGCGAGCTCAAAACCATGTTAGACCAAATGACCAAAAGTTACAGCCAGT
TGCAAGCTCAGCTACTCATGGCGATGCAGAAACAGGCACAAAACAGACTACGAGAGCCGATTAAGTG
TGAGGCAAATGGCATGTTGGCCAGACAATTCATGGATCCACGGCCATCTACAGCTGCTGCAATGGACC
ATGTCCGCGATCCATCAGTTGCCTATTCTTCTGGAAAGACACCGGCTGATCATGAAGCCTTCTCATCATT
TGCTCCATCAAATTTGAATATAGAGGTGATGTCAATGGAGCGTGACCAATATCAGAGGAGGCTGCAAA
CTAATATTAATTGTGCGGAAGAAGCTCTTGACCGGTCATCCCAGTGTCCAGGGTCCCCTAATTATACCA
GTAAAAGTAGCGATCCCAATTTATTTGATGATGATAATGATGAGCCCAAACGTAGTACTGATCAAGAAC
AAGTTCCAGTGGCTGATCAGATTCCTTTTAGGAAGGCAAGGGTTTCCGTACGAGCAAGATCAGAAGCT
CCTATGATTAGCGATGGATGTCAATGGAGGAAATACGGTCAAAAGATGGCAAAGGGTAACCCTTGCCC
ACGTGCTTACTACCGTTGTACTATGGCCATTGGATGCCCCGTTCGCAAACAGGTGCAAAGATTGGCGG
AGGATAAAACCATTCTCGTAACTACTTATGAAGGTAATCACAGCCACCCTCTGCCTCCAGCAGCCACA
GCCATGGCCAAAACCACGTCAGCAGCAGCTGCCATGCTGCTATCAGGTTCAACTACAAGCAAGGAAG
CTCACCAGTATCATCATCATCATCTAGCAAATTCTGGCTTCTTCTCCAATTCCCAGCTGCCCTTCTTCAC
TTCGTCCATGGCCACATTATCAGCCTCTGCACCATTTCCCACCATCACGCTGGACTTGACTCAGAGCCC
CATGCAGCAATTCCACCGAATCCCGCCACCTTCCTCATCCACATTTCCTCTGCCTTTGCATGGTTATCAC
CAGCTTATGGGTGGGCTGGGGCATCCTATCCAAGCCCCAATGTATTTCCCTCCTAATTACAAGGCACCC
CCACCACCTGCTGGAGTGCCGCTAGGCGGCCAACGTAGTACTAGTACTCATGATAGTGGCATGATTGA
GACTGTTGGTGCAGCCATTGCTTCTGACCCCAATTTCACTGCAGCCCTAGCTGCGGCCATCTCAACTAT
TATGGGGGCACCCCGCCCCCACCAGGGCCACCAGGGGGGGATTAATATTAATGATGGAGACATCGCTA
ATAATAATGCAGCTAGAGGAGTAGTTGCAAGTACCAATCATTCCCCTCCTAGTGCTAATATTGGGGTTCC
ACCCGGATCATTACAGCCTGGATCCCCACAACTTCCTCAGTCCTGCACCACTTTCTCCACCAACTAA
>PmWRKY32
ATGGATGTGAAGAAGGATGTGGTAAAGATGGAGGACAATAGTACCAATATTGGATGTGGGTTTTCCTCA
TCACCATTTTCAGGCATATTTGATTTCTGTGAAGGAGAAAAGAGCTCATCTTTAGGGTTTATGGAGCTT
TTGGGTGCTGGCCAGGACTTTTGTACTAACTCCTTGTTTGATTATTTGCCTCAGACACCATCTATGTTGC
CTTCATTAGCTCCAAACTTTCCAAATACTTCCATAATGGCAAAGGAGTGCTCTGACTACTCTTTGAATC
AGCAGCCTGCAACACCAAACTCTTCATCGATTTCATCTGCATCCAGTGAGGCTCTGAACGAGGAACAG
ACTGATAATAAAGGTGCTGCAGACCAAGATGAAGAAGAAGAACGTGACCAACCAAAGACCAAGAAA
GAGTTGAAAGCGAAGAAGGCAAGTCAGAAGAGACAGAGAGAACCCAGATTTGCATTCATGACCAAG
AGTGAGGTTGATCATCTGGAAGATGGCTACAGATGGAGAAAGTACGGTCAAAAAGCTGTGAAAAACA
GCCCCTTCCCCAGGAGTTACTATCGATGCACAAGTACAGCATGTAATGTGAAGAAGCGAGTGGAGCGA
TCTTTCAATGACCCAAGCATCGTTGTGACAACTTATGAAGGCCAACACACACACCCAAGCCCACTCAT
ACCTCGCCCAACTCTCACGGCTTCGGCTTCTGCTCAACCCAATATCTCTACCACCTTTGCCATGCCTTC
AATGCCCAGAACCCTATTATCTCATCACTATCAGCAGCAACTGCAACCTTTCAATTTCTGCAACTATGTT
AATGGTGGCTCACCAACAGCAAATGCTAGCGGCACTGGCTTTCATCACGAAAGGCGGTTTTGCACCCC
AGCAACTGGTTCTGCTATGTTGACAGACCATGGGCTTCTTCAAGACATAGTCCCCTCCCATATGCTGAA
GCAAGAGTAG
>PmWRKY33
ATGGACTACTCATCTGCTGCATATGATGATACTTCTTTGGATCTTAATACCAAGCCTCTCCGACTTTTCGA
TGATACTCCGATCAAGAAAGAGGCGCAAAGCAAAATATTGATTGGCTTTGGGAGGCAGCTTTCACCAG
ATGAAGAGAGTGGTGCTCTATTGGAGGAATTGCAACGGGTGAGTGCAGAGAACAAGAAGCTAACCGA
AATGTTGACGGTGATGGGTGAGAGCTACAATGGTTTAAGAAACCAGTTGCTGGATTACATGAGCAAGA
ACCCAGAGAAGGAGCTTAGCCCAATTTCAAAGAAAAGAAAGTCTGAAAGCAGTAACAACAACAACA

CCAACAGCAACAATAACATCAATGGAGCAGTGAATGGAAACTCTGAGAGCAGCTCCAGTGATGGAGA
ATCTTGCAAGAAACCAAGGGAAGAGAACATCAAGGCAAAGATTTCAAGGGCTTATGTTCGTACCGAA
GCATCAGATACAACAAGCCTGGTTGTGAAGGATGGATATCAATGGAGAAAGTATGGCCAAAAAGTTAC
TAGAGATAATCCTTGTCCTAGAGCTTACTTCAAATGCTCTTTTGCTCCAAGCTGCCCTGTCAAAAAGAA
GGTGCAGAGAAGTGTTGAAGATCAATCTATTCTGGTGGCAACTTATGAAGGTGAACACAATCATTCCC
ACCCTTCTCAAATTGAAGCAACATCAGGCTCAAACCGCTGCATGACCTTAGGATCAGTCCCCTGCTCA
ACCTCCCTTGCCTCATCCGGACCTACCATCACTCTTGACTTGACCAAATCCAAGTCCAGTGCTGACACC
AAAAGTACGAAAACAAAAACCGAAACACCGGAAGTTCGAAAGTTTTTGGTGGAGCAGATGGCTTCTT
CCTTGACAAAAGATCCCGATTTCACGAAAGCACTAGCAGCAGCCATTTCAGGAAGAATACTTCAACAT
AATTCTTACTGA
>PmWRKY34
ATGGACCCACAATTCTACAGAATTTCCCCATTTGGGACTGACCCAGCAGACCCTGATCAGCCCATGAC
GTCAGAGAATGGTCCGGGCTCTCCCTCCTCAGGGGAGGAGACAAAGGTGGCCACTGCCCCATCACCC
AAGAAAAGGAGGGGAGTGCAGAAGAGGGTGGTGACAGTACCGATCGGTGACGTGGAGGGATCCAAG
AGCAAAGGGGAGGGCCATCCACCGTCCGATTCTTGGGCTTGGAGGAAGTACGGCCAAAAGCCCATCA
AAGGGTCTCCATATCCCAGGGGATATTATCGATGTAGTAGCTCCAAGGGTTGCCCGGCAAGGAAACAA
GTGGAGAGAAGTCGTGTGGACCCCACAATGCTCTTGATCACCTATGCTTGTGAGCACAACCACCCCAA
GCCCACCACCAAGCCACACCAAACCTCCACCACCACGTCACCCAACGCCGAACCCGAACTTCGTGCC
AAAACAGTCACACCCAATGAAGAGGAGCTCACAATTTTTGCGAGCCAAGTCGACCTTGACCTTAGTG
ACGACTCGGCCACGTTGCTTAGTGCCTTCGGTTGGTTCAGTGACGTGGCATCGACAGCTGTTCTAGAA
AGTCCGATTTGTGCGGGAAATAGTACTTGCGCTGATTATGACGTGGCAACGAGGTTGGGAGATGAGGA
GGACGAGTATTTGTTCGCCGACCTGGGCGAGTTACCGGAGGGTTCAGTGATTTTCCGACACAAGATGG
TGGAATCGGACGAGCAGAACCGGAGATGTAGTTTAAGTGTAGTTCCTTGTTGCAATAGTAGATGA
>PmWRKY35
ATGTCAAATGAAAAGAAAAACCCTTACCAGTATGACCCTTTCGACTACAACCCCCATGAAATCAACAG
GTCAAGCTTTCCATTCTTCAATTATGGCACTCCCTCCATACAAGATCCACAAAACCTACATGGGTTCGA
ATCCGATCACCCCAATTCTTCGTTCATGAGCTTCACTGACTGCCTCCATGGCTCAATGGACTACAACAC
CCTCTCAAGAGCCTTTGACATGTCATGTTCTTCATCTGAAGTCATTTCTCCCCAGTTGGATCATGAGAAT
TCCAAGAACCAGCAGGCTGCTGCAGCTGCAGGTGTAGGAGATCACTCGGTGGGGACTAGCACCACCG
AAAACCCATCTACACCAAACTCCTCAGTTTCTTCATCATCTAATGAAGGTGCTGGTTCTCATGAACATG
AAGATTCAGAGAAGAAGAAGAAAGAAAAGCAGCCAAAAGTGGCAGTGTGTGATGAAGCGGCAGGA
GATGAGGAAGACAAGTCAAAGAAAGGGAGCAAAGCGAAAAAGAAAGAGAAACGGCAGAGGGAACC
ACGGTTTGCCTTCCTGACTAAGAGCGAAGTGGATCACCTTGAAGACGGATACAGATGGAGAAAGTAC
GGACAGAAGGCAGTCAAGAACAGCCCTTATCCTAGAAGTTATTATAGATGCACTACTCAAAAGTGCGT
AGTAAAGAAGCGCGTCGAGAGATCATTTCAAGATCCATCTATTGTGATCACAACATATGAGGGTCAGC
ACAACCATCAGTGTCCGGCAACACTCCGGGGAAATCTCAATCTCAATGCCGTTGGAATGCTGTCCCCT
AATTCCCTTTTGACATCTGCGTCTCTCAATGGATCAGCGAGATTTCAACATGAATTCTTAACTCAGTTTC
TCCCAATGAACAACCAATTGCAATTGCAATTGCAAAGCCATCATCATCAGGATGATCACCAAGCAAGT
AATTCCATGATATATTCAAACCTCGTGGCCCCTCGCCCTCATCATCATCAGCAGCAGCATCAGCGGCAG
CATCAGCGGCAGCAGCTACATGTTCCTGACTATGGGTTATTGCAAGATTTAGTTCCTTCATTTGGTCACA
AGCAAGAGCCATGA
>PmWRKY36
ATGATTTCTCTAGGGGAACCTGGGACAGATAAAATTGCTTCTGATATAGTACCGAAGAAAGAGAGTTC
AAATAGTGAAATTCATGCCCCACATCAAACTCCTGATAATGGGATCTGTTCCCTGCAATCAGATCATAG

AGGAAATGTTCAGTCTCTAATACCTGAGAAATCGTTACAGCTGCCTGATGATGTTGGTACTGCATCTCA
ATCAAATCAAGAAGGAAGTGTTACCTCTTTAACATCTGAGAAAGCACCACAAACCCCTGAAACCTCTG
CCCTTGTCTTGCGATCTGGTCAAGAAGGAAGCACTCCATCTACAGCACGAGAGAGAGGGTTAGAGGA
TGGTTATCACTGGAGAAAATACGGCCAGAAACTTGTTAAGGGAAATGCATATGTACGAAGTTACTACA
GATGTACGCATGCAAAATGTCCAGTGAAGAGGCAAGTGGAGCGCACACATAATGGGCAGATAACAGAT
ACTGTTTACTTTGGTGAGCATCAACATCCTAAAGCTCAAGTTAACGTCCCAGTAGCTGTTAGTTTTCTC
GTGTCCATTGTCGAAGAAAGACCAGAAGAGCTTTTGTTAACTGGTGTCGAAGGCAAATCATCGGATGT
GCATGGCCACACATCTAACCAGATTGAGCCAGTGGATCCCGCTCAGCTATCAACCGTTGCAGATAATGA
AGGTGTGCAGAGAGTGCTCTCTCAATCAAATAGAACCAGAGATGGTGATCCAGACTCAAAAAGACAG
AAGAAGGAAAAACATAATGGCAACTCAATTCCGGTGGATAAGCCAGCTGGTGAACCGCGTGTTGTTGT
TCAGACTATGAGTGAGGCTGATATAGTGAATGATGGCTACAGATGGCGCAAATATGGGCAGAAGTTAGT
AAAAGGCAACCCAAATCCAAGGAGTTACTACAGATGCTCAAATCCTGGGTGCCCTGTTAAGAAACATG
TAGAGAGGGCGTCTCATGATTCAAAAGTTGTTATAGCCACATATGAGGGGCAACATGATCATGATATGC
CACCCACAAGGACTGTGACCCACAATGCAGCAGCATCAAATGTGATTACAACGGCCCGTAATGGTGAG
TCTGGCACTACGTCAGAAGGGAATGCTGTCTGCCATGATACTAGCCCAGAACACGAAGATAAACCAAA
CAAGCAACTTAATGTTGAGCCAAGAACTAAATCAAGTGATGTTGCTGGCTGTGATATGGTCGTTGATTC
TGATCTGGGTCCTGAAAGAAAATTAAATGAGCAAGTGGTTGGCAAAGCATGTACCACAGAAGAAAGT
GATGCCCCCGATATAATTGTTCCTAGGGCCAATGAATTGCAAAATGGCGAGTCAGGAATTAAATCAGAA
GGAAACAACGCCTGCATTGATACGGTCATTCACGGCAATCTGTGTCCCGAAAGTAATTCACCGGAGCA
AAAAAATCCAAAAGCAGAACCTGTCTAA
>PmWRKY37
ATGGCCAAGGGAAGTGGACTCTCCATTGATTCAGATCCATTTGGGTTCTCTCTCCACAATCCCATAGTA
CTCAACTCCTTTCAACAAGACCAATATAACCACCATCAACCCTGCAGGACCAAGAAGCAGCAGCAGC
AGCTTCATCACTCCTCACTCAACATGGACATGGACGCCTCCACAATTCACCCGAGGTCTCCTCCTCCCC
CTCCTCTTCCTCCTCCAACTCTGCAATTCTCAGTCAACCTCAACTGCACCCATGAAGATGTTGATAATC
AACATCATCATCACCACCATCAGCACTCTCCTGAACCACCCTCCAATGAAAAACGTAAAGTCATCGAC
GAGAGGGACTTCTTCGCCGATAACAAGAGCCATGTTGATCAAGACAAGTCTGCCTCCGCCGACCCTGC
TGATAAAATGGACTTACATGGCCCGACGGATATGGAATTCAACGTAAATACTGGCTTGAATCTTCTCCTT
ACAAACACTAGCAGTGACCAATCAGTGGTGGATGATGGCATTTCGTCGAACATAGAAGATAAAAGAGC
TAAAAGTGAGCTGGCTGTTCTTCAAGCTGAGCTTGAGCGAATGAACGCGGAGAATCAACGTTTGAGA
GGCATGCTTAATCAGGTGACTACCAATTACAACGCTCTTCAGGTGCATTTGCTGACGTTGATGCAAAGC
CAGAAGGCTGAGCAGAACAGTAGCGCTGCTGAAGGCCATGGAGTGTTTGATGGGAACAACAAAATGG
TGGTGGAAGAAAAGAAGCTCATTAATGGCAATGGATCGCCAGTAGTGGTCCCTAGGCAGTTTATGGAT
CTCGGGTTGGCTGCCAATAATGCTGACGCTGATGAGCCTTCACAGTCTTCATCTGAAGAAAGAAGCCG
CGAACGGTCTGGATCGCTTGGAGAGAATGTGAAGGTTGCTGGGCATAGTGATGATCAGGAGAAGAAG
GAATTTGGAAGAGGGATTGGGAGAGAGGAGAGCCCAGATCAGCCATCACAGAGTTGGGCCCCTAACA
AAGTTCCAAGGCTCAATTCTCCCAAAGAGGTTGATCAAACTGAGGCTACCATGAGGAAGGCAAGAGT
TTCGGTCAGAGCTCGATCAGAGGCACCTATGATCACTGATGGATGTCAATGGCGAAAGTATGGACAAA
AGATGGCAAAGGGAAACCCGTGTCCTCGAGCTTATTATCGATGCACCATGGCTGCTGGTTGCCCAGTT
CGTAAACAAGTACAAAGATGTGCAGAAGATAGGACAATCCTAATTACTACATATGAAGGCAATCACAA
CCACCCATTGCCTCCAGCAGCCATGGCAATGGCATCAACTACTTCATCCGCGGCACGAATGCTACTCTC
GGGGTCCATGCCGAGTGCAGATGGCCTAATGGACTCAAACTTCCTCACCAGGACAATCCTCCCATGCT
CCTCTAGCATGGCCACCATCTCAGCCTCAGCACCATTCCCTACTGTTACATTGGACTTAACACAATCAC
CAAACCCTTTACAGCTCCAAAGGCCACCAGGCCAATTCAACATCCCATTCCCAAACCCATCTCAGAAT

TTCACCAATGGACCCGTCTCATTGCTGCCTCAGATTTTTGGTCAAGCACTCTATAACCAGTCAAAATTC
TCTGGCCTGCAAATGTCACAAGACATGGAGGGTGCCCAACTGGGTCACCAACAGCAACCAGGGCACC
AAGGGCAGCAGCAGAACTCATTGGCTGACACTGTCACTGCAGCCACTGCCGCCATTGCAGCTGATCC
AAACTTCACTGCAGCCCTGGCAGCAGCCATCACCTCCATTATTGGCAATGCTCATCCAAACAACAATAG
TAACAACGGCACCAACCCCGCAACCAACTCAAACAACAACAATGGCAATGGCAACGGCAACGCCAC
CAGCAACAACAATAAACTTAGCAATTCAAGTTTTCCAGCCAACTAA
>PmWRKY38
ATGCCAAACTCTAATAGTAGAACAGCCCCAGAGAAGAAAGTTGATGAATCTGTTGGCTATCAAGAGAT
TTACAGTCAGGAGGAGAGTGAACTTGAAAATGCCAGAGCTGAAATGGGTGAGGTGAGAGAAGAAAA
TGCCAGACTAAAATTGACCTTACAGCATATGGAGAAGGATTACCAGTCTCTCCAATGTCGTTTTTTGA
CATCCTTAGACAAGAGGCTTCCAAGAAAGCTACAAATGTTGATGTTGGTGTTCATCGAATTGAAGAAC
CCAATCAGCTCTTGTCTCTTTGCCTCGGAAGAAGTCCAAGGGAGCCCAAACATGATGAAACTAATACC
GCCAACTTCAAACTTGTACAAGTTGATCATGAAGACTTGAATGCCAACCTTACTCTTGGATTAGGTAAC
TCTAAATTAATGGAGTTGCCTATGGAGCTTGTGCGTAGCCAAAAACCTCAGGAGACTAGTTTGGAAGA
GCCCAAGGATCATTCAGAAGCTGGAGCTGGAGAATCTTTGCCTCCATCAAGTAAAACACCAAAGACA
ACGAGAAATGAAGATGATGAAGTTCCACAGCAGGCCAATGCAAAAAGAGCTAGGGTTTCCGTCAGAG
TTAGATGCGATACCCCAACGATGAACGATGGATGCCAATGGAGAAAATATGGACAGAAGATTGCAAAA
GGAAATCCATGCCCACGAGCATACTACCGCTGCACAGTTGCACCAGCATGCCCTGTGAGAAAGCAGGT
GCAAAGATGTTATGAGGATATGTCCATCTTAATCACCACCTACGAAGGAACACACAACCACCCGCTTCC
ATTCACAGCCTCTTCCATGGCTTCCACCACTGCTGCGGCAGCTTCCATGCTATTGTCTGGCTCCTCCAC
ATCTCAGCCGGGTTTCGGTTCCACAGCCACTCTACTCAATGGATCAGATTTTGGTGTCTTTGATAGTTC
AAGAACAAACCAGCTCTACTTGCCCAAATCCAACCCTTTGCTCCCCACAATCACTCTAGACCTCACTG
CCTCTCCATCCTCTTCACCAATCCATTTGAATAGGTTGTCTTCTAGCTTTGCTTCAGCTTGCACATTCCC
TTCAGGCCTCAGCTTTTGTTCCTCAGAGTCCAACATTTCGCCCAACAGTAGGGGCAATGGATACCTTAA
ATATGGCTCACTGCCGTTTGACAAAGGGTCCTTCAATCTAGATCAACCTTACGCAGAAAAGAATCACC
AATCTTCTTCTCAGGTGTCTTTAACAGAATCTTTAACCAAGGCAATCACGTCAGACCCTAATTTCAAAT
CAGTAATTGCCGTAGCACTTTCATCCATGGTTGGAGGTGGTGCTGCTACTCATGGAAACCAAAGCGAA
AGATTAGGACACCACTTGAAGTGGAGTGAAGCTGCTCATCAGTTTACTTCTCATAATCCATTGATACAG
AACGGGAAAGGATGCAGCACACCGAGCATTTTCAACAGATTGTCATCTTCAGACTCTCAAAAGTGA
>PmWRKY39
ATGGCCAAAAACCAAGACTCTGAGAGAGTCTCAGTTTCAGCACCACCCCAACCGCAAAGACCAGTCA
TAACACTGCCACCGAGGCCTTCCGCTGAGGCCCTTTTCAGCGGCGGATCTGGAGCCAGCCCGGGTCC
GATGACCCTGGTCTCAAGCTTCTTCCCCGACACATACCCTGACTCAGAGTACAGGTCCTTCTCTCAGCT
CCTGGCCGGAGCCATGGGCTCGCCTATGGGGTCCACAAGGCCAATCCAATTTAACGAAAACCCAGTTG
ATGGTTCGGCCCAATTAGAAGGAGGCTCTGAAAATGGCGGTGAGAACAAGTCTGGGTTTAAGCAGAG
TAGGCCTATGAATTTGATGGTGGCTCGGTCGCCATTGTTCACTGTTCCACCTGGATTGAGTCCCTCTGG
GTTGCTTAACTCGCCTGGCTTCTTTTCACCTCCGAGTCCATTTGGAATATCACATCAGCAGGCCTTGGC
GCAGGTTACTGCCCAAGCTGCACTAGCCCAATCTCGTATGCACATGCAAGCTGAATATCAACCTTCGTC
GGTAGGAGCTCCCACAGAGCCACAGGCATATCATCCATCCGTAATGCCCAATGAAGCATCTCAACAGC
AGACATTACCTTCAACATCTGACCATAGAAGTTCCGCGAGGCAATCATCAGAGGCATCTCATTCTGATA
GGAAATACCAACCTTCATCTGTGGCCACTGATAGGCCTGCAGACGATAGCTACAACTGGCGGAAATAT
GGGCAGAAGCAGGTTAAGGGCAGTGAGTATCCTCGAAGTTACTACAAATGCACACATCTGAATTGCCC
TGTCAAAAAGAAAGTGGAGCGTTCTCCTAATGGCGAAATAACTGAGATTATATACAAAGGGCAGCATA
ACCATGAAGCCCCTCAACCTAAACGTGGAAAAGATGGTGGTGATCTGAACGGACACTTGCATTCGCA

GCCTAGGCCTGAAAATGGATTGCAAAGATTGGTTGGAGATTCAAACGGTTCTAGTGAAAATATAGCTTC
TCACTCAATGCTTGAGAGGCATCAAGAATCTACTCAAGCTGCTCCTGGACAGTTACCAGGGGCAAGTG
ACAGTGAAGAGCTACGCGATGGGGAAATTAGAGAAGAGGGGGATGCTGATGAACCAAATCCAAAGAG
AAGGAACATAGATGTTGGGGCATCTGAGGTAGCTTTGTCACACAAGACAGTGACAGAACCAAAAATC
ATTGTGCAAACAAGGAGTGAAGTCGATCTTCTAGATGATGGCTACAGGTGGCGCAAGTATGGACAGAA
GGTGGTCAAGGGGAATCCTCATCCAAGAAGCTATTACAAATGCACTTATGCGGGGTGCAATGTCCGTA
AGCATGTTGAGAGAGCTTCCACTGACCCCAAAGCTGTCATCACTACATACGAGGGGAAACATAACCAT
GATGTTCCAGCAGCTAGAAACAGCAGCCACAACACAGCCAATAACAATGCATCACAGTTAAAACCAC
TGGCGGTTGTGAGAGAGAAGCATCCCCTTCTTAAAGGGAGAGAATTCGGAAACAATGACCAGAGACC
AGTACTTCTACAGCTAAAAGAAGAGCAAATCTTTGTGTGA
>PmWRKY40
ATGGAAGGAGATCAAAGAGGGGTTCCGAGTTATGAAGTTCAGATTTCGTTTTCGAGCACCCCAAACCC
TCAACAATCGATCCACGAAATGGGGTTTGTGCAGTTCGAAGATCATCATCCTCATCATAATCAGGTTTT
GAGCTTCATGGCCCCATCATCACATATACAGCAGCAGCAGCCTAACAATTCTCATCACCCTCATCATCAT
CAGCCAGCTGAGCTCAGCAGCGGTGCCTCCGCCGCCACCACCACCACCAGCAACGGGGTTACCGTGG
GGTTTAGTACTCATACTGACCTACTTGTTGCAAGACCTTCTTGGAATAATAGTAACGACCAGGTGGGAA
CGTTGGATCCAAAGGCTATTAGTGATGAAAATGGGACTGGTAATGCTAGTGATTGCAGCAACTCATGGT
GGAGGAGCTCAAGCTCAGAGAAGAGCAAGATGAAGGTGAGGAGAAAGCTTAGGGAGCCAAGATTCT
GTTTCCAAACCAGAAGTGATGTGGATGTGCTTGATGATGGTTACAAATGGAGGAAATATGGCCAGAAA
GTTGTCAAGAACAGCCTTCATCCTAGAAGCTACTACCGCTGTACTCACAGTAACTGTCGGGTGAAGAA
AAGGGTTGAACGACTGTCTGAAGATTGTCGAATGGTGATAACAACCTATGAAGGTAGACACAACCAC
ACCCCTTGTGATGACTCTAATTCCTCCGAACATGAATGCTTTAGCTCTTTCTGA
>PmWRKY41
ATGGAAGGTCATCAAGAGCCTAATTATCCACCCCCATTAACCCCATCATCCTTAATCTCATTGCCTTTCT
TGTTGCCACCCCAAAACCCTCTCTTCACACCCTCATCAGCATCAACAGCCCCATCCTCCTCCTCACAGC
TGATCAGCCCTCCTCTTCTAGAACCTCAGCAGCATCAAGTCCTCCCAGACATTGATTGGGTCAGCCTTC
TCTCTGGTTCTCCAGCAGCAGGGTTTGATGGGCAGGAGATTAATAATTATAAGCCACTGGTGGAAAATA
ATAATGTTGGTGCCTCTGCTGAGAATATTAATGAAGCTGATCGGGAAGGAAAGGGTGCAAATAGTAAA
AGGAAAGGTGGGGGTAGGATGAGAAAGCCAGCGAGCCGGCCTAGATTTGCTTTTCAGACCCGGAGTG
CCGATGATATTCTTGATGATGGTTATCGTTGGAGAAAGTACGGACAGAAGGCCGTCAAGAACAACTTAT
ATCCCAGGAGCTATTATCGGTGCACACATCACACATGCAATGTGAAAAAGCAGGTCCAGAGGCTATCT
AAGGACACGAGCATTGTGGTGACAACATATGAAGGAACTCACAATCATCCCTGTGAGAAGTTGATGGA
AACCCTAACTCCTCTTCTGAAGCAAATGCAGTTCCTCTCTAGGTTCTGA
>PmWRKY42
ATGCCTTTGGTCGTCGAGCCGTTAGATCAGCGCTTGGACTTTGACTCCTTGATGGCGGCATTATTGGGC
ACACCGTACTCGCTCGAAGGAGATAGAAATACAAACTCGCTGTGGACGGGCGATACGTGGAGGTATAA
CTTGAGAGCACCGCTACCTGGAACCCATGTGCCGGTCACGAGCGTTATGGCGTGGGAAGGTGCTCCTT
TGATGAAGAAAGTGGGGACAGGGAGAGTACAGAAGAAACAAAGGCATCAAAACCCAAAAAGAGGG
GAACAGATGCTGCTTCTGCTGCTGCTCCTTTTTGATATAGAAATGGAATTCTACAGCCCCAATGCACAA
CCTGGCCTACAGCAACAACAATTCCAAGTGCTTTTCTCTCTTCCAGATCCCATTAAACCCAGAAATGCC
ATGGAGGACTTGCATGAGCTTTACAAGCCCTTCTTCCCCAAATCTCAGCCTCCCCTCTCCTCACAAATC
ACACCACCACCCAATCTCTCTCCTTTGACTTCTCTCACTCCTCTGACTGCACCCAAAGATCAAACACAT
CCCATTCAACAACATCAACACCAAAACCACTACCAGCAACAACAACAACAACAACAACAACAGCAA
CAGTCCAAGCCATCATCTCATTCTGTTTCCTCCACCACCACCCCACGATCCAAAAAAAGAAAGAACCA

GCTTAAGAAAGTGTGCCAAGTACCAGCTGAGAGCCTCTCTGCAGACATATGGGCATGGCGTAAATATG
GCCAAAAACCAATCAAGGGCTCCCCATATCCAAGGGGATATTACAGATGCAGCAGCTCAAAGGGTTGT
ATGGCCCGAAAACAAGTGGAGCGGAACAGATCCGACCCGAATATGTTCATAGTTACCTACACGGCCGA
GCACAACCATCCGGCCCCAACTCACCGCAACTCGCTTGCCGGCTCCACCCGCCAGAAGCCCTTCTCGC
CGCAAACCGTCACGGGCAGTGACTCTACCAAACCCACTTCTCCGGCAACCTCGGCGTCAGTCGACGA
AGACCCTGTTGTACCTCAGAGTACAACCATGGAAAGCTTCAAAGAAGAAAAGGGCAGTCCTATGGTC
GACGACGACGACGACGAGCTCTTTGGGATGTGTGATTCGGTCGTGAGCGACGATTTCTTTGTGGGTTT
GGAGGGTCTCGCCGGAGATTACTTTTCCGATCACTCTCCGACGAGCTTTGGCGTCCCCTGGGTTTCTAG
TAATGCTGCGACCGCCGCTGGCAGTATCTGA
>PmWRKY43
ATGGAGAAGAGGAAGAGCATGGAGTGGGAGCAAGAGACTCTAACCAGTGAGCTAACCCAAGGGAAG
GAGCTGGCAAAGCAGCTTATGAACTGTCTTCACCCTTCTGCATCCCAAGAAAAAAGAGACTTTCTGAT
TTCAAAAATACTATTTTCCTATGAAAAGGCACTCTCACTGCTCAAAAGGGATGTTGGTTCTGATGGAGA
GTACCACATCCCTAACACCATGTTGGAATCACCTACTTCATTTGGCAATGGCAGCCCAATGAGTGAGAT
CTCTGACCAAGATTGCAAGAACAAAAATGTCTTCAAGAAAAGGAAAACAATGCCCAGGTGGACTGAA
GAAGTGAAGGTTTACTCTGGAACAGGGCTAGATGGGAGTCTTGATGATGGCTACAGTTGGAGAAAATA
TGGCCAAAAGGATATCCTTGGAGCTACCTATCCAAGAGGCTACTACAGATGCACACATCGCGGCACGC
AGGGTTGTTTAGCCACCAAGCAAGTTCAAAAGGCAGATGCAGACCCGTCAACCATGGTGGTAACCTA
CAGAGGAGAACATACTTGTAGCCAAGTCCTTCAGTTAGCCAGGTCATCAGTATTATCCCTAGCTAAACA
AGCTTCAACAGGAAATCAAAATGCAACCCGAGAAGTAGAAAAACCAAAAGCATCAGAAGAGATGTCT
TTCAGTTTTGGGGCAGGGCTTAGAGTTAAAACTGAGGATTTGGACACAAGGGAAGATGATATATTTCCA
TCATTTTCCTTCCCTTCTACACCAATTGAACCTGAAAATGTCGGAGACCATATTTTCTGTGCAACTTTGA
TGGAGAATGATTTGATGGATGGCTATTCTCCCACATTTGCATCTCCAGCAGCAACATTTGAACCAGACT
ACTTGCAGGCCACGAGCAGTTTTGGACTTGGCCTTGATTATGTGCAGACTTCAGAATCTGGTCTCAGT
GAGATCATCTCAGCCCCAACTTCAGTGACCAACTCACCAATTGGGGATTTTGGTTTCTCACTTGATGAT
TTGGATTTTCACCATTTTGAAAACCCAGAAAGTTTTGCTTACGAGTCATAA
>PmWRKY44
ATGGAAGGTCATCAAGAGCAATACTATCCACCTCCATCAACATCGTCATCACTACCATTATGGCCACCC
CACCCTTCCTACCTTTTCCCATCCTCCTCAACCTCAATGAACCCTCCTCCTCTTCCAGAGCCTCATCATC
AGCAGCAAGCCCAGCTTCCAGACATTGACTGGATCAGCCTTCTCTCTGGTCATCATGATCATCAGATTA
ATACTAATGAGCCTGCTGCTATGGTGGAGGTTAATAATAAAGAAAATAATGTTATTGAAGCTGCTCAGG
AAGAAAAGGGTAGCAATAAAAGGAAAGGTGGTGAGGGCAGGAAAATTAGTGTGATGAAAAAAGCGA
GCAGGCCTAGGTTTGCCTTCCAGACTCGGAGTGCCGATGATATTCTTGATGATGGCTACCGCTGGAGAA
AATATGGGCAGAAAGCCGTCAAGAACAGCTTGTATCCCAGGAGCTACTATCGATGCACACATCACACA
TGCAGTGTGAAAAAGCAGGTTCAGAGGCTATCAAAGGACACAAGCATCGTCGTTACAACTTATGAAG
GAGTTCACAATCATCCATGTGAGAAGCTGATGGAAACCCTTACTCCTCTTCTCAAGCAAATGCAATTTC
TCTCTAGGTTCTAA
>PmWRKY45
ATGGATGAAACCACCATTTCTTTGATCCTTCAGGCTTGTGAGTTAGCCAGAGACCTCGAATCAAACCTA
CACAACTTGGCCAACCAGCCCAACTTGCTCTCAAACTCCTTGGATGAGATCACAAAGAAATTTGTTAC
AGCCAGAGAGAGAGTGTATGGTCAAGATCCAAGTACTTCTTCAAGTCTGCACAACATGCTTACTCTTG
TGCACCAGCAGCAGATTGGTACAAGTCATGTACAAGAATGGCTGAGGTCTAGTTATGCAACCCAACTT
GTAGCTGATCAGAAGGAAGCTAAGATTGGAGGCTGCATCGATGACGATGATGCAGAGGTTAAGGGCCT
TCAGGCAATGGATATAGTATCAGCTTCTGACACGAACATTGCTTCATCATCCTCACAAAGAGCAAGAAG

AAGGAAGGATCAGGGGTTAATCAGCAAAATTACAGTTCCAGCACCTCGGATTGGAAATACAGAAATCC
CACCAGAGGATGGCTTCACTTGGAGAAAATATGGTCAGAAGGAGATCATGGGCTCCAGGTTCCCAAG
GAGTTACTATAGGTGCACCCACCAAAAGCTCTACAATTGCCCAGCCAAGAAGCAAGTCCAGAGGCTTA
ACAATGATCCCTTAACATTTGAAGTAATGTACAGGGGTGAACATACATGTCACATGTCAGCCACTGCAC
CTTCAATTCCACCACCATCAGCAGAACATCATAATGCTACACAAGAGAGTATGGCCCAAACCCTAGCG
ACTACCACAACCGCTGACCCTCCTACAGCATCTCTATGGCTTTCCATGGACTTTAACCCAATTAGAGGA
GGCGGCGGCAGCAGCAGCAGGATAATAGGTGGCGACCATGGCGGTGGTGATGGTGTAGGCACATCCA
CCACCACGCGTTACGGTAAAGAAGTTGACTTTCCTGTAGTGGATTTGGCTGATGCAATGTTCAATTCAG
GCAGCAGCAGCAGCAATAGTATGGATTTTATTTTCCATTCTGCTGAAAACAAATGGGAGTCAGAGGAC
AAGAAAAACTGA
>PmWRKY46
ATGGGAAGCAACCATAAGAGACTGATCAAGGAGCTAGTTGAAGGGAAGAAGACGGCGGCTGAGCTTC
AGATGCTGCTTCACAAGCCCTTTGGAGATCATGGGTCGGCCTCAGCTGAGGAGCTTGTGGTGAAGATC
ATGACAAGTTTTACAGAGAGTCTCTCTGTTTTGGCTGCTGAGAAGAAGAACCCAGGTGATGGCCATGA
AGATCACCAGTCTGGTGCTTTTGGCGAGGTTTATCAGATCAAACCGGAGCCTTCCCATTCACATTGTGA
CGACCGGAGCTCTGGAGATTCCGGCGAGAGTAGGAAGGTGCAGGGTTCCAAGGATCGGAGAGGTTGC
TACAAGAGAAGAAAGACTTCCCAATCATGGACAACAATCTCTTCTGCAATTGAAGATGGCCGGGCTTG
GAGAAAATATGGCCAAAAGGAAATCCTCAATGCTCCATATCCAAGAGCTTACTTCAGATGCACACGCA
AGTATGATCAGGGCTGCAGAGCAACCAAACAAGTCCAGCAAGTCCAAGACAACCCTCGCCTGTACCA
AACCACATATATTGGCCAGCACACATGCAAAAGCATGGTCCCTCCACAGATGATCATAGGCTCCTCTGA
TCATTGGGAATCTCAGACTGTGAGCTCAGAATCCGAGACCCCAAACAAGCAAAACCATGATTTCCTTG
GCTCATCAGCAATTCCCATTGTAAAACAGGAAGAATACAAGGAAGGGACACCAACACCAAGTGACCT
AACAGACAACCTTTCCTCATTGGAGACTAATCATTTGTGGTCTGATTTCAAGGATGATTTTGCTTTGTGT
GACCCTGCAGCAATGTGTGTGTCTACCAAAATGGGGTCTGATAATGAGGACGTGGTTTCAAACATGTA
CTTGGACATGGATTTCGTAGTCAAGTCTATCGATTTCGATCGCGATTTTAATTTTGATGAAGTTGAATTT
CCTAAGAACTCCTTGTAA
>PmWRKY47
ATGGAGAACTGCAATACGCATGGGGAGAAAATGAGTCTTATAAATGAGCTCGCACAAGGCAGGGAGC
TAGCTAGGCAACTCCAGATCCATCTCAATGTTCCGTCTTCCTCTCATGGAACCCGGGAATCGTTGGTTC
AAAAAATCATAGTTTCATACGAAAAGGCACTTTCCATGCTCAATTCTAGCAGCCCAGCCTCAGGGGGA
GAGCAACAACTGCCCACAGGTCATGCTGCGATTCGAATGATTGAGTCCCCCCCACCTTCTCTCAATGG
AAGTCCCCGGAGTGAAGACTCGGATCGCGAATTCAAGGACCAGGACAACAAAGATTCGTCCAGGAAG
AGGAAAACTCTGCCAAGGTGGACACAACAAATAAGAATTACATCAGGTATAGGGCTTGAGGGGCCTC
TTGATGATGGCTTCAGCTGGAGGAAATATGGCCAAAAGGACATACTTGGAGCCAAATATCCAAGAGGC
TATTACAGATGCACTCATCGGAATGCTCAATGCTGTTCGGCCACAAAGCAAGTGCAACGCTCCGATGA
GGACCCAACAGTCTTCGAAATTACTTACAGAGGAAGGCACACATGTACACAAGCCTCCACCAGCATAA
CCAGCGCTCCTCCTCCTCCTCCTCCTCCTCCTCCACAGAATAGCATGGACATAGTGGACCCTCAACAGA
ATGAGCAGCAACCACAAGACTTGCTCTTAGCCATCCCACAAGGCCTTACAGTAGTAACTGAAGGCTTA
GACGCTGGTGATTTTTCTCCTCCATTTATGGGCCCTGCAACTTCTGGAACACAGTATTTCTCAGCTTCA
GAGCAGGGCTTTGGAGGAGGCAGCCAAGGTTTTCAGGGTGCTGAGTGTGAGATTGCAGAGATTCTTT
CATCTTCTACTTCAGCAGCCAACTCTCCTGCTGCTTTGGGTTTCCCATTTGGTCAGGCTGATCAAAACT
TATGCCCCAACTTCTCATTTGACGGTCCAGGCTTCTTTTCCTCATAG
>PmWRKY48
ATGCCAATCAACTCCCCCCGGCCAAGAAAAAGGAAGTGTCAGCAGAAAAGACAGGTTTGTCAAGTAA

GCGCAGAGAATCTCTCCGCCGATTTATGGGCATGGCGTAAGTACGGCCAAAAACCTATCAAGGGTTCT
CCTCATCCAAGGAACTATTACCGCTGCAGTAGCTCCAAAGGATGTTCAGCGAGAAAACAAGTGGAGC
GGAGCACGGCGGACCCCAACATCTTCGTCGTCACATACACAGGCGACCACAATCACCCTCGTCCAAC
GCATCGAAACTCCCTTGCTGGCAGCACCAGAAACAAGTTATCAGCGACGGCGGGTCAAAACCAGCCC
ATCAACAATGACCCGGGTTCACCACCAGCCCAAGCTGACCAAGACGCTGTTGCCAATAACGTCAATTC
TGACAATTCACTTGACAAAGAAAATGAGGAACTTGATGAGGAGGAGGAAAGGGATGAGAACGAGAT
AGAGGACGAGGACGTCGAGGAGGACGATGTTCTGATCCCGAACACAGCCATGCCGGATGAGATATTC
TTGGGGCTGAAGCAATTGGGTTGTACTAGTTCTAGTGGTAGCGGGGGTTCTCTGGCGGCAGGTGCTTC
TGGTGATACTTTTTCGGATCAAGAGCCGTCAAGTTTGGGGTCTTCTTGGGCCGCCGGAGCCACCGTTG
GCGGAGGCTGCTAG
>PmWRKY49
ATGAAACCCTCTCTCCAGAACCAGACTCAGAAGTTCCAAAAGAACTCAGGCCAGAAGGTCAGGCATC
CAAAAGAAGGAAGGTGGTTCATGAGAAGACTGTGGAGGAAATATGGGCAAAAACCTATCAAAGGATC
ACCTTATCCAAGAGGCTACTACAGGTGCAGTACATCAAAGGGTTGTTCTGCCAAAAAACAAGTGGAG
AGAAGCAAAACTGATGCTTCAGTGCTCATAATCACCTACACTTGTAGCCATAATCATCCAGGCCCTGAT
GTCTCCACCACTAACTTGACCCAATTGCAACCCAGCACTGAAGATCATAATCTCCCAGCCCTACTTCCC
AAACAGGAAGAGCAAGAGAAAGAAGAGCAAGAGAAAGAGCCAGAGAAAGAGAAAGAAGAAAAAC
AAGGAAAACTAGAAGACCATCCTGCAATGAAAAGTGATCATGAAGATCATTTTCACTACATTCAGTCC
CCAATTAGGTCTTCCCAAAATATTATGATTGAACAAGAAGAAGACCCTTTCCTAGAGAAAACCCATGTC
TCTAGCACTCTTGGGTTCCTCTTGGATGAAGAGCCCCTCTCTTATTCCCAGCTCATGAGCTTCTCAACA
CCCAAATCTGAAGAAAATGACTTCTTTGATGAGCTTGAAGAACTACCCACATTTTCATCTTTCCCAAGC
TTCATGAGGAGCAATCTCTCCCTTGAAAGGATTCCCTCTGTCCCCTCTTGA
>PmWRKY50
ATGGAGGACGTCGAAGAAGCTAACAAAGCAGCTGTTGAAAGCTGCCATAGAGTTCTAAGTCTTTTTTC
TCAGACCCAAGATCAAGTTCAGTATAGGAATTTGATGGTGGAAACTGGGAAGGCCGTGTCTAAGTTCA
AGAAAGTTGTTTCCCTTCTCAATACTGGTTTAGGTCATGCTAGAGTTAGAAAGCGTAAGAAGCTTCAAA
TTCCTTTTCCTGAAAGAATCCTCTTAGATAATCCCAATTGTATTACAGACTATCCATCCAAAACTCCTCA
CTTTATTCAGTCTAGCTTTCCTGAAAACCCAGTTCAAGATTTGGGTTTAAATGTTAAAAATTCCTTGTGT
TTGGGAAACCCATCATTGGAATTAAGCACTAATGGGAAAAATCCGCTTCAACCGGCGCAACAAGCACC
TCCAACACAGTATCATTTCCTTCAGCAACAACAGCAGCAACAGCAAGTGCAACAACAGCAACAGCAA
CAGCAGAGGTTGTTCCTTCAGCAGCAGCAGCAGATGAAACACCAAGCAGAAATGATGTACCGTAGGA
GCAATAGTGGCATAAACCTGAATTTTGATAGCTCTAGCTGCACACCTACGATGTCATCTACAAGATCCTT
CATTTCTTCCTTGAGCATAGATGGAAGTGTGGCTAACTTTGATGGTAATTCCTTCCATTTGATTGGGGCT
CCTCTCTCTTCGGATCAGAATTCACAACATAAGAGGAAGTGTTCTGCAAGGGGAGACGATGGGAGTGT
GAAATGTGGTAGCAGTGGTAGATGTCACTGCTCAAAGAAGAGGAAACATAGAGTGAAAAGATCTATTA
AGGTGCCTGCTATTAGTAACAAGCTTGCAGATATCCCTCCAGATGATTATTCATGGAGGAAGTATGGAC
AGAAGCCAATCAAGGGTTCTCCTCACCCTAGGGGATACTATAAATGTAGTAGCATGAGAGGTTGTCCTG
CAAGGAAGCATGTTGAAAGGTGCTTGGAAGAACCCTCTATGCTTATTGTTACCTATGAAGGTGAGCATA
ACCACCCAAGGATACCATCACAATCTACAACGACATGA
>PmWRKY51
ATGGATGCCTCCTCTGGTTGGATCAAGCTCAACTTCGATGGATTAGTAAAAAATGGAGTTGCTACCACA
GGATTTGTAATTAGAGATGACAACGCCCACACCCTTCTCGCTGGTGCTAAAACAATTGGAGACAATTC
CATTGCTGTGGCTGAATGCCTTGCTCTGAGGGATGGTCTAGCCCATGCAGTTCATCATGATTGGCACAA
TATCATAATTGAAGGTGACTCCAAATTGGTCATTGATGCTATTAACAAGAAATGCTCTGCCCCTGGAGTA

TTATGCAGCTTCTCCAAGATGTGTCACACTTGGCCTCCTTTTCAAGCGATGGAGAGCCATGAGCATCTA
AATAACAACTTTGGGGCAGTCAACTCTTCGGGGACTATAATGGAAAACTGGAAGAGGCTATGTGCCAT
GCTGCAGAACTCCAACGAACCGCCTGTCGTCGTCGGTGATCTTCAGCGCATTATGCAGGAAGAATCAA
TTGAAAGCAAACAGGGAGCACCGCGTCTCAAAAAAAACTTTTGCCAAGTTTATTCCTCAACAAAGAT
GACAGGAGCCGCAGAATCCAAATTGGTACCAATATCAGAAGATGGTTACAGTTGGAGAAAATATGGAC
AAAAAGGAGAGAAAGGTAATGAAGGCTCGACGAGTTATTACATGTGCACATATCCCTATTGCAAAAGA
AAGAAAAAGGTGGGCAGATCACTGGATGGACAGATTACTCAAGTAGCATACAAGGGAACTCACAACC
ATGAGGAAAGGAAGGACATGAATCAAAGTCTTTCTAGGAGTGTGTGGGAGTTGCGCGGCGGGGCGCC
TTTGCTTAGCATTACTCCAGATGTCCTACCTAGCATCGGCTCCAGTGTGGACAACGTCGACCATGAAGA
GCCAATATTGCATGAGCAGAAACATGATCATGATCAACCAAGCCACGAGAACACTAACTATGGCAAGC
CAAGGCAGGCGACAAAGACTACTTCAGGGACAGAATGGTTCTTTTTACTCACAAGCCTCGGCTTGGA
GATCTTATCAGCTGCTTTTGATCAGGCTTCCTCCCCAAGTAAACCACACTACGCACTATTCGGTATGCTG
TTGGCTTTTGGGGCTCTACTCACTTGCATATGTGAGCTCCTTCACAAGGGTATAAAAGAAGGAGTCGTG
TTGAGGAGGTGGGGAATGCTATGGTGGTTTTATTATCCACCTCCTCGTTACACAGTTTTTGGTACCCTCC
CTGACATCTATGGCTTAGCTGGTGGCATCTCTCAGTGCGTTTGCTCGACAATTCAATATATTTACTTCTG
TCGCCGTGTCAATAATCCTATCAAAATATCCCTTTGGCCTACCATCTTTCTTATATGCTTGGCTGCTTCAA
GATTGAATGGGAATCGAAATGAGACCCTTGTACATTATGATTAG

>PmWRKY52

ATGAACCCAGGATTGGATTTCAAAATTCATGAGGCTGCTCAAAGTAGTTTGAAACATGCCCATCACCTG
TTTGGTTGTGTATCTGAGAATAAACAAAAGAGAAGTGTTCAAGAAGTAAGCTTGATTGCTCAAGATGC
AGTGGAAGAATTCAGAAAACTACTTGCTCTCCTTGATGGATCATTGTTATCAAACCAGAAGAGGATCA
GAAGAGGTCCTTTGCCAAAATCCCATGACATAAACCAGGTTGAACTGTTGGACTCTCCTAACCCTTCAT
CCCAAAATACTACACACAATTGGCCTCAGCCTCATACCCTGCCCCAGCCTCGGAATTGCTTGGTTAGAC
AGTTTTTTAGTGCTGTTCATCAAAGTGATCAAGCAACTGCAAATGTGATCCACAGTAATAGCTTTAGCA
TCGCTAGAGAGAAAAAACCAAACCCGGCATTGCAGCAAGGGCAATCTGAAGCCGGTGTGGTTCTTCC
TAATAATTTTATCATGGGGTTGAACCAATTCTCACAAAAGCCTACTAGCACTTCTTTGATCAGTATGGAT
GGAAGTAGCCCTAACACGCGGATGATTCACTATTCGTCGTCTGAACTCTTGGGTTCTCGAGATTGTACC
TCTATGTTTTCTTCCAAAAGGAAGTGTGGGGTGAAAAGTGAAGAAGCCACTCCAAGATGCCTAGTCTC
TGCTGGTGGATGCCATTGCTCAAAGCGAAGGAAACTTAGAATAAAGAGAAGAATCCGAGTTCCTGCTT
TAAGCAATAAGCTAGCTGATATACCTCCTGATGACTATACCTGGAGGAAGTATGGGCAGAAACCTATTA
AAGGGTCTCCATATCCTAGGAGTTATTACAAGTGCAGCAGTGTGAGGGGATGCCCTGCAAGAAAGCAT
GTCGAACGGTGCTTGGAAGATCCTACCATGTTGGTTGTTACATATGAAGGCGACCATCAACACCCCAA
GATTACATTTCAAGCACCTACAGTTGCGATTTAA

>PmWRKY53

ATGGAGAACTCTCAAATACCATATTGCTTTGCTCCGTTAAGCATTGAAGAAGAAGATGACTTTTTCCAT
GCTTTGCCTCAGCTGTTTGAAGCCACAGCAGCTTTAGATGAATTGGAGGAGCTTTACAAGCCATTCTAT
CCAGTTTTGCAACCCTCAACCCCACCAATCATTGTTGCCTCCTCCATGTCTGTTCCCCAAGAGGCCATC
ACTGTAGAAGTACCAAAGAAGCTGAAGGAATCATCAACTATCTGTAAAAAAATTAGGAAGAATCAGA
GCAAAAGGGTGGTGAAAGAGGTGAGAGCAGAAGAGCTTTTTTCAGATGTGTGGGCGTGGCGTAAATA
TGGGCAAAAACCCATCAAGGGATCGCCATATCCGAGGAGCTATTACAGGTGCAGCAGCTCAAAAGGAT
GCTTAGCAAGAAAACAAGTGGAAAGGAGCTGTTCCAATCCTGAAACCTTCATCATAACCTACACTGCA
GAACACAACCATGTCCATCCGACTCGTCGAAACTCTCTTGCCGGAAGCACTAGATCCAAGTTCCCATC
ATCTAAAAACAAAGGCAAGTGTAAAAGTTCTTTGAATTCTCCAGCCATGACCAATTTGGCGGCTTCCAT
TGAAGAAGATCCCGATGTGCAAAGTGCAAGTACTAATGCTGTAAAAGAGGAGGCACAATTGTTGTTGG

AAGAGGATGAAATTAGTCATGAAAATGTCATGTTGGATGTGATGTTGAGTGATGAAATGATCCCAAGCT
TAGAGAATTTGGATAGAGAGTTGGAACCAGTTATGGATGGTTGGTTTTTGGATCAATTTTCGGATAATTT
TCCAAGTCCTTGGTTCAATATTGGTCACTCTAATACTGTAACCGGTGCTTGCTGA

>PmWRKY54

ATGGATTCTAGTAAGAGCTGGGAGCACAAGTCACTGCTAACCGAGATCACTGAAGGGATGGAGCTAG
CAAAACAGTTGAGGCTAAGTCTAAGTGCAGCATCTTCATCAGACACCAGACAATTTTTAGTGCAGAGG
ATATTGTCCTCCTATGAGAAAGCCCTTTTGATACTGAACTTCAGTGGGCCGGCTCAAAGTACTGCGGGT
GCAATAGCCAGTGTGCCGGAGTCTCTTGTGTCAGCTAACGCAGGTCCTTGTTTTGATGACTATAACAAG
AGTCCCAAGGATCATCAGGACCTCACAGATGTCTCCAAGAAGAGAAAGATCATGGCCAAATGGACAG
ACCATGTAATGAGAGTTAGCTCTGAGAATGGGATTGAAGGACCTCATGAAGATGGCCATAGCTGGAGA
AAATATGGACAGAAAGACATCCTAGGAACCAAACATCCAAGAAGCTATTACAGATGCACCTACCGAAA
CACGCAAAGCTGTTATGCTACAAAGCAAGTGCAAAGATCAGATGAAGACCCCACGATATTCGAAATCA
CATACAAAGGAAAGCATACATGTTCTCATGGTAGCAATTCAGTTCTACCACCACCATCACCAGAACAG
CAAGAACAAAAACGAAACAAGCAAAATAATACTTCTCACCAACAGCAGTCTCAAGGAAACCAAATGA
GCTTTCCGACTAATCTGAGAGTCGATACCAAGTTCTTGGAAGACAGAGAGAACATGACATCTCCATTC
TCTTTCACTTCAACTTCATTTGGATGTATGATGGCTGATGATGCCTTTCTATCTTCAATGCTTGATGATAA
CAACACTTTCTTTGACAACTTCAATCATTCATTGCTATCTCCAGCCGCAGGCGAATCAAACTACTATTTG
ATGCCACCAAGCCAGATGAGAAACATTGCAGGAAACGAGCAACTTTCAGAATGTGGTCTTACTGAGAT
CATCTCAGCCAACAATTCGTCAACCAATTCTCCAATTCCAGACATGGATTTTCCACTGGAGCCAGTGGA
AATTGAGCCCAATTTCCCATTTGACACCACAGGAATTTTCTCATAA

>PmWRKY55

ATGGAGCACAACCAAATACTTTTCCTGGCTTTACCAAAATCATCTGATTCAGCTTCTCCTAATCTTCCAT
CAAACCCATCAAATATTTCTCAAGTGTTTCCTCGTTTTCACTTTGACAGCGCAGGTCTAATGAAGAATG
AAGCAAAAATTTATCAGCATTGTGGAACCTCTTATGGTTCAGTGGACAAGATGAAATCCGGCAAGAAA
GAAGGTGGCAAAGAGACCAAGAAGCACAAATATGCATTTCAAACAAGGAGCCAGGTTGATATACTTG
ATGATGGGTACCGATGGCGAAAATACGGGCAAAAAACAGTTAAAAACAGTAAATTTCCTAGAAGCTAC
TATAGGTGCACGCATCAAGGGTGCGTTGTCAAAAAGCAAGTCCAACGCCTTTCGAAAGATGAAGAGA
TTGTTGTGACGACATATGAAGGAATCCATTCACATCCCACTGATGAAACTTCCGCAGAAAACTTTGACC
AGATACTGAGACATTTACAAACATATAATGCCTAA

>PmWRKY56

ATGGAGGCTGCTTTGGGAAAGTCCAATTCTCATGAAGGATCAGAAAAGGAAGAGAAGAGACTGGTAC
TTGAATCTAGCAGTCTTGCAGATCATGATGATCAAGAAGCAGCTGGTAGCGAAGAAGATACTCTTCTC
AAAGTTGGAAATGGAGGATCATGCCAGGAAAATAACGAAATGAAATCATCCTCACCTACACAGAAAGA
TTTGAGTAGCAGCAAGCAGATATCAACGACGAGTAATATGAAGGTGGAACCTGATCATTCAATGGCGT
CTACTTCATCAAGTCGAAAGGAACAGGATTATCAGCTTGAATCTGCTAGAGCTGAAATGGGTGAGGTT
AGAGAAGAGAATCAAAGGCTGAAGAAGTACTTAGATCGGATAACAGACGAGTACCAGACACTTCAAA
TGCAGTTGTATGACATTCAAGAAAAAGCAAACAAGTCCAAGGATGTTACAATTGCTAGTACTAACAAT
ATTTATAACCATCAATACGAGGAATCCGAGATGGTCTCACTCAGCCTTGGATCGTTTTCTAGCAGAGCC
AAAAAGGATGAAAAGAACAAAAACCGTAGTACCAGTAGTCAAGGGAAAGAAAACGAGTCAGATAGA
GAAAGCTTGTCTTTGGGGCTAGACTGCAAATATGAAGCACCTAAATCAAGTGCTGCAACTACTGAAGT
GCCTTTATCGAATTCAAGCCCTGCAAGCAGCCTTGAAGAGGTGCCCAAGGAAGAAGCAGGTGAGACC
TGGCCACCTAAGAAAGTTCTCAAGACAGCGAGAAGTGTAGAAGATGAAGTTGCCCAACAGAACCCAG
TGAAGAAAGCTAGGGTTTCAGTGAGAGCCAGATGTGATACCCCAACGATGAATGATGGATGTCAGTGG
AGGAAATATGGACAGAAGATTGCAAAAGGAAATCCATGCCCTCGTGCATACTACCGTTGCACCATCGC

ACCGTCATGTCCTGTACGGAAACAGGTACAAAGATGTGCGGAGGACATGTCCATCTTAATCACCACATA
TGAAGGAACGCACAATCACCCACTTCCTATGTCAGCCACAGCTATGGCTTCCACCACCTCTGCAGCCG
CTTCCATGCTATTATCCGGCTCATCATCATCAGCCCGGTCTGGCCTGAACCCATCAGTTGGGGCCACCA
CAACTGCTGCTCACGATCTTCATGGATACAATTTCTATCTCTCTGACAACTCAAAATCCAGATTCTTCAT
ACCCAACTCTTCATTGTCATCTTCTCTTCCAACAATCACCTTAGACCTAACTTCAAACCCACCACCACC
ATCATCATCCTCCAATCCCTTGTCCCATTTCAATAAATTTTCTTCTTCATCAAATTCTTCTCACCAACTAC
ACCCTCCCACCAGCCTCAACTTTGGTTCTAATTCTGAATCCAACACCAACATGTCATGGAGCAATGGGT
TCCTTTCCTATGGGGCTCAATTACCACCATACAATAGCAACAACAAGAACCAAATTGGATCACTAAGTA
GTCTTGGAATTAGGCAACAGCAGCAGCCTCTACAAAACAATATATACCAAAACTACATGCACAAGAAT
AATCTTAACCCAACTCCTCCTCCTCATCCTTCTCAAGGAGCAACTCCTCATCAGTTTCAACCAGACCCC
ATTGCTGCTGCAACCAAGGCAATCACAGCTGACCCAAGTTTCCAATCTGCCTTAGCAGCTGCACTTTC
TTCAATCATTGGCAGCAACAGTAATGTTGGGACAGCTGGCACTACTGGAATGCTAGGCAACAACAACA
ATCAGGCTGGTGGTGGTGACAATAACAATATGGCTCAGAAATTCAACTGGGGCGACCAATTTGCAGGA
AGTACAACTACTTCTTCTTCTCCATACCTCCAAACGCAGATAGGGAACAATAGTACCATTGGATGTGCA
TCAAGCTATTTGAACAAAACGACTTCTGCGAATTCGCAGCCCGGGAGCTTGATGTTTCTGCCACCTTCA
TTGCCATTTGCAAGTGCCCCCAAGAGCGCATCTACGTCTCCTGGTGACACTAGAGATCACAAAAGTTT
ATAG
>PmWRKY57
ATGGCTGAGCACGAGAGCTTCGATGCTCTCCAAGTAGAAAAGCTAAAGCCAAACGAACAAGAACAAG
AAGAAGATGAAGAAGGCGATGAAGATGAAGACGAGGACGAAGAAGGAAAGCGACTCGGTGAAATTC
ATCTGGGCGAGTTACAGAACTCTGCACCAGAGTCAAGAGAGACGCAATTAGAAACCCTAGCGGTGCC
TTCTACGCTGGAATTATCAGAGAACGATCAGTGTGCAGGTTTTCAGGTTAACTCCACGTCACAGTCGAT
TGATGGAGCTCAATTACAGGAGCAACTTGGGGTGAGCCACCAGGAAATTTTGGCAATTGTAACACACC
AGGATTCTCAGATGCAGACACAAAGTCCGGTTCAGTTGGCTGTTTATCCAACTCCATTGTCAGAACTAT
CACCAACGTCTGTTACACAATCCATTCAGACTCAGACACAAAGTCCGCTTCAGTTGACTGTTTATCCAA
CTCCGTTGTCAGAACTATCACCAACTTCTGTTACGCAATCCATTTCATCTGCTCCCAGCCCGATTCTGCT
AGAACAAAAACTGCCACCAGAAAAGGTTAATACTTTATGTACACCAGAGGTGGACAAGCAAAATTCT
TCTGACCATAAATTTATATCTTCTGTTCCTCTTGTGAAGACTTCTGCTTCTGATGGTTACAACTGGAGAA
AATATGGTCAGAAGCAAGTGAAGAGCCCTCAAGGTTCTCGAAGTTATTATAGGTGCACATATTCTGAGT
GTTATGCCAAGAAGATTGAATGCTGTGACCACTCGGGCTATGTAACAGAAATTGTTTACAAAAGCCAA
CATACTCATGAGCCCCCTCAAAAAAGTAACTGCACAAAGGAAAGTAAACTTGCATTATCTGCTGAGTG
TGTAAGGAATAGTGTTACAGAACATCCCTGCAGAACATTTAATGATTCAGAGGTGTCCACATCCTCAAA
AGAACGTATTCAAGAAACACCTTCAATTCCTGAAAGAAAGCGGCAAAGCCCAAGTGACTCTGATGGT
AATGGTGATGTTAAGATCAAGGAGGAGCATGGTGATGGTGATGAACCAGAACCCAAACGAAGGCAAG
TTTACTCAGTGAAGAAAAGCAACTTAGAGTATTCAACTTCTCTCCTAAAACCTGGCAAGAAACCTAAA
TTTGTTGTGCATGCGGCGGGGGATGTTGGAATATCAGGTGATGGATACAGATGGCGCAAATACGGACA
GAAGATGGTGAAAGGAAATCCGCATCCCAGGAACTACTACAGATGCACTTCTGCTGGGTGTCCAGTCC
GGAAGCACATTGAAACAGCCATAGATAACACAAGTGCTGTGATTATAACATACAAGGGGATACATGAC
CATGACATGCCTGTTCCAAAGAAACGACATGGCCCTCCGAGTGCTCCTCTTGTTGCTGCCGCTGCTCCT
GCTTCCATGAACAATTTGCATATCAAGAAGACTGATACACACCAAAACCAGATTTCTTCAACCCAGTG
GTCAGTGGACACCGGGGGAGAATTGACTGGTGAGGCCTTGGACCTTGGAGGTGAGAAGGCAATGGAA
TCAGCTCGAACTCTTTTGAGCATTGGATTTGAAATCAAGCCTTGCTGA
>PmWRKY58
ATGACTTCCTCTTTCACTCACCTCCTCACAAGTAACATGAACAACATGGACACCAATATTGACCAGGAA

AGAACTAACTGGGGACTTTCAGACTATTCTTCAGACCGTTTAATGGACAGAAATGGCATTGAAATCCC
AAAATTCAAGTCACTTCAGCCCCCTTCTCTGCCTATGTCTCCTCCTCCTGTTTCCCCTTCTTCGTACTTA
GCATCAACACCAGCTTTTAGCCCAACTGATTTTTTCAGCTCACCCATGTTCCTATCTTCCTCTAATACTC
TTCAATCTCCAACAAGTGGAGCCTTCTCTAGTCAAATCTTTGACTGGATGAGTAATTCTAAAGAAACCC
AGCAAGGAATGGAGAGGGAACAGAAAATGTTCTCTGAATTCTCTTTCCAACCAGAAACAAGGCCTGC
TGCAACATCATCCTCATCCTTTAACATTCAAGCTTCTTCAATCATGGCTTCAGTGGAAGAATCGTTGAA
AATGGAACAGAAACCATGGGATTTCAACAGAATCTCAAGGCAAGCTGATTCTTCAACAGAGAAGACA
GGAGTTAAGTCTGAATTTGAGCCACTACAGATCATCTTGCCAGAGATTGGCACAAACCAAACAAATAT
GCAGACCAATGGACCTAGTGGGGCTCCTAGGCCTGATTCAATACATTGCACTCAATCGTCTCAGTTTGT
CAGAGAACAAAAATCAGATGATGGGTTCAATTGGAGAAAATATGGGCAGAAACAAGTGAAGGGCAGC
GAAAATCCGCGGAGTTATTACAAGTGCACATATCCGAATTGCCCAACAAAAAAAAAGGTTGAGAGATC
ATTGGATGGACATATCACTCAAATTGTATACAAGGGAAGTCACAACCATCCCAAGCCTCAGTCTACAAG
AAGATCAACCTCTCAGTCAATTCAGGGTTCTTCATATGGCATTTCTGATCAATCTGTGCCAACAATATCC
AATCCGAAAATGGAATCTGTCACGATGCAGGAGGACTCTTCAGCCTCAATTGGAGAGGATGAGTTTGA
ACAAAACTCACCACTAAGTAATTCAGGAGGAGCTGAAGATGAAAATGAGCCTGAGGCCAAAAGATGG
AAGGGAGAAAATGCGAACGATCAGCCCTTTTCAGCTGCTGGGAGTAGAATTGTAAAAGAGCCAAGAA
TTGTAGTGCAGACAACAAGCGAAATAGATATTCTGGATGATGGGTACAGATGGAGGAAATATGGACAG
AAAGTAGTGAAGGGAAATCCAAATCCAAGGAGCTACTACAAATGCACATCGGTAGGTTGTCCGGTGA
GGAAACATGTGGAGCGAGCTTCACATGATACAAGGGCTGTGATCACCACATACGAAGGGAAACACAA
CCATGATGTTCCTGCAGCGCGCGGCAGCGGCAGTTATAGCAATGGAAATAGACCTGCTTCTGATAATGG
CAGCAACAACAACAGCAATGTGTCCATGGCTGTGAGGCCCTTAGCCTTGCCTAATCATTCGAACTTGA
GCTACCTCAGCTCTCTTCAGAATACAAGGCAGCCAACAACAGAAAGGCAATCACCCTATACCCTCAAA
ATGTTGCAAAGTGAAGGAAGTTATGGATTTCCGGAGTTCTAA

Supplementary Data 2. The genomic sequences of 58 *P.mume WRKY* genes
>PmWRKY01
ATGGCCTCCTCTTCTGGGAGCTTAGACACCTCTGCAAATTCACACCCAACCTTCACTTTCTCGACGCAC
CCTTTCATGACCACCTCCTTCTCTGACCTCTTAGCCTCTGGCACAGATGAAGACCCCAGCACCAACAC
CACAGTTCAACATGTTGGCCATGGCGGGCTAGCAGATCGAATAGCAGAACGTACTGGGTCTGGTGTGC
CCAAATTCAAGTCTCTTCCTCCTCCTTCGCTGCCCATTTCTCCTCCCTCCGTTTCTCCCTCTTCTTACTTC
GCAATCCCAGCTGGGTTGAGCCCAGCTGAGCTTCTCGACTCTCCTGTCCTCCTAAGCACTTCAAACGT
ATGTATATGTTATATGTGTATTTTGAATCTGTTTTCTTGGGTTTTGCAACATGCTCTGTTTGGGTTTCGAG
AAAATTGGGATAAAGGAAAAGAAAAGTTAAAAAATAAGAAAATATTTTTCATTTCCCTTGAGACCAAA
TGATTGGACTTTGCTAACAAGGTTTTAACTTTGAGTCAAAGTCTTCTGGTTTTTTTAACTGCTCTCTTTG
AGTTTGATAATTTTGCTGCTTAATTGTTGGGTTTTGTTTTGATGTTTTTACTAATGGGTATTTCTTATTTTG
TGTTATTGTGGGCAGATTCTTCCATCTCCAACTACCGGAAGTTTTGCAGCTCAGGCCTTTTGGAAGGCC
AATTCTGGAAACAACCAGCAGATTGTTAAACACGAAAGCAAGAACTACTCAGATTTCTCTTTTCAAAC
ACAAACAAGACCCTTCACTTCATCATCAACAATGTTTCAATCTTCTAACGGCACAATTCAAACTGTATG
TGGTGTTTTATTTTTTCAATTTTCACTTGTTTATTTTTCTTTGTTAGATATTCTGAATTGTTTACTAATTTGT
TGCTTTTCTTTCAGGCACAAAAACAGGGATGGAGTAACAGTTACTTTCAGGCACAAGAACCCCAAAA
GCAAGATGATTTTTCCTCAGGGAAGAGTATGGTGAAACCTGAATATGGTTCTGTGCAGAGCTTCTCATC
TGGAATGGCCACCAATATCCAAAATAATAGTCAGGCCAATGGTGGTTTCCAATCTGAGTATAGCAACTA
CAACCACCAAACATCTCAGACCTTGAGCAGGAAGTCAGATGATGGGTTCAATTGGAGAAAATATGGTC
AAAAACAAGTGAAGGGAAGTGAAAATCCAAGAAGCTATTACAAGTGTACTTACCCCAATTGCCCAACT

AAGAAGAAAGTAGAGAGGTCTTTGGATGGGCAAATCACTGAGATAGTTTACAAGGGCAACCATAACC
ATCCCAAGCCACAGAATACTAGAAGATCATCATCAAATTCTTCGCATGCAATTCAGGCTTCTAATCCCA
ACACCAATGAAATCCCAGATCAGTCTTTTGCCAATCATGGTAATTCACAAATGGATTCCATTGGAACTC
CAGAAAATTCATCCATTTCAATGGGAGATGATGATTTTGAACAGAGTTCTCAGAAAAGCAAGTCAGGA
GGAGGGGATGAATTTGATGAAGATGAACCTAATGCCAAAAGATGGTAAGTTTGATTTATTAATGCATGA
ACAATATTTTGTGTTTGATGAGTTAAGTTGAAGTATTTCCATTGAGTTTTTTGGGTTTTTGTGTGTGATTA
ATTTGTGATTTTCCATGATTATGCAGGAAAAAGGATGTTGATAATGAAGGTATTTCAGCTCCTGGGAGC
AGAACAGTGAGAGAGCCTAGAGTTGTAGTTCAAACAACCAGTGATATTGATATTCTAGATGATGGGTA
CAGATGGAGGAAGTACGGGCAGAAGGTGGTAAAGGGCAATCCAAATCCAAGGTAAAGATCCTCTTGG
TCATGATTTTGATATATACTTTTTCAAACTTTTGAAAGATGGATTGCTTTGATAATTCAATATTATTATTGC
CATTGTTCAGTCTCTACCTAATTGGACACTTTTCCCGGAAAACATATCACTAAATTAAGTCTCATTTTGT
AAAGAAGATATTAAAAAAGTGCCACGCAAAGTGGATTCTTTATATGTACCTTCTTCTAGATAACATCTTA
GTTTCTTTTTAAAAAGACACTTTCTTCTAAAGTTTTCTTTTGATGGGTGTGAATGCTGTGAATTTTTACT
TCTGCATTTGGGAAAAAGTGGACTTCCTTTAAGAAACTAGATTCTGTAACTTGTTTGCTAATTGGGGTG
TGTTTTATTTTTGAAATTGCAGGAGCTACTACAAGTGCACAAATCCAGGATGTCCAGTGAGAAAGCAT
GTTGAGAGAGCTTCTCATGATCTTAGAGCTGTGATCACAACCTATGAAGGAAAGCACAACCATGATGT
TCCGGCAGCTCGTGGCAGCGGCAGCCATGCTTCTGTCAATAGGGCTCTACCAAACAACAACAACATTA
ACAACAGCAATAACAATGTAGCCACAGCAATGAGGCCTGTGGCCCATCAAACTAACAACCTAAGGCA
ACAAACATCAGAAGGGCAACAAGCACCCTTTACCCTAGAGATGTTGCAGAGCCCCGGGAGTTTCGGA
TTTGCAGGGTTTGATAACTCTATGGGGTCATACATGAACCAAGCGCAGCTCAATGAGAACATGTTTTCT
AAAACCAAGGAAGAGCCAAGAGATGATGCCTTTTTTGAATCATTGCTATGCTGA
>PmWRKY02
CTACATCTGTGGTCCAAGAGGAAGCCTACTCATTAGCTGTTGGTATACAGATGAACCATTGGACATGTT
TAGACCAGATTCTGACATAGGCTCCACTTTTGGTTCTCCTTTTGGTAACATGAAGCCCATTTCATTGACC
TGGCGCTGTTGTGCAAAGTACGGATGATGAACAGGCATAACAGGGAGTTTGGGTTGGCCTGGACCCA
ACCCAGCCATTGCCAGATTGGCCAGGCCGGGTTGGTTCATTCCGAAAGAGAAGCCATGGGATGGCCCC
AGCTGCTGCCTTCCAGGTAGGCTGAATGAACCCAGTGATGAAGGCCTTTCAAATCTTGCCATGCTGTT
GTGAACTTGTGATGGCTCCGGTCTATGCGGATGGGTTTGAATGCCAGCAGAGGAAGCTTGGCCAGACA
TGGTGTTGGACGGGCCAGAGTTGACATGGCTGCTATTTCGAGCAGCGGGAACATCATGATTGTGCTTT
CCCTCATATGTGGTGATCACTGACTTAAGGTCATGGGATGCTCTTTCCACATGCTTCCTCACTGTGCAGC
CAGCATTGGTGCACTTGTAGTAACTCCTGCAGAAAAAAAGAATTTTGTTTCATCCTCATACAAATTAAG
AAAGAACAAATCAAGAAAGAAAAAAATTAATCGTCATAATGAGTAATAACCTTGGATTTGGATTGCCTT
TCACAACCTTCTGCCCATACTTGCGCCAACGATATCCATCATCAAGGATATCTACTTCACTGGTTGTCTG
GACAACGACTCTAGGCTCACGAATGGCTCTGGTAGCTCCACTCATTTCTGTTGCATAGGCTTCAATTTT
CCTGAAACATCATAAGTGTAAAGACACTTTTATGGTGACCCCAATGTCTAATACTTGAGATTGTTCACC
AAAAGATAGAAAGAGGTGAGCAGTAACCTTCTTTTCGACTCTGATTCATCTCCTTCACCATCATAAGCC
AATGAAACACTGCCATGTGTCCCTCGATCATCTTCATCTTCATCATTAGAAAAGGTAGATGACGCGTCC
ACCACATCACCTGATTCAAGGTGCGTACCATTCTGAGCCTGCATAGAAGAGGATTGGTTGCAGTAGTC
AGGGCCCACAGATGCTGATGAAGTCACCTCAAGGTTTTCATGCTTCCAATCAGGAGCTCCAACATTTG
CCTTTTGTGTACTTGCCCAAACTAAATCACCATCAGCACCACTCTGTGGTCCACCTTGTTCAGGGATGT
CTGGTCGCATGTCATTAAGTGGGTTAGATGATCCAATGGCGGCTGATCGACGATTGGGAGGAGGTTTAG
GGTGGTTATGGGCCCCTTTGTAGATGATCTCTGTTATATGACCCTCATGAGATCGTTCAACCTTTTTCTTA
ACCTGACAATTTGGATGCGTGCACTTGTAATAACTTCGTGGATACTCACTACCTTTTACTTGTTTCTGTC
CATATTTTCTCCAATTATATACATCTTCGGATGGTGTACCACCAGCAGCAGCAGCCATGGAATCTCCACT

ACCTCTTTGATCTCCTTCTTCATCTGGTTGCTCATCAAGGGGTGGAGAATGCTCAGTACTGCCACCAAC
AGTATCAAAAGCCCTTGGATCTGCTGAGTTAGCTCCATTATCTTTTTCAGTAGATGTGCGAGAGAAGTC
TGCCTGGAGCTGAAGATTGTTTGTATTCTGGTTTTGAACTTTGGTTGGTTCTATACTTTGAGAAGAATTT
TCTGACTGGACTGACACCTCGATGCTGGCAAAAGATTGCTGTGGAAAACTAGTTGAACCCATCTGTAT
ACAGGAGAAAAAATTACTTATGAACTATGAGAGATATTATGACATAAATGCTGTTTTGTTTGCAGCGGG
AGTATACTAGGACTCTCCTCTTAAGTACTGACCAGTGGTTATCTAAATTTCACTATAAAAAAATACAATA
GAACTAGCATGTGTGAATACTTGTGTGTTCTAAAATGAGGAAAAATCGAGGGTGAAATTACTTTGCTAG
TTGGACCAAGAAAGAAGGAGCCTGATTCCGCAATAGGCTTGAAAGCGAATGAAGTATTTATGTCCTCA
AAGAAATTAGTTTTATCGGGGCCCTCTGTCATTAATGTGGAACTCCTGCTATGACCATTTGAGACAAAT
GGAAATTTTCCAGTTGTTGGAGATGGCTGTGCCTGCAAGACATCCAGAAGAAAAGTTTTAACATATGG
CAGGACAAATGTCAGCGAGACTAAACTTACTTTTAAATTTCATCACAAGTGGAAGCAAATTTTAACTTA
AATCAGGGATACTAAACAATCTCTATAATAATTTACATCTACATGCCCTATTTCAAAGGAAAGCAATCCC
AAGTCAAATAACTTCATATGATGTATTACAAGAACATGAATTTCAATTTCCTGAAGGTACTCAAATCATA
CCAAGGTGAAAGGCATGTATAGAACCAGTTAACAGCTCATCCCTGTACACAAGATACAGATATCTGCAA
TTTTGCATTCAGGTTAATCATAAACTAATTTAAAGTTCAAAACTCTGCCAAAATATGCAAAAACCGATTA
TCTTGGTTTTACAACATTTTGTCCATGTTCCTACCATTGCAAGAACAAAAAATATTTCACCAACCCCATA
TGCACGCCTTGTACTTGATGAGATCAATTTTCCGCTTTAGTCCATTAGAGGTAAATAACCCTATACCAAC
ATACAAACCCTCAGCCCCAGTAAAGGCAAGATCTGATCCCAGGACTTTACCTAAACTAGCTAGCACCT
TCCATGAAATCAAATTTTTATTATATACTAACAACACAATAAAAGTAAAAATTAACAAAGAAGCAAAAA
TATAAGGAAAAGAAATTATATTTAAGCACTACAGATATTTTCAAAGTTGCAATCTTACCAATGAACTTGA
AAGGAAAACAGGAGAGTCTAGAAGTATGGTTGGACTGAGACCAGGAGGTATTGTCAGATAAGGGGAC
CGAATGTCCGAATTAAGTGAAAGGTCAGAAGATCTAATGCTTTCTGTATTCAGCCGCGGAGCATTAAAC
CCAGCTCTGGCTGCAATCCTTTCCACAAGCCCTCCGCGTGAGTTTGACTTGTACTCCGAAAATGAACC
CACTTCATTTAGTTCCTCACCAGAAGCATTGCCTTGTGACAAATTTTTTCCATTGTTATCTTCTTCTTGA
GACCCCAAAAAGAACTCCGCAGTTTTATTACTGCTGGGAGGTTCCAGCATTGATCTTGAGCCTATGTCG
TCAACTAACATTGAAGAGAAGAAGGCTCTTGGGCTTGTACTTGGAGGCACCCAATCTCCAATTATAGC
AACATTATCATCGATGCCAGCCAT
>PmWRKY03
TCAGACCCATCTACCCGTCAAATCTCCAGCGCCAAGCCTGTAAAAGCCTAATGATAATAATGATAACAA
TGACAATAATGAGAGAGAGAGAGAGAGAGAGAGAGAGGAGCACATCATGTGTATGGGGTACAACTAGAA
GCTAAAATCCAATTTGACATGCAATAAACTTTTGCAGAAGATGTGATTAAAGCAAAAGAATTGAAGAA
AAAGAGAAAAGAAAAGGAGAGACGAGTGTAGAAAATGTGAATTGTCTCACCTGGGATGCTGCGTGTT
CTTTACCACCTTCTGTCCGTACTTCCTCCACTTGTATCCATCATCAAGCACATCAACCTCACTCATGGTT
TTGAAGCAAAACCTAGGCTCCCTCACCTTCCTCCTAATTGCCTTCATCTTCTTCATCTTCATTGCTGAAA
CTCCCATATGGTGATGATGGCGATCATCTCCACCGTTTGATCTCTTGCTGCTTAGGCACTCATGATCATC
GCTCACCTCTCCCCATGCCCTAGACCATATGTACCAAAACATGTTTATACTTTTAATATTCTTAGTTTTAC
ATGACTAATTGTATTGCATGCAAGAGAATTCAATTCAAAAAGGAGATCAAGATTCAAGACTACATATAT
AATCACTAGATGTGGAAATTAAGTGTGATATTAATTATATTATTTTACCAGAGATTTGCAGTGGATCTTTG
CAAGGAAAGGAGTTGGGATCCTCCAAAATCAGAACTAGTAGTGATGTTATGATCTTCTCTATGCTTTAG
AGGAGTGAGTGATGGGAGTAGGGTTTCAGAGAGATTGGTGCTTGAAGGTATGCTAGAAGAACCGAAG
GCTTTGAGAGATCCCAATGAAGAAAAGTTCAAGTTTGGTGGGATTGAAAAGAAACCCATCTGTGCATT
AGAACCCTCTTCATTCTCAAACAAGCCCTGCTGCTGGCTGAGCATGGCCTGGGACGTCGTAGATGCCA
T
>PmWRKY04

ATGGACTGTCTTCAGAATCCAAACCCTAGCTCTGCCGGCCCTTATCACTTCGGAGAGAGCATCGATCCT
TCCATCGATTTTGATGAGTTTTCAGACTGTTTCATGCTTGATTATGGTGTTGATGATCATCAAGATTCTTC
GTCTTTAAGTACCGTGTCGCCGGAGAAGTTCATGGCTGACTGCTTTACTGGATCCAGTGGTGGTGCAA
CATCAAGAAATAGCAACAATAATATGTAAGTAATCCTTATATTTATTATCATTTTTTTTTTTAAAATTTTTT
TTTTCTGAATATAATTAGCAAGGTCTATTTTATTTTGTTTTTGAAACTATGAAAAAAACTTATTTTGATTT
GATTTTGTAGGAAATGCAGAAATGAGGGGAGGAGAAACAAGACAGAAATGGGTCATAGTAGGGTTGC
TTTCAGAACTAAATCGGAGCTGGAGGTTATGGATGATGGATTCAAGTGGAGGAAGTACGGAAAGAAG
TCAGTCAAGAACAGCCCATATCCAAGGTAATAATGATAATATTTATTCTATATCTAAGATTAGGAGGAGA
TCAATTATATTATCTTTTTATTAATACCATTATCCATAATGCTTTTCTTAATTGAAATTAGATTGCATAAGAT
AATGACAAACTTGGATATGATAATTAATTATAAGATAATAAATTTAATAATTAGGCTATTTGAAAAACTCA
GCAGGATGTAAGAGCTCCTACTTACTTGATAAGAAGAGAAGTATGGCATTATTAGAGATGACAATTTTC
CTCACGGGTAAGATCTTCTCAGGGATTCAACCTAGTGGGTCGGATATGAAGGAAAAAAAAACGAGTAT
GGGTCGGGTATGAATGACAATTTTATAAATATGCTATTAAAAGTGATTGTACCGACTAAGTTTTATATTTT
ATATTATTATATTATATAATTTATACAAATCTTAAGCCACACTAGTTATTTATTTAGTTAACTTCCAATCTAT
ATATAAACATTCTTATATTGTCTTATAATTATATATTATATATATTCATTATCTTGTCTTTGTACTTTTTTTTTT
TCAACAAAATAGTTATATATTGAATTTTTTTTTTTTCTGTAATGGGGTGGAACAGGGAACCCATTCCCCA
ACGAAGGTGGGAATGGGGAATCCCCGATATGAATTTTGCCAGTATTAAGGCAGGGATAAGGGAAAAAT
GCTTAAAGAGGATGGGGATAGGGATAGGGATGGGAATGACAATACCCTCCATGGATTCGACCCATTGC
CATCCCTAAGCATTATTTAGGCTATTTGATCCGGTGGAGAGAGAGAGAGAGAGGACTTACTGTTGAAG
AGGCATGCTTATGTTTGCTGCTTCTGCATCCAATAATATTGTTCAAAATGTTGGGGTTGAAGATGACTAG
AGAAAGGCATGTAGTTGTCGATTGCCATTGATCACATCAAACGGTTCAAAATGTTGGGGTTGAAGATG
ACTAGAGAAAGGCATGTTGTCGATTGCCATTGATCACATCAAACAATCAATTACCACACACACACGCAT
AACACAAGGGCATAAAAACCAAGACAGGTCACAACTTGTCATTTTGTTACTCTCAAACTACATACTAC
GATAATTAAGATAATCCGCTCGAGATGATTTTTGAAAGAAACCTAAAAAAGAAAATAGACCGTCGGAA
TAATTGAAATAAAATACGTGTTGGCGGCTTGACTTGAGAGTTGTGGTGTCTCTCCTCTCTAACCTTGTC
CATAGCACTTCTGGAGCACATATCACTTCCAATCACTACACTCAAGTTGACAGACAAATCTAAAACTAT
TATTTCCTTTAAATAAAGGGCACACACACTTCATTATTTTGTACTTTCTTTGTCTACGTGTGCTTAGTTCC
TTTCCCTTTTACTAGATTCATATTCTCCTATATATATATAACTATATATGTATATATATATATATGTGTCATGTC
TAACTTGAGAAAAATAGAATCCCTCATGAACATGGGCGGAGTTAGAATTGTAAGATTTGGGGCCGATAT
ATAAAATACATAAGTATTCAAAAATTACGTTAAAAGCTATATATTTTAAGTTATTTATGTAAAATTTGTTA
GAGTTTTAACTAGTGTCCCTATCTATTTGTAATTGTTGTTTATCACCCTATTTTTGGTAAGTAAATAACAT
AAAAAATCATGTATATACATAAAACGTAGTGGTGACTAATTTCACTATCTGTGGCTACGCCGCGCGCCCT
TTGTATTTTTTATTAATTATTTCTAATTCGGGAATGGTTCTCATGACGTTTTTGTGCCTAAATTATATTCAA
CAGAAATTACTACAAATGTTCAAGTGGAGGGTGCAATGTAAAGAAGAGGGTGGAGAGGGACCGAGA
AGACTCGAGCTATGTGATAACCACATATGATGGAGTGCACAATCATGAGAGCCCTTGTGTGGTATATTA
CAATCAGATGCCTCCTCCTGTGGATCCCAACAACATCTGGACTTTGCGAGCTTCTTCACAGTCTTCTGC
TTCTTCATAG
>PmWRKY05
ATGGCTGTAGATTTTATGGGTTATAGGAACAGCAGCTTCTCTGCGAAATTGGAAGAGAACGCGGTGCA
AGAAGCAGCTTCTGGGCTCGAGAGCGTTGAGAAGCTCATTAGACTCTTGTCTCAGGCTCAGCAGAAC
CAGCACCAAGATAAGTACCCTTCCATGGTTATGGACATGGATTGCAGAGCCGTCGCGGACGTCGCCGT
TTCCAAGTTCAAGAAGGTCATTTCTCTTCTGGGTCGTACCCGGACCGGCCATGCCCGCTTCCGGCGAG
CACCTTTGACTTTGACTTCTGGGTCTTCTTCTTCTTCTCAAAACCAAGCCCAAACCCAAGAGACCTTTG
TCAAGCAAGCTCCTTTAGAGTCTACCAAGGTTTACCATGCGACGCCGATCCAGCAGATCCCGCCTCCT

CTGCATCATCACAGTACTGTGCTCGAGAGCACCAAGGACTCCTCCACCACTATAAATTTCTCCTATTCA
GCTACGACGTCGTTTATGTCGTCGTTGACTGGAGACTCCGACAGCAAGCAGCCATTGTCGTCTTCGGC
TTTTCAAATTACCAACATGTCCCAGGTGTCTTCAGCTGGAAAGCCACCGCTTTCGTCAGCTTCGTTGAA
GAGGAAGTGCAGTTCTGATAACTTGGGCTCTGGCAAGTGCGGTGCTGGGTCCTCCGGCCGCTGCCATT
GCTCTAAGAAGAGGTGGGTCCCTCTCTCTCTCAAATCAATTTCTACGTTCTAATTAATTCGATTTAGTTG
TTGGTCGAGTTTAACTTGTTGACTAAAATGAAATTTTGGGTTGTGGGGTTTGTTGCAGAAAGCTGAGAT
TGAAAAGGGTTGTGAGAGTTCCAGCTATAAGCTTAAAGATGGCTGATATTCCACCTGATGATTACTCTT
GGAGAAAGTATGGACAGAAACCCATTAAGGGATCTCCACATCCAAGGTATAATCTTGGGAAGTTTTGA
CCTTTCAAGTTCAATTAGTTCAATTTCACACTTTTCTCTGCCTAAAACTCTGAAATAGCAACCCTTTTAG
GCTTTCAGGCTTTGAGATGTTTTGGTCTTGTTTTAATCTGTGTTTTGGTTGTTGTTTTTTATGAAACAGG
GGATACTATAAGTGCAGCAGTGTCAGAGGGTGCCCAGCTCGAAAACATGTAGAGAGAGCTCTCGATGA
TCCAGCAATGCTTGTAGTGACCTATGAAGGCGAGCACAATCACTCTCTCTCAGTTGCAGAGACCTCCA
ATCTCATTCTAGAATCGTCTTAG
>PmWRKY06
ATGGAGGACGCAATGGGTGCCACGTGGTCCGACTGGTCGGAGGAAGAGCTTGTGAGAGAACTTCTGG
ACAATGAGACACCATTCTTTGTGCTACCAGAGGAGGCTCTACAATCCCAGATGAGTGTTTCAAATGAA
GATAGTGTTGTTAATCGGTTCATTCCGACCGTCTATTCTGGTCCAACAATCAAAGATATCGAGACCGCTT
TGTCTGTCACCACTGGAGCAATTCAACCCCAAGAACTTTCACCGGCTAGGTTTGTATATGTTACAATAA
TAATAATTATTTTCATTTAATTTTAATTTTAATTGGGTTTTTCTTTTCTTTCGGCGTATTATTTTGCAGGCTC
TCAATGCTGGAAAGGGGTTTGAGCAAGGTTGAGCATAAGTACACTCTGAAGATTAAAAGCTGTGACAA
TGGAGCAATGGCTGATGATGGCTATAAATGGAGGAAGTATGGGCAGAAATCCATTAAGAATAGCCCAA
ATCCTAGGTAACTATTTAGTCTCATTCTTTCTTCTTCGTTTTCGTCTTCCCCTCTATGGAATTAAGTATTT
TAAAAGCTGTACGGTTTGGTATTATTGCCGCCATAGTAAATATCATATGATTGTTTTTTTTCAAGATGAGA
GCCATAGTTTCTCTTTATTAGGTGCAAAGGTAAGTACTTTATCGCTGAAATTATATGAATTTTAATTTCAA
AATACTAATTATTAAACGTTGAGTAACTTGTCGTGAAGGAATAATGCAAGAAGCTTCAAGAGAAGTGT
GAACAACGATATATTTCTTTCGTAACAAGATAATATATGACTATTAAAAAGAGGAAAAATAAGAAAAAA
TAGATCAAATCTATATATCTGTATTCATAGATTAGAATGCTAGTTATTTCAAACTTGAAAGAAATGACAA
GTGATGCATAGAAGTCGCATCATTTGTTTATTTCAAACCTAAACCATTCAACTTTGCATTGAAGGCTTAA
TTAGGGTCTTAAACCTACTTCATTATAGTATACGTATGAGTCCGAATCTGCATGATTTAGGCAACTTTAAT
ACTAATTAAGCAGACTAGTTTACAAATTGCTCTCTAATTAGTACTGCCCCATGAATCCAACCGCCCTAAT
CAACACCTTAAGCAGGTAGATTATCCTTAAATATGCCTACCTATGCAAGTAGCTTGACCTAAATGAGGTC
CAAGCCTAGGCCTAGGTCTAGGCCACACTATCAACCCAGACAATAAATTAAAACAGAGTTTGACACAT
TGCTACAATCATGCAATTAAAGTATATCTCAAATTCTTTTCTTTTTTTTTAATAATTATTTATTTCCATTTTC
TTACCAAGCTTTTTTTTGGGCTGTGTTGATTATGAAAAAATGAAAAATAGGAGCTACTACAGGTGCACA
AACCCAAGGTGCAGTGCTAAGAAGCAAGTTGAGAGGTCCAGTGACGACCCAGACACACTCATCATCA
CCTATGAAGGGCTCCACTTACACTTTGCCTACCCATTTTTCCCACTCAACAATCAGGCCCAGAACACCA
GCCCACCTATGAAGAAGCCCAGAAACAAAACCTCACAGCCACAAGCAGAAGACCGTGAACATGAAG
CCCAAGAAAGCCCGCGAAGTATTACCCCTGAACCTGACCCGGATCTGCAGCCCGGCCCATTCCCCGAC
CCACATGAAGAATTTGTTGAAAAAGAGCCAAGCGCACAAGGGTTGCTTCAAGATGTGGTGCCATTTAT
GATTAGGAACCCTAATCCATCGAGTGCTTCTTCAAATTCTTCTTGTTCCTCCTACCGGTCGTCTCCAACA
TCTCCTTCTTCTCTGTCATGGGCTACTTCGTATTTCGATGTTGGTTTTAATCATAGCTTTGGATGA
>PmWRKY07
TCATGGCTTTTCTTTGGTTGTTGGGGCCTGTGCATCCTTTTCGGATGCTCCTGGGTTTGTGTTCCTCAGT
GGCATGTCATGGTTGTGCTTTCCCTCGTATGTTGTAATAAAAGCTTTGGGATCGTCTGAGACTCTTTCGA

CATGCTTACGCACACTGCATTTTGGACTGGTGCATCTATAGTAACTTCTGCAAGATCGAAGAAAGCCAG
ATTGTGTAAGCACAAGCTTGCTAGCCAAAGAGAATCCTGTGTCCCGTTGTAAATGTCATGAATGTACTT
CAGCCATTTATGAACAGCTTAATCAACCGAGTCTTAACATAATCGAAAGGGGTCAATTTGAGGTTTACA
TTTANNNNNNNNNNNNNNNNNNNNNNNNNNNNNNNNNNNNNNNNNNNNNNNNNNNNNNNNNNNNNN
NNNNNNNCAAAAAAAAAAGGATTTCCGAGGTAATTACCAGCTCAGACAGACAGGTGATACTCATGCA
AGAGGATTATTAGGCAATTGTTCTAAGTGGTCTGGATACATTGACTAAAAGAGAGAGAAAAATCTTTCT
CACCTGGGATATGGATTTCCCTTAACAACCTTCTGCCCATATTTCCTCCAACGGAAGCCATCACCCGTCA
TATCAGAATCTACAGAACTTTGCACCACAACACGGGGATCTGGCACACCTTCTCCTGATATGCCCCCTT
CATTCGATTGATTCTCACTTTTTCTGAAGGACAAAGCATAGGTAAACATCGTCAATGACATTAAAGTTG
AATAGATAAAATTATGATAGAAATGCTCATTAAGAATGAAAGAATCAAGATACATCGAAAGCATATTTAT
TCCATGCGGTCAACGTCAAGCACAGAGAAGGGAAGAGGCAAGAAAATGGATACAAACTAGCATATAA
CCATAACAATGATAAGCAATCATAGTTTTGTTTCAAATGTTAAGGTGCACTACCTTTCTAAGCTAAAATA
TGCATTGCTAATCTAAAGCATAGGCCTTTGATTTTCATCTACCTGCTACTGAATGATCTTACCTTCTTTTA
CTTCTAGGTTCATAATCCTCTGCTTCAGGTCCCTTGCTTCCTTCCTCACATTCTCCACTAAGACCACAAG
AATTATCAGGAGTTCCACCACCAGCATTTATTCCTCCACTTGCAAGAGGATCATAAAGTAGTGGAGCTT
TACTTTGATAAGAATGCGCAGGTAGTCCAACTTCATTCTGATCCTCTACTCTACCTTCAGAACCTTCATT
CCTTTCATTAAGTTGACTATTCCATAACCGGTTGTTGGTATCTTGACCAGTTCCATCAGATGCTAAGCCT
AACCCTTGCGTCCCCGATGAGCTTTTCTTTGGAGGTTGAGGCTTTGAGTGGTTGTGTTCACCCTTGTAG
ACAATCTCAGCAATCTGTCCATCCAAAGATCTCTCAACCTTCTTTTTCACAGGACAATTTGGATGTGTG
CACTTATAGTAACTTCGCGGGTACTCACTTCCTTTGACTTGCTTCTGTCCATATTTTCTCCAATTATACCC
ATCATAAGAAGGCCTATCAGTATTGGCTGTGGACGGTATATGTTTTGGATCCTCTTCCACATTTTGTGAT
GCTATCTTCAAAGGCTCTATTGTCTGATTTGTCTCAGCACATGATGGATTATTATGGCGCAGATTTGGGC
TAAGTTGGGATCTAAAGCATTTATCTTGATTTTGATGTAGAATACCAGCCTCGACTGATGATTGTGTAGA
TTGGTGGCTGGTATTGAAGTTTCCCTGGTAACATTGAACCAAAAAAAAGATAAGTTTTTTTCCAAATTT
GAGGTTCAAAAGTTTCACCACATTTTCCAAGTTCATGTACTTAACACATTTATTCTACATACCACAGATA
ATACTCAGAAAAATCACATTTATTACCAGGAAAGTCACATTTATTCAGTTAAATTACAAACATACAATAC
CTTGCTACTGACCATATTTGCCAAGACAGAAACAGTTGCCCTTGATACAACTTTTGCCAATGGTTTGTAT
ACCACACTTGCTCTGCTGTCTGATTTTGAGATCTTCTCTGATGAATTACTATGTGCAGTACCAGACATCT
CTGCCTGAAATTTATGGGCACCAACACTCAGAAGGAAAGAACTTTTCAAAAGAGACAAGCATTTAGTA
GAATTAAGGTCACCTGGGAAGAAACCAATCCAGAGACAGCATGATTCACCGTTGGCTTGAACCTCAC
AGTCTTTGGTCTGATGGCAGGAACAGCAGTTTCAGAAGATATATTAGAAGGAGAGGCATCGATAGCTC
CTGCAAGAAGCTCCGTGAAGGACCTAAAGCTTGAACAAGTAGGCCTTGAAGCAACTGGTTTAGCTATA
ACTACCCGCTCTGCCTCCTTAATATCCAT
>PmWRKY08
ATGGAGTTGTCTTGGCCGGAGAGTGTGCTATCAAATAGGGAAAGGGTGATGGAGGAGTTGATCCAAG
GGCGTGAGCTTGCAAGTCAACTTTGTAAGGTTCTTGATGATCACAAGTCAACACTTGTTAGTGGTTGT
GGTGGTGGTGGTGGAGGAGATGTTGGGTCGGCAGAGGGTCTTGTTAATAAGATTTTGGGGTCATTTAC
AAATACCCTTTTGATCTTAAATGGGAAGGAGGCTGACGGGGAGATTGTTTCTGGTGATCAGATTCAAG
GGAATAGTAGTGGTATTGGGTCTGCAGATTCATCATCTTGGGATGCCAATCATGCTATTATTAAGTCTGA
AGATTTTCATGAGGAGATCAGTTGTAAGAGTGCTTCAACCTTCAAGGATCGTAGGGGTTCTTACAAGA
GAAGGTAACTTAATTATTGTTTACTACACTTCAAGTGATTTCTTTTAATTTTTTTTTGGCTCAGGATTCTT
AATTGCTCTATGAGGGTCTATCCCACATATGAGCTCCCATGAGAAATTACACTAAAGAATTTTGTTTAAT
AACAAATGTTAATTTAATCACAACAACTGTTAGATTCACATAAATTCATAAATTTTTTCTGAAATAATTAT
TTACATGCATGCTAATCACAATAGTTAATTTACTTACAACAATTGTTGAATTATGTTAATATTAATCTAAG

AGCTGTCGTGAATAATTTACTCACGACAGCTGTTGGACTCGATATACGCATAAAGTTTCTATTAAGACA
AAATAAGGGAGAAGTAAGGACCATGCCACTTGCTTTTGATAGGTTCATATTCATTCTATTCTATGCCAAA
AACTAACAAAATTAGGATATACTTTAGTATTTCCCTCTATTGAGTGACTTAAATACCCCTTAATCCTATCT
TAATTACCACCATATAGCATTATCAGTAATAAACAAACCAATCATTTCATCCACATGAGAATTTTTCCAAA
TGAATGAAGTTCTAAACAAAAGAGTCACTTGTTAATCATTTGCAGGAAAACTTCACACTCTTGGACTA
GAGACACTCCGGCTTTGACTGACGACGGTCATGCATGGAGGAAGTACGGACAAAAAGTCATCCATAAT
GCCAAGCACCCAAGGTACACCTTACTTGGGAAATCAAATATAAATTCGAAATATTCGACACAAATTAAA
GTGACAATAATTTTTTCAAGAGATGAAGTTGACCAAGATCATGTTAAATGATGCAGGAACTACTTTAGG
TGCACCCACAAATTCGATCAATCGTGCCAAGCAACCAAACACGTGCAACAAATTCTAGATGATCCACC
AGTTTTTCGGACCACATATTATGGCAACCACACTTGCAGAGACTACTTGAAAGCTTCTGAGTTGATCTT
GGATTGCACAAGCCCTAGAGAGTCTTCAAGTTTCATCAGATTTGGCGACATTAAGCAAGACCACCCCT
TTTTCTCATCTTTCACATCAGTGAAGAAAGAGGTCGTCATCAAGGAAGAGAAACCACAACCAAGTGAT
GATATGATGGCAAGCCACCACCACAACCATTCATCATCCGGTGATTATAATGTGTCACCTCATCCGACG
GAGTTCAAGTCGTCTTGTCCCCTGAGCGGGCTTTCGTCAACCATTGATCCGTATGATCATGAGGGCGAT
GTGATGTCTGGTTTAATTGTGGGGCCTTATTACTTTGATGATGAAGTTTTGCAATATGAATTTTGA
>PmWRKY09
ATGGAGTGGTCTTGGCCGGAGAGTGTGTTATCAAATGGGGAAAGGGTGAAGGAAGAGTTGATGCAAG
GGCGTGAGCTTGCAGGTAAACTTTGTAGGGTTTTTGATAATCACAAGTCAACACTTGTTAGTGGTTTTG
GTGGTGGTGGTGGTGGTTCGGCTGAGGATCTTGTTGATAAGATTTTGGGATCATTTGCAAATACCCTTT
TGATCTTAAATGGAAAAGAGTCTGACGGGGAGATTGTTTCTGGTGATCAGATTCAAGGGATCAGTAGT
GGTGGTGGCTCTGCAGATTCATCATCTTGGGATGCTAATCATACGGCGGCTGTTATTAAGTCTGAAGATT
TTTATGAGGAGAGTTGTAAGACTACTTCAACCTTCAAGGATCGTAGAGGTTCCTACAAGAGAAGGTAA
ATTAATTCATTGACAATTAATACATCCATTTAAAGAGTGATCATCATCAACTCGTTTAGTTAATTGGGTGG
ATTGATGGTTTAATCTTAAAATTTCATATGCTGTATGCGGGATCTGACTACTGGTAGGAGTTCATGTGAA
ATATATTACTAAAAAGTTTTGCTCATAACAATTATCCACACACACGTAAATAAAATATAGTAATTTTATTG
TGATAAAATAAGACAGTTGTTAATCTAGTCTTCAATAAATAAAAAATAAAAAAAATTGGTCACACCAAT
TGTTAGTTTCACATAAATTCATAAAATTTATATCGCGACAATATAATTCACATGCATGCTATATAATAAATG
TTAATTTACTCACAACAGCTGTTAGATTCGTATAGATGCATAAAATTTCTATTGAGATAAAATAAGGTAA
GAGTTAGCTAGGACCACCCTAGTTGCTTTGATAGGTTCATTCCATGCCATATACTAATAAAATTAGGATA
TGCTTTACTCNNNNNNNNNNNNNNNNNNNNNNNNNNNNNNNNNNNNNNNNNNNNNNNNNNNNNNN
NNNNNNNNNNNNNNNNNNNNNNNNNNNNNNNNNNNNNNNNNNNNNNNNNNNNNNNNNNNNNNNN
NNNNNNNNNNNNNNNNNNNNNNNNNNNNNNNNNNNNNNNNNNNNNNNNNNNNNNNNNNNNNNNN
NNNNNNNNNNNNNNNNNNNNNNNNNNNNNNNNNNNNNNNNNNNNNNNNNNNNNNNNNNNNNNNN
NNNNNNNNNNNNNNNNNNNNNAATGAGTGACTGAAATGTCCCTTACTCCCATCTTAATTATCACCATA
GCATTATCAATAATCCATTCATTTCATTTTACTAATTTAATTGAGTTCTAAACAAAAGAATTACTTTGTCA
ATTGCTTGCAGAAAGACTTCACACTGTTGGACTAGAGACACTCCTGCTTTGGCTGACGACGGTCATGG
ATGGAGAAAATACGGACAAAAACGCATCCTTAATTCCAAGCACTCAAGGTAACATGCATACGATTTGG
TCTACTTATAGCTCTCATAATTCCATATGGAAATATATAAATTCGAGTCTTCATTGGACTCAAATTAATAA
AGTAATAACTTTTGAATAGTTGATGTTGACCTTGGTCATGTTATACGATGCAGGAATTACTATAGGTGCA
CTCACAAGTTCGAACAATCTTGCAAAGCAACCAAATACGTGCAACAAATTCAAGATCATCCACCAATG
TTTCGGACCACATATCACGGCAACCACACATGCAGAGACTACCTAAAAGCTTCTGAGTTGCTCTTGGA
TTGTACAAGTCCTAGAGAGTCTTCAAATTTCATCAGATTTGGCGACACCAAACAAGATCACCCCTTTTT
CTCATTCTTCACATCAGTTAGAAAAGAAGAATTGGTCTTCAAGGAAGAATCGATCATCAAGGAAGAGA
TACCACCAACAAGTGATGATCGTGTGATGACAAGCCACGACAACTATTCATGGTTGTGTGATTATTTTG

TGTCACCCGATCTGACGACGTTCGAGTCCTCTGGCCCCCGAGTGGGTTCTCATCAACCCTCGATGAG
TTTGATCATGAGGATGTGATTTCTAGGTTAATGATGGGGTCTTTTGACTTGGATGAAGTTTTGCAATATA
TTTGA
>PmWRKY10
ATGGAGGGAGTGTCGTCAAATCGGCAAAGTGAGATGGAGGAGCTGATCCAAGGTGGTGAAGTTGCCA
GTGGTCAACTTAGTAGGGTAATTAATCTTCATAATAATAGGTCACTTACTGTTAATGGTGATCATAATCAT
GATTTGGGATCGGGCGATGATGAAGGTTTTGTTAATAAGATGTTGGGGTCATTTTCGAATACTCTTTTCA
TCGCAAATGGGAAGGAGATGAAGGAGTTTCATCATCATGATGATCAGGAGCTGATTTCTGATCAGATTA
TTCAAGTGAATACTGGTGGTGGTGGTGGTGGTGGTGGTGGTGGTGGTGGCGGTGGTGCAGGTGCATCA
TCTTGGGATGTTGATCATCTACTTGATCATGCTATTAAGTGCGAAGCTTCTCATGAGGAGAGTTGTAAG
AGTGCTTCGACCTTCTCCACGGATCGCAGAGGCTCTTACAAGAAAAGGTATATAATCAATTAATTACAT
TCACTATACTATTGATATATATATATATATATATATAACTATTTCATTACTATAATTTACAGAGATTCATCAAC
TCGCGGTAATTATATGAGCAAAATATATAGATCTCACTGCAAGATTAACTGTTTGTTTTTGTTTCTAATAG
AGGTAATTTCTTTCGTGCATGATTGATTGATTGTTCAATGTCAAAAGTATTCAATATAAACGTCTTAATG
AGGTATATGATCGAGTTAATTGATGCGTGAAACGATCTTTGTTCTTCGGGAGTGCTTGAATCGTGAGAG
TAAATTAGGTCGGGAAAAAGGAGAATGAGATTGGGTCGAGCAAGTGAACTATCTTTGTTGAAGAACTT
TTGTTTTTTTTACAAGTCATCCACACACTCATTAGGTCTTTGTTAGGACCTTTCTCCTAACAATTTAACA
AAAGAAGGGATGGAGGCCTGCCAATTTGATTTGATAAGCTCATGCATGCATGTCATGCCATATACTAAG
AAAAACATATACTTTTCGTTCCTTTCAAAACCAAGATACTCTTAAAGTGTAGCTGTTCTTTGGGGTCTTC
CCCCAATTTCATTCTTTTCCTTTTTCCTTCTATCATTTTTCAATTTTATTACTTCATTTCATCAATTTGGCGT
AAATTAATGATCAATGAATTTGTAATCAATCCAGTTCTAAACCAAGAGTTGGAAGTTGTTAATGACTTG
CAGGAAGACTTGTCACTCTTGGATTAAAGACTCTCCTGCTTTGACAGAAGATGGTCATGCATGGAGGA
AGTACGGACAAAAACTCATCCTTAATGCCAAGCACCCAAGGTACGTGCACATACATACAATTTCTCTAC
AACTTATAGGTGTTGGCGAGAATATTTTTGTTTATTTTGTGTAATTACTTACGCTTTGTACGTATAGTTTTA
TTGTAATTAGGATTTCTTTTCTATTGTAACTCTTTTAGGGTTTACCACTCCGAAAATTATCATATACTTTGT
TTACTTATATTGCAACCTTGGTTAATATCTCAATTAATTGGATATGTTGTGAGCATGTGCAATGCAGGAAC
TACTTTAGGTGCACCCACAAATTCGATCAAGCATGCCAAGCAACCAAGCACGTACAACAAGTTGAAGA
TGATCCACCATTGTTTCGTACCACATATTATGGCAACCACACATGCAGAGTCTACTTGAAAGCTTCTGA
GCTGATCTTGGATTGTACAAGCCCTAGAGAGTCTTCAAAGTTCATCAGATTTGACGACACCAACCCCTC
AAGCAAACAAGAGCACCCCTTTTTCTCGTCGTTTAAATCGCTAAAAGGAGAAGAATGTTTCAAGGAA
GAGACGATGCCAAGTGATTATATGACAACCCAGCACCACAACCAATTAGCATTGAGTGATTATCTTGTA
TCACCTCCTGATCTTCCGGCATTCACGTCCCCTGGGCTCATGAGCGGGTTTGCATCAGCCAATTATGAT
GACTTTGCATTTGATGAGTTTCTGCAGTATGAGTTGAGGTAG
>PmWRKY11
ATGGATGAGGTTGAAGAAGCTAACAAAGCAGCTGTTGAGAGCTGCCACGGAGTTCTCAGCCATTTGT
GTCAACCTAAGGGCCAGGTTCATTGTAAAAATTTACTGGCGGAAACTGAGGAGGCTGTGTTCAAGTTC
AAGAGAGTTGTGTCTCTTCTGGGCGGTGGCTTAGGCCATGGAAGAGTGAGAAAGTTGAAGAAGTTCA
AGCCGCCACCTTTGCCTCAGAACATCTTCCTAGATGGCCCTAATTACAGATTAGATATTTCAACAAAAC
CCCTCCAATTACTCCCACCTAATTCTCTTGAAAACCGACGCGCAGAAAAGGATTCTAAGTATGGAAGTT
CTCTGCAGCACACACACAGCCAGAAAGTGTTTCTTGGAAACCCAGTAGTGGATTTGGAAATGAAAAT
CAAGCTCCCTCTCCAAATTCCCAAAACAAAACCATTGCAACAGTATCATTTCCTCGAACAGAAGCATG
ACCATCAGCAAGAGATACAAAGATTGCAGCTTCAGCCCCAACAGTTGAAATATCAGGCTGATACAATG
TACTCTGGGAGAAACCGTGGCATCAACCTTGCATTTGATAGGTCCACTACCCGCACTCCCTCCATGTCG
TCAGCCAGATCATTTGTATCATGTTTGAGCATGGATGGTGGTGTGGCTAACACTCACTGCGATTCGTTC

CAACTAATCCGCGGTGTGCCTCAGCCGCCTGATCGGATCTCACAACAACAAAGGAGGTGCAATGGTGC
CGGGGAAAACGGAACTGTAAAATGCGGCAGTAGTGGTAAATGCCAGTATCCAAAGAAGAGGTGAGTC
TTGACTCTGAAATTTATGTTTTTCAAGTGGGTGCGGGTGTTGTTTGTTTGCTTGTTTAATTAATTAATTTT
GTTAATAGGAAAGTGAGGGTGAAAAGATCGTTTAAGGTTCCTGCCATTAGTAACAAGATTGCAGACAT
TCCAGCTGATGAATATTCATGGAGGAAATATGGGCAGAAGCCAATTAAGGGTTCCCCCTATCCTAGGTA
ATATTTTTATATTCAAACATGCCTAGCTTCTGTTGTTTTGTACAATATTGTGGTGAATTTGATTATGGAAA
GATAAGCAATGTGAGTCTAACTGAGAAGGGTGTGATTGTAGGGGATACTATAAGTGCAGCAGCATGAG
AGGGTGTCCCGCGAGGAAACATGTTGAGAGAAGCCTTGAAGACCCCGCTATGCTTATCGTCACCTATG
AAGGCGAGCACAAGCATTCACCATCTGACCAGTGA
>PmWRKY12
TTATGATGGATAACCTCCCATGATCCTTTGATATAACAGCGATGATGATGATGATGACGACAATGATGCA
TTTGCCTGGTCAATGATAGGCAAACAGGCATCATAAAGGTTATCATCCTTTTGCTCTTGTTTAGGCTTTA
GGAACCTCATATCATCCTCCTGCAGCTGCTGTCCTGAAAAATAAGGCTGTACTGGACAAACTGATTTTC
CATTGTTGAAATCAAAACCAGAGAGAGGGAGATTCCCAGATGGAGGAAGGTTTAAAGGCAGTGAAAT
TGGGAAGTCTGGGACAACAGAACCAGCCTGATGGGTCACACCGTGATTAGCATGTAGTCCAAACGAG
CTATATGGCATGGTATTTTGCAAAGGAGGATATTTCATTTGGTAAATGGAGGGAACTCCAAATTTCAATT
CATTATTGAAATTCCCAAGAAAACTAGATCTCAGGTATTCTTCATGGAACTCCGGTTTTCTATCAAAATG
AGGTGCAAGATCTTGTACTTGTGTCTCAGGCTTTGGATTATTGCTACTCCTGGGTAATGCAAGAGCTGG
CTGAGCATTGGCAGTAGATGGATGTGCATTGCCACCGTTTGAATTTATGTGATTACTGTTTCTCGCTGCG
GGCACTTCATGGTTGTGTTTTCCCTCGTATGTTGTAATTACGAATTTCAGATTGTGGGAGGCCCTTTCCA
CATGCTTCCTTACTGAACAGCCCGCACTTGTACATTTGTAGTAGCTCCTATAAGATAGAGAATAAGTAG
AAAGATTAATTGCATACATATTTATGATTGGAAGCACAAGCCCTATCCAGCCATCATGAGAACTGAACAT
ACCCCAGTATATGCTCAAATGATTTTATGCAACCTAAATTGTGAAAATAAGGAATATGAGCTTTTAATCT
TATTTGAAGAAATACACTAGACCAAGAAATGCTATCCTCTGTTTCTTTTTCTATTTTTTTTTCCTGAACTC
TTCTATGGTCATAACTCAGTAGAAATCCACACAGTAGTCCAACCAATATGATGCGAGAGAGAGAGAGA
GCATACCTAGGGTTTGGATTTCCTTTGACAACTTTTTGCCCATACTTCCGCCAACGATAGCCATCATCAA
GTATGTCGACTTCACTCTCAATTTGGACAACTACTCTTGGTTCACGGACAGCCCTGGATGCTAAACTTG
GTTCCATCAAGCAGCTCTCTTTCTTCCTGGAAGGGAAAATCAATGCAGGAAAATATATCCTGGTTAGTC
ATGATGGCCTGGAAATAGACACCTGTAAGATAAACGGAAAATGACATACCTTCTTTTTGACTCAGATTC
TTCATCATCTGCATCATCTCCAAGTGATATGCTTCCTTGGGTGCCCCGATCATCATCATCATGACTAGCA
AGTGTGGATGAAAACTCTGGAGTTTCTGCTGATTCAAAGATACTCATGGATTTCCCTTGGGTAGTTGAT
AATGGATCAGAAAGGTCGGTGACAACAGATGTTGAGGATGTCCTTTCCAGACCTTCAGCCCTCCCATC
ATAACCAGTTTTAATATCCTTACCAGACTGAATATTTGTCCAAACTAACCCACCTTCAACTTTGACGGA
GGCCCTACTTCCTTCACTCATCTCTGACGTCTCATCAAATGAAAATGATGATCCAAGTGATGCTCCAGC
TCGACGATTAGGCTGAGGCATTGCATGATTATGAGGAGCTCCCTTGTAGATGATTTCTGTTATTTGACCA
TCAAAGGATCGTTCCACCTTTTTCTTCACCTGACAATTTGGATGGGTGCATTTATAATAGCTCCTTGGAT
ATTCACTGCCTTTGACCTGTTTCTGCCCATACTTCCTCCAATTATATCCATCTTCTGAAGTCCTGACCATT
CCCGCAGATGGGTATGACCCTCTATGCTCTCCTTCCAAAAATGGGTGGCTCCCAACGTCCTCTCCATGA
ATCGGTTCTTTGGGAAGAGAGTTTTGTTCACTAGCTGCGCTTGAATTAGACCTTTGCAAGTCTACGCAG
TTGGCATTCAGAATCCCACTATTTAAGTTTTTAACATGGGTGGATGGATCCACAGCATAGTTCTTTGCAT
TAGCTTCTTCCGGAAACTCCATTGGGAATTCAAAATCTATTGGTTGTTGCTCCAAAACCAGAGATTGGT
AGTCAACAGTAGATCCCTGAGGAACAATGGAAAGAAATTAATCCAAACACAATCTCATATAAAAATCG
TAAATAAATTACTGGTCAGAATTTAGCAGCTCATCCCTATGGCAACTTATCAAAATCTACACTGGAACCA
TTAACATTTTGGTTTAAACAAAGTACCTTCGGACATTCATGGTTTGAAGATATGCGGACTCATGTGTATC

GAAGAACCATACAAAGTTGTTTCCAACAACTATGTATAAAATTACAGCTAGAGATTATGAGGAGATACT
GTTTTAAGGTTTTTCGTGGTTACAATTGAAAATTTAAATATTATATTTAGATATAACATAATCACTAGGTT
AGTAATTAAAATACCAATATCTTGTGTTGCCACATTTGGAAAAAGTAATTAGACCAAAAAAGAAGTTTA
AAAACCTCTCTCCAGCTTAAGAGTTTTATACTACTGAGTTCCAAAACATTAAGTTGAGGCATCTAATCC
CTAATTATTTTCGGACGAAAGAAACATGCCATGTAACCATTACCTGATTTTCGAAACTAGAATAGCCTG
GCAGATATTTGGGATCGCCCTGTCTCTTGAATGTGAAGGATGAACCCAAATCACTCCCCCTGTGACCAT
CTTCATGAGTTCCAGATTTCAGCACTGAACTGTCATCATTGGGAGGAAGCAACGGAAAAGTTCCGGTG
GTTGGAGATGGGAGCGCCTGGAAGAATATAAGAGTTGAAGATCACATATCAAACAGCATCAAGCAGG
AATTACTAGACATCATTTAAGACTTTCCATAATTAACTTCACTCCCACACCAAAAAAATAAAATTTCTTT
TGTCTACTTCGTAACTATCAACGTCCACAATGTTCGTTTGCCTTCTTGGTTATGGTGATTGTCCTTTAGTT
CAGCTGCCAGTCCCTCAACAAACCTCAGTTTAAACCAAGAAATTACAAAACAGAGACTGTTATACAAA
AAGTGGATCTGGAAGTGCATAATCACAAATTCAGATGTTTTACTTTTCTTTCTTAAAAAGAACATATTAT
GTTGAGAGAACACACTCAGAGAACTAGCGTACCCAACATGAGGAATGTCAGTTAATAATAATTAAAGA
GTAGCAGCAAAGTACTAAATAGAAGTAAATTACAATTTTTTTTTTCGCTTGTACTTCTACCCTACTACCA
ATTGCATTAACCATTTCCAACAAGACTCATAAAATTCACATATTGAGGTTGAGAAACCCAAAAATACAA
AATTATACGTTTCGTTGATCAGAAAATCACCAAACAAAGCCCAGATAGAATCTCACACATTTAGCTCGA
TTCAAACCCCAAAATTTACAACAGCAAAAGAATTGCAACAAAATTACCAAAAATAAGAAAACCCCGA
ACGAGAAGAAAAAGAAAGAAAAGAAAAAACCTGAGAATTGGGGAGCATCATGGGGGAGTCGAGCA
AAGCCGTGGGGCTGATACCAGGTGGAATAGTGAGGCAGGGGGACCTGGCAGCCGCGGCCGGCGAAG
GCAAAGGGCTTGTTGTCCTAAAACGCGCCGTATTGATCCTCTCCGCATTGAATCCACACTTGGCTGCCC
TCCTCTCTGCTATACTAATGGTGTTAGCACCAGGTCCACAACCTTTGGTTTTGTTCTCCGAAACGGCGT
CGTCTTCTTGGTCCAT
>PmWRKY13
TTATTCTAAGCTGCTTTTGCGAATGGGCTTACCATTGGCTGAGAAGGACTGGTGATCAAGGGCCCAATT
GTTGCTAGTAGAACTACCATTCTCTCCACCATCCCTTGGACCAAAAGGATGATTAGCTGTATGATTCTCT
TTATTAATGAGGGATGTGATAGCAGCTGCAACAGCAACCCTAAATTTAGGATCTGAAGCAATGGCAGTC
ACATTCTCAGCCAATGACATATTATTTTCTTCCCCTCTCCAACTTCTCTGATCTTCCACTCCAACTCCTGA
TGATCTGGAAATAGTGCTGTTAGCCAAAAGATGGCTGGTTATAGAATTAGGGTTTTGATAACTACTTGC
AGATCTGCTTGTGCTCATCATCCAGTTACTAAAACCTGGTGGTACTGATGAGTGTGATGAGGAGCTGCC
CATGGGAATGTTTTGTGGTGTATTGTTGTGATCATAAAAGTTGTTTGTGAGGTCAAGAACAATTCCTTT
GGAAGGGTCATGAGGATTGATGGTTCTAAAGTTTGATGAGGAATGAGATGAAGGGTTCATCCTGTGAT
CAGGGACATTATAGTAATTAGGGAGGGATGCTTGGGTGTAATTAGAGGGTGTTCCATCTAAAAGATGAT
GGTTACTTGAATCTAATAACATGAATGAAGCTGCTGCAGAAGCTGTTGAAGCCATGGCTGTTGCACCA
ACAGGAAGTGGATGGTTATGTGTTCCTTCATATGTTGTTATGAGTATGGACATGTCCTCTAGGCATCTTT
GGACCTACAGTACCAAACAAAACATCCAAAAAGATATTAAAACTTTTTTTTATAGATGTTATTTAAGAA
AATCAACTGCTATTTATAGTCTAAAGTGCATATTATTTTGCACAGTTGCACTCACTCGTAGAATGAGTGC
AAAAGGATAAACTCGGAGTGTGAACAACATTTCTTAGAAAAAGAAATCGATATTCAATGCCAGTAATC
TTACTAATTAAATACTTAATTTATATGCAAATTTACCTGTTTCCTAACTGGGCATCCAGGAGCTACTGTGC
AACGATAATAAGCTCGTGGGCAAGGGTTTCCTTTTGCAATTTTCTGTCCGTATTTCCTCCATTGACACCC
ATCATTCATCTGAAATATATATACTTATTTATTATCTTAATTACTACAATAATTATAATTTATATGCATGAAG
CTGAAGCTACATATACTCAACGTATATGCATAGAAGAAGTACTAATTATACTCACTGTGGCGGATTCACA
TCTAGCTCTAACCGAAACCCTAGCTTTTCTGTTGGGTTGGGAAGTAACGTGACTCGAAATTCCGGCCA
ATTCGGTTCGCTGAAGCTTGTTCTGCAGTAGTGATGATGCAAGACTTGAACTTGTCAAGTCTTCCTTCT
TCTTGTTCTCTTCTTTATCATCATCATCTTCTTCTCTTCCGTGTGATTGAATATTGGTAGTAGGGCTGGTT

TGTAGCCTCAGTGACAACCCTAGTTGACTCTCTTTCATCATATCTTCACGTGTTGGTGATAATGGAGATC
TCTTTGGAATCGTCTTGGGTCCTTGAACAGCATCGGGATCATTACCATCAAGTGATAGAAAGGTTTGAG
GATCCTAAATCAATGAAATTCAATTTTATATTAAATAATAAATAAAACAAGGCCAGGCCTGATTTATAATT
CATCTCTAAAGGGGGAAAAACAAAAACAAAATTAATAACACAGCATAATTAAAATGGGATTAACTTAA
GTATATATACGCACCTTGTTTTGGTTGCTTTGGTGAAGAGCCGTAAATTTCATTTGGAGATCATAATAATC
TTTCATGGTTTGCTGCACGCCCTTCCTCAAAACTTTGTTCTCTTCTTTTATGCGGCTCATCTCCATTTGTA
AAACGCATAGCTAGTTATGCATTAATTATATACATAATAGTAGTTAGTTAATTTCACCAATTATTAAAAAA
ATAATTATAATTTACAAGGTTGCTATATATGTTTATACATAAAATTCAATCTATACGTACCTCTTGATCCGT
CTTCATGTTATTCTCCTCCAAAGATGTTTCTACTACTGAGGCATCATCTTCAACTTCCCCAGCAGCGGCT
TCTGCTGCTTTTTGGTTGTTATTCTGTGCTCTTGGTGCTTGCTCATCCTCCTCCTCTTGTTTTGCTCCTTG
TTCAATATCATCATCGTCGTTGCGATCTTCCTCTTCCTCTCTCTCTTCCTCTTCGCCTTCGCCTTGGTCTC
CGTCTTCTCCTTGCTTTTCTTGTTTAGAATCAATCTTGAGTGAGAGATCAATATCCAAGCCTATCTCCTT
CTCTCTCCCCAT
>PmWRKY14
TCACGTTGACTCGAACACCAACCCCACGTTCTCCGGCGCATGACGGTGTTCCCCTTCGTATGTTACGAT
CAGCATCGCCGGATCATCCGGGGCACGCTCCACGTGTTTCCTTGCTGGGCACCCCCTAACCGTACTGC
ACTTGTAATATCCCCTGATAACAAAAAACAATTGTCCAAAATATTAGACCTAATCCAGACCGTCAGATC
ATGCTAAATTCTGATTATTTTGCTCCTTTGTATATGAAGGGCACAGATTTAAATTCAATACCTTGGGTAGG
GTGAGCCCTTGATCGGCTTTTGACCGTACTTCCTCCACGAGTACTCGTCCGGTGGAATATCGGCGATTT
TTGAACTAATCGCTGGGACCCGAATGGTTTTCTTCACCCGATTTTTCCTGAATTATAGAAAGAAAAAAA
CAATTAATTTATTTTCTAAAAAAGAAAAAGGTCGTCGGTCAAACTTAAATAACCAAGTATTTTTCCCTC
GCGCGCGAAAAAAGCCAAAAAAATATTTCGCTAAGATGCGCCATTTGGAAAAAGGCGCATCTTATCAT
GTCATCGTCAACAAGTGCTGCGCCCATCAATGGCCCAGCAACCAAACTAGTTGACCCGAAACTTGGAC
GGATCCACCAGATTATAGCCGAGTTGAAATCGTCCGAGTCAACTCGCTTCCCCGGATAATCAACTAAAA
TTTCATTTGAAAATCTTTGGAAAAACCAACTCTATCGTCTCTCAGTAACGGTAAATGCGTATTTTAGCTT
AACAATTAACAGCGGACACAAAAATGCGCAGACGGTGAAGCACGCATACCTTCTCTTGGAGCAATGG
CACTTGCCGGAGCCAGAAGCAGAACCCGAGCCGGAATACTTGCACGACGCGTCGTCGGAGTGGTGGT
CGTGCTCGTGGCACCTCTTCTTAAACGGCGCCGTAGAGAGCGGAGGCTTTGGGCCGGAGACGGCCGG
CGCGGGTGCCAGGAAGATCGACGATCCTTGTTTGCCGTTCGAAACGCTGCCGTCTCCGGTGATCGCCG
ACGACATGAACGACGAGCTCGACGAGACGCTGAAGCTCTCCTTCGCGAACTCGATCTCCGTGCTCTTA
GGATTTGATGCGAAGACATTAGGCCTCGTGAAGTCTAGCGTCATGCTGTGCGGCTGAGATTGGACATA
GCTCGACTCGATCGGAGCCGGCGGGACAATCGGTAGTGTAGTGACCGGAGCCGGCGCAGGAGAGGGC
CTCGGATTCAGAGCCGGAGCCAAGCTCAATGTTTGTGAATACGAGGGATGAGAAGAAGAAGAGGGGA
ACTGAACCGGTTGATCCGGTTGAACAGGTCCGCGTCTGAACCGGGCGTGGCCGGTCCGGTTCAGGAG
CGAAATGACCTTTTTGAACTTGGAGACGGTGTGGTCGGTGATGTCCGTACAGTCCAAGCGAGACGACT
GATTAGTTTGCTGCTGGTGCGATAGAAAGCGGATCAGGTGCTCCATGCTCTTCAAGCCTTGCGATGCAG
CTTCCTGTATAGCCTTCTGATCTTCCATCTTCGGAAAGTTCATCAGGTCCACCGCCAT
>PmWRKY15
TTAAGATGATTCGAGGACGAGAGCAGGCGTTGGATCCGTGACACTGTGGGAGTGATTGTGATCACCTT
CGTAGGTCACAATCAGCATGGTTGGATCGTCTAGAGCCCGCTCCACGTGCTTGCGTGCAAGACAGCCT
CTCTGGCTACTACACTTGTAATATCCTCTGCATTGTGACAAAAGCAATCAGACATTCAGAAAACTCAGC
GGCTACGTTACTCAATTTATTGATTCACACAACTTAGAAAAACGAGTTGAGTGTATCCAATGTCAAATG
TGATATTCACGCTCATCGGAATTCATTCAAATTACAACATTCAAAGAATAATTTATTATAATTTAAATGTC
ACACAATAAATTCCCTTAACAATCAATTCGAACAAGATTAATGAATGAATGACTGATTGAATGAGAATC

TTATACCTTGGATGAGGGGAACCCTTGATGGGTTTTTGACCGTACTTTCTCCATGAATAATCATCCGGTG
GAATATCAGCCATTTTCATACTAATTGCAGGGACTCTGACCACTCTTTTCACTTTTGATTTCCTGATCAA
TCCAAGCAATACAAGTTAAAAAGTCGCAATTTTGAACACATCCCACATTGAAAAAACTACAACCAAAT
TGAAAATCATGATGATTGATGACCGTGGACCAACCTTTTCTTGGAACAGTGGCATCGCCCAGAAGATG
AGCCGCACCTGAGAGCTGCAACATCGTCCATGGAATTGCACTTCCTCTTCAACGAAGAAGAAGAAAG
AGGCGGCTTGCCCGAGGACGACTGTGACATGTTTGTGAACTGAAACCCAGAAGACATTGAATGCTGC
ACAGAACCCTCAGCCTCTCCAGTGGTTAAAGAAGACATAAATGAATTGGCAGCGGAAATCGAAGGTG
AGGGTGAGAAATTGATGGTGGTAGGAGCATCCAGCCTTCTCTCCGCGCACAAAACACTCTTTGTCAAC
ACAACAGGCGTGGTTTTCAGATGGGGATTGTGAGGCAGAGGCGGTAAACGCACACTCGGTGTGGGGC
AGTAAACCTTGAAAGCAGAGGATTGCTCTGTTTTGAAACCAGGGGGTTGATCTGTTTTTGGGTTATGG
GGTTCTTGAATTGAAGGCCTTGGGATTTGGGTCTCTGTTTCCTGAAGCGGCGGTGGCGGTGGAGGCGG
AGATGGAGTGACAGGAGCTCTTCTGAACCGAGCGTGGCCGGTTCTTCCATTGTCCAGTAATGAAATGA
CCTTCCTGAACTTGGTCACGGCCATGTCTGCAACAGCTTTGTACTCTGTAGCAGCTTCAGGGTTCGATG
AGGAAGAAAAACAAGAGTCTTGGAATTGGTGGTGGTGTTGCGAAATCAAGCTTATGAACTTCTCGAC
GCTTTGAATTCCAGCTGAGGCCGCTTCCTTCACAGCATTCTCTTCCATTTGCGCTGCAAAACTGTCAAT
AAATCCCATCATGAGCTCCACAGCCAT
>PmWRKY16
TTAGAGGCTCTGATGGTTATGAATGCCCTCATACGTTGTAATTACAAACCTTGGATCATCTCTATCTCTTT
CTACTCTCTTTTTCACAGGGCAGCCTTCAACTGAACACTTGTAGTAATTCCTGCAACCAAACATAA
CATGCTCAGATCAGATCAAACATGTCTAAAATCACATCTATATTCGATCAATCTTGCTATATTTTTACTTT
GTTTTTTACCTTGGATTTGGGCTGTTCTTCACCATCTTTTTACCATACTTCCTCCACTTGAAACCATCATC
CAATATTTCAACCTCTGATTTTGTTTTGAATGCAACTCTTTCTCTAACTTCCTGCCTCTCCCGTACGGTG
CCGCTTTCATTCTCTCCTGCTAACACAAACACAACCAAATGGCTCGAGCATGAGATGAGATATAGAGAT
TATAGAACAAAAAGAAGAATTAACAAGTAGTTTTATTTTATATGCTTACTGCTATTAGACCCTCCAAGTT
GGCTGCTACTTCCACGAGACTCATCAACTTCATTTGCTTGATACCCCGAATTCTGGACGGTCTCCAAAG
CCATGGAAGTCCGATGATCTTCATCTAGCCACTCACCAATCATCAAGTACTCGGAAAACTCGAAATTGG
ACTGGTCGGAAAAGTCATTCTCAGGTGACTCTTGTGCTCTAAAGTTGTTGCTGTTAGACAT
>PmWRKY17
TCACTGGTTTTCTGTGCCTGCAGGAGGTCTTGTAATGGAGCTTGCAACCGCAGCAGCCAAAGCTTGTG
TGAAATTATGATCTTTGGTTAAATAAGCAACGTAATCTTCAATTCTACTTTCACAGTTGATGTTCGGGGC
ACATGATGATGAGTTTTGAGGGCTTCCAGCTGATCCATTATTTTCTTGATTGCTCATGGATCCCGAGAGT
GTCAGATCAAGGGTGATAGGCACCGGCCGAGAAGAATTCGAAGGGCTAGCAATATTTATGACATCATTA
TTATTAATAGTTGCTGAGATACCATGCGAAGGAAAATTAATAGGATTAAAAATAATATTATTATTATGATC
AGCGGTGGATGATGAACAGCAAAATTGTCCAAGTGGTGAGCCATTGATGGCGTCATGGTTGTGCTCTC
CTTCATATGTTGCCACAAGAACAGACTTATCCACCATGCATCTCTGCACCTAAAATACATACATATATTTC
AAACAAATTAATATGCGAAAATTTTCCATTTTATATTATTAATTTTTTAATTCTGCATTGTTTTATAGAGCA
TCTATTAATAATAAATATGCATGGAAGCTTAGCCAATTGGTGATCAATTCATTTACGTGAGCAACTCATG
TCATGACTAAAACCAGATCTATCCAAAATTTTGGTCCGAATTCAAAACTCTGACTGGTAAATGATATATT
AAATTAATGTTAATTTCTCTGGAAAATTCAAAGCAAAAACGAAGGATTCTAGTTCAAGCTAAGGCATAG
CGTATAGCGTAGGTTACCTTCTTCTTGACCGGACATCGAGGAGCCAAGGAACACTTGAAATAAGCTCG
AGGGGATGATGGGTTGTCTTTCGTAACCTTCTGTCCGTATTTCCTCCATTGATATCCATCTTTCACAATCT
AATCATTTAATTCACAGATAATATTAGTAAACGGTGTATTGGGGGTATTAACTTACCAACTGATGATTAG
CGTTTAGACTTACTAGGGTGTTGTCTTTGGAGTCGGTTCTCACAAGAAACTGTGTTGTCTTCTGGGCAA
CCGGAAACTGATCATGTCGTTCAATGTTTTTCTCTCGGATAAGCTGCTCAAGTGTGTTGCATTTGGTGG

TCATAGCTTGAAGCAAGAATCTCAGAGCTTCATTCTCTTTTCGCAGACGCAGCAAGTCTTGTTGGAGAT
CATCCACCTGCTCATCAAACAAAAATCTAATTATGCTAAGGCCAACATGCAAACATATTTTCATATCAAA
GGCCGAAAAGAAACCCCATATATTTGATACAAATGATATTTGAGAAGGGAATTCGAACAGAGGAACTC
GATGCGCAGGGAACATGATAGCATATCCATGTTAATTACCTTTCTTTTGGTAGGGGTGTGAGCATCCATG
TTCATCAAGATTTCAAGAGCCACAGGACAATATGAAATCAT

>PmWRKY18

TCACCAATTTCCCATCCGAGAATGGTCTGAAAATCTTCCTGACATGGCAGCAGCAAGTGCAGATGCGA
AATTGGGATCTTTGGTCAAGGAAGAAGCCATTTGTTGGACCAGAAACTGTTGAAAAGCCGGTGGAGC
GCCTTCGATTTCTCGGGGGCTCAGATTATTATTTTTGTCACATGAGAAAGTTGGAACTGGCGATGTTCT
CTTGGGCATGGATGGAGAAAGCGGACTAAGCTGCTGGTTTGGAGATATTGAGCCTATCAATGTTACTTC
AGCTCTGGTTTCAGGGTGCATGTGGTTATGTTCTCCTTCATATGTAGCCACCAACACACATGGATTTTCC
GCACTTTTTTGCACCTGAAAATAAAAACGTTAACAAATATATCAATATTATTTCTTCTTTATGAGAGGTG
AGGAGGAAAGGGAAAAAAAAAGGGTAAGTACCTTCTTTTTAACTTGGCAACTTGGGGCAAAGGAGCA
CTTGTAGTAAGCCCTAGGAGATGGGTTATCTCTCGTGACCTTTTGACCATATTTTCTCCATTGATATCCAT
CCTTCACAATCTGAAATAAATTTAAATCCATGAAATTAATATTTTGGACAACTTTTCAGCATCAAATCTC
AAGCAATGCATTGGGTTAATAATTAGGGCTTACCAGGCGTGTATCAGAGGCTTCAGTGCGTACATAAAC
CCTAGAAATCTTCAAGTTCATGTTCTCCTTCGGCCTCTTACATCCGTACTCTTCATCACTGATGGAGCTG
GTCTCAGTACTAGTTAATCCAATCATGTTGCTGTAGTCTTCGCTGTCCGGTTTTCGCTTCTTGTTGAAGT
TTGTAGCCAAATCGTTTTGATCAGAACTTTGCTGCTTGGTGATCATCAACTCCTTCACATGGGATTGCA
AATTGGTGTAGTTTTCACAGAGAGAGGCAAGCATCTCAGTCAGCTTCTTGTTCTCCAAACTTATCCTAT
TCAACTCTTCAGTCAATACATGGCTAGCTGCTGCCTGCTTCAACATATCCATCAAATCAAAGAAGACGC
TAATCTTATTAGTTTCTTAATTAAATAGTTAAGCAAAAGAGATTAATTAGTGGCTTTGATTTCTAGCTACG
AGGGTTTGAAGTAGAAATGAACGTTCAAAAGCATTAGCTCACCTCTTGTTTCACCGATGCATGGCCTTC
AAATACTTTAGTATAATCCCCTTCAAGCTCTAGTTGCTTCTGATAATAAAAAAAAGTAGATTTGAATATT
AATTAGAGGTTGGAATTAGAACTTAATTAATTAGATAGCAAGAGAGCTCAAACCAATTATAAGGCAGCA
GAAACAAATCTCACCTTCACAGGAGCTTGATCATAACTAGCAGGGTGGAAAGGAATAACATTGAGGCC
GAGAGAAGTGTTCACCCATTCTGAGTCCAT

>PmWRKY19

TCATCGATTACGCATCCCAGGAGGTACAATGTCCCCAAGAAGCCCTTCATCTGCAGGAACTGAAGTTG
TCTGTGGATTAAGGCAGTGGGATGATCCTGCTCCTGCAGCTTGATCATCATCATCATCATCATCACCACG
TGGGGATGCTAATTGGTGCGAAGTCGTTCGTGTCAAGTTAACAGGACTAACTCGCTCTCTGGGTTGAA
TTGCAGGATAATACATGAAGTGTGAGCTGACTGGAGGCAGCTGCGCCAAATGGTGGCCTGCAAAGCC
AGATTCTTGAGCAATCACTCCGCCCCGGGGGAATGCAACAGTGTGATGACAGTGTTGGCCTTCGTACG
TGGTAATTACAATTGTGGGATCTTCAGAGGAACGTTCAACCCTTTTCTTCACTGTGCATTTGCTATTTGT
GCAGCGATAGTAGCTCCTGCAAAACACACGCTTCACTTGAGAGTGTAACAAAGAAATTAAACGGTTC
GGATTATGAAATGTAATTTCGTAAAATCATTGGAGGACAACGTTATTCTCACTCTCCTCAATTTATCATAT
TCAATATATATATATGCGATCCCTGTGTTTTCAAACCAACAAATTGCCTTGAAACCTACAGAATTACTTG
AAATGAATTCAATACTAAAAAAAAAATTGTAAAGTTGGTCTCCAGATAAGTTAATTCAAAAGTTGCAGA
CCTTGGAAAGGGGCTGTTTTTGACAGCTTTCTGTCCATATTTCCGCCAGCGGTAGCCATCCTCAAGATG
ATCAACTTCACTCTTAGTCATAAATGCAAATCGTGGCTGCCGGATTCTCTTCTGCCCCTTCTTTTTAACC
TTACTCCTACAAAATATTTTACCTACAATTATCACAATAAAAACAATATCTTGAAAATCCAAAAGGAAA
AAGCAAAACTTAGTGAATGAATTCATGTCCTATACATATATACATATATATATATATATTTCAAAACACACA
GGAATGCTAAAATTACTTATAACAATAAGTCATGTAAAATTTTATCAGTTCTAACAAGAAATAAATTAAA
ATCCGTGCATTCTATGAATTTTACATAGAAATCTAAAGTGAATGTGCATTGTAATTCTTGTTCATTAGAGT

TTTTCGAAACGAAACCGTAAATGATTTTGCTTATGTTATAGCTCTTACTTCACATGAGGAGGCATGCAG
AGTGTAGAACCATGCAGTGTAGGCTCTCTAAGATCATCAAAATGATATGCTACATGTAATTATTAGCCAC
CATTATTAGGAAGAACTGTAACAATAAAATGGACCACAAATGAAGAAGAATCCCATCCAAGAATATTAT
GATAAAATCACACCACTAAGCATTGTATTAAATAGGACACTAAGCAACTTGGAGACAGATATGTCGCAT
GCATTCATCACATAATTTATGACGCAAATGCTAGGACACTACAGCAAGAACAAAGATTTTAAAAATAAA
AAATAATTATTTAAAAGGCTGAGTATCTTCGTTCTAAACATTTTAGTAATTTACGAGAACTAATATTTATT
TTAAAAAAAAGACACAGAATTATTTTACCCAAAAGCGTGAAAAGTTATGGCACGCCATTCCCGCCGTA
ATAACGGCAGTGTTCGAGTGCACAAAAACTGCAGCCCTTTAATTTCGCTCAATTCCCTTCTCGTGTTGA
AAATTACGAAAATGCCACCCCAACTTGCATGAATATTATGCTGTTAGTAATATTCTGTTGTGTGTTTCTG
TTTTCTGTGTGCGAGGAATTTTCTACTTTCTAGACCGTACGGACGCAGATTTTTGCAGACTGCACCCAA
ACTCTCTCACTCCCTCCCTCACTCTCTCTCTTTTTCATTTTCGAATTCAATTATTGATTGGTTTTGTAAAG
AGATACCTGGCACAGCTTAGCCCACGGAAAAACAAGTTAATTACGCATTTAAGCCATTAATCATTGAAA
CCCACTCACGGTATCTCGGGCGGTGGTTTGCCGCCGGACCCCGTAGATTTCTCCGGCGGATCCTCGCTG
GAGGTCGAAGAACCGGACGGGTTCGTCGACGCGGATCCGACTGCGGGGGCCGGGTCGGAGCTCCGA
AACGCCGCGGTGCTGCTGCTGCTGCTGTCGGCTGGTCGTAAGCAGTCCGCAACAAGCTGGCTACTAGT
TGCCGCCAAATCGGACATGTCGCGGGTTCCGATCGGGTCAAGTTCTGAAAAGCCGTCGGGTCTGGAG
CCGTCCGGGTGGAAGTTCCAGCCGAATTCGGTGAGGAGTGTGTTGTCTCTGACGTCGTGGCTGGCGA
AGAAGTAGGCGCTGTCCGGGTCGAGTGGCCAGGTGGACTGAGTTGTGAACTCGGTCGTACTAGCTGG
ATCGCGGTTGTATTTGTCGTCGTCCAT
>PmWRKY20
ATGGCTGAGAACCAAGAGCAGCCACCGGGCCCACCGCCGTCCACTTCATCGTCACCCTTGAAATCAG
CTGCTCCTCCTCCTCCTCCTCCGCAGCGCCCTACGATCACCCTCCCTCCGCGCTCCTCTTTCGAGATGC
TCTTCAACTCCGGCGGCGCCAATAGTGGGCCGGGTTTCGGCCTCGGCTTCAGCCCAGGGCCCATGACG
CTCGTCTCCAGCTTCTTGTCCGATGGCGAAGACTGCAAGTCCTTCTCTCAGCTGCTCGCAGGAGCCAT
GTCTCCGGCGGCCAGGCCGCCTGGCTTCCCTCAGCTCGAAGACCGAGGCTCCGGCGACGGCGACGAC
AACTCCGATTTCCGGTTCAAGCAGAACAGACCGTCGCCGATGTTTTCGGTCCCGTCTCCGGGCTTGCT
CGACTCGCCGGGGCTCTTCTCGCCTGGTCAGGTACTCAACTCATTTACCGTTACCTCTTCTTGCTAGCTT
ATTTGAGAGTTGGGCATTTTTGAAAAGTTTCGTTTTTTTTTTTTTTGGGTTTAGATTTTTCCGAGTTCAA
TGTCTTGTCCTTAGTTGCTGCCTAGACTGAGGAAAAAATGGTAACTTTATTTATTTATAAAGAACTGAC
GAGAAAAGAAAGTAGAAATTTTTTGATAGATTGAGTCCGTTTTAAATGGCGGCAAGTAGTTGTAATTAA
GGTACAGATTAAAGATTCTAAATTTAGTAACTTTTTTTTTTTTTTTTTTTTTTTTTTTTTTTTTTTTTTTTT
TTTTTTGCCCGGGGAAGTGGGGAATCTGGCAATGGTTTTGGGTTCTTAGAATTGATTCACTTGTTTTTTA
GGAAAATTGGATAAAGCTACTTCAAACCAATCTGCTTAGGGCTTTGTTGTTGCTTTGATACGCTACTTG
GAATCGGTTTCTTATGGTTGTGATTTTTGTTTCATGTAGGGACCCTTTGGAATGACACACCAGCAGGCC
CTAGCACAGGTCACAGCTCAGGCTGCACAATCCTCTTCCTATTTCCATATCCCAACTGAATACTCATCTT
CTTTATCGACAGTGCCCGCCACATCCTTGACACAGCTTCCAGCCTTTACTTCCGATTCAACAGCACCCC
AGGAGATGCCATCTGGAGCAGCTGACTCTGGGGTGGCTATAAAAGAATCATCTGACATCTCCCATTCTG
ATCAGAGATCCCAACCGTCTTCGTTTACTGTTGATAAGCCTAATGATGATGGATACAATTGGCGTAAATA
TGGGCAGAAGCAGGTGAAGGGCAGTGAGTTTCCTCGAAGTTATTACAAATGCACGCATTCAAATTGTC
CTGTTAAGAAGAAGGTCGAGCGCTCAATTGATGGACAAATAACTGAGATTATTTACAAGGGTGAGCAC
AACCATGAACGTCCTCAGTCCAAGCGTGCAAAGGATTCTGGAAATCCGATTGGAAACATTCAGGCGAA
TCCTGACTTAGCTTCCCAAGTTCATGGTGGGCATTTGAACAAATCTAAGAAGGGTCAGGAATCTAGCC
AAGCAACACACGATCATTTATCTGGGACTAGTGACAGTGAGGAAGTAGGAGATGCTGAAACCAGAGT
AGATGAAAAAGATGAAGATCAACCTGACCCAAAGAGACGGTAGATATTTTATGTAGATATGGATATGCA

TACTTGTGGTATAGTTAATGTTCCCTGTTTGTCTTGTATCCTTAACACCTTTCCTTTTTCCAGAAATACAG
AGGTTAGGGCAGAGCCAGCTTCTTCACATCGGACTCTGACAGAGCCTAGAATCGTAGTGCAGACAAC
CAGTGAAGTCGATCTTTTAGACGATGGCTATAGGTGGCGCAAGTATGGGCAGAAAGTCGTCAAAGGCA
ACCCTTATCCCCGGTAATACTGCTTATCCTAATTTCTAAGCTTAGTCATATTTACTGTTCGAATATAATAGA
CTTTTTCAAGGAGGTTTGGTGCTGATCTTTAGTTTGATTTTGAGGACGGAACTAGATTTTCTTATGTGAC
TACAAAACTAACGTTTTATGTCCTTCACATGACATAAAATGTTTAAAAATCCATTGCCATATAATTACTTG
TCATTAACTGTTTACAAATCTAATAGCATAGATTTAAATCCTGGAAAATGAATTTTGAGAGCTAAAAACC
TTCTAGTTTTCATTGCCTATTCTCTTTATGTTGCCTAACATTTTCAATAAAAGGAGTATATCCTTTGGTGC
ATGGTTGGTTAAAAAAGTTCCTCATCTGTCATGCTGCATTCTGCAGGAGCTATTACAAATGCACGTTCC
CTGCCTGCAATGTCCGTAAGCATGTAGAGAGAGCCTCGACAGATCCTAAAGCTGTCATAACAACGTAT
GAGGGAAAACATAATCATGATGTACCAGCTTCTAAAACTGGCAGCCACTACGCAGCCAACAACAATGC
CTCAA
>PmWRKY21
TCACGAAGAT
TTGTTCAAGTAGCTTGATGCGCACCCTATTCCATTTGGGCTTGGTGGGTAGATTGGATTTGTTGTCAAA
GATTCAGCCCACTTCAATTTCAGCCCAGATGCACTTTCTGTAGAAGTGCTACTTTGATGCTGATGATGAT
GATGATTTTCTCTGACCCCAGTAGTAGCACTACCCCCATTATTAGTACCAACAAACGATGTGAGTGCAG
CTGCTAATGCTGATTGGAATTTAGGGTTTGATGTGATTGCTTTTGTTGCAGAGGCTATTGTCTCTGTTAG
CATCAACTGCTGATGAGGAGGGGGAGGAAGAGGAGGAGGAGAAGGAGGAGGAGGAGGAGGAGGAG
GCTTCTGATTTTGTATGTAAGATTGGTACAAATTATTAGGTTCTTGAAATATTGATTGCTTTCCCATGCTT
AGAGCTGACCCAACTTGGTTAAGTACTCTATTCCCGTAGTTCAAATAGCCAGCTGCTGTGTGATTATTG
TTGTTCCAAGGTGCTTGCAACTGCAATGTGTGGTTGTTGTGGTCTAAAGAAGAGGAGGGAGATGAGG
AAAAATTGAGACATGTGGAGGGATATCTTGGGTTGGAAGAGAAGACAGCTGCAGGAAATCTTCCAAA
GTGAGAGGGAGATGGAGCAGTAAGATCAAGAGTGATGGTTGGGTGGGAATTTGTGGTTGAGATTGAA
CTGTTGGGGAAATAGAAGTGTTGTGGTAGTCTTGAATTTTGGGAGAGAGTACTTGTACTGAAGTTTAG
TCCTTGGAGGTTTGTGGAGGCAGAGATGGGTGCCGTGGCGGTGTGGCCTTGTTGGGAGGTGGAGGAG
TGAGACAGAAGCATGGAAGCCGCGGCGGAGGTGGTGGAGGCCATGGCTGTGGCTGACATAGGAAGT
GGATGGTTGTGGCTTCCTTCGTAGGTTGTGATTAGTATGGACATGTCATCAGCACATCTTTGCACCTGA
AAGTTGAACAATAACAAATTTCATATCCTAACCATCCAAGCAATGCTATTTAATAGTTTCTTTTTCTCATG
CTTGCAATAGAATATATTCAAATAATTTGAGGATCAGAAGATATATGTATACCTGTTTTCTCACGGGGCA
GGATGGTGAGACAGTGCATCGATAGTACGCTCGAGGGCATGGATTTCCTTTTGCTATTTTCTGTCCATAT
TTTCTCCATTGGCATCCATCATTCATCTATAAAACAATATTCGATTAATCCATGATCAAGTATATATAAAAT
AGCTAGCCGTGCATGTCATGATGAATAATCATGTTGTCAAGCAACATATAAAGATTGTGAATCTAAATCA
TTGATTAAGTTAATTTTTCCACTTGAATATGTGATCTACATTTACAATCTGACAATATAATCTTAACCCGAT
AATATCAAAGGTGTTAATAAATTATTCAAGCAAGCTAGCATGCAAGTAACTTTCAGTATATTGTTGATGG
CATGCATGAAAATTATGCTTGAAAGGTTAAATTAAGTTTGATATATACTAACCGTTGGAGCATCGCATCT
AGCTCTGATAGAAACCCTAGCTTTCTTTAAATGGGTTTGTTGTGAAACCTCATCATCCCCACTTCTTGTT
GTCTTCGAAGTCTTGCCAGGTGGCCATATCTCAGTCGGGTCGTCTTCCTTGGGGTCTCCGCAGCTATTA
TCAGAGCTTGAATTCTTCATAACAACTTCAGTTGATTGATCAGCAGCTGGCTCAAATCTGCAGCCCAAC
TCCAGTGCAAGTCCAGCTCCGTTGAGCCCTTCTTCATCGTCACCTTTCCCATTTTTACTGAAATGGCTA
GTCTTTTTCATCTGTTCATCCTTTCTGGGCTGATCAATGCTTGAGCTTCTTCCAAGGCTAAGGGACACA
AGTTCATCAGCTTCTTCATCTTGTTCGTTCGACTGATCACGAGCAGAACTACTAGGATCCATAGATTTCT
TAGCTTCTTCATCTTTTTGAAGAAGGCCATGAAAATGCATCTGAAGAGACTGGTAATTCCTCACGATTC
GCGATAGTAACAGCTTTAACCTTTCATTTTCTTCTTTAACCTCCCCCATTTCAGCTTTAGCTGATTGGAG

CTCATCCTCCTGGTGACAATACAAATAAATTTTTTTGGCTTAATTTTATGAATTAACTTTTAGTGAAATCT
TACTGGAAGGGAGAACTATATATACAAGTTATGAATGATTTAGATTTAGATTTTTATTTAAAAAAATCTAA
AAAAAAAAAAAAAAGAAGCAAATGATTTAGAAGATGGTGTCGAGTTCAATAATATCATGTTGAGATATA
AAAATTTGCACATGGGAAAAAGGTGGTGTCGAGTTCAATAATATCATGTTGAGATATAAAAATTTATAC
ATGGGAAAAAAATAAAAAAATAAAAAAATATATATATATTAATTAGACCTTAATATCACTGAATGAAATC
AAGATATGGTGGTTCATACGACGACTTAAATATATATTTACTTGGTGGGCCCGTCTGCTAGATTCATGTG
AGCATCTTTTAAAGGCATAGGTTTTGATGATAAGCCAGATCTATTGTATATGAGCTGATGAGATTAAGAT
TTCATCATCCTATATGCTTAAATCAGCTATATTTGATCAAAACATATAAAGAAAAAGAAATTATTAATAAC
TCTATAGACAATACAGCCTCATGTGTGTTTCCTTCTTACTCTAAGTTTGTAATTTTCTCACCTGTGGGTG
CTTGATAAGGGCTCTATCTTTTCCTGCAGAAGAAGACGGCATCAAAGTGTTTGTTTGCTCTGGTTCAGC
AAGCCACCCACGTCCTCCCTGAATTGTGATATCCATGGATGAGGAAGACTAAGATCAAAACAAGACGA
TATATACGTAAAATACGAGAATATATAGTACTCTTAAATCAGAAAGAAATTTATGATGATGAATGGAATA
AGAGAACACTAAAAAGAGTGAAAACATCTCATGTATATACATCAATAAACAGTGAACAATCTCATCAA
GTTTCAAGTTTCAACGACATTAATTTAAATAAGTGGCATGCAAATACACATGAAATTCACCTTGCTTATT
TCCATCTCCATGGAAGTACCCTCTTCATCATGACCATCAGATTTCTTAGTTTTGTCTTCCACTGGTGTATT
TCCAAGGAAAGAATTTTCCTCAGTATTGTTGTTCTCCATTGCTTTTGAACGCCTCTTCGGCATTTCCAT
>PmWRKY22
TTAAATTGAAGTGTAGATTTGCATCTGGCTCAAAATATGCTCAAAGTTATCGGTAGATTTCTCGATGGGA
TGAGAGTGCATGCCTTCATAAGTTGTCACAACAATGCCTTCGTCTTTGGTTAGCCTCTGAACTTGCTTC
TTCACATTGCACCCCTGATGCGTGCACCGGTAGTAGCTTCTGTATATAATAAACAAACTCACAAGTTAA
CTCAATATGCATGGTCGTATGTCACCATAGATAACTAACTTAAATGATATATTTTTTCTATGTAGTGCACT
AGAAAAGGGTCTTACGAAAAAAGGTCTTAATTTGTGGACCCGTGATTTGAATCCCTATGCAGTGACGG
AGCCAGAAATTTAAGTGAGGGGCACATTTAAAAATTGAAACTCTTTTCACTTTCTCTATTGTTACTTTTT
GTATAAATAATCTAATATTCATTTATAATAACAAAACATATGCAACTTAAAAGAAATTTAGACACTATAAT
AAAAGAGAAAACAAAAAATAGGGGGAAATTTAAGAATTTAAGAGGAATTTCATAATATTTGTAACAAT
ATTTGAGGAGGGGCACACGCTCGCATGCATTGGCTCCGCCACTGTCCCTACGACACCTTTTGTAATTTA
GACTATCACTTGTATTCAAAAAATTATTATTTTTTCCTATGCATGTTTGAATTAATGTACTAACTACTAATA
TATTTAATCTAATTAACTTGCACTAGTTCTAGAACAGTTGAAGAATCAGTATTCTGTCAACGATTAATCT
TCCCATGCTGCTTGAATATTAATAATTGTACACATTGAGAATCCAATATCCCATTTCGCTGCATGTATATT
GTAGAACTTCATAATTAATATATCAGAAACATATGAACTAGAATATTATAAAATGGAGCAGATATACAAG
GACAGACTAGTTCTTTCCCCTGATAAATGTCTCAATTTGTTAATTATACTTCTTTTGAATTACAACAAATA
TCAAATGTTGCTCGGCAACCTCATGACCAATATGTCATTCTTGACTATGAAAACTCCATATTAATAAGCT
GCTTAACTTTCATTACTTGAGCGATTGTTCTAAATTTTTTAGGTTTTCAAGTGTGAGATTGATGTCTCCG
TAAAGAAGAGTGACTGGTTTGCATTTTCAGCCACCACAGAAAAGTATAATCAGATTTGCAACCATGATT
AGTTCAACATGAAATAATAAACCTAATTCTTAATTATTTTTTTATACAGGCTATATTCTACTCTAAACTAAT
CTAAACAACGGGAAGGGAGATTTGAATTTGAGTGCAGAGTGGGGAGCATATCTGCTTGTAGAGGAATT
TTTAACATATTTTTCAGAAAAGCATAACCTAAGTTTTACATATTTTGTATGCAGAGGTCGAATAAGCCTA
GAATGCTTGATGTAACGCATTTATATGAAATAATTGATCACGGTCGACTTATAGATCGGTGCCATAGAAC
TAACTATAAGGAGCTACACATACCTTGGAAACTTGTTGTTCTTCACCGCTTTTTGACCATACTTCCTCCA
TCGATATCCATCATCAAGGATATCAACTTGGCTCCTCGTTTGAAAAGCATATCTGGGTTTTCTTATCTTCT
TCTCCCCCTTCTTCGTCCCTAACCTCACAGCACCTTCAGGCCCCCCAAAGCTTTTGCTCTGAGAAGAAT
TAATATTGTTTGAAACCTCCATATCTGACATCAGCCCCAAGAACCCATTTGCTGCTGGTGGTGACTTGTT
ATTTTGGAATTGAAGATGATCAGTAGTACTGTAAGCGCCCTGAGGGTTCCCCAT
>PmWRKY23

ATGGATGGTAGAGAAACAGGAGAGCTAAAGACGACCATCGATCAAATTAGCATGGCGAATTCCACCGT
TTTTTCGGATGAGATTATTCCAGGCAGCAGCTTTGCTTCTTTTGCATCATCAGGCGGCAACATATTTGAC
ATGTCTGATAATAATCAGAGAGGCTCTGGGTTCATGGACTTGCTTGGATTTCAAGACTTCAACATGCCC
CCTTCTTTATTCGATTTCTCCTCTTCCCAAACGACGTCGTCCTCCTCGTCGATGATGATGGTGATGATGC
CACCTCACCACCAACAGCAACAACAACCACCACTTGCCTCTCCGGCCTCCACCCCTACTGCAGTTGCT
CTGCCGGAGAGGGAGAGTACGACGTCGGAGGTGCTGAACGGTAGTACGGCTCCAACGACCCCCAACT
CCTCTTCAATCTCTTCGTCGTCCAATGAAGCTGCTGCCAATAAGAATGAGGACCAGACGACGACCAAG
GCTCAGGATGAGGAAGCTGATGAGCAGAACCAAGATCCCGAAAAAACCCAGAAACAGTAAGAGTAG
CTAAGGTTTTAAGTTGTTATGTCATTTTTTGTCCTTTTTTTGGTTTTATGAAAATCAAATGGATGAATCAA
CTTTTTTGTGTGATGTTGTGTGTGAACTAATTAAGCATATGGGAAGATGATTTTCTCTTCTTTTTTGATGG
GGTCAGTTCATGTTGTTGTTTCCTTTTCTGATTTTGATGAATTTTGTTTTTGTTGATGAGATGGAGAAAG
TAGTAACAAGTTGCGTTTGCTTTTTGTGTGATTGAATCACAGATTGAAGCCCAAAAAGAAGAACCAAA
AAAGGCAAAGAGAGCCGAGATTTGCGTTCATGACCAAGAGTGAGGTTGATAACCTAGATGATGGCTAC
CGATGGCGCAAGTACGGCCAAAAAGCTGTCAAAAACAGCCCTTATCCCAGGTCAATATCCATACACTC
CCAAACCCCATCTTCATTCCCATCCTCCTATTCATATTCAATCAATCATTCATCTACTTATAATAAATACAA
AACTTGTATATCGATGCAAATTTTCTTCGTTAATTTCTTATGGGGCACGCAATAATTATAATTAAAAATGT
TAATATCTATATGTTCATGTGTAACAGGAGCTATTATCGTTGCACCACTGCGGCCTGTGGTGTGAAGAAG
AGGGTGGAGAGGTCCTCCGACGACCCTTCCACTGTGGTCACAACATACGAAGGCCAGCACACCCATC
CAAGCCCCATTACGCCACGTGGCACCATGGGGATTGCACCGTTGCCTGACCAGCCTTGTGCCTTCCCC
TCATCTCCTTTTGGTGTTCAACAGTTATTACCTCATCATCACTATCAACAACAACAACAACAGCAGCAG
CAGCCGCAGTATTCGTATATTTATAGCTCAGCCCCTTCTTTGAATATTAGCTCACCTGCTTATGGGGGTG
GTGCTTTTAACCCCTCCTCATTTTCTACGGGTTTACTTCAAGAGAGATATAATTTTGGATCACCTTCTTCT
TCTTCCTCTGCGGCTTCTAATTTGCTTAGAGACCATGGACTTCTTCAGGACATCGTGCCACAGATTCGA
AAGGAGGCCAAAGAAGAAGATCATCTTCATCAATAG
>PmWRKY24
TTAATGTCCTGGGAAGCCGCTCATTTTGCTGTTGCTGTTGTTGTTGCCTCCATTGCTGTTGTTGGAGGTC
GTCGAGGTGCTGTTGTTGTTGTTGTTGTTGTTGTTCGGATGACCTCCACCGATGATGGAGGTGATGGCG
GCCGCAAGAGCAGCAGTGAAAGTTGGGTCGGCAGTTATGGCAGCTGTAGCGGCACTCACTGTGTCAG
CAAACGAGGCAGACTGAGTTTGATGCGGCAAATGTTGCTGCTGCTGTTGGGAATTAGACCCCATTAAA
TCCTGGGACAGCTGAAGCCCAGAAAACTTGGATTGGTTATAAAGGGCCTGTCCAAAAACTTGAGGCA
GTTGTGGCTGTTGCCCTGGGAAAGGACTTGAAATTGGGACTGTGGNNNNNNNNNNNNNNNNNNNNNN
NNNNNNNNNNNNNNNNNNNNNNNNNNNNNNNNNNNNNNNNNNNNNNNNNNNNNNNNNNNNNNNN
NNNNNNNNNNNNNNNNNNNNNNNNNNNNNNNNNNNNNNNNNNNNNNNNNNNNNNNNNNNNNNNN
NNNNNNNNNNNNNNNNNNNNNNNNNNNNNNNNNNNNNNNNNNNNNNNNNNNNNNNNNNNNNNNN
NNNNNNNNNNNNNNNNNNNNNNNNNNNNNNNNNNNNNNNNNNNNNNNNNNNNNNNNNNNNNNNN
NNNNNNNNNNNNNNNNNNNNNNNNNNNNNNNNNNNNNNNNNNNNNNNNNNNNNNNNNNNNNNNN
NNNNNNNNNNNNNNNNNNNNNNNNNNNNNNNNNNNNNNNNNNNNNNNNNNNNNNNNNNNNNNNN
NNNNNNNNNNNNNNNNNNNNNNNNNNNNNNNNNNNNNNNNNNNNNNNNNNNNNNNNNNNNNNNN
NNNNNNNNNNNNNNNNNNNNNNNNNNNNNNNNNNNNNNNNNNTTGTTGCCACACTTGATGAGCAGG
GAAGAATTGCTCTGGCTAGCAAATTGTTTGGGTTCATTATCCCATCTGCGCTTGACATGGAGCCCGAAA
GCAACATGCTTGCTGCTGCTGTTGTGGTTGATGCCATGGCCATTGCAGCTGGAGGCAGAGGGTGGTTA
TGGTTGCCTTCATATGTTGTGATCAATATGGTTCTGTCCTCAGCACAACGTTGTACCTATCAAATAATTA
ACATATATATATATATGTATCAGTACTTTTAAATCTAACCAGTTTTAATTAAATTTTCAAATTGATCATATCA
TTAAATTGAAACATACTTGTTTGCGAACAGGACAACCAACAGCCATGGTACACCTATAATATGCACGAG

GACATGGGTTTCCTTTTGCCATCTTTTGTCCGTATTTTCGCCATTGACATCCATCAGTAATCTGCAATCA
AACGATCAATCGCGATTAATTCATCAAAGATGGAGACTATGAATTAATTTATTGTTGCTGAGTGAAGTTG
AACCATCTTAATTAATTACTTACCATGGGAGCTTCGGATCTGGCTCGAACCGATACGCGGGCTTTCCTCA
TGGTGGCCTCGGTGGATTGATCAATTGGCTTATTAGCTGCAGAGTTGTTGAGCTTAGGAGCCTTGTTGG
GCACCCAGCCTTGTGATTCAGATTCAGGACTCTCTTCCCTTCCAACCCTTTTACCATCCCTGAAATTGTT
AGAGTTTTCGGGATCCAATGGACCAATCGGATGATCATTTTTCTTCAGGTGATGATCTTTTGATGATGCA
GCTTCATTAATGTTCTGAGGTGAAGCCGATCGGGTTCTGGCTTCTGATGAAGAATTGGAAACTTGGTCA
TCGGTCTCGGCTGTGGCTCTGGGGCCCAAGTCTAGAAACTGCCTTGGCACCAACCCTTGTTTCTTCTC
TTGATCAGCCTTTGCTTCAACATTCTGAGAATTAATTATACCAACAACAAATAATAATATGTTAAGCTATT
TGGCTCATATAGTTAATCGAGTTTTGATCATTAAGGCAAACCATATTGAATTATAGGTACCTGATCATGTT
TTAATTGAGAGCTTTGATCAGCCGCGGCTGTGTGATTTTGCTGCTGCTGCTGTTGTTGCATTACAGCCG
CAACATGCATCTGCAGAGCACTGTAGTTGTTGGTCACCTGACCAAGCATTTCTTTCAGCCTCAGATTTT
CAGAATTCATACGTTGGAGCTCCACTTGCAACTGTGCCAGCTGATTATGATGATCAACCCAAAATTTTAT
ATTATTAATTAATTAAGTGGGTAATACTAATAATAACATCCTTAAATAGCATAATTAGCAAGCAACTAATA
GAAAGTTGATCAACTTTAGTCTAAGAATAGGAAAATTCTGATCATGAATTTGCTTTCAGGTGGGTGTTC
ATCTCATTAATTACCTCATGATTCTTTGCTCTTTTGTTATCCACATCGGATGAAATCCCATCATCAACCAT
TGACTGATCACTTCCGGTGTTAGCAGTAACAAGGTGCAGGCCAGTCTATTTTTAGATTTTTTTATAAAA
AAAGCAACAACAAGAAGAAAAATAAGTACCACTGAAAATTAAATATCTGTTACATGCAAGTAGTAGTA
GTATATATATATTAATGAGAACGAATTATTTTCCATATATAACAAATAAGAAATAAATTACCAAAAAGAAA
ACTCAAAATTGAAATTGTGTGGGGTGCTTACATTTACATCCAAGCCAGTCCAATTCTCCTTCTTGACGC
TGATTGTACCTTTGGATTTCATATCCTGATGATGATCATCAGTGGTGGTTGTATTGTGCTTATTCTTACGA
TCAGAAAAGAAGTCGACCTCATCCACAACCACACGATTATTGTTGTCATGAATGGATGGCTGTGGAGC
AGCCAGCTGGTCTTCCCTGCCGCCAAGTTTGACGGGGAATTCAATACCCGGAAACATCCTTTCTCCAC
CAAAAAAATTGCTTATCTTGCTGTTGTTTTGATGATGATCGAGCTTAACGGCAGCCGGAGGCTTGTTGA
GGAAGAAGCCGAAGGAGTCAGAATCAAGGGTGAGCCCCCATCCTTTGTCCAT
>PmWRKY25
ATGGACTCTACATTCTTCAACAGCAGCAACAGCAACAGCAACAGCAGAAGCAAGCAGTTTATGAGCG
ATCAAGAAGAGAACGACAATACTACCAGCTCCACACCAGAAAATAGCTCAGGCTCTCCTCCTCCACCT
TCTACCAATTTCAGTGACTTCTCCAAGATCACTTCCACCTCCTCCCCCAAGAAAAGGTCTCAACTTTAT
CCTCAATTCACCAAATTAATTCGACCCAGTGTTCAATAATGGGAATTAATTATTTATTCAATTAATATTTA
CGTTAATTCAATTGTTACAGTGCTCGGCGAGCCATACAGAAACGAGTGGTGTCGATACCCATCAAAGG
CGACAACAGCAACACTCCGCCTCCATCCGATTCTTGGGCTTGGAGAAAGTACGGCCAAAAACCCATC
AAGGGCTCGCCTTACCCCAGGTAATTTGATCATCAAATCCATAACCCAATTCGAATTTCAACCCAAGCT
CCAATTTTGCAGAGCTGGTGTTTTTGATCAGTGACTAATTTTGTGCTCTGTTTTTTGTTTTCCTTTTTTGT
TTCTGGGATTTGGTTAATTTACCAAATTGGGCAGAGGATATTACAGGTGCAGTAGCTCAAAGGGCTGCC
CGGCAAGGAAGCAAGTAGAGAGAAGCCGCGTGGACCCCTCCATGCTCGTCATCACCTACTCCTCCGA
ACACAACCACCCCTGGCCCGCTTCTCGCAACCACCACAACCATCACCAGAGCAACAGCTCGAGCTCC
GCCGCAGCCGCCGCCACCACAACCAAACCCGGCTCCAACAAGACCGAAGCCCCGGAGGCCCAGCAG
CCCGAGCACCAAGACCACGACCCGACATTCGCGGATCTCAACGACGAGTCGCTCCTCACACACGACG
AGTTCGGCTGGTTCGCCGACATGGAAACGACGTCGTCGACGGTGCTCGAGAGCCCAATTTTCGCCGA
GAGCGGCTGCGCTGGTGGGGCCGACTCCGCTGACATGGCAGCGATGGTTTTCCCGATGGGGGAAGAG
GACGAGTCTCTCTTCGCTGATCTGGGTGAGCTGCCGGAGTGCTCTCTCGTTTTTCGGCACCGGGGAGT
GGGACCACAAGTTCAGATCTGTTGA
>PmWRKY26

TCATGGCTCTTGTTTGAGGAACATGGAGGGAAACAACAAGTCCTGCAGAAGTCCATGCTGATCAGCAC
GAGGTAGTTGATGCTGCTGCTGATGATGAGGATGATGAGGGTTAACAACAACACTTCTTGATGACCCTT
GATTGTTATTATTGATCATGTAGTGGTGGGGCATTTGAAACAAGAGTTCTTGAGGAAAGTTGGAGCTTG
GACCTGCAGATCCTGTGATGGGCGCATAGTTGAAGGCAGCAGCAGCATGTGGGAAGAATGCAGAAGA
AGCAGCAGCATTTCCTCTGAGGGTTGCAGGGAGTGGGTGGTTGTGTTGGCCTTCATACGTCGTAATCA
CAGTGGATGGGTCCTCGAACGACCTCTCTACACGTTTCTTGACCCCGCATTTTTGGGTCGTACATCGAT
AGTAGCTTCTGCAATGCCCAACAACAACAACAACAATTATAAAGAGAGTCGTGACCAAATCAAACTTT
GTATTTTTTTTCTTTTTTCTCTTCTGGGTATGAATCAAACGGGGCTAAGCTCCTAAAACTCACACTTGGC
CAACCATGAACATAATATATAGAAGATCTTATGCCAAAGTCAAGCCTTTTGGTTTTAGAAATCAGTTCGT
ATGTCTTTTTGCAGGACTTTTTGCACAAAATTTGGTGAGGTTAAAAGAAAAATATTAGTGAAAACAAAT
CAAGTTTTTAAACCTTGTTCACATTTAGTTTCCGGTTTGAGTGACTATTTCTTGGTGGGTTATTTTGAGT
GGAAACACAGAAATGAACCCATTTTGTATATCAATCCAAATCAATGAGTTAATAGTACCTTGGATATGG
ACTATTTTTCACAGCCTTCTGCCCATATTTTCTCCATCTGTATCCATCTTCTAGATGATCAACCTCACTCT
TGGTCATGAAGGCAAATCGTGGCTCTCTTTGCTTTTTCTCTCCTTTCTTCTTTGCTTTGCTCATCCTGTT
GATTTGATTGATTATACAACATCAAACATGTTCATCAGATTCATCACCATTTAGATCAAGCATATATACAC
ATATTTGAATACACTATGAAAGTATTCAATTCAAACTTACACTTTCTTCGAGCTATCTCCTCCATCTTCTG
ATGACCCTTTTGGCTGCCTGTCTTTCTTACTCTTACCAGAGTCCTCTTCGGCACCGGCCTCAGCTGAAG
AAGACGACACCGAAGAGTTTGGTGTCAAAGGAGTTTCTCCACCTCCACCATCGCCATCGCCACCACC
ACCTGAATATCGAATATGCTCAGCAGGCTTTTGATCTCCTTCATTAGCGGAGGAAAATATAACTTCATCA
GACGTTGAAGAAGCCAGGCCAAAAGCAGTTGCGAGCGTATTGTAATCCATAGGTCCTTTCAAACACTC
TGCGAAGCTGGTGTTGTTCATGTATGAAGGAACTTCAATCCCTTGCAAGTTTTGGTTGTGATTGATGCT
TATCATCCCACCATTGAGGTGTTCATGATCTTCATACTGAAATAGGTCGTGGTAGTAAAGGTCTTTATGT
TCATGATCAGACAT

>PmWRKY27

TTATAAGCGCCTCTTAGCTGCTTCACCAAATGAGGTGGCGTTGTTGTTGTTGTTGTTGTTGGTGTTTTGA
TCCCCAGACCAATCAAAGAGATTGAATGGATCCAAGGCTGCAGCCTTGCTTCCCTTTTGCTGCTCATCT
GCTGTGAAACATTGTGAAAACAAGAGATTTAGTGGATCAGCCTCAATTTCTCCTAAATCAGCAAAGAA
GTCCTCATTGTGATGAGACTGGTTGGACTGATCATCAGGCATTGATGGTCTATACGGAAACCCTGCTTG
ATGATCACCGTTGAATTGATGATGATCAAGCTTCAATTGCTTCTCAATGTCCTCAAATTCTTCTTTCACT
GTTGAAGCACTCCCAGCAGTGATAACTGGAGACACATGATTATTATCATTCATGGTCTCATTTTGTTCTT
CCTTTGTAGGGCTTGCTGGCTTCTGATGAGGCTGATGAGAACTATTGGAAGAGGTCTTCGAACCGGCA
CCGTTTTTGGATGGCTGGGATCTCGTTGAGCCAGCTAGAGCATTTCTCTGAGTTGGCCAGGCATGGTTG
TGTTCAGAAGTGTAAGTGATGACCAACATGTTTGGATCAGTCCGGCTACGCTCTACTTGTTTCCTTGCT
GAGCAACCCTTTGAGCTGCTGCATCGATAGTAACCCCTGCAAGTAGTATCAATTATTGATTAATTAAGTT
TGAAACCATGTTCAATAGCATTGATAATAGAGTATGATGACGATTATAATGATGATAAAATCCTTCATTAT
ATCTTTGCTTTCATCATAAATCACTGGTAATAGTTAAATTAAAAGTATTTTACGATACATACGATGTAAAA
GTGTTACGAATGCGTGCGTACGAGCATACTATTCTTTTCCACGCACGCATTTTCTAGACACACAAACTC
CTCATATACGAGAGACACTCAAATTAAAATAAAGATATGTGTCTTCATATCCTCGCTAGTTGAGCTAGCT
ACACTCATAATGAATTAACTCACTCATCTGCATTGAATACTGCATTGAATGCGAAAAGATATATGAAGAA
AGAAAAGGGAACACAACCCCTCATGGTTGTCAGTGAGATATGCCTTAGCCTTAGTTTTACTTAGGAAC
TTTCTATTATTTGACAAACGGAGACAAAGAAGTAAAACTTACTGACCTTGGATAAGGTGAACCTTTGAT
GGGTTTCTGACCGTACTTTCTCCATGCCCACAGATCAGATGGAACTACTTCTCCAGTTGGCCTGCTGTT
TGCCGCCGCAGGCGCCGGAATGCACACAACCTTCTTTGCCTGGCTCTTCCTGCTCATATGATTAGATTA
AGATAATTAATATGTTTATGTTTTAATTAAGCTAAGCGGTCTAGCTCTACACACACACACACACACATAT

ACATGCATGGTTTGGACTTGACATTTAGATTAAGCTTTTTCTTTGAAATTAAACTGTTTTGGATTGCTTT
CTTTAGGAAGCAGGAAGAGATGGATTGTATATTCCAAAGGAAGGCTAATATCAATCAGAACAAGAGAG
GTCCCTTCAGGTGGGTTGGGGAAGAAAAGTAGTTTCCAGATATAAAAAAAGAAAGGAAAAGACGAA
AGCTAAGTGTGTGATTGTTGTTTTGACACAAAGCAAAGAACCCCACCTTTCCATGCTTTGATTAAAACC
CACAAAGATATCATGTTTAGGTAGCTATAGAGCTAGTAATGTTGACAATACTTAACAATCTCACCTGAAA
CAACAGTCATATAGAGAGCCTACTAGCTCTTCAAGACTTTAGAGCTTAGTCAAACCAAAAAATATACTT
TAAATAACAAAACCCTATCTTCTAGATCTTCACTTCACCATGTTTCAATCAAAACACTAGTTGCAATAAA
AGGAGAAGAAAAAACCCACTTCTCATTAAGGTGGTTAATCTATATGATGATGATGATGATGATGATGAT
AATGTAAGTACTAATGAGGATTAATTATTAAACATATAATTAAGTGGTAGAGTAATAATTAATTCATGGGA
AACATGGTCAAGTTGTAAGAAAAATATTAAATGCTCTCTCTCTCACCTTCGCTTGATGCCCGGATTCCG
TGGAGATGAGATCTGCACCCCGGCGTTCTCGAGCAAGCAACCTTTTCCTGATGAGCTGTTCCCATTAAT
AATCATGTCGCTTCCAACCATGGCACCAGATATCGAACCAGACACCGCCTTACTAATTCCCCGTGGAGA
CGACACTGCTGTTGCTGCACCCAGCAACGGCGAGTCACATGCCGTCACAGGGACCTTTGCACTCGGA
GAGATCTGAAGCATCCGCGAGAATATGTTGCAAGGCCTCTTTATTGGATCCCCATCAACAACACCACCT
TGTTGATGATGATGAAGATGGTGGTGATGAGGTGGAGGAGGAGGAGGAGCAAGAACTATATGTGCAC
CGCCAAAAGTGCTACTTGTACCGCCAGCTTCATCCACAGAAGTGATGTTAATATTCATATCTGATGAGG
AATTGGGGCTGCTGAAAAAGCTGGAGCTGGACGATATGTCCAGTTCATGAAGAAGTGGATCGCGCATG
TAGGATGAAAAGGGATCGCCAAAATTGTCCTTGGAATCTTCTTCTTCCTCCTCCTCCATGACTGAGGAC
GCTGAGGAAAATTTCATGGGAGGAGGATGGTCAGATGGGAAGTGCCAGCTGTCGGCGGCGGCTTCAG
GTTCTTGATGATGATGATGAAGAAGATGATCAGAATGGTAAGCTCCTGCGGCGGAAGTAGCAGCACTG
CTAGCTCTAAGAATATCAGTTAAATCACCTTGGTAATTCTCCAT
>PmWRKY28
ATGGAATCTGCAGCTAGTGATCATTCGATTCACAACTGCAGATCAATCGAGCAGCCTCTACTTTCTCCC
GAATTCACAGCCGTTGATCACGATCATCACGGGAAGCGGGTTGTTGATGAGTTGGACTTCTTTGCTGAT
AACAAGGGTCGTCTGATGGAAATGAGAGATCAGACGGTTGAGGTTAAAGAAGAAGGCGCACATGATC
ATCACGGCGTTGGTCAAGAAAAGCAACTACCAGACGTAAATGTATGAAAAGTTCTAGTCGTATAGTTC
GTCCAATATATTTACCGAGTCTTTTTTTTTTTTCCTTTGAATTTCTTACACCACCTGAATCAGTGCTAATT
GTTTTTCTTTTTTGTCATGGTTTTGATTATTATAGACTGGTTTAAATCTTCTCACAACATACACCAGTAGC
GACAAATCATCCATGGATGATGGAACTTCATCGTCCCATAATATGGAAGACAAGCATAGAACAAATGAG
GTAGGCACGTATATATAAATGGAATATTTGGACAGTTTTTTTCTTTTTTTTCTTTCTGGGACTAGTGGTTT
AAAATAATTACAAACTATAGGAGCTTGAATTTCTATTAATTTTGTTGGTTCATTAATTTAGATGAATTAAT
ATATATTCATGTGAGAGATTAATTTTGGGTTTTCACGCAGTTGGCAGTTCTTCAGGCTGAATTAGGCCGT
ATGAACGTAGAAAATCAGCGATTGAGAGTTATGATTAGCCAGGTGAACAACAATTACCAGGCATTACA
GGTGCAAATTGTGACACTGATGCAACGCCAGCAAAATCAGAAGGCTGATCATCAAACTCCAGAACAA
CACAAGGTATATATATATATTAATCTGATTCCTTGACTACATAATATATATTTCTTTAATTTCACTAATATCCGC
AACTTCATGGGGTTGCTTAGATGATCAATAATGGATCAGTCGTGGTAGAAGAGAAGCAGATGATGAAC
GGGTTCAACCATATTGTTCCAAGGCAATTTATGGATATGGGCAGGGCTGAGAAGGATGAGCGTTCACA
GTGTCCTTTGGAAGGCTGCAGATCCCAAGATTGTTCCGGGTCGCCTCCACGAAACGACATCGTTGAGT
CAATGGAGTGCTGCAAGAGTACTAGTCATGTACTTCACAGGGATCTTAGTGGAAGAATTAGTACTACTA
ATGGGGGAGAAGATAGCCCTGACCAAGAGTTTCAAGGGTGGGTTCCTAAGAAAGTCTCCAAGATGAT
GAGTCCCAGGGACGTGGATCAAGCCTCATCAGAAACTATGTCCATGATTAAGAAAGCCCGCGTTTCCG
TTCGGGCACGATCTGAAGCGTCCATGGTAAGAAACTATCCTATAGTTGAGATTTGTTTGTCAAACTGTA
TACATAGACCATTGATCGATGATGTTGATTCTCATCTGAATGTGTGATACAGATTACAATCTTCAATATTA
GTAAACGTATTATAACATTAATTGGAAAACTCTGAAAGAAATGTCTTCTTAATGAGATGGGTGTGGACA

AATTATGCAGTACATATATATTCAGAAGCAAGTTCTTTTCTGATCATTTCAAACCACACCTCAATAACCC
AAACAGAAAACAGGAAATTAAAACACACATGCTTCTGTTGGAGTATAATTCAACGACAGAGAAATATG
GATTTCATCATCCTAATATCAAATTCTCTTGCAGATATCTGATGGATGCCAATGGAGAAAGTATGGTCAA
AAAATGGCTAAAGGAAACCCATGCCCTCGAGCTTATTATCGTTGTACCATGGGAACTGGTTGCGCAGTT
CGCAAACAGGTACATACTCTCTTTCTTGTTCTTAGGTTAAAAAGTACATACCTCTTTCTTGTTTTTAGAT
TAACAGTTTGACAGCATAACATGTTAATAACTGGACCTAATTGGAATTGTAATTGACAGGTACAAAGAT
GTGCAGAAGACCGAACGATACTGGTAACGACCTACGAAGGCCACCACAACCATCCACTCCCTCCTGC
GGCAATGGCAATGGCGTCTACAACATCAGCAGCAGCATCAATGCTACTATCAGGCTCAATGCCTAGTGC
TGATGGCCTAATAAGCTCAAACAGCTTCCTGGCAAGAAGTGCCCTACCAAACTGCCCACCAAGTTTGG
CAACACTCTCAGCTTCAGCCCCATTTCCCACTGTCACATTGGACCTCACTCGAACTCCAACTTCCTCAG
AGATGCCACTTGGTCAGCCAAACCAGCTGCCTCCAAGTTTTCCTCAAAACATGATGTCTGTGCCACAA
ATTCTAGGTCAAGCCCTTTCCAGCCAATCGAAATTCTCGGTTCTCGATAGCTTTCAGGGATTGGATAGC
GCTACACACTCATTGGCTGACAAAGTCAATGCAGCAACAGCGGCCATCACAGCTGACCCTAACTTCAC
AGCGGCTCTGGTAGCAGCCATCACCTCTATCGTTGGCAATGTTCATTCAAACAATAATACCAACAACAA
TATTACAACAAGAAACAATAGTGATAGCAACACATGA
>PmWRKY29
ATGGAATCTGCAGCTAGTGATCATTCGATTCACAACTGCAGATCAATCGAGCAGCCTCTACTTTCTCCG
GAAACAGCCGTTGATCACGATCATCACGGGAAGCGGATTGTTGATGAGTTGGACTTCTTTGCTGATAA
CAAGGGTCGTCTGATGGAAATGAGAGATCAGACGGTTGAGGTTCAAGAAGAAGGCGCACATGATCAT
CACGGCGTTGGTCAAGAAAAGCAACTCCCAGACGTAAATGTATGAAAAGTTCTAGTCGTATAGTTCGT
CCAATATATTTACCGAGTCTTCTTTTTTTTTCCTTTGAATTTCTTACACCACCTGAATCAGTGCTAATTGT
TTTTCTTTTTTGTCATGGTTTTGATTATTATAGACTGGTTTAAATCTTCTCACAACATACACCAGTAGCGA
CAAATCATCCATGGATGATGGAACTTCATCGTCCCATAATATGGAAGACAAGCATAGAACAAATGAGGT
AGGCACGTATGTATAAATGGAATATTTGGACAGTTTTTTCTTTTTTTTCTTTCTGGGACTAGAGTTTTAA
AATAATTACAAACTATAGGAGCTTGAATTTCTATTAATTTTGTTGGTTCATTAATTTAGATGAATTAATATA
TATTCATGTGAGAGATTAATTTTGGGTTTTCATGCAGTTGGCAGTTCTTCAGGCTGAATTAGGCCGTATG
AACGTAGAAAATCAGCGATTGAGAGTTATGATTAGCCAGGTGAACAACAATTACCAGGCATTACAGGT
GCAAATTGTGACACTGATGCAACGCCAGCAAAATCAAAAGGTTGATCATCAAACTCCAGAACAACATA
AGGTATATATATATTAATCTGATTCCTTGACTACATAATATATATTTCTTTAATTTCACTAATATCCGCAACT
TCATGGGTTTGCTTAGATGAACAATAATGGATCAGTCGTGGTAGAAGAGAAGCAGATGATGAACGGGT
TCAACCATATTGTTCCAAGGCAATTTATGGATATGGGCAGGGCTGAGAAAGATGAGCTTTCACAGTGTT
CTTTGGAAGGCTGCAGATCCCAAGATTGTTCCGGGTCGTCTCCACGAAATGACATCGTTGAGTCAATG
GAGTGCTGCAAGAGTACTAGTCATGTACTTCACAGGGATCTTAGCGGAAGAATTAGTACTACTAATGGG
GGAGAAGACAGCCCTGACCAAGAGTTTCAAGGGTGGGTTCCTAAGAAAGTGTCCAAGATGATGAGTC
CCAGGCACGTGGATCAAGCCTCATCAGAAACCATGTCCATGATTAAGAAAGCCCGCGTTTCTGTTCGG
GCAAGATCTGAAGCGTCCATGGTAAGAAACTATCCTGTAGTTGAGATTTGTTTGTCAAACTGTATACAT
AGTTGTGATACAAATTGCAATCTTCAATATTAGTAAACGTATTATAACAATTAATTGGAAAACTCTGAAA
CAAATGTCTTCTTAATGAGACGGGTGGACAAATTGTGCAGTACAAATGTCTTCTGATAATTTCAAAGCG
CACCTCGATAACCCAAACAGAAAATAGGAAATTAGAACACACAAGCTTCTGTTGGAGTATAATTCAAC
GACAGAGAAATATGGATTTCATCATCCTAATATCAAATTCTCTTGCAGATATCTGATGGATGCCAATGGA
GAAAGTATGGTCAGAAAATGGCTAAAGGAAACCCATGCCCTCGAGCTTATTATCGTTGTACCATGGGA
ACTGGTTGCCCAGTTCGCAAACAGGTACATACTCTCTTTCTTGTTCTTAGGTTAACAAGTACATGCTTC
TTTCTTGTTTTTAGATTAACAGTCCGACAGCATAATATGTTAATAACTGGGCCTAATTGGAATCGTGGTT
GACAGGTACAAAGATGTGCAGAAGACCGAACGATACTGGTAACGACCTACGAAGGCCACCACAACCA

TCCACTCCCTCCTGCTGCAATGGCAATGGCGTCTACAACATCAGCAGCAGCATCAATGCTACTATCAGG
ATCAATGCCTAGTGCTGATGGCCTAATAAGCTCAAACAGCTTCCTGGCAAGAAGTGCCCTACAAAATT
GCCCACCAAGTTTGGCAACACTCTCAGCTTCAGCCCCATTTCCCACTGTCACATTGGACCTCACTCGA
ACTCCAACTTCCTCAGAGATGCCACTTGGCCAGCCAAACCAGCTGCCTCCAAGTTTTCCTCAAAACAT
GATGCCTGTGCCACAAATTCTAGGTCAAGCCCTTTCCAGCCAATCGACATTCTCGGTTCTCGAGAGCTT
TCCGGGATTGGATAGCGCTACACACTCATTGGCTGACAAAGTCAATGCAGCAACAGCGGCCATCACAG
CTGACCCTAACTTCACAGCGGCTCTGGTAGCAGCCATCACCTCTATCGTTGGCAATGTTCATTCAAACA
ATAATACCAACGGCAATATTACAACAAGAAACAATAGTGATAGCAATACATGA
>PmWRKY30
ATGGACGCGACCACACTCGACCACCCCTCTGGACCCTCCGATGACTTCGATCCGGGTCTAACCGACTT
CAATCCCGGGTCTGACCCGACCCTTTTTTCTTCTACTGGTGGTGGTGGTGGTGCAAAGTACAAGCTCAT
GTCCCCGGCCAAGCTTCCGATCTCGAGGTCACCCTGCCTCACTATCCCTCCCGGCCTCAGTCCGACGT
CGTTTCTCGAGTCCCCCGTCCTCCTTTCCAACATGAAGGTCTGCTTCGACGTTGTTTTGTTTCGATCCTT
GTTCTATTTTCTATTGTTGATTTTTATTTTCTTTTTGGCTGCTGAGGAAAAGCAAATGCTCTTTTTGAATC
TTATGCTGTGTTTGGCTTGCTCAGTGAAGTGTGGGAAGTGGGAACATACATAAAAATTAAACATGATCT
TCTAATTTTTATTTTGAAGGTTTTTTTGGGTGTGAATTAAATGTAAGGCTCTGTTTGGTTGCTGAGAAAA
GGAAATGTTGGTAATTGGAGTGGAAAAGTTTTAATTTTGAACTTTTCTCAGGCATTTTCTTGGTGACCA
AACATGGGGTATATAGGGAACTGGGTCTAACGGCCTGAGATTCCCCGTGGTATATGCTGTAGGAGGGAA
TTTTGTGGACTTAAAAAATTGATGTGATGTTGATGATGAGTGAGCTAAAAAGATGCTCCTCCTTTCAGC
CAAATTCGAAAATTCTGATTACAATTTCCGTTTGTTTTGTGGCATAACAGTGAATTTAATTAAATGGGTT
TGCTGCGAATGTTTTATTTCTTTTTATTTTTCAATAAACCAGTGGGATTTTATATGTGGGTGCTTAGGATG
GGACCCGAAGGTTCTGATGCTTTGTTGCTGTTTAAGAGTAATGTAATGCTAGTATTGGCCTATTGGGTCT
TTTCTCTTTATGCCCGTATGGATTGTTCGAAAGCAATTGCTTTGGGAATATGGGGCTTTCACTTACAACT
CTTTGAACTTTGTTGTTCTTTATTACTATCGACTGTGTTGTTTTTTCACATAATGAATCTTTCTTCTCTTCT
TTTTGCTTTGGTGGTGGGTTTTAGCCTTTTTGTTTGTCTTTGTTTAACGGTGGCAGTGGATGCAAGTAC
GACAAAAACGTTTACATTTGTGTATGTAGAATAAGGAATAAATGCATCAGCAGGTGGAAGGAATAGGA
AGGCTAGAAAGTTTGTGGGTCAAGGTGTTGATATAAGTCGTCATTGGCATGTGTTTTATGGAGGATATG
ACCTTCATTGTGCTGATTGTGATTAGAGATGATTGTTCTTGTTCAAAGCAGTTTGCAATAACTTCAGTTA
TGTGGGTAATCCATATAAATATATTGCTCTTCCAATCTTTAGTGGATTTACTTAAGAAATTTATCTTTTATT
TACTAGTATTTGTTTCATTTGCATCGGTAATGAAGGAACTTGTTAGAGTTATTTTGGTAGTCTAGGATGG
ATGGACAAATTACATATCTGTTAAGTGATGCCAATCAGAAGAATGCCCGAGTAGTTGTCAGAGTTTCCT
GTTATTGAACAGATATGTAACAGTATGTTTAAGTATTGCCACATTTAAAGGGGTTGGAAATTGTTCTGAA
TCTTCTTGGCCATCTCTCTCTTGTTCTTTCACGAAATTTTCTTTTTTCTGTACATGCCCCTTCATTCCATC
AAAAGTCTTCTGGATCAGGAATTCCTGAATGAAAATATATCGTTTCTGGTCTTGCAGGCAGAACCTTCC
CCAACTACTGGGTCCTTTTTGAAGCCTCAAATGGTGTACGGTTCTCTGAGTTCTACTACATATTCAGCA
AACACAATGTGCTCTGATTTCGATACCTTTGATGAAAGAAATTCTGGAAGTTTCGAGTTTAAGCCCCAT
GCTGGATCAAATATGGTAATTTCTTTATCAATTGTTGGTTATTTATCCTTTTATTAGTTGCATCTATGAGGA
CTACGTTTTATTTATGTAGTGAAAGATACTGATTAAATGGTGGCCTAGATCCAAAGTTTAGCTGATTGGT
CTTTGGCCTATTTTGTGCACTAACTTAAACTTGCAATATTGGTGGATGTCTACTTAATGCCTCAAAATCT
TTTAAGTCCCCATTCTAAAGCTTAACTAATTTGCTACGGTATTGCTCTCATACTTCCTCTGTGTCTCAAG
CGCTATATGATGAGTACTGGATTGGTTTTCTTGTGTCAACTAATACTTTATTAAGTTATCTACAATTGTTT
TCTGAAAATGCTTTATAGAGAACCAATGTTTTCTAAGGAAACTGTAAGAAGAATTGAAACAAAAGGTG
GTAGATGAATCTCTTTGTCGAGATGGACTGTCATATTTCTTTTTGAATTATAGAACAATGATATATCTTGA
TTTGAGTTCATTATATATCTGAACGTGTGTCAAAGTCGTTGTAAAGTACTGGTTGCCTAATGCTTCACAT

TTATTCAAGGTTACTACAGATTATAACCACCAGAGAAATGACCAGTTAGTCCAAGGTCAAGCTCAGCCT
CAATCACTCGTGTCTCCACCTTTGGTTAAAAGTGAGATGGCGGTCTCCTCAAATGAATTGAGTTTGTCA
GCACCTGTTCACATGGTCACTTCAGGAGCTAGTGCACCTGCTGAAGGTGATTCAGATGACTTAAGTCA
GAGGGGACATCCAAATCCTGGGGTCCAAACATCACAGTTTGATCATAAAGGAAGTGGGCCTTCAGTCA
TATCTTCTGATGATGGTTATAACTGGAGAAAATATGGACAAAAACACGTTAAAGGAAGTGAATTTCCTC
GCAGTTATTATAAATGTACCCATCCTAATTGTGAAGTGAAGAAGCTGTTTGAGCGATCTCACGATGGAC
AGATAACAGAGATTATCTACAAGGGTACACATGATCATCCTAAGCCTCAACCTAGTCGACGATATAATAC
TGGTGCTATGATGCCTATCCAGGAAGAAAGATCTGAAAAGGCTTCCTCTTTGATCGGCCGTGATGGTAA
GCATCAGAGTATTTCTCTTTAGCTGTTTTATGAATATATGGGCAATAATCTTTTATCATAGCTACCAATAAT
CTTGTTCCTTTCTAACTGTAGACAAGCCATCCAGCATATACGGGCAAATGTCGAGTACTAATGAGCCAA
ATAGTACTCCTGAACGATCTCCTGTCACAGGAAATGATGATAGTGTAGAAGGCACAGGTTCACTATCTA
ATAGGATGGCCGAGGAGATTGATGATGATGACCCATTCTCAAAAAGGAGGTATAGAGATTCAAGAAAT
GCTTATGTTTTTCCTGTGTTGTTCATGTTCCCTAACTAACAAAGTGTCAAACTTGTTAAGCAGGAGGAT
GGATGTTGGTGGGGTTGATGTCACACCAGTTGTTAAACCCATCCGTGAACCACGAGTTGTTGTTCAGA
CTCTGAGTGAGGTTGATATATTGGATGATGGATATCGCTGGCGGAAATATGGTCAGAAAGTGGTGAGGG
GAAATCCTAATCCAAGGTATAGTTTATCATCAGGCAGATGGTTTTCAACAATAATTCTGACTCATTTCAT
CTGTTTCAACATGTAGCTTTAGTGCTTGTTTGATAACTTAGTGAATGCTCATAAAATTGCTGGGCGTTCT
TCTCTAACCATTAATGGAATTTGATAACTTTCTGATACTCATACAAAAGTTGGGCTTTCTTCTCTGACGG
TTAGTGGATTATTCTGAAAAATATTGAGCTAATGATAACATTTATTCAAACAATTAAATCCCAATCTCTCT
GCAATGGAACATGGTATTGTAAGGACGGATTTTGGTAGTTCATTCTCCCACCCTCTGGCCGTTTCACAC
TCAAGGAATAAATGCGGATATTCTTAAATCCAGTATAAGAACTTAGTCTGACAGTTGATAGCTATGATTT
GAAAAATTGATGTCTTTCGAATTTGAACTTTTAGCCTTCCATTCTTTTTCCTTTTTTTTTTTTTTCTCTTT
CTTTAAGTGACTGTTTGCATCAAAATTAAGTTTTAGTGTAGGTAATTGTGCAATTCTTTGTTGCTACTGT
TGCAATTCTTCTCATTTAGTTTAACTGGAACTTTTCCATCATCTCCCTTAGCCACTTTCCCATTTTGATGT
AAGAAAATAGCTGTGCAGACCCTGCTTATTCATAAAGGACTCCTGAGAAGTGGCACCTTCAGCACAGA
TGGTTGGGGTATTTGGAATATTGCTAATTTCGCCTGATGTTTTACCCCAGTAATTGCATTAAACGTCAAA
CTTAAAGGAACAAAAGAATAAATTATAGATGTATTCTAGATCTCTTTCTGTAATTGAACCGACCTATAAA
TTATTTACCTTAGAATGTTACCTCATTATTTTGGATTGTGTTGGGTTAGAGATAAACCTGCATCATGCTTG
TGTAGTGTTTTTGGTTATCATGTATGTAAATGTACCCTGCTAACTTTGTAATTACAATGTTGCTTCTGAAT
AACTAGGAAGTGGTTCTATTACGTGTATCTCCCATATGTGCATGTTCATCGTGTGGCTAATATTTGGCTCT
AGTCTTTTTTTAACTGCTACTTTTTTCCAACTGGTTTATGGGGTTTCTCATCGTTCTCACATGCTATGCAG
GAGCTATTACAAGTGCACCAATGCTGGATGCCCTGTTAGAAAACATGTGGAGAGGGCATCCCATGATC
CAAAAGCGGTTATAACTACATATGAGGGGAAACATAACCATGACGTCCCAACTGCAAGGAATAGTAGC
CATGACACTTCAGGACCAACAACTGTGAATGTCCCATCGAGGATTAGATCAGAAGAAAGTGACACCAT
AAGCCTCGATCTTGGTGTTGGAATCAACTCTGCTGCTGAAAATAAATCCAACGAGCACCTGCAACTGC
ACTCTGAACTAATGGAACGCCAATCTCACACTAGTTCCAATTTTAAGGCCATTCAAACTACCCCTGTTT
CAACATACTACGGTGTTTTAAATAGTGGCATGAATCAGTACGGATCTAGAGAAAATCCAAGTGAATCAC
GTAGCATCGAGATTCCACCTTTAAATCATTCCTCTTATCCATATCCACAGAACATGGGAAGGGTACTAAC
AGGTCCATAGAATTGCTTAAGATGAAGCCAACAGAAATAAGCTACTTTTCCCCTTCTTTTGGCCACGGG
TGAAGGTCAAAGGGCATCTCAATTTGAAAGAAAGAAACAAAAGGTATTCTTCTTTGCCTATTTCAAAG
ATGTGGTATAGGTGCCTGCTGCGAAATTCCTTCTCTGTCCGATTGATGTATGTGCAACATTTACTTGTCA
GGGAATACTATGTACAAATATGAATTAATACTGATACTATATTCAGAGTCATTGACCGCATGTGTTGTAAT
TCGATCAAATGCTTAAATTTTACAATATTTATAAGAAAGTTTTGTTTCGCTACTTGGCCTGTGTGATGAG
CTTATTGAGCTCCTGAGTCTTAAATTTGCTTGGCATTGTGAACCCTGTTGCTAAAGCTTGAGCAATGTA

GATGCCGGGGCATTGTTCATTTGCGTCTGACGATTGCGATACTGTTGCTGCAATGAAACGACGAGATAA
AAGATAAATTGTACTCATGCCTGTCATTGTAGAATGGTCCCAATGGGAAGATCAATGCTGACCCTGTGA
ACTGCTGGGACTCTTATCTTGCTGTGTGGAGGGGGTTTGAGCTTTAATTGTTCCATTCTCCAGGAGAGA
AGGACAAAAGGTTCTCTATAGGCCTTTCGATGTGGGGTAAAAAATTTGCAGCTACTTGTTTTGGAGCCT
GTGAAGTAACTTGGTATTTCAACTACCACACAGAAGTAGTTTTTCATTTCTTCATACATTTAGACTAGAT
AGAAACTATTAATTGACCAAGGCAACAAATCACATGGATCAAAATCTGTGAGGTAAAAATTATGGACC
CTAAATAGAAAAGAAAAATAGAACACAGTGAAAAGTAAGAAGTTTACCAAAACAGTGAACACAGCC
AATGGGTTCCCACCATAGTGGTGGTGGCTTCCCCCTTCCCCCATGCCACCCCAGTTTGAACCGTTCGAA
CTCCAATGTAACCCAAAAAAAAAGGAAAAAAAAAAAAAGAACAAGAGAAAAATCATTAGAGATGAAGC
CAGAAGAAGATAAATTGTGGAGATGAGAATTATTTGTAGTTTTGTTCTGGTGGCCTGTTGTAAAGCTTA
CCGACCCTGGCTGGCATGGCAGCATTTGAACTTGCTCTTTAAATGCATTTGTATTTGTATAATAGGATGC
AAAATTCCTTAAATACATAATTTGTCTTTTTGCTTGGTGGATAATGTAGCACCACGCACTCTTGCAGCCA
CGTTGTGGCCCAAAACTCCACTCCCTGTAGGCCCCTCCAAGGGCCCCTTCAAAGTACGCAATTAATTT
GCTTTAGCCATGCTCAGTCCAAGTTAATTTTCTTGCCAAAAAATAAAATAAAATAGTATTTTGCTCTTTC
TTCTTAAGGACTTGTTTTATCTTAATCATTAATTTTTATTTATTTATGAATATTTCATTCAGTATTTTATTTTC
ATGTCAAACAAGTATCGGTTTTATTCTATTTTAATTTTTACTGCGCTTACCGTGGCTCCAGTCCTGCCTCC
TGTTAATGAAAAAAGATAGTAACAAGTCGCATATTCTTTTATACGTCAGCAACAGTATCGAGTATAAGGT
ATAGTTATGGGTGCATTTGTTTGATTTAAACTCAGAAAGATCTGCAACTTTGCAAAGATAAAATACTGC
ACCAGTACGAGTTAACTAGAATTCACACAAACACACTCCCACTCAGTCACTCGGTCAATCAAACAGAG
ATGCATGCTTTGTGGGTGTCTTTGAAGGACAATGTCAGGTGCAATGGGAACAAGGTGGCAGATGTAGT
TGGCCGGGCATCTGAAATATGTAACAGAAGAAAAAGCAGTTCAGGAAAGGCAAATTCTGGTGGTGAA
ATTGTTCATGGCAAAGAGACTCCGGCCCGAGAATTGATGCACCGGGGAAGCTACACACAAGCACAGC
TATATGGTAAGTCTCACAGCTTTGCTCTGGTTTTGCTTTATATTACCAATGACAAAGATCTTTAAGGATTT
TGAATTAAGTTTTCAGCAGTAAACTGCTGCATCATATCGTTGCAGCAGCAGCCATCTTCGAAGCGATAG
GGAGAACTATAAATGGTGTAAATTCTTCCTTACTCCCTGCAAGAGTCCATAGAGTGAGGTTTTAGATGG
TGACAGATGTTGTTTATAATTTGTCATGAAACCTCGTTAAACAAGATACAACGTAATCAAGTAGCTTTTC
ATCTCCATTTTTAAGCTCCACTTGTTAGACAACCAATAGACCCCATAAGCTTAACGTGTTAGGTACCAA
CCAAAAATATACGACAAGCAAAGCAAACACCATTATCATACTTTACAGTGTTTGCTTTATTATTCTCTTC
TTCTGGAATTATTCATGTAGACTGGAATGAAATCCTATATAGATACTATAAACCTAGGACTACCTTTCAAT
GCTTGGAATTACTGTTTCTTAAGGTACATGTCCTTCTTGTCTTAATTCTTTAATCAGAGCTAGACATTGG
AGACCCATCAAGGAATGTCATTGAGATGATTGTCCGAAGAGCAACGGTAAATCCATCAGAGCCATCAA
ACAGGATTAAAAGGGTCCTGAGAGTGCAGAACTCCATAGAAACGCTTGAAAGGTTTGAGAAGTACAG
AGAGATGGTAAAGAAGATGGCCAAAGAGAGATATATGAGGCATCCAAGAAGCACAGTAGATGGAAAC
GAGCTGTTGAGGTTCTATGGCACAACGGTAGCCTGCTGCAGTGGCGAGTCAAAGCGAGTCTCTGAGC
TTTGTAAGGCTCCAAATTGTCAAGTTTGCATGATAATTCAATCAGATTTTTACACAGAATATAGCGTGAG
CAATGAGATTCAACTGAGTACAAGTAGCGAAAAATTCGGTGAAAACAGCATTACCATCACAAGGAGG
AACAAGATAAGAAGGGCTGTAATAGTTTGCAGGACAATTGCAGGGAGCATGGTTAATATGACAGACAA
GGAATATGAAGAGTCTGATTCAATTGAAAGCCAAAAGCTGCTTTCTACTTCAGAATATTTGGTAGTGAG
GAATCCCTCTGCTGTACTTCCCTGTTTTGTCATAGCTTTCACCTGA
>PmWRKY31
ATGGAAAAACAAGGCCGTCGTCATCACATAATCCCCAAACTCTTCCCCCACTCCGTCTCCGATTGCTCA
TTTAATGCTTCCGATGATGGTCTTGTTGACGCTACCAACAACACTAGTAGCAGCAACCCCTCAATCCCA
ATCCCAATCCCAATCCCAATCAAACAACTTTTCAGACAACCAAAAGAAATCAAAGTAATGGATTTCTTT
TCCGATAATAATATCATGAATGATGATGCTGATGATGATGAGGAGCCCCGTCGTCAACCCCAACAAGAC

TGCAACAATAATCCTCCGGCTGTTAACGTAAGATTATACTTCCTTTTTATTGTTCTTTTCTTTTATATATCA
TCTTATATATCTATATATAAACAAAACCCACCTAATTACCATCATCACTACTCACAAATTATTATATATCATC
CATGGCCAGACAGGATTGAATCTCCTCACTCTAAATTCCGGAATTTCATCAACATCTGCCAGTGATATTC
ATCAAAATTCCAATAACAAGGTACTGATTAGTCTTACCACATATTTATTTACATATGGCTGTGTTTTTTTT
ATTATAAATTTCCGGCGCTCGATTCATTTTAGAATTTGATGTTTCTCTCTCATATGTGTTTCCTAATTAATT
TCCAATTATCATAATTAATTGTTGATACTATTAAATCGTAACTTGGTAACTTTTCATCAGCTGATGACTAG
TCTTCAAGTTGAGTTAGAGCGGCTACATGAAGAAAATGGCGAGCTCAAAACCATGTTAGACCAAATGA
CCAAAAGTTACAGCCAGTTGCAAGCTCAGCTACTCATGGCGATGCAGAAACAGGCACAAAACAGACT
ACGAGAGCCGGTATATTAAGTTTATATTTTTACTTTTAATCTTGTCTCCCCTCCCTAGAACTATCGCATAT
ACGTGATCAACATGATCCTGTTATATATAAAATTATGAATTGACAGGATTAATATATTAGGGTTTAGGCCT
GTTAAAAGGCAAGTGCTGAGGTGTTCTAATGTCCATTACACCATATATATTGGTGTCGTGCTTGAGATAA
TACCTATATGAACAGATGTGCACTCCACATTATTCTATTAAAAATTCAACAACATTGTAAAACCAGTCCA
GCATAAATATAAAGAATATGAAGTTAATATTTGATCACAACAGTACGTAAGGCATGGTTTCTTGAACAA
CAGATTAAGTGTGAGGCAAATGGCATGTTGGCCAGACAATTCATGGATCCACGGCCATCTACAGCTGC
TGCAATGGACCATGTCCGCGATCCATCAGTTGCCTATTCTTCTGGAAAGACACCGGCTGATCATGAAGC
CTTCTCATCATTTGCTCCATCAAATTTGAATATAGAGGTGATGTCAATGGAGCGTGACCAATATCAGAGG
AGGCTGCAAACTAATATTAATTGTGCGGAAGAAGCTCTTGACCGGTCATCCCAGTGTCCAGGGTCCCC
TAATTATACCAGTAAAAGTAGCGATCCCAATTTATTTGATGATGATAATGATGAGCCCAAACGTAGTACT
GATCAAGAACAAGTTCCAGTGGCTGATCAGATTCCTTTTAGGAAGGCAAGGGTTTCCGTACGAGCAAG
ATCAGAAGCTCCTATGGTATGTACATTGAACCACTTGTACTCAACTAAATATATACATGCATATATGAGA
GTTTTGCACATGAACTCAATCATTCTATTGCTAAACTTATGTTACATACAAATGCTAATTATGTTGCAATA
TTGTGAATTTCGACCACATCAATGGTACATATTCACAGTCTAGCTAGCATAGCAAGACAGTTTGACAAT
GTGTGACTTCAATTGACTTGTGATATGAATCCAGCAACTAATGGTTTCTTAATTTGAAAATGTTTGTGGA
AATAAATATATGCAGATTAGCGATGGATGTCAATGGAGGAAATACGGTCAAAAGATGGCAAAGGGTAA
CCCTTGCCCACGTGCTTACTACCGTTGTACTATGGCCATTGGATGCCCCGTTCGCAAACAGGTGCAAAG
ATTGGCGGAGGATAAAACCATTCTCGTAACTACTTATGAAGGTAATCACAGCCACCCTCTGCCTCCAGC
AGCCACAGCCATGGCCAAAACCACGTCAGCAGCAGCTGCCATGCTGCTATCAGGTTCAACTACAAGC
AAGGAAGCTCACCAGTATCATCATCATCATCTAGCAAATTCTGGCTTCTTCTCCAATTCCCAGCTGCCCT
TCTTCACTTCGTCCATGGCCACATTATCAGCCTCTGCACCATTTCCCACCATCACGCTGGACTTGACTC
AGAGCCCCATGCAGCAATTCCACCGAATCCCGCCACCTTCCTCATCCACATTTCCTCTGCCTTTGCATG
GTTATCACCAGCTTATGGGTGGGCTGGGGCATCCTATCCAAGCCCCAATGTATTTCCCTCCTAATTACAA
GGCACCCCCACCACCTGCTGGAGTGCCGCTAGGCGGCCAACGTAGTACTAGTACTCATGATAGTGGCA
TGATTGAGACTGTTGGTGCAGCCATTGCTTCTGACCCCAATTTCACTGCAGCCCTAGCTGCGGCCATCT
CAACTATTATGGGGGCACCCCGCCCCCACCAGGGCCACCAGGGGGGGATTAATATTAATGATGGAGAC
ATCGCTAATAATAATGCAGCTAGAGGAGTAGTTGCAAGTACCAATCATTCCCCTCCTAGTGCTAATATTG
GGGTTCCACCCGGATCATTACAGCCTGGATCCCCACAACTTCCTCAGTCCTGCACCACTTTCTCCACCA
ACTAA
>PmWRKY32
CTACTCTTGCTTCAGCATATGGGAGGGGACTATGTCTTGAAGAAGCCCATGGTCTGTCAACATAGCAGA
ACCAGTTGCTGGGGTGCAAAACCGCCTTTCGTGATGAAAGCCAGTGCCGCTAGCATTTGCTGTTGGTG
AGCCACCATTAACATAGTTGCAGAAATTGAAAGGTTGCAGTTGCTGCTGATAGTGATGAGATAATAGGG
TTCTGGGCATTGAAGGCATGGCAAAGGTGGTAGAGATATTGGGTTGAGCAGAAGCCGAAGCCGTGAG
AGTTGGGCGAGGTATGAGTGGGCTTGGGTGTGTGTGTTGGCCTTCATAAGTTGTCACAACGATGCTTG
GGTCATTGAAAGATCGCTCCACTCGCTTCTTCACATTACATGCTGTACTTGTGCATCGATAGTAACTCCT

ACAATCAACCAAAATCATGAAATACATACATAAGATCAAAGATTAAAAAAAATAATAATAAGGAAGATT
ATGATTTCTGATTCAAAACCCATAACTAATTTAATTCTGGGTTTTGATTAATTTGAACTGAGGATGATTAT
TATTAACAAGCAGTTAGCAATGCCATGATAATAAATAAGTGTTAATTTGGCACTGAAGTTTAGAGTGAG
TACCTGGGGAAGGGGCTGTTTTTCACAGCTTTTTGACCGTACTTTCTCCATCTGTAGCCATCTTCCAGA
TGATCAACCTCACTCTTGGTCATGAATGCAAATCTGGGTTCTCTCTGTCTCTTCTGACTTGCCTTCTTCG
CTTTCAACCTTTGCTCATCCACACAATGAACACAGAGAAAAAACAACCTTTCATCAATACACCATCAG
GCTTTCTATTATATAATGAAAGTTGAGTTCACTTTAACCCAAAGTCCCAATTTTCAATTTCAGGTCGCTG
TACTATAAATCCGAAAATGATAAAAACCCCATCTTTATCTTGATCTGTTAGGAAATGGATCTCTCTCAAA
AACAAAACTTAAAGCAAAGCAGGAATGAAAAACTCACTCTTTCTTGGTCTTTGGTTGGTCACGTTCTT
CTTCTTCATCTTGGTCTGCAGCACCTTTATTATCAGTCTGTTCCTCGTTCAGAGCCTCACTGGATGCAGA
TGAAATCGATGAAGAGTTTGGTGTTGCAGGCTGCTGATTCAAAGAGTAGTCAGAGCACTCCTTTGCCA
TTATGGAAGTATTTGGAAAGTTTGGAGCTAATGAAGGCAACATAGATGGTGTCTGAGGCAAATAATCA
AACAAGGAGTTAGTACAAAAGTCCTGGCCAGCACCCAAAAGCTCCATAAACCCTAAAGATGAGCTCT
TTTCTCCTTCACAGAAATCAAATATGCCTGAAAATGGTGATGAGGAAAACCCACATCCAATATTGGTAC
TATTGTCCTCCATCTTTACCACATCCTTCTTCACATCCAT
>PmWRKY33
ATGGACTACTCATCTGCTGCATATGATGATACTTCTTTGGATCTTAATACCAAGCCTCTCCGACTTTTCGA
TGATACTCCGGTAGGTGCAAACTTTATGCATCTGAACTTTTAGTAGAGATCTAATCTCTCTCTCTCTCTC
TCTCTCTCTCTCTCTCTACTAGCTAGTTATCTTTGAACTTACAGAAGTTGATTGGTTGTTCTCAGATCAA
GAAAGAGGCGCAAAGCAAAATATTGATTGGCTTTGGGAGGCAGCTTTCACCAGATGAAGAGGTTTGT
TTTCAATTCTTTCTCCACCTTAGCTTCTCTATCTTCTGTTTTGTGGCTATATCTTCTGTTTTGTGTCTAGGA
TTTAATGTGTCTAGGAGCATATTTTTCAAGCTATTATCATATTGGATCAAAAGCGCGTAAATCTTCGACA
GACGTCTTACAAATTAGGATGATTAAAGTACTAAATATGAAACATGAAATATCAAATGATTTATTAACTA
TTGGATTGCATGTCTAAAGAGTTTGTTTTGTTCTCAGAGTGGTGCTCTATTGGAGGAATTGCAACGGGT
GAGTGCAGAGAACAAGAAGCTAACCGAAATGTTGACGGTGATGGGTGAGAGCTACAATGGTTTAAGA
AACCAGTTGCTGGATTACATGAGCAAGAACCCAGAGAAGGAGCTTAGCCCAATTTCAAAGAAAAGAA
AGTCTGAAAGCAGTAACAACAACAACACCAACAGCAACAATAACATCAATGGAGCAGTGAATGGAAA
CTCTGAGAGCAGCTCCAGTGATGGAGAATCTTGCAAGAAACCAAGGGAAGAGAACATCAAGGCAAA
GATTTCAAGGGCTTATGTTCGTACCGAAGCATCAGATACAACAAGCCTGGTAAGTAATCAGTCCTTGAG
GAATGTTCTTGTTGGTTTAGTTAATAATTTAAGGTTTGTATTGAGAAATTTTATGTGTATTTATAGGTTGT
GAAGGATGGATATCAATGGAGAAAGTATGGCCAAAAAGTTACTAGAGATAATCCTTGTCCTAGAGCTTA
CTTCAAATGCTCTTTTGCTCCAAGCTGCCCTGTCAAAAAGAAGGTGAGCCCAAATTCAAAACTTTCCC
CCTCTTAATGCTTCAAAACTTATTATGTAAACAGAAGAATTAATTGGTTTTAAGAGCAGGATTTCTGTTC
AGTTTTTTCCCCCTTTTAAGTGCCATATTAATTAAAGGTGCTTCTTGTCCATGGTTTTTGCTCTTCAGGTG
CAGAGAAGTGTTGAAGATCAATCTATTCTGGTGGCAACTTATGAAGGTGAACACAATCATTCCCACCCT
TCTCAAATTGAAGCAACATCAGGCTCAAACCGCTGCATGACCTTAGGATCAGTCCCCTGCTCAACCTC
CCTTGCCTCATCCGGACCTACCATCACTCTTGACTTGACCAAATCCAAGTCCAGTGCTGACACCAAAA
GTACGAAAACAAAAACCGAAACACCGGAAGTTCGAAAGTTTTTGGTGGAGCAGATGGCTTCTTCCTT
GACAAAAGATCCCGATTTCACGAAAGCACTAGCAGCAGCCATTTCAGGAAGAATACTTCAACATAATT
CTTACTGA
>PmWRKY34
ATGGACCCACAATTCTACAGAATTTCCCCATTTGGGACTGACCCAGCAGACCCTGATCAGCCCATGAC
GTCAGAGAATGGTCCGGGCTCTCCCTCCTCAGGGGAGGAGACAAAGGTGGCCACTGCCCCATCACCC
AAGAAAAGGTAATTAATTAATTAATTAACAAGGTTTTTTTTTTCTTTCTCTTTTTGGGTTTTTTCTTTTGC

AAAAAAAGGAAAAAAAAAATTGTATGGTTGTTTATAATTTTGGAAATTAAATTGACCGTTATAATGGTTG
GTTCTGTTGGTTTCTGTTTTAGGAGGGGAGTGCAGAAGAGGGTGGTGACAGTACCGATCGGTGACGTG
GAGGGATCCAAGAGCAAAGGGGAGGGCCATCCACCGTCCGATTCTTGGGCTTGGAGGAAGTACGGCC
AAAAGCCCATCAAAGGGTCTCCATATCCCAGGTATTATTCATCTTTATATTTTTCTCAGTACAATTTTTTA
TAAAATTCAACAACTATCGTTCATGAGATTTGAATTTAGAATTTTTCGAGACAAAAAAAAATATTACAGAT
GCAGTAATTTTAATCATTTAGTATGTGAATATCATATATTATATGGTTTAAATTCAAGAGATTATATTTTTAT
AGTATTTTGAAAATCCTAAAAAAATCCACTCAATTAAATTTTTTTTTTTGGGTGGAAAATGAAGAAAAAT
AAAATAATTGATGCATTGAGCTTCATAAATTTCGTGGTGGAATAGGCATGTAATTTTCTTGTATTCGCCG
CCGGATGTTGTATTGATTATTTTTTTTATTTTTTTTAATCAATGATGTATTGATTATTTAGTGTCTTTCATTT
GCTACACTGCGGCGTATTTTAGTCTATGAAGGCGGCTCTATTAAAATGCGCCTGCTGTCGTAGTAGGGA
ACACCGTCCACGCTCTCACCACATGTCGTGAAAAGCACGCTCTAGGACCCAGTTCAAAAATGACAGAT
TCTCTCTCACCCTTTCTCAAGATTTTAAGGTTAACTGAACCACAATATATAATGGGCTTTTCATGAATAG
GTCCATTCTACAAGTATCCATATAGAGCCTATTTAGGGAAGACAAAAAAAAAAAACTCAAAACTAGCA
AAACTAGCCACTTGTCATTTTCTAATTGGTTCTTAGAAAATTTTAACACACTAAGAACCAATTAAAAAA
AGACAAGTTGCTAGTTTTGGATTTTTCTCTTCAAATAGGCTCTATATGGTTATTTGTATAATATGTCATGC
AAAAATAAGGTTTTTCCTAAAAACCTAATATATAATTGCCGTGGACTTTCATTTGGCAATAATTTACGGG
AATATTGTTATTTGTACCATATTTACTGACCACATTGTTGACCACCTCTCTAATATAAGTGAGTCTCACAT
ACAACAGTATAGTCCACGTTTATTAGAGACGAGGTGGTCAACAAGGTCGTCAATAAATGTGGTACAAA
TAGTAATATTGAACATTGAGATGTAAAAAGATATTTTATATGAACCAAAAAAATAGGAATACGAAGTTTTA
CTTTTATGCCAAGGGGGTCTAGCCTAGTGGCAAATAGCCTTAATTTGTAGACAAATAGTCTCTGGTTTG
AATCCTCATGGCAACATGGTAATGTTTGTGAGAAATCTCCCTCCCTATTCCTAAGCAAAAAGAGAAATG
TCGCATGTCTCATTCTATTAAGAAAATATGAAGTTTGAGAAAGAACAATTGGTTTCTGAATATTTTCTAA
ATTTTAGAAGAATTTTCCCGAGCTATATATAGTTAGTAGATGCATCTTCCGTATAGCTTTTACATGTTTACA
TGTAAAGCGACCTCATTTTGAGATTTAAAAGTTAATTTAGGATTTAGGATTTAGTAATATCTTGTGCTAC
CCACTCACATGAGGGTTTGAGACATGCTTAATGAGGCTTCACCTACTTGTAATGATGAACAGATTAAGA
CCACCTTGCTTACTTGATTTAGCTCATTATACTAGCAACTCAGTTACAAGCCCACTTTACAAGACTTTGG
TAAACGGCATTATTTTTAACACGAACGAACATCCTCATTCGAAAATAATGTATGCTTCTTTGTTAATAAA
AAATAAGTTTGCTCTCTTTCAAATGTGTTAAATGTGACACAAATTTTTATATAAGATTTTGATTATGATAC
GACTGCAAATTTTTTATTGATGGCGTTTGTTTTAACTACTTGTTTGTTAATAATTTTATTTAATTCACGTTT
GGTACTTTTTGCAGGGGATATTATCGATGTAGTAGCTCCAAGGGTTGCCCGGCAAGGAAACAAGTGGA
GAGAAGTCGTGTGGACCCCACAATGCTCTTGATCACCTATGCTTGTGAGCACAACCACCCCAAGCCCA
CCACCAAGCCACACCAAACCTCCACCACCACGTCACCCAACGCCGAACCCGAACTTCGTGCCAAAAC
AGTCACACCCAATGAAGAGGAGCTCACAATTTTTGCGAGCCAAGTCGACCTTGACCTTAGTGACGACT
CGGCCACGTTGCTTAGTGCCTTCGGTTGGTTCAGTGACGTGGCATCGACAGCTGTTCTAGAAAGTCCG
ATTTGTGCGGGAAATAGTACTTGCGCTGATTATGACGTGGCAACGAGGTTGGGAGATGAGGAGGACGA
GTATTTGTTCGCCGACCTGGGCGAGTTACCGGAGGGTTCAGTGATTTTCCGACACAAGATGGTGGAAT
CGGACGAGCAGAACCGGAGATGTAGTTTAAGTGTAGTTCCTTGTTGCAATAGTAGATGA
>PmWRKY35
ATGTCAAATGAAAAGAAAAACCCTTACCAGTATGACCCTTTCGACTACAACCCCCATGAAATCAACAG
GTCAAGCTTTCCATTCTTCAATTATGGCACTCCCTCCATACAAGATCCACAAAACCTACATGGGTTCGA
ATCCGATCACCCCAATTCTTCGTTCATGAGCTTCACTGACTGCCTCCATGGCTCAATGGACTACAACAC
CCTCTCAAGAGCCTTTGACATGTCATGTTCTTCATCTGAAGTCATTTCTCCCCAGTTGGATCATGAGAAT
TCCAAGAACCAGCAGGCTGCTGCAGCTGCAGGTGTAGGAGATCACTCGGTGGGGACTAGCACCACCG
AAAACCCATCTACACCAAACTCCTCAGTTTCTTCATCATCTAATGAAGGTGCTGGTTCTCATGAACATG

AAGATTCAGAGAAGAAGAAGAAAGAAAAGCAGCCAAAAGTGGCAGTGTGTGATGAAGCGGCAGGA
GATGAGGAAGACAAGTCAAAGAAAGGGTAGGTTCTTTTTTTGGTTCTTTGTAGCCAGATTCATGGTAA
AGGTTAATTAGCAGCTTTTGAGGGATATCACTTGTTACTCCACTATTTTCCACTCTTGGTATATATATATAT
ATATATATATATATATGTATGGATAAATATAGGGCTTGGATTGACTAATATTTTGTCAGGAGGTAGACTGTC
ATATAAAAGGTAAACAAAAAAAAACTTTTTAGTGCAATTTTCCCCTTGTGTTTTCAACTCACATGTCATT
AATTAATACGTCTTTAATTGAAGAGATATTTCTTTAATTATTTTGCTTTCTCATTAGATTTCTGATGATTAA
CCAATAACCATACATGCAACATCTAGAAACTTCTGGGGAGCAAAAAGGAAAATAATGGGATCTGTGTT
CAGAACCCTAAAATCGAGTGCATATACACATCTAATTTGCTTAGATATATGTGATTCAGGAGCAAAGCG
AAAAAGAAAGAGAAACGGCAGAGGGAACCACGGTTTGCCTTCCTGACTAAGAGCGAAGTGGATCAC
CTTGAAGACGGATACAGATGGAGAAAGTACGGACAGAAGGCAGTCAAGAACAGCCCTTATCCTAGGT
ATACTTAATTATACTTAATTGCCTTTCTTTACAATTTCCACACTAGCTCAACGTACATTCCATTAAAAAAA
GATTATTAAAAAAAAAAAAAGAAAAAGAAAAGAAAAGAGTCCAACTTATAATGATTTCTGGATGTGTGA
TGTGAACAATTCTCCAATTTTTCAAACCAGCCGTTATTTTTTGGCCGGTCAATAGGCGTTATTTGATGAT
CAAATATTTGTTATATGTCATTCATATCTGTAATGGTTACTAGTTAATTAAAACTCTGTTCTATTATATGCA
GAAATGATGTTAGCCTTCGCATTAAGCCCTAACTATGTAGAACAGTTAGGGCCCCTTCGATAACCATTTC
AATTTTAGTTTTTAGTTTTCATTTTTTGTGCTAAACTATAGCGGAAAATGAGAGTTCGAATGGGAGGATG
AGTAACAAACTTGAAAGTGAAAGTAATTTCAAATGGATTTCAATTTTTAGGTTTTGTTATAACTTTTGTT
ACTCCTCCCTCCCATCCTCCCATCTCAATCCCATATTCCACTCATTTTCTCACTTCCATTCCCCTTCTTTT
TCACTTTTGTTACTCATCCCTCGTATTCTCTCTTGCAATCTCATCTTCACTTTCATTCTTTCTCTTTTTCCT
CTATACTATAGGACAAAAAATAAAAACTAATAAAAACTAAAGTTGAAATGATTAAACGTAACGGGCCCT
TAGTTTGTTTGATTGGCTCTACTTTGTAGTTTGTTCTTTAATTGACATCATTCATTTCTATGTTTGTGATAG
AGAAACTTATATAACCTGAACATTAAAAGAGTAATTTGGTGATCTCACACAGCTTTGTAACTACCTTCTT
CCATTTTCAGAAGTTATTATAGATGCACTACTCAAAAGTGCGTAGTAAAGAAGCGCGTCGAGAGATCAT
TTCAAGATCCATCTATTGTGATCACAACATATGAGGGTCAGCACAACCATCAGTGTCCGGCAACACTCC
GGGGAAATCTCAATCTCAATGCCGTTGGAATGCTGTCCCCTAATTCCCTTTTGACATCTGCGTCTCTCAA
TGGATCAGCGAGATTTCAACATGAATTCTTAACTCAGTTTCTCCCAATGAACAACCAATTGCAATTGCA
ATTGCAAAGCCATCATCATCAGGATGATCACCAAGCAAGTAATTCCATGATATATTCAAACCTCGTGGC
CCCTCGCCCTCATCATCATCAGCAGCAGCATCAGCGGCAGCATCAGCGGCAGCAGCTACATGTTCCTG
ACTATGGGTTATTGCAAGATTTAGTTCCTTCATTTGGTCACAAGCAAGAGCCATGA
>PmWRKY36
ATGATTTCTCTAGGGGAACCTGGGACAGATAAAATTGCTTCTGATATAGTACCGAAGAAAGAGAGTTC
AAATAGTGAAATTCATGCCCCACATCAAACTCCTGATAATGGGATCTGTTCCCTGCAATCAGATCATAG
AGGAAATGTTCAGTCTCTAATACCTGAGAAATCGTTACAGCTGCCTGATGATGTTGGTACTGCATCTCA
ATCAAATCAAGAAGGAAGTGTTACCTCTTTAACATCTGAGAAAGCACCACAAACCCCTGAAACCTCTG
CCCTTGTCTTGCGATCTGGTCAAGAAGGAAGCACTCCATCTACAGCACGAGAGAGAGGGTTAGAGGA
TGGTTATCACTGGAGAAAATACGGCCAGAAACTTGTTAAGGGAAATGCATATGTACGAAGTTACTACA
GATGTACGCATGCAAAATGTCCAGTGAAGAGGCAAGTGGAGCGCACACATAATGGGCAGATAACAGAT
ACTGTTTACTTTGGTGAGCATCAACATCCTAAAGCTCAAGTTAACGTCCCAGTAGCTGTTAGTTTTCTC
GTGTCCATTGTCGAAGAAAGACCAGAAGAGCTTTTGTTAACTGGTGTCGAAGGTGAGAATGAAACTT
AGTTATTATCTAGTTCTGGTTACAGTACCCAATCTTGTTTGATTTTTATTTGATGACCTTGTATTTGAAGG
CAAATCATCGGATGTGCATGGCCACACATCTAACCAGATTGAGCCAGTGGATCCCGCTCAGCTATCAAC
CGTTGCAGATAATGAAGGTGTGCAGAGAGTGCTCTCTCAATCAAATAGAACCAGAGATGGTGATCCAG
ACTCAAAAAGACAGTATGTTCATTCTTAGCTATTTTGGATTTTCGTGTGTAGTGGTATATGGTATTCACCT
TTGTTTAACTGCCATTTTGGGGGGGGAAAAAAAAAAAAAAAAAAAAAAAACAGGAAGAAGGAAAAAC

ATAATGGCAACTCAATTCCGGTGGATAAGCCAGCTGGTGAACCGCGTGTTGTTGTTCAGACTATGAGT
GAGGCTGATATAGTGAATGATGGCTACAGATGGCGCAAATATGGGCAGAAGTTAGTAAAAGGCAACCC
AAATCCAAGGTACATATCAGTTTAATGGTAGCATGTATTAACTCTTTATATTAGGTGTGTACCTGAATTTT
GCCACCCTGCTTCTTGTTTTGGAGATATAATAATGTATAGATCATGGTTTTGATAGATGCACTGCTTATGC
AGGCTAAGTTTACATTAAGAGTTTTGGACTGTGAGCCTAATATTTCTGCTTGATACTTGCAAGCAAGGG
GAAGGAAGAAAATTTTAAACGTAGTGATCATTGCAAGTTTGCAACTGCTTAGTAGCATCAAACTGCAA
CTGCAGCAATATGATAGTATCCGGTGGAAGTAATTTACATGCTTTATGACACCCCTGAGCTGGACTAAG
GACTTAATTGCGGATGTGGTCTAATGGTGCAAACCAATGTCTGGCAAAGTGCCTTAATAAAAAAAAATT
CATAAAAAACACACGTTCTGAGACTTTTGTGCCTCCATGGAACCATTTCCTATGCAGTAACTGCCTAGA
CCAACTAAGGCATTGTTTTCCTTCATTTAACTTGCATATACTTCTGTAGTCTTGTATCTTGCTTTGATGAT
AAATCGGAAGAAGTAGATCCGATTACTTCTTAGACAATAGGGCAACTGGGTAAATGATTCACTATGCAT
CTGTAAGCTTTGTTCAAACATGTCCTGCTTCTGGTTTTATTTCGTTTTGTCTTCCTTGGTATGTATCTTGG
AGAAGGATGTGGACGAGATTTGTTTATGTTTTGAAAATTTGAAATTAATCTCCTTGGTATTGATTTTTCG
GTCCTATTTTCTTTGGTGTAATTTTTTGCTGACTCTGCTCTGCTTCTGCACTTGCATTGCGTAGGAGTTA
CTACAGATGCTCAAATCCTGGGTGCCCTGTTAAGAAACATGTAGAGAGGGCGTCTCATGATTCAAAAG
TTGTTATAGCCACATATGAGGGGCAACATGATCATGATATGCCACCCACAAGGACTGTGACCCACAATG
CAGCAGCATCAAATGTGATTACAACGGCCCGTAATGGTGAGTCTGGCACTACGTCAGAAGGGAATGCT
GTCTGCCATGATACTAGCCCAGAACACGAAGATAAACCAAACAAGCAACTTAATGTTGAGCCAAGAA
CTAAATCAAGTGATGTTGCTGGCTGTGATATGGTCGTTGATTCTGATCTGGGTCCTGAAAGAAAATTAA
ATGAGCAAGTGGTTGGCAAAGCATGTACCACAGAAGAAAGTGATGCCCCCGATATAATTGTTCCTAGG
GCCAATGAATTGCAAAATGGCGAGTCAGGAATTAAATCAGAAGGAAACAACGCCTGCATTGATACGGT
CATTCACGGCAATCTGTGTCCCGAAAGTAATTCACCGGAGCAAAAAAATCCAAAAGCAGAACCTGTCT
AA
>PmWRKY37
TTAGTTGGCTGGAAAACTTGAATTGCTAAGTTTATTGTTGTTGCTGGTGGCGTTGCCGTTGCCATTGCC
ATTGTTGTTGTTTGAGTTGGTTGCGGGGTTGGTGCCGTTGTTACTATTGTTGTTTGGATGAGCATTGCCA
ATAATGGAGGTGATGGCTGCTGCCAGGGCTGCAGTGAAGTTTGGATCAGCTGCAATGGCGGCAGTGGC
TGCAGTGACAGTGTCAGCCAATGAGTTCTGCTGCTGCCCTTGGTGCCCTGGTTGCTGTTGGTGACCCA
GTTGGGCACCCTCCATGTCTTGTGACATTTGCAGGCCAGAGAATTTTGACTGGTTATAGAGTGCTTGAC
CAAAAATCTGAGGCAGCAATGAGACGGGTCCATTGGTGAAATTCTGAGATGGGTTTGGGAATGGGATG
TTGAATTGGCCTGGTGGCCTTTGGAGCTGTAAAGGGTTTGGTGATTGTGTTAAGTCCAATGTAACAGTA
GGGAATGGTGCTGAGGCTGAGATGGTGGCCATGCTAGAGGAGCATGGGAGGATTGTCCTGGTGAGGA
AGTTTGAGTCCATTAGGCCATCTGCACTCGGCATGGACCCCGAGAGTAGCATTCGTGCCGCGGATGAA
GTAGTTGATGCCATTGCCATGGCTGCTGGAGGCAATGGGTGGTTGTGATTGCCTTCATATGTAGTAATTA
GGATTGTCCTATCTTCTGCACATCTTTGTACCTGTTTGATCATAGTAGCAATTAAAATTAGTACAAAGAT
CGAAGATTAACATGTTGTGAATCATAACCGAAAATGGAAACAATCTGACAAAATAACATGTCGCATATC
TAATAAGAGAATGTTACCCTAAGCAAGCGAGCTTACTTGTTTACGAACTGGGCAACCAGCAGCCATGG
TGCATCGATAATAAGCTCGAGGACACGGGTTTCCCTTTGCCATCTTTTGTCCATACTTTCGCCATTGACA
TCCATCAGTGATCTATAAACCCAACAAAATTTATCAAATTAGCAAACATGTTGGTACAAAATCTGATGC
GTCATATATATAAAACTCCAACATAGTCCAAAACACATACCATAGGTGCCTCTGATCGAGCTCTGACCG
AAACTCTTGCCTTCCTCATGGTAGCCTCAGTTTGATCAACCTCTTTGGGAGAATTGAGCCTTGGAACTT
TGTTAGGGGCCCAACTCTGTGATGGCTGATCTGGGCTCTCCTCTCTCCCAATCCCTCTTCCAAATTCCTT
CTTCTCCTGATCATCACTATGCCCAGCAACCTTCACATTCTCTCCAAGCGATCCAGACCGTTCGCGGCT
TCTTTCTTCAGATGAAGACTGTGAAGGCTCATCAGCGTCAGCATTATTGGCAGCCAACCCGAGATCCAT

AAACTGCCTAGGGACCACTACTGGCGATCCATTGCCATTAATGAGCTTCTTTTCTTCCACCACCATTTTG
TTGTTCCCATCAAACACTCCATGGCCTTCAGCAGCGCTACTGTTCTGCTCAGCCTTCTGGCTTTGCATC
AACGTCAGCAAATGCACCTGAAGAGCGTTGTAATTGGTAGTCACCTGATTAAGCATGCCTCTCAAACG
TTGATTCTCCGCGTTCATTCGCTCAAGCTCAGCTTGAAGAACAGCCAGCTGAGAAAACAAAAGTGATA
CCAACATATATTAAATATAGCAAATTAGCAATAACCCAAGAATCAGGTCCAAAATTCAGCACAAATTAA
ATAAATGCAAAAGCTAAATTTTGTATCTGTACCTCACTTTTAGCTCTTTTATCTTCTATGTTCGACGAAAT
GCCATCATCCACCACTGATTGGTCACTGCTAGTGTTTGTAAGGAGAAGATTCAAGCCAGTCTATAAACA
CATAAACAAAAACTAAAAACAATAAGAAAATGATGATGACTGTTTGTCGAAAAAGGTTCCGACTTCAT
TAAAATCTCATGGACGACAGTTGTTGAATTAAGAACACCGATAAATCAATAATAACAATGAAGATGAAG
CAAAATGGTGAAAAGGGTCGCTTACATTTACGTTGAATTCCATATCCGTCGGGCCATGTAAGTCCATTTT
ATCAGCAGGGTCGGCGGAGGCAGACTTGTCTTGATCAACATGGCTCTTGTTATCGGCGAAGAAGTCCC
TCTCGTCGATGACTTTACGTTTTTCATTGGAGGGTGGTTCAGGAGAGTGCTGATGGTGGTGATGATGAT
GTTGATTATCAACATCTTCATGGGTGCAGTTGAGGTTGACTGAGAATTGCAGAGTTGGAGGAGGAAGA
GGAGGGGGAGGAGGAGACCTCGGGTGAATTGTGGAGGCGTCCATGTCCATGTTGAGTGAGGAGTGAT
GAAGCTGCTGCTGCTGCTTCTTGGTCCTGCAGGGTTGATGGTGGTTATATTGGTCTTGTTGAAAGGAGT
TGAGTACTATGGGATTGTGGAGAGAGAACCCAAATGGATCTGAATCAATGGAGAGTCCACTTCCCTTG
GCCAT
>PmWRKY38
ATGCCAAACTCTAATAGTAGAACAGCCCCAGAGAAGAAAGTTGATGAATCTGTTGGCTATCAAGAGAT
TTACAGTCAGGAGGTAAGAGAGGTATATGTCTTAAAATAAGTAATTACTTTGATAATCTCTTTTTTTGTG
GCAAATTAATGATGTTTTTCTATTTAATTTCCATGTTCTCTCATGAGAGATATGGTTGCTGTTCAATATACT
ACTGAATATGAATATCTCTCACTGAGTTTCTATGAAAGAGATCGATGTATATGTCTTGAAAATTCTACTA
GTTTTCTGAATAGAATCGCAGTTTTTGTTTTTGTTTTAATTTTGAGTACTTTTCTTAATTATATATTTTATG
GAGTTTCTGTTCATGTATAGTTTCACATTCAATGTACTGTCCATTACCAGATATAATCAGTGATCACTTGT
TGCTAGCTACTGTTAATAATTTTAATTAATTTTTAATTTCCTTTACAGGTTTTTAATATCTTCAAATTTCTA
ATACATATCTGTTAATCTTGACTCTGAGTCCTTGTTAAGTATTTCGTTTTTTTATTTTATTTTATATATGATG
GATGTATAGGAGAGTGAACTTGAAAATGCCAGAGCTGAAATGGGTGAGGTGAGAGAAGAAAATGCCA
GACTAAAATTGACCTTACAGCATATGGAGAAGGATTACCAGTCTCTCCAATGTCGTTTTTTTGACATCC
TTAGACAAGAGGCTTCCAAGAAAGCTACAAATGTTGATGTTGGTGTTCATCGAATTGAAGAACCCAAT
CAGCTCTTGTCTCTTTGCCTCGGAAGAAGTCCAAGGGAGCCCAAACATGATGAAACTAATACCGCCAA
CTTCAAACTTGTACAAGTTGATCATGAAGACTTGAATGCCAACCTTACTCTTGGATTAGGTAACTCTAA
ATTAATGGAGTTGCCTATGGAGCTTGTGCGTAGCCAAAAACCTCAGGAGACTAGTTTGGAAGAGCCCA
AGGATCATTCAGAAGCTGGAGCTGGAGAATCTTTGCCTCCATCAAGTAAAACACCAAAGACAACGAG
AAATGAAGATGATGAAGTTCCACAGCAGGCCAATGCAAAAAGAGCTAGGGTTTCCGTCAGAGTTAGA
TGCGATACCCCAACGGTATGATTTCTTCTTGTTATAAATCTATTTTCTCACAATGCTTTTGCTTTTGTTTTT
GTTTCATTATGTACATGTGTGTTGGGTTTTTGGTTTCTTAAAGATATTAACTTAGGATCAATATATACTTGA
TTTCCTCATACAGATGAACGATGGATGCCAATGGAGAAAATATGGACAGAAGATTGCAAAAGGAAATC
CATGCCCACGAGCATACTACCGCTGCACAGTTGCACCAGCATGCCCTGTGAGAAAGCAGGTAGGTCCC
CAAATGTACATACAAAATCATATATCTTCATAATATTATGAAAATATATGATTGAAAAGATAGATAGATCC
TTATGTTTTTGAAATTGAAAGATTGATTAATATATGTGTACTCCTTTTGTACATGATTTCAGGTGCAAAGA
TGTTATGAGGATATGTCCATCTTAATCACCACCTACGAAGGAACACACAACCACCCGCTTCCATTCACA
GCCTCTTCCATGGCTTCCACCACTGCTGCGGCAGCTTCCATGCTATTGTCTGGCTCCTCCACATCTCAG
CCGGGTTTCGGTTCCACAGCCACTCTACTCAATGGATCAGATTTTGGTGTCTTTGATAGTTCAAGAACA
AACCAGCTCTACTTGCCCAAATCCAACCCTTTGCTCCCCACAATCACTCTAGACCTCACTGCCTCTCCA

TCCTCTTCACCAATCCATTTGAATAGGTTGTCTTCTAGCTTTGCTTCAGCTTGCACATTCCCTTCAGGCC
TCAGCTTTTGTTCCTCAGAGTCCAACATTTCGCCCAACAGTAGGGGCAATGGATACCTTAAATATGGCT
CACTGCCGTTTGACAAAGGGTCCTTCAATCTAGATCAACCTTACGCAGAAAAGAATCACCAATCTTCTT
CTCAGGTGTCTTTAACAGAATCTTTAACCAAGGCAATCACGTCAGACCCTAATTTCAAATCAGTAATTG
CCGTAGCACTTTCATCCATGGTTGGAGGTGGTGCTGCTACTCATGGAAACCAAAGCGAAAGATTAGGA
CACCACTTGAAGTGGAGTGAAGCTGCTCATCAGTTTACTTCTCATAATCCATTGATACAGAACGGGAA
AGGATGCAGCACACCGAGCATTTTCAACAGATTGTCATCTTCAGACTCTCAAAAGTGA
>PmWRKY39
TCACACAAAGATTTGCTCTTCTTTTAGCTGTAGAAGTACTGGTCTCTGGTCATTGTTTCCGAATTCTCTC
CCTTTAAGAAGGGGATGCTTCTCTCTCACAACCGCCAGTGGTTTTAACTGTGATGCATTGTTATTGGCT
GTGTTGTGGCTGCTGTTTCTAGCTGCTGGAACATCATGGTTATGTTTCCCCTCGTATGTAGTGATGACAG
CTTTGGGGTCAGTGGAAGCTCTCTCAACATGCTTACGGACATTGCACCCCGCATAAGTGCATTTGTAAT
AGCTTCTGTTAACACATTTGTATCAGAAACGATGCTCATAATAAGGAAACCATATCTCAAGTAGAAAGA
AAAATATTAGAGAATAAATTATTCACATATTGTTTAAAGGTATGCTAAGCAAGTGACTCTCTAAGATCAG
AAAGTAATTGTTCTTTAATTAATGGCTTGCTCCACATCAGTAATCGAGAGTTTATCCCACTCCTCAAAAG
TTAATTTCTAAGTCCAATGGTTCATAATTTTACCTCCATCTATCAAATAGGATACACAATAAAAGAAAGT
GTGAAGAATTTTTAGAATACTTTATATCCTTTTTTAACTGGGGAAAAAAAGATGCCATCCTAATGCTTAA
TTTATAGGATCTTAAATATAAACATGTCAGAAACAATTTAGAGAAACCATCCTAATTCCATTTGCATAAT
GCAATCAGTAGAACTGAAAATAAGCAAAGATTCATATGGAGAAAGTTCCCACCTTGGATGAGGATTCC
CCTTGACCACCTTCTGTCCATACTTGCGCCACCTGTAGCCATCATCTAGAAGATCGACTTCACTCCTTGT
TTGCACAATGATTTTTGGTTCTGTCACTGTCTTGTGTGACAAAGCTACCTCAGATGCCCCAACATCTAT
GTTCCTGCACTCCCAGACAAGGAGATTTTAACGTTAAGACAAAACAAAGGAGAAAGTATCCTGTGATT
ACATGAAATGATTGGTTTTGAAAATACCTTCTCTTTGGATTTGGTTCATCAGCATCCCCCTCTTCTCTAA
TTTCCCCATCGCGTAGCTCTTCACTGTCACTTGCCCCTGGTAACTGTCCAGGAGCAGCTTGAGTAGATT
CTTGATGCCTCTCAAGCATTGAGTGAGAAGCTATATTTTCACTAGAACCGTTTGAATCTCCAACCAATC
TTTGCAATCCATTTTCAGGCCTAGGCTGCGAATGCAAGTGTCCGTTCAGATCACCACCATCTTTTCCAC
GTTTAGGTTGAGGGGCTTCATGGTTATGCTGCCCTTTGTATATAATCTCAGTTATTTCGCCATTAGGAGA
ACGCTCCACTTTCTTTTTGACAGGGCAATTCAGATGTGTGCATTTGTAGTAACTTCGAGGATACTCACT
GCCCTTAACCTGCTTCTGCCCATATTTCCGCCAGTTGTAGCTATCGTCTGCAGGCCTATCAGTGGCCACA
GATGAAGGTTGGTATTTCCTATCAGAATGAGATGCCTCTGATGATTGCCTCGCGGAACTTCTATGGTCA
GATGTTGAAGGTAATGTCTGCTGTTGAGATGCTTCATTGGGCATTACGGATGGATGATATGCCTGTGGCT
CTGTGGGAGCTCCTACCGACGAAGGTTGATATTCAGCTTGCATGTGCATACGAGATTGGGCTAGTGCA
GCTTGGGCAGTAACCTGCGCCAAGGCCTGCTGATGTGATATTCCAAATGGACTCTGCACCAATACGAG
GAAAAATTCATGAGTACCTGATGCTAATACAAGTCATCCAACTTCAGAAATCATACTATATTGCATAGAA
AGACAATATGGTATGAAGCACCAATAACAACATATGTTCCATATGGTCTGTCGCCAATGCATCACTATAT
TGAATGGCTTAGATTTGTAGATCACCTCTTTATCAGCTTCAAGGTTCTACATGTTTAACCATAGTTGAAG
GAGCCTTTAACAAATGTTACACGATGCAGAGTAGAAAAGATACTTACCTAAGCAAAATTTTGACTTTTG
ACAATATGTAACATGTAATAGGGGCATGCACATACACACACATGACACAACAAACTAAACAAATTCGTC
CACCAAAATTGGTCATGATTGTATCTTTGAAGTCCCATGCTACCCATGTGTCCTTTTTATATGAGACACA
TCATAGTTAGCCATCCTTAATCGGGGATTCTAACCACAGAGCAAAAATAAACTTATGACTGGAGTGAAA
TAATACAAACTAGGACCAACACTCAGGGAAAATAGGCATTAATTTAGTTGCGGCTATAAATGCACGATT
TTCACACAGTTGTTGTGAATGGCAGAACCTGAGGGCCTGATTTGTTGTTGGATATTAGTTGATTTCTTC
AAACTTTCCTTGTATACAACATAATTATGTGAAGAAATCCATCATTTCTTCAAACTTTCCTTGTATACAA
CACAATTTTGTGAAGAAATTCATCATTTTGCATGGGGTATCAGGAAGGGCCAGCCATTATCATCAAAAC

AAAATTGTTGTAAAGGAAGCACCTTTGACCAGCCCATCATTGAGTAATAGCTTCTGTTAATACTCAATA
ACCACCCCAATCATATCGTCAACCCACAAATGTACACCCTTTGAGCACAAGAAGCATTCCACAAGTAC
AAAAGAAGCACCTTTGACCAACCCATGTTCACAAAATCACACAAGTACATAACCATTGATCAACAGTT
ACTAATCAAACACACACAAATACCGAAAAAACAGAGCACACCCAGTATTAAATATAGCATTAGGAGAG
CTTTGTAAACTTACCGGAGGTGAAAAGAAGCCAGGCGAGTTAAGCAACCCAGAGGGACTCAATCCAG
GTGGAACAGTGAACAATGGCGACCGAGCCACCATCAAATTCATAGGCCTACTCTGCTTAAACCCAGAC
TTGTTCTCACCGCCATTTTCAGAGCCTCCTTCTAATTGGGCCGAACCATCAACTGGGTTTTCGTTAAATT
GGATTGGCCTTGTGGACCCCATAGGCGAGCCCATGGCTCCGGCCAGGAGCTGAGAGAAGGACCTGTA
CTCTGAGTCAGGGTATGTGTCGGGGAAGAAGCTTGAGACCAGGGTCATCGGACCCGGGCTGGCTCCA
GATCCGCCGCTGAAAAGGGCCTCAGCGGAAGGCCTCGGTGGCAGTGTTATGACTGGTCTTTGCGGTTG
GGGTGGTGCTGAAACTGAGACTCTCTCAGAGTCTTGGTTTTTGGCCAT
>PmWRKY40
ATGGAAGGAGATCAAAGAGGGGTTCCGAGTTATGAAGTTCAGATTTCGTTTTCGAGCACCCCAAACCC
TCAACAATCGATCCACGAAATGGGGTTTGTGCAGTTCGAAGATCATCATCCTCATCATAATCAGGTTTT
GAGCTTCATGGCCCCATCATCACATATACAGCAGCAGCAGCCTAACAATTCTCATCACCCTCATCATCAT
CAGCCAGCTGAGCTCAGCAGCGGTGCCTCCGCCGCCACCACCACCACCAGCAACGGGGTTACCGTGG
GGTTTAGTACTCATACTGACCTACTTGTTGCAAGACCTTCTTGGAATAATAGTAACGACCAGGTGGTTC
AATCTTGAATTTGAAAAGAAGCATGAAATTGGAGACATTTTCCTTGCTTTTCCATGTTATATATGTTAATT
TTTTGCCTGTCCAACAAATGGGTTATAGATTTCATCCAAAGAAAGAATCAGTTATACGCGTTGTCGGAT
TTTATTTTTGTAGTCTTATAATTGTGTCTTTTTTATGGAAAATTTAGGTGGGAACGTTGGATCCAAAGGCT
ATTAGTGATGAAAATGGGACTGGTAATGCTAGTGATTGCAGCAACTCATGGTATTTACTTACTTTATAGG
TTTTAATTTGTTCCTCTCTCTCTCTCTCTCTCTCTCTCTCTCTCTCTCTCTCTACCTAGCTAGATCTCAATCTC
TCTCTCCAAAGTACACACATGCATATGTACATGCACAAATGCGTGCGCACATCTAAGAACGCATTCTAA
AAGCTCTTCTACCAGAATCTTAGAAAGATTTAGCCACTTATAGAAGAGAGAGAGAATATTGCTTCTGTG
TTGCCATTTGTACGTCCTACACCATGTTCTTATATATTCTCTCTCCCTCTCTCTCTCTCTCTCTCTCTTTCT
CTCTCTGCGTGTGTGCCTGTGCCAGTCTTTCGTTGTGGCCATATGGCAGTATATCGATCAAGTACTTAAC
ACAGAGATTCCAATTATATAGATCCCACCATGACAGGATACAGATCTTTTATCTCATTTTCGTATCACACA
TTAATTTGTTCTATATATATATAAATATATTATTGTATAAAAGTAAATTAAAGAGATCAGGCAATCAATTAA
TTAGCGAGTAGATGAGTGGTAAATCTGGTTTTCATGCATCAATTAGCTAGTATATATGTATTAAGAGAGG
AGGAGCTTGAAATTAATTAATTATTTGACAGGTGGAGGAGCTCAAGCTCAGAGAAGAGCAAGATGAA
GGTGAGGAGAAAGCTTAGGGAGCCAAGATTCTGTTTCCAAACCAGAAGTGATGTGGATGTGCTTGAT
GATGGTTACAAATGGAGGAAATATGGCCAGAAAGTTGTCAAGAACAGCCTTCATCCTAGGTATACTTAA
TTTTATTTCTATGGCTAATTTAATCTTTCATATTTATCGTATACATCATAAATTGGTGCACATGCATTTCTAT
AGAGGTCCAGAATGTGTATATGGTTTGCACCAACTTTCAACTTTTTTTGTACAAGCCATATTAGGAGGG
AAGAAACGAACCTAAGACCTCGGGTGTAAGAGTAAATGTTTTAAACCACTTTTCAGCTACAAGCCCCT
TGCACACCAGCTTTCAACCTAGGTTTTACTTAATTGTTTACCAATGCCTAGGGGTACAGAAAATATATGC
AGGATATGATTGTCTATTTTTAGTATTTAGATGTATGCAATCGGTGACAATTATTTACAAATTTAAAAATT
AATGGTATACATGATTCGCAATTTTTGGCACCAATAGAAAAAAATTCTGAGAATACACATGTGAAATAC
ACTTTACATAACTGGAAATGTTCATCATTCTTGAAGGAAGTTGGAGTTCAAGTCCCAAAACCAACTAA
CAATTTGAGGAGTGAATCGATCCTTATAAACGATTGCAAAGTTTCTTAATTTTCCTATGTGGGACAATTC
TTCACATCCTCACAAGTACCACGTGCTTGTCTAACAAAGTTATAAGGTTGTTATCAATTCTAAGATCATG
TACTTTCATCTATGACGTGCCTGCGCCACATTATTTTTGCATCATCTATCTTGTGCGTATATATATGTTTTC
TTCTCAAAACCACAATTCATTGGTGTTGTATATATCAAGAAACACCTTCCATAATAGTTACTCCTCCACC
TTTTATTACTAACTGGTTTTGTGTTAGATGCTCTAATATTATTACGCAATTCACATATCCAATTCAACGGT

CACGTGCTTTAGCGTACTTGATTCAAGTTGTTTATGTATTTGACTCGAATCACGCCACATGTGAGGAGC
GGTGTTGAGAACGTTATCTCATATTATCACTAGTAAAAAAAGTGGCAACAATCACCATTATTTGGTGAC
ATTTGTGGCCGTGACAAAAGACATTAGTTAGCTTTTTTGGCTCGCATTTTTTGCTTTTTTTGGTGACTAA
AGCCTTTAGTCACCCTCACAACAGTCACCAAATGTCACCAAATAATGGTGACTATTACCACTTTTTTTT
TTTAGTATATATTACAAACTTCCCCTTTCATGCATATGTATATATTGTTTGTGTCTCTACCGATTTCCAATT
GATTATGAAAGGGTTTACGTGACATAATACTCTCAACAAGTCCGTCGGTTATAATCGTTTGCCACTCTG
AAGTATATACCACGTTGCATATGAGTGATATATTTAGAGATCATGAGAGGGTTTTAGTTTATTGTTGTTGT
GCTGAAACTGATGAATGGCATAAATATATGCATGCAGAAGCTACTACCGCTGTACTCACAGTAACTGTC
GGGTGAAGAAAAGGGTTGAACGACTGTCTGAAGATTGTCGAATGGTGATAACAACCTATGAAGGTAG
ACACAACCACACCCCTTGTGATGACTCTAATTCCTCCGAACATGAATGCTTTAGCTCTTTCTGA
>PmWRKY41
TCAGAACCTAGAGAGGAACTGCATTTGCTTCAGAAGAGGAGTTAGGGTTTCCATCAACTTCTCACAGG
GATGATTGTGAGTTCCTTCATATGTTGTCACCACAATGCTCGTGTCCTTAGATAGCCTCTGGACCTGCTT
TTTCACATTGCATGTGTGATGTGTGCACCGATAATAGCTCCTACATATGACCATGTTTGAAAGTATGAAA
GCGAGGAATGTGTAGAATTTTTTGAACCCAAAGAAGAACTTTGGCTCATAAGTAAATGCCCTAATACTA
ATAAAGCTGGAGATAGATAGATCATAGCAAACTTTTAAATTTATATATATTAATATTGCACCCAACATAAT
GAAGAACATGACTAAATCCTTGCTATTTATATAGATAACTCAACAGTCCCCTTCTATTGAGGGATCCCTC
AAATAATTTTATTTGAGGGACGCCCTTTAGGATACCCTATAATTTTTTTTCCAATGATCCAAACCATCTAT
TTTTTAGGTCCTCATTCATAGATCATCCTTACAAAAACTTAGACAAATCGGAAACCGTTTCGACATCCA
ATTGGGTCCTACAAAATCAATGAACACGAGTGCTTCAAGAAAGTACTAAAATTTCAATAACTCAATTG
AATAGTCAAATGATATCGGATTCAAGTGATTTTTTGTAGAGATGATCTTTGAATGTGTATCTACAAAATA
GATTGTTTGGATTAGTGAAATACAATCCGGAGTAGGCCCTATAAGGGATGTCCCTCAAATAAGCTTATTT
GAGGGATCCCTCAATGGAAGCTCTCTGGATAACTCAATATACAATATATATATATATATATATATATATTC
AAGTTGATAGGTCATAAGAATTTTTCTGTTTTCAAATAGATAAGTCTTTTTATTGATTCGGGAGTTCAGA
GTTATTTTTCTATTTTCAATCGGAGTTTTTATTTTTTGAAGTTTTCGATCAAAATTTCTAGGACCAAATAT
CTGAGTCTAAGTATTTATGTATTCAGTTTCCTTATTGGCTGAGGAGAAAGTAGAAATGCTTCGTTGAGC
AACTAAAAATATGAATGTATAAATTAGCTGGCTGAGGAAATATCAAATTAGTTGAGCAACTAAAAATAT
GAATGTATAAGCAAGAGGTTTATAGCTCAGGTGGTTAGGAACATTTACTTTTGCACCCGAAGTCTTAGT
TTCGATTCTCCCTTAAAATGGCTTGTATATTAAAAAAAAAACTATATGAATATATAAATGTTTTGTTTCTA
TCTCAAATTTGATGTCAGTAGCCAGCTAATTAACGTTTGCTTTATGATTTTGTTTTACAGCATCACATTTT
GGTGTCTCTCTATCTTTTTTCTTCTTCTTCTTTTTTTCCCTTCTCTTGTTCGGTGATGTGCTAAATTGTAA
CAAACAAAAAAAAAAAATATAATAGTGTTAGAAAGTGAATTAACAAACCTGGGATATAAGTTGTTCTTG
ACGGCCTTCTGTCCGTACTTTCTCCAACGATAACCATCATCAAGAATATCATCGGCACTCCGGGTCTGA
AAAGCAAATCTAGGCCGGCTCGCTGGCTTTCTCATCCTACCCCCACCTTTCCTTTTACTATTTGCACCCT
TTCCTTCCCGATCAGCTTCATTAATATTCTCAGCAGAGGCACCAACATTATTATTTTCCACCAGTGGCTT
ATAATTATTAATCTCCTGCCCATCAAACCCTGCTGCTGGAGAACCAGAGAGAAGGCTGACCCAATCAAT
GTCTGGGAGGACTTGATGCTGCTGAGGTTCTAGAAGAGGAGGGCTGATCAGCTGTGAGGAGGAGGAT
GGGGCTGTTGATGCTGATGAGGGTGTGAAGAGAGGGTTTTGGGGTGGCAACAAGAAAGGCAATGAGA
TTAAGGATGATGGGGTTAATGGGGGTGGATAATTAGGCTCTTGATGACCTTCCAT
>PmWRKY42
TCAGATACTGCCAGCGGCGGTCGCAGCATTACTAGAAACCCAGGGGACGCCAAAGCTCGTCGGAGAG
TGATCGGAAAAGTAATCTCCGGCGAGACCCTCCAAACCCACAAAGAAATCGTCGCTCACGACCGAAT
CACACATCCCAAAGAGCTCGTCGTCGTCGTCGTCGACCATAGGACTGCCCTTTTCTTCTTTGAAGCTTT
CCATGGTTGTACTCTGAGGTACAACAGGGTCTTCGTCGACTGACGCCGAGGTTGCCGGAGAAGTGGG

TTTGGTAGAGTCACTGCCCGTGACGGTTTGCGGCGAGAAGGGCTTCTGGCGGGTGGAGCCGGCAAGC
GAGTTGCGGTGAGTTGGGGCCGGATGGTTGTGCTCGGCCGTGTAGGTAACTATGAACATATTCGGGTC
GGATCTGTTCCGCTCCACTTGTTTTCGGGCCATACAACCCTTTGAGCTGCTGCATCTGTAATATCCCCTG
TTTTGGTTGAAACAACATAAAATTAGCCATTTTCCTTCATAGACAATTAAAATGGGTAAAAGCAATTTTA
TATAAATAAAAAATTTGCCATTTTTATTTAAGTTTCGGGAGAAATGAAACCCACCCACCTTGGATATGGG
GAGCCCTTGATTGGTTTTTGGCCATATTTACGCCATGCCCATATGTCTGCAGAGAGGCTCTCAGCTGGT
ACTTGGCACACTTTCTTAAGCTGGTTCTTTCTGAAATGGGAAAGAAACGCATAAATAACTTAAATTATG
GATTAAACAGCGCAATTTACGAGATGGGCATCTCCCAAAATTGAGTTTTTTTTAAGTTACAAAGAAAAT
CAAAGAGAAAAATTGTACCTTTTTTTGGATCGTGGGGTGGTGGTGGAGGAAACAGAATGAGATGATG
GCTTGGACTGTTGCTGTTGTTGTTGTTGTTGTTGTTGTTGCTGGTAGTGGTTTTGGTGTTGATGTTGTTG
AATGGGATGTGTTTGATCTTTGGGTGCAGTCAGAGGAGTGAGAGAAGTCAAAGGAGAGAGATTGGGT
GGTGGTGTGATTTGTGAGGAGAGGGGAGGCTGAGATTTGGGGAAGAAGGGCTTGTAAAGCTCATGCA
AGTCCTCCATGGCATTTCTGGGTTTAATGGGATCTGGAAGAGAGAAAAGCACTTGGAATTGTTGTTGC
TGTAGGCCAGGCTGCAGAGTAGCGGTTCCAAATGAAGAAGAAAAAGCAGGGCTTGAATGGAAGTTCG
AGATTTTGAAGCATGTGCTGTTGTTGCTGTTGGTGGTGGCTGTGCTTCTGTTGCTGGTGGTTGTGCCGG
TGGAGCAGCCTCTGACCACCGCCTGAAGATCCCAATCGTCTTCCATTTAGAGAGAGAGAGAGAGAGA
GAGAGAGAGAGAGAGACAAGGTTTCTGAATCTGTACCTTGTGTCTGCTAGCTATCACTGTTTGAGGAC
AAAGAAAGGAGGAGGCAAACAAAAGAAGGCGCTGACTTTTTATTGGTAAGAGAGAGGGGAGAGAGA
TGATAAGGGTGTGATGAGTAGGAGAGGAATTATATAAACAATGTATATATAGAGAGAGAGAGAGAGGG
ACAGTGGGATTTTATGCACAAAAGTCTAAAGGGTGCCTGAAAACAAGTGGTTTTCCTAGTGAGAGAA
AGGGCGAGAGGAAGAAGGCATCTCTATGCATTGTTTTGCTTTTGGAGAGAGAGAGAGAGAGAGAG
GAAAAAAAGAAAAAGGAAATGAAAAATGAAATTCAAATGGGGAAAACTGGTTTTCGGTTTTGGGGTT
GACTTGCTCAAATGTCAAAGCGGGTTTTGATTAAGGAAAAGTTCCACACGGTATATAGCGAGTGGAGC
TCGTGAGTTATACCGACAAAAGGAGAAAGAGATAATAAATTTAAAAAAAAAAATTTCAACTTTTTTTATA
ATAAAAATAAATAAATAAATAATGGAAAAGTGGAAGCCAGTTAATTTGGGCGCTCCTGTCAAAAATGCT
TGCTTGGTGGTATTGCAAAGATTTTTTAGTGGGTAGGTAAAGAATTACAAAAGCCTAAATTGCGCCCTC
CTCCTGCTTACGTGCCCTATCCGTAATCTTGGGAAAAATGACAGATTAGTGTTTTTATTAGGGATGTTTT
AACATGTAATTAAATGAATTCTGAATCAATCAGTTCAATCAAGCTTTGATTTAGTATGATCAATTTCCATG
ATTGTGGCATTTTATTAAGACCCTTCTAATTTAAGAAACCAATGCTTTGATGCTGCTTTGGCATTCAATT
AGGTCTCCAATTCATTGCTGCATCGCATCTAATTTTACCAAAATTTGTGGTTTGATTTCATGACAACTTT
GTTTTGTTTGAATAATTTTCTGTTTTGACAACAAATCAATCATCAAATACCATTTAATTCAATTGAAATTA
GATTGGATCCTAATAGTATTTCGAATATAAATCAATGAACACCCCCCCTTAAATATAATATGATCATGGTC
AATCCTAAATATCAAAATTGACTCACAAAGGATCCCAAAAGACTTAAAATTGTTCATCAATCTAATTTG
AAAAGTTTTTATCACTAGATTACTATTGAACGTAAAAATCTATGGTGTGACACCCTTGAATCATTTGAAT
GATTGGCATTCAAATTTGTTTCAAATCCCTCCAAACCACAATTGATTTTTCAAGGTCAATGCTGACCAA
AAAAAAAAAATTGAGAATTACTTACCCAATGATTCGTTTTTAGAGAACGTATTATAGCAAAGTTTTGAA
AGTACGAGAACTTATTTATTGAAAATCAAAAAATACAACTAAGAGGCATGAAACATGATTCATATTCAT
AGAATCAATAAACACAGTATCCTGAATAAAGTGAGGAACACATCTAACTAAACATAAAAGGATTGCAT
GATTTAATTAAGTAAAATAGTAGGTTTAAACTTTTCATAATCAATTCATTCCCCCACTTTTAAAGGTGTTT
TACCCAATTATTTGATTTTAATTCGATAAGGAAATGTGTTTGTTGTACTTGTGCATTGGGGCTGTAGAAT
TCCATTTCTATATCAAAAAGGAGCAGCAGCAGAAGCAGCATCTGTTCCCCTCTTTTTGGGTTTTGATGC
CTTTGTTTCTTCTGTACTCTCCCTGTCCCCACTTTCTTCATCAAAGGAGCACCTTCCCACGCCATAACGC
TCGTGACCGGCACATGGGTTCCAGGTAGCGGTGCTCTCAAGTTATACCTCCACGTATCGCCCGTCCACA
GCGAGTTTGTATTTCTATCTCCTTCGAGCGAGTACGGTGTGCCCAATAATGCCGCCATCAAGGAGTCAA

AGTCCAAGCGCTGATCTAACGGCTCGACGACCAAAGGCAT

>PmWRKY43

ATGGAGAAGAGGAAGAGCATGGAGTGGGAGCAAGAGACTCTAACCAGTGAGCTAACCCAAGGGAAG
GAGCTGGCAAAGCAGCTTATGAACTGTCTTCACCCTTCTGCATCCCAAGAAAAAAGAGACTTTCTGAT
TTCAAAAATACTATTTTCCTATGAAAAGGCACTCTCACTGCTCAAAAGGGATGTTGGTTCTGATGGAGA
GTACCACATCCCTAACACCATGTTGGAATCACCTACTTCATTTGGCAATGGCAGCCCAATGAGTGAGAT
CTCTGACCAAGATTGCAAGAACAAAAATGTCTTCAAGAAAAGGTATTTTTTTAACTTGGATTTTAATTT
TATTAATTAGGGCAATTTTGGATTAGCCTAATTATGGTTGAAATTGATCTGATTTTGACTGTTGGGATTTC
TGTGTTGCAGGAAAACAATGCCCAGGTGGACTGAAGAAGTGAAGGTTTACTCTGGAACAGGGCTAGA
TGGGAGTCTTGATGATGGCTACAGTTGGAGAAAATATGGCCAAAAGGATATCCTTGGAGCTACCTATCC
AAGGTGAGAACTTTTAGTCTTTAAAGTTAGGAGTTTGAACCATTACATGTTCAATTTACATAATTAATAC
AAATAAAATAACACAAAAACCACCAGTGGTTGGATTGGATGCTCCACCAGTGAAGCCTCTATTTTGCT
CATAGTGAAAAATACCTTTTTCTATTTATCACATGAGCCTGATTAATCATTGGTCAGACATCAGCTCTTG
TCCTCAAATCTACGCAGCACGTCAAAAATTAGAGTTCTTATTACTGCTAGGATGAAGAGGCTATTTTTT
CCTCTCTTTTTTTCTTCTTCTTTCACAAGTGGCATAATGAAGACAAAAAGTACTTGAACTTATCTAAAG
GTGACTTGTATCTGGCTCCGTAAGCTAGCAGTCTTTAAATCAATTCACTCTATCTAGTTGTTTGTGTTTT
TTTGTTTGGCCTCCAGGATTGACAATTCTTCAGCCTAGACAGTACCTTTTAGCATTAAAAATAAAAACG
CCATGTCCTGGCAACCGGTCCAACTCCCAAAACGGGTCCTATTGGCATCTTGTGTTAAGTTTGCTTTAG
CTCAACCGGGACCATGTTGATATTTAGTGAAGAAAATGTCATGTAACATTTCACTGTTGATGAATTTGG
TCTAAATTGCATGTAAGATTTGCAGAGATATTCATATGAATGATGTTGATGTCCCATATTTCTCAAAAATC
ACCTTTTTGTTTTCTTTGTATTGCAGAGGCTACTACAGATGCACACATCGCGGCACGCAGGGTTGTTTA
GCCACCAAGCAAGTTCAAAAGGCAGATGCAGACCCGTCAACCATGGTGGTAACCTACAGAGGAGAAC
ATACTTGTAGCCAAGTCCTTCAGTTAGCCAGGTCATCAGTATTATCCCTAGCTAAACAAGCTTCAACAG
GAAATCAAAATGCAACCCGAGAAGTAGAAAAACCAAAAGCATCAGAAGAGATGTCTTTCAGTTTTGG
GGCAGGGCTTAGAGTTAAAACTGAGGATTTGGACACAAGGGAAGATGATATATTTCCATCATTTTCCTT
CCCTTCTACACCAATTGAACCTGAAAATGTCGGAGACCATATTTTCTGTGCAACTTTGATGGAGAATGA
TTTGATGGATGGCTATTCTCCCACATTTGCATCTCCAGCAGCAACATTTGAACCAGACTACTTGCAGGC
CACGAGCAGTTTTGGACTTGGCCTTGATTATGTGCAGACTTCAGAATCTGGTCTCAGTGAGATCATCTC
AGCCCCAACTTCAGTGACCAACTCACCAATTGGGGATTTTGGTTTCTCACTTGATGATTTGGATTTTCA
CCATTTTGAAAACCCAGAAAGTTTTGCTTACGAGTCATAA

>PmWRKY44

TTAGAACCTAGAGAGAAATTGCATTTGCTTGAGAAGAGGAGTAAGGGTTTCCATCAGCTTCTCACATG
GATGATTGTGAACTCCTTCATAAGTTGTAACGACGATGCTTGTGTCCTTTGATAGCCTCTGAACCTGCTT
TTTCACACTGCATGTGTGATGTGTGCATCGATAGTAGCTCCTATATATGAAAAATATTTGATATTGAAGTG
AGAATCAAAGCAATATCTATTTATTTGTGCTGTGTTTTCCAAGAACAAATTATAATATATAGCAATTAAA
TCAATGAATAACCTGGGATACAAGCTGTTCTTGACGGCTTTCTGCCCATATTTTCTCCAGCGGTAGCCAT
CATCAAGAATATCATCGGCACTCCGAGTCTGGAAGGCAAACCTAGGCCTGCTCGCTTTTTTCATCACAC
TAATTTTCCTGCCCTCACCACCTTTCCTTTTATTGCTACCCTTTTCTTCCTGAGCAGCTTCAATAACATTA
TTTTCTTTATTATTAACCTCCACCATAGCAGCAGGCTCATTAGTATTAATCTGATGATCATGATGACCAGA
GAGAAGGCTGATCCAGTCAATGTCTGGAAGCTGGGCTTGCTGCTGATGATGAGGCTCTGGAAGAGGA
GGAGGGTTCATTGAGGTTGAGGAGGATGGGAAAAGGTAGGAAGGGTGGGGTGGCCATAATGGTAGTG
ATGACGATGTTGATGGAGGTGGATAGTATTGCTCTTGATGACCTTCCAT

>PmWRKY45

ATGGATGAAACCACCATTTCTTTGATCCTTCAGGCTTGTGAGTTAGCCAGAGACCTCGAATCAAACCTA

CACAACTTGGCCAACCAGCCCAACTTGCTCTCAAACTCCTTGGATGAGATCACAAAGAAATTTGTTAC
AGCCAGAGAGAGAGTGTATGGTCAAGATCCAAGTACTTCTTCAAGTCTGCACAACATGCTTACTCTTG
TGCACCAGCAGCAGATTGGTACAAGTCATGTACAAGAATGGCTGAGGTCTAGTTATGCAACCCAACTT
GTAGCTGATCAGAAGGAAGCTAAGATTGGAGGCTGCATCGATGACGATGATGCAGAGGTTAAGGGCCT
TCAGGCAATGGATATAGTATCAGCTTCTGACACGAACATTGCTTCATCATCCTCACAAAGAGCAAGAAG
AAGGTAACTGTGTAGTCTTAGATTATGTAAATTTTCTATTTGATCTTAGTTTGCACATGCAATTAAAAAC
AATGTCTTGAATTATTGCTTTTGTCATTTGAAACAACCTTTGTAAGTTGCGAAGAAGAATTAAGGAAAA
TGTGCAGAAAGCAGAAGAGCATTACCAAGCTGTTTCTCAGAAAAAGACCCCTATATAAAGGCATATAT
GCTATTTCTGTCTTTTACTGTGTGAACCAAATACATTTTTCTAACTGTTTTGGAAGCATGCAAATGTTTG
ATGTTTTACAGGCACAAGAAAAGCTTTGTTATAATTGCTAAGCAAGTTCACATATTGTCTTTCTGGACA
GTTGGGCTAACAGCTTTGTGTTCATTTAATTAATTAAATAGGAAGGATCAGGGGTTAATCAGCAAAATT
ACAGTTCCAGCACCTCGGATTGGAAATACAGAAATCCCACCAGAGGATGGCTTCACTTGGAGAAAATA
TGGTCAGAAGGAGATCATGGGCTCCAGGTTCCCAAGGTAATTAATTTTTTTTCCTTTTCATATTGACATA
AGGAACTTCACCCGAAGTTTTGTATTCGATTTCCCCTCCACCGATATTGTTTGTCTAAACAAAAGGATC
ATATTCAAATGGATTGAAGGTTACAGCTTATTTGACACATTCAACTCAAAAATATACCAGCTTATATTATA
TATATGCCAGCTTGAGTGAGTGATTATCAGAGATTGGAGCTCTTTGATTCAAGTTGGTACAATTAATTAG
TGTCAATTTTTATTTATTTTATTTTTTATGCTAGCTAGCTTTTACCTTTGTTTATGATTAGCGGAATGAAAA
ATTCATGGGGTTGACCAAAATTGACTTGGTCAGCATTCAAATAGTTTTGACTGGAACTTGGCAAATGGT
CTTGAATTCCACTGATCAATCAGCTCCTTAGTAAATGCGCAGACCTCTGTCCAGAGTGGAGCATTGACT
TGAGAATGTTCCATTCAACTAGCCATATATATATATATTGTCTATATTAAATGTCGGGTCATAATTAACTAC
CATGAATTAATTAAATAATTAACCACACATCTCTTTCAGGAGTTACTATAGGTGCACCCACCAAAAGCTC
TACAATTGCCCAGCCAAGAAGCAAGTCCAGAGGCTTAACAATGATCCCTTAACATTTGAAGTAATGTA
CAGGGGTGAACATACATGTCACATGTCAGCCACTGCACCTTCAATTCCACCACCATCAGCAGAACATC
ATAATGCTACACAAGAGAGTATGGCCCAAACCCTAGCGACTACCACAACCGCTGACCCTCCTACAGCA
TCTCTATGGCTTTCCATGGACTTTAACCCAATTAGAGGAGGCGGCGGCAGCAGCAGCAGGATAATAGG
TGGCGACCATGGCGGTGGTGATGGTGTAGGCACATCCACCACCACGCGTTACGGTAAAGAAGTTGACT
TTCCTGTAGTGGATTTGGCTGATGCAATGTTCAATTCAGGCAGCAGCAGCAGCAATAGTATGGATTTTAT
TTTCCATTCTGCTGAAAACAAATGGGAGTCAGAG
GACAAGAAAAACTGA
>PmWRKY46
ATGGGAAGCAACCATAAGAGACTGATCAAGGAGCTAGTTGAAGGGAAGAAGACGGCGGCTGAGCTTC
AGATGCTGCTTCACAAGCCCTTTGGAGATCATGGGTCGGCCTCAGCTGAGGAGCTTGTGGTGAAGATC
ATGACAAGTTTTACAGAGAGTCTCTCTGTTTTGGCTGCTGAGAAGAAGAACCCAGGTGATGGCCATGA
AGATCACCAGTCTGGTGCTTTTGGCGAGGTTTATCAGATCAAACCGGAGCCTTCCCATTCACATTGTGA
CGACCGGAGCTCTGGAGATTCCGGCGAGAGTAGGAAGGTGCAGGGTTCCAAGGATCGGAGAGGTTGC
TACAAGAGAAGGTACTGATTTGAATGATCTCTCATATCTGAATAATTGTTTGCCAGTTTCTGTTTTCTTC
TTCTTCTTCTTGTGGTTTTGTTTTTTTGGGTTATATTGTCATTTTACTAAAAGATTTGGAAATCTCTTGCC
TTTTGGGTTAAGGAAGATATGTGATATGACAAGGAGTTGGATATTTATGCCTAGTTTTCCAGCATATTTG
TTGAGCCTTTATCTTTGGGACTTTCTTTTATAGCAAATCTCAATTTTCTTAACACAGTTAATTAGGTGTTT
CACTGTTTTTGCTTCTGTGGTTTGACCTGGCTGTTTCATCTTCTTGGTTGCAGAAAGACTTCCCAATCAT
GGACAACAATCTCTTCTGCAATTGAAGATGGCCGGGCTTGGAGAAAATATGGCCAAAAGGAAATCCTC
AATGCTCCATATCCAAGGTTAGGTTTTACTTTTTTTTACTCTAAAAAATGAATAAATTCCCTTTTGGCCCA
TGCATCATCTCACTGTTAAAAATATAAGTTAGCCCACCCCTTACACCCAAGTTCAAATTCCCTTTCCAGT
AGACAAAGATTGTAACAGCTTAGGGCCGGGTATGAAAATATCATCCCATCTCCGGCCCCATCCCATCCT

AATTCCCGACTCCGTTCCGTCCCATTGAGGGACTTGTCTAAATCTACGGATTAGATTAAGTTAGAGTATA
TTATCACTCGTATAAAAGAATAAGGGAATCCGAAACTGCAAACGAAAGGAAGGAATTGTAACAACCA
AAGTTGCGTTCCCATTTCAGTCCAAAGAAAAAAAGTTGGGTTCACATTACACGTCTGATGGGATGACA
TACACATAAAGTTTATCTTCTATTTTCCAAATACCTTAAGCCACGTAAAGTTAAAATAGCAGGCCCCAGC
AATTATTGCAGCAGTGCTTATTGAAAGCCATTAAAGAAACAGAGGAACCAAACATGCATAGACTTTTAT
CAATAAAGAACTTTACAAAATTCCATACCTAACAGAAAACAAAAACTGAATTCTATTTATGCTTACTGA
TTCTGTGTTGGGATTTTTTCCTTTTTCTTTTTCTCTTGGTAATTACCACAAAAAGCCTAACATATTTATCT
CATGGACTTTGGTTGACAGAGCTTACTTCAGATGCACACGCAAGTATGATCAGGGCTGCAGAGCAACC
AAACAAGTCCAGCAAGTCCAAGACAACCCTCGCCTGTACCAAACCACATATATTGGCCAGCACACATG
CAAAAGCATGGTCCCTCCACAGATGATCATAGGCTCCTCTGATCATTGGGAATCTCAGACTGTGAGCTC
AGAATCCGAGACCCCAAACAAGCAAAACCATGATTTCCTTGGCTCATCAGCAATTCCCATTGTAAAAC
AGGAAGAATACAAGGAAGGGACACCAACACCAAGTGACCTAACAGACAACCTTTCCTCATTGGAGAC
TAATCATTTGTGGTCTGATTTCAAGGATGATTTTGCTTTGTGTGACCCTGCAGCAATGTGTGTGTCTACC
AAAATGGGGTCTGATAATGAGGACGTGGTTTCAAACATGTACTTGGACATGGATTTCGTAGTCAAGTCT
ATCGATTTCGATCGCGATTTTAATTTTGATGAAGTTGAATTTCCTAAGAACTCCTTGTAA
>PmWRKY47
ATGGAGAACTGCAATACGCATGGGGAGAAAATGAGTCTTATAAATGAGCTCGCACAAGGCAGGGAGC
TAGCTAGGCAACTCCAGATCCATCTCAATGTTCCGTCTTCCTCTCATGGAACCCGGGAATCGTTGGTTC
AAAAAATCATAGTTTCATACGAAAAGGCACTTTCCATGCTCAATTCTAGCAGCCCAGCCTCAGGGGGA
GAGCAACAACTGCCCACAGGTCATGCTGCGATTCGAATGATTGAGTCCCCCCCACCTTCTCTCAATGG
AAGTCCCCGGAGTGAAGACTCGGATCGCGAATTCAAGGACCAGGACAACAAAGATTCGTCCAGGAAG
AGGTAGTTTGTCATGATAATGTTTTGTTGGTTTATCAGTACTTGATTGAGATGATAATTCTGTGGTGGTTA
ATTTTTGAATTTTCATAAATGTTGCAGGAAAACTCTGCCAAGGTGGACACAACAAATAAGAATTACATC
AGGTATAGGGCTTGAGGGGCCTCTTGATGATGGCTTCAGCTGGAGGAAATATGGCCAAAAGGACATAC
TTGGAGCCAAATATCCAAGGTAGGCCAGGCAGAACATTTTCATTAAAGAACCCCTTTGTGACTTGGTC
AACTCAACCAATGTGCAACTTGCATATTAACATTAGATTGTTGATCTTGGCAGAGGCTATTACAGATGC
ACTCATCGGAATGCTCAATGCTGTTCGGCCACAAAGCAAGTGCAACGCTCCGATGAGGACCCAACAG
TCTTCGAAATTACTTACAGAGGAAGGCACACATGTACACAAGCCTCCACCAGCATAACCAGCGCTCCT
CCTCCTCCTCCTCCTCCTCCTCCACAGAATAGCATGGACATAGTGGACCCTCAACAGAATGAGCAGCA
ACCACAAGACTTGCTCTTAGCCATCCCACAAGGCCTTACAGTAGTAACTGAAGGCTTAGACGCTGGTG
ATTTTTCTCCTCCATTTATGGGCCCTGCAACTTCTGGAACACAGTATTTCTCAGCTTCAGAGCAGGGCT
TTGGAGGAGGCAGCCAAGGTTTTCAGGGTGCTGAGTGTGAGATTGCAGAGATTCTTTCATCTTCTACT
TCAGCAGCCAACTCTCCTGCTGCTTTGGGTTTCCCATTTGGTCAGGCTGATCAAAACTTATGCCCCAAC
TTCTCATTTGACGGTCCAGGCTTCTTTTCCTCATAG
>PmWRKY48
CTAGCAGCCTCCGCCAACGGTGGCTCCGGCGGCCCAAGAAGACCCCAAACTTGACGGCTCTTGATCC
GAAAAAGTATCACCAGAAGCACCTGCCGCCAGAGAACCCCCGCTACCACTAGAACTAGTACAACCCA
ATTGCTTCAGCCCCAAGAATATCTCATCCGGCATGGCTGTGTTCGGGATCAGAACATCGTCCTCCTCGA
CGTCCTCGTCCTCTATCTCGTTCTCATCCCTTTCCTCCTCCTCATCAAGTTCCTCATTTTCTTTGTCAAGT
GAATTGTCAGAATTGACGTTATTGGCAACAGCGTCTTGGTCAGCTTGGGCTGGTGGTGAACCCGGGTC
ATTGTTGATGGGCTGGTTTTGACCCGCCGTCGCTGATAACTTGTTTCTGGTGCTGCCAGCAAGGGAGTT
TCGATGCGTTGGACGAGGGTGATTGTGGTCGCCTGTGTATGTGACGACGAAGATGTTGGGGTCCGCCG
TGCTCCGCTCCACTTGTTTTCTCGCTGAACATCCTTTGGAGCTACTGCAGCGGTAATAGTTCCTGTGGA
GACGCCAAAAGAGAAGGGAAAAAGAAAATAAAAAGAAGCATCTTTGGTATTTCAGAGAGGAAATCG

CAAAAGGAAAGGAAAATCATTGCAGAGAAATTAACCTAATTTACCTTGGATGAGGAGAACCCTTGATA
GGTTTTTGGCCGTACTTACGCCATGCCCATAAATCGGCGGAGAGATTCTCTGCGCTTACTTGACAAACC
TGTCTTTTCTGCTGACACTTCCTGTGAACCAGTTGACATCAAAAGCAATTGAGTAAGAAGAAATAATAT
AAAAGGGAATCTATACTGCAAATATACCTTTTTCTTGGCCGGGGGGAGTTGATTGGCAT
>PmWRKY49
TCAAGAGGGGACAGAGGGAATCCTTTCAAGGGAGAGATTGCTCCTCATGAAGCTTGGGAAAGATGAA
AATGTGGGTAGTTCTTCAAGCTCATCAAAGAAGTCATTTTCTTCAGATTTGGGTGTTGAGAAGCTCATG
AGCTGGGAATAAGAGAGGGGCTCTTCATCCAAGAGGAACCCAAGAGTGCTAGAGACATGGGTTTTCT
CTAGGAAAGGGTCTTCTTCTTGTTCAATCATAATATTTTGGGAAGACCTAATTGGGGACTGAATGTAGT
GAAAATGATCTTCATGATCACTTTTCATTGCAGGATGGTCTTCTAGTTTTCCTTGTTTTTCTTCTTTCTCT
TTCTCTGGCTCTTTCTCTTGCTCTTCTTTCTCTTGCTCTTCCTGTTTGGGAAGTAGGGCTGGGAGATTAT
GATCTTCAGTGCTGGGTTGCAATTGGGTCAAGTTAGTGGTGGAGACATCAGGGCCTGGATGATTATGG
CTACAAGTGTAGGTGATTATGAGCACTGAAGCATCAGTTTTGCTTCTCTCCACTTGTTTTTTGGCAGAA
CAACCCTTTGATGTACTGCACCTGTAGTAGCCTCTGCATCATCAGAGAGAATTAAAAAGCAGCAATATT
TAAACAGAGAGTCCTAATTGTTAATCTCATTTCTGAAATTAAATACAGCCTCGCTTGCAAATTCTTGATG
ATATGTGATCAGAGATGATAACATGCATGACTACAAATAATGAACTGATAACTTTTTCTTTGGGGACTTT
AAGATTTAATTAGAGGAAATTAAAAGATAAAATGTGAGGCCATGTAAGATGTTAAAATTAAGAGACCAA
TATTGATCATGAGCAAAAAGAAAAAAAGAAAAAAACCTTGGATAAGGTGATCCTTTGATAGGTTTTTG
CCCATATTTCCTCCACAGTCTTCTCATGAACCACCTTCCTATAACAATAAACGTATTTATGTAAAAAAAA
AAGTAGTACTTATATGTAAGTTTTTTTGATGAAATATACACACACACACACACACACACCTTCTTTTGGA
TGCCTGACCTTCTGGCCTGAGTTCTTTTGGAACTTCTGAGTCTGGTTCTGGAGAGAGGGTTTCAT
>PmWRKY50
TCATGTCGTTGTAGATTGTGATGGTATCCTTGGGTGGTTATGCTCACCTTCATAGGTAACAATAAGCATA
GAGGGTTCTTCCAAGCACCTTTCAACATGCTTCCTTGCAGGACAACCTCTCATGCTACTACATTTATAGT
ATCCCCTGCAAAAGTTAAAAGGAGTTTCAACAGTTAATTCAGTACCAAATAATTTTAAACTTAAAAGTC
CATGATCTTCTACAAACATAGTAGTATCGAATAAACTCAAAAGTGCATCAACAAGCATTCTTAAATATCA
CTTAAAGTCATATTCAAAATTTATTCTTTGAACAAATCTTGACCCCTACATTTGAAAGTGCATAGTATAG
AATAAACTCAAAAGTGCATGCTAGTATCGAACAAACTCGAAAGTGTAGTAGTATTGAATAAAGTTGATG
TGCATCAACAAACATTACAATATCAATCACATATCATCCTCAAAATTGATTCTTTTAACAAATCTTGACC
ATCATTCAACTGTGAGAGTCTGGATAAAAATTTGAACAAAAGAAAATAGAATCTGACAACAACCAAAC
ATGCATATTGCAACATGCAGGATTGCTAGGAAACTGAACTTTTTTGGCCACCATCTCTTCTCAGCAACT
TCAAACCACTTGACACTAGATGTTTTTCTATTGAAGGTCATAATTAGCATACTTGATAAAATAAGTAAAT
CCAGGGCTATTCCACATTCCCAACGGAGCCTACTAGACATTACATCTAACAAAACAATCCAGTGGCATC
AATTTGAAGAATACTAACAACAGATAACCCACAAGATGACTTGCTGATTACCAAAGATATCACAATCCA
AATGGTCAATCACACTATCTGATTTTTGAATGGGAGAATGCATGCCTCTACTGTGTATGTGAAGAAGTT
AAATAAAAAGAGTTAACAACACTAACATTCGCAAAAAATAAATATTCAACCATGCTCATCAACTTATTT
TGCATTTCAAGCTAAATTCCCGTAACTATCAATAAGATTAACTAAAATACTGCAATATTAGATATAAAACA
TTGAAACAAAATTTTATGCATCACAATCAATCAAGTTCTCATTTCAAAGTGCAGAAATATATTATACCTA
GGGTGAGGAGAACCCTTGATTGGCTTCTGTCCATACTTCCTCCATGAATAATCATCTGGAGGGATATCT
GCAAGCTTGTTACTAATAGCAGGCACCTTAATAGATCTTTTCACTCTATGTTTCCTGCCAACAGAACATA
ATTAACAAAAAGTAGTTATAAACAAATATAACTGCTCCTCCATGACATGAAAGAGAAACATTACAAGAT
ATTTGTTGACAAGGTTTAGATGAACCAAGTAGAACATAAGGAGATAAATCCCACCTCTTCTTTGAGCAG
TGACATCTACCACTGCTACCACATTTCACACTCCCATCGTCTCCCCTTGCAGAACACTTCCTCTTATGTT
GTGAATTCTGATCCGAAGAGAGAGGAGCCCCAATCAAATGGAAGGAATTACCATCAAAGTTAGCCACA

CTTCCATCTATGCTCAAGGAAGAAATGAAGGATCTTGTAGATGACATCGTAGGTGTGCAGCTAGAGCTA
TCAAAATTCAGGTTTATGCCACTATTGCTCCTACGGTACATCATTTCTGCTTGGTGTTTCATCTGCTGCT
GCTGCTGAAGGAACAACCTCTGCTGTTGCTGTTGCTGTTGTTGCACTTGCTGTTGCTGCTGTTGTTGCT
GAAGGAAATGATACTGTGTTGGAGGTGCTTGTTGCGCCGGTTGAAGCGGATTTTTCCCATTAGTGCTTA
ATTCCAATGATGGGTTTCCCAAACACAAGGAATTTTTAACATTTAAACCCAAATCTTGAACTGGGTTTT
CAGGAAAGCTAGACTGAATAAAGTGAGGAGTTTTGGATGGATAGTCTGTAATACAATTGGGATTATCTA
AGAGGATTCTTTCAGGAAAAGGAATTTGAAGCTTCTTACGCTTTCTAACTCTAGCATGACCTAAACCAG
TATTGAGAAGGGAAACAACTTTCTTGAACTTAGACACGGCCTTCCCAGTTTCCACCATCAAATTCCTAT
ACTGAACTTGATCTTGGGTCTGAGAAAAAAGACTTAGAACTCTATGGCAGCTTTCAACAGCTGCTTTG
TTAGCTTCTTCGACGTCCTCCAT
>PmWRKY51
ATGGATGCCTCCTCTGGTTGGATCAAGCTCAACTTCGATGGATTAGTAAAAAATGGAGTTGCTACCACA
GGATTTGTAATTAGAGATGACAACGCCCACACCCTTCTCGCTGGTGCTAAAACAATTGGAGACAATTC
CATTGCTGTGGCTGAATGCCTTGCTCTGAGGGATGGTCTAGCCCATGCAGTTCATCATGATTGGCACAA
TATCATAATTGAAGGTGACTCCAAATTGGTCATTGATGCTATTAACAAGAAATGCTCTGCCCCTGGAGTA
TTATGCAGCTTCTCCAAGATGTGTCACACTTGGCCTCCTTTTGTAATTTGATTAAATTCCAGCATGCTTT
CAAGGAGTCAAACTCGGTTGCTATGCTTTACCTTCCTTAGGACACTCCTTGACCCCCCTTCCAAGCTTT
GGTAGATAGTCTTCCTCTTTCTATTGTTAGCTCCTTTTACCTTGACCTTTTTTGGGCATGCGTGCCCCCAT
GAGTCAAGGTTATTGTAAATTTTTTTTTTTTCTCATAAAAAAAGGACTTCTTTATTTATTTTTAAATATGG
AAGTAACTTAATCTCTTACTCTTACTTTTTCAGACATCCATAGCATCTTGTTTTTGACACAAATTTTAAAT
CATTAAATTTAGGCATTGGCTCAAATTAAATCTCACAACTAATTTATTAATATTTTTGAAATAATACATTTA
TTCAAATTATTATTATTTTAATTTAATTATTATGAATTGCACAGTTCCCCAAATTCTACATCTCTCTCCCTC
CCTGCCTAGCGTTGGTAATCTCCTCCATAGCCTGGATGTGCAAAAAATCTCCATTTGCCCGGTGTTGATT
ATTATTTTAATTTAATGATTCAAATTTTTTTGTGTCGGAACTTGTGTCAAAAATATAATGCCACGATCCGC
CTCCTTAAATATTTCAGTTAAGTCACATTGACTTGGAAAGAAAAAACACAGGTGCTAGGAAATTATGCA
AATCGCGTTGATTTGGCTTTTAGTGACAGGAAACTGCTTCGAAAAGATGACGCCGAGTAAGCAAGTTA
CCTAATATATAAGTAACATGAGTTTGATTTTTATTACAATCAATCACCTTCACCACTGAGTTAGGAATTTG
AAGAGTCTGATTTAATTTTTTATTTTTACAAGTGAAGCAAGCGATGGAGAGCCATGAGCATCTAAATAA
CAACTTTGGGGCAGTCAACTCTTCGGGGACTATAATGGAAAACTGGAAGAGGCTATGTGCCATGCTGC
AGAACTCCAACGAACCGCCTGTCGTCGTCGGTGATCTTCAGCGCATTATGCAGGTTTCACCTCTCCCTT
CTCTTCAATTTTGCTATCTCCCACGTTATTCTTTTTTGTAAATAGTATGAGAGAGTGAGAGTGAACAAAT
AATCTATTGTTTGAGACTTTGTCAGTTTGCATTCCACTCATTTATATTATATTACAAATTACAATGAGATAT
GGACAAACAATATACAAATATCATCCACCAATCATGATAGAGACCATTGAGCATGTTGGAAAGATTTGA
CCAGGGAGGGAATGTCGGTTTCAACGGCGTGACCGGATAATATGGGGATCTGCCATATCCGAGTTCGC
CGGTTTCTTTGGTGGTGGCAGTGTCGCGAGGAGACGAGTGGAACGTCGTCGGCCGTGCGTGGCAGAG
GTTGAGATTTTTCCGACGGTGATTTCACCAGTTTCTGTCATCCGATGACTCTGTTTCTCCTGGATTTTTC
TTGTTGGAGCTTGGTGGCTCCAATTCCACCCTTTTTTTTGTGGTTGATGAGTCCAATTGCACGAATTTG
AACACGAAAATGGGTTGTTAATTGAGAAATTTGGGATTTTGGGTTGGAATTGATTGACGTCGTGGGTTT
TGTTCATTGAAACCCCAACGAACGGGGAGGGGAAGAAACAGCCCCAGAACAATACAGAAACACAAA
TATACCAACACAAAGGGAAAGGTCAAACACAAATATACCTCACAAATTTTTATTTTGCTTTTGTCATCAT
GTAATATTTGTTGATGGAGTTATCTCCCATCGAATTTTTCTTTTGCTTGTGCCATGACATTTTCCTGATAA
ATCTCTTGTGTCCCCGTGCTCTACCTAGTTTTGAATCTTATTCTGAGGCTACCTCATTCTTTGGGGACAG
GAAGAATCAATTGAAAGCAAACAGGGAGCACCGCGTCTCAAAAAAAACTTTTGCCAAGTTTATTCCT
CAACAAAGATGACAGGAGCCGCAGAATCCAAATTGGTACCAATATCAGAAGATGGTTACAGTTGGAG

AAAATATGGACAAAAAGGAGAGAAAGGTAATGAAGGCTCGACGAGTTATTACATGTGCACATATCCCT
ATTGCAAAAGAAAGAAAAAGGTGGGCAGATCACTGGATGGACAGATTACTCAAGTAGCATACAAGGG
AACTCACAACCATGAGGTTTTATTTTTTGACACTATTTTTTTTTTTTTTATGTTTCACTAAAAATGATGCA
AAGGCAATGGATGATCTGTGACCTTTGTTGGGGTGGCCATGGATTTCATCTGTGACCTTTGACAAAGA
GGGTTCTGGCTTTGTACAATGCTAAAGAAAATATGAACGGCTGGAAATACAGTTATAAACAGAGAAGA
GAATTCAGAACCTCTGAATTATACTGGTCAAGAGTGTGCATGCTTAATAGATGATTAGACATTACAGCTT
CGTATTCCAGATACAGGGCTTGTCGCTTTTCACACTATCATTGCATATTGTAGGAGAAGCGGCATCTGCA
ACCTTCGTTTCAACTGGAAGGCTGCAACTCAATTTATAACTGAAATTTTAATTTGTTATGTTATTTCAGG
AAAGGAAGGACATGAATCAAAGTCTTTCTAGGAGTGTGTGGGTAAGCGCTCTCACTGCTATAGAGGCA
TAAGCTCCAAACATTAAATTTCGTATTTTTGGTTAATTTTCTTAATTTGGTGGTTTCATCCCTATTAAGTTA
TGTTTAAAAAGTTTTATTTATTTTTAGATGGTTTAGTTCTAATTGAAGTATACTTTCATTGTAATTTGATTG
GCATATGACATGTCTCTGCATTTGCAAGACATTGTATTTCGAGTTACAAGGCTTTGGCTTTCCCCTGATC
TTTGCATGTTGTAGGAGTTGCGCGGCGGGGCGCCTTTGCTTAGCATTACTCCAGATGTCCTACCTAGCA
TCGGCTCCAGTGTGGACAACGTCGACCATGAAGAGCCAATATTGCATGAGCAGAAACATGATCATGAT
CAACCAAGCCACGAGAACACTAACTATGGCAAGCCAAGGCAGGCGACAAAGGTAGTGATCGAAACA
CATAACCATATACTAGGGTCATGCTGTCTTATTATACTTATGTTGCTACACTGTGAATTTGGGCTATATATT
CATGCAAGAACCAACTTGATCACTGGTCCAGATTGACGGTCTTCTCATGTACACACTCATCTTATTTCTT
GCTAGTTCTCTTTAATTTTGTATTGAACTTATTCCTCGTTTCTTTTGTAGACTACTTCAGGGACAGAATG
GTTCTTTTTACTCACAAGCCTCGGCTTGGAGATCTTATCAGCTGCTTTTGATCAGGCTTCCTCCCCAAGT
AAACCACACTACGCACTATTCGGTATGCTGTTGGCTTTTGGGGCTCTACTCACTTGCATATGTGAGCTC
CTTCACAAGGGTATAAAAGAAGGAGTCGTGTTGAGGAGGTGGGGAATGCTATGGTGGTTTTATTATCC
ACCTCCTCGTTACACAGTTTTTGGTACCCTCCCTGACATCTATGGCTTAGCTGGTGGCATCTCTCAGTGC
GTTTGCTCGACAATTCAATATATTTACTTCTGTCGCCGTGTCAATAATCCTATCAAAATATCCCTTTGGCC
TACCATCTTTCTTATATGCTTGGCTGCTTCAAGATTGAATGGGAATCGAAATGAGACCCTTGTACATTAT
GATTAG
>PmWRKY52
TTAAATCGCAACTGTAGGTGCTTGAAATGTAATCTTGGGGTGTTGATGGTCGCCTTCATATGTAACAAC
CAACATGGTAGGATCTTCCAAGCACCGTTCGACATGCTTTCTTGCAGGGCATCCCCTCACACTGCTGCA
CTTGTAATAACTCCTTTGAAGGCAAAGCACCATGGACAGACTTATCAGCTAAGCTTGGAAATGGCTAA
GCATATATATAACATTCAAGAAGGGCACATATAAAGCTCATGGCCATCATCTAATATATTGTTGCAAATTC
TATATCCAAGGACAATGTTAGAGTAATAATCTATAAGAAGCATATCAAAGTAAGGGAAGTGATTTTGTTG
AAATAATTAATTCATTTTCCTCTGTTTCTCCAGCCAAGTAAGGACTCATTCATTTCATGGAATTATGGACT
AACTTTCATCTCTGCCATAAGCTATGGATTGTTTGTGCATGATGAATAAGCCAAAATGTATGAAACAGA
AGAGAAAATATACGTATTAGACAATCTAGACCTTAAGATTTGAAATCATTATGGAAAGGCAAAAATAAA
ATAACATAGACATACCTAGGATATGGAGACCCTTTAATAGGTTTCTGCCCATACTTCCTCCAGGTATAGT
CATCAGGAGGTATATCAGCTAGCTTATTGCTTAAAGCAGGAACTCGGATTCTTCTCTTTATTCTAAGTTT
CCTGTTAGGAGATGAACAAATACAAGAAATTAAAAACCGTTAGCAGGACACAAGAAGATAGAAAATC
AGAAATCCAATCAGAAATATTGTGTAAACCAACAAAATGTAATGTCATAAAAAACTGCATTTTAATCAG
GATTTCAATACCTTCGCTTTGAGCAATGGCATCCACCAGCAGAGACTAGGCATCTTGGAGTGGCTTCTT
CACTTTTCACCCCACACTTCCTTTTGGAAGAAAACATAGAGGTACAATCTCGAGAACCCAAGAGTTCA
GACGACGAATAGTGAATCATCCGCGTGTTAGGGCTACTTCCATCCATACTGATCAAAGAAGTGCTAGTA
GGCTTTTGTGAGAATTGGTTCAACCCCATGATAAAATTATTAGGAAGAACCACACCGGCTTCAGATTGC
CCTTGCTGCAATGCCGGGTTTGGTTTTTTCTCTCTAGCGATGCTAAAGCTATTACTGTGGATCACATTTG
CAGTTGCTTGATCACTTTGATGAACAGCACTAAAAAACTGTCTAACCAAGCAATTCCGAGGCTGGGGC

AGGGTATGAGGCTGAGGCCAATTGTGTGTAGTATTTTGGGATGAAGGGTTAGGAGAGTCCAACAGTTC
AACCTGGTTTATGTCATGGGATTTTGGCAAAGGACCTCTTCTGATCCTCTTCTGGTTTGATAACAATGAT
CCATCAAGGAGAGCAAGTAGTTTTCTGAATTCTTCCACTGCATCTTGAGCAATCAAGCTTACTTCTTGA
ACACTTCTCTTTTGTTTATTCTCAGATACACAACCAAACAGGTGATGGGCATGTTTCAAACTACTTTGA
GCAGCCTCATGAATTTTGAAATCCAATCCTGGGTTCAT
>PmWRKY53
ATGGAGAACTCTCAAATACCATATT
GCTTTGCTCCGTTAAGCATTGAAGAAGAAGATGACTTTTTCCATGCTTTGCCTCAGCTGTTTGAAGCCA
CAGCAGCTTTAGATGAATTGGAGGAGCTTTACAAGCCATTCTATCCAGTTTTGCAACCCTCAACCCCAC
CAATCATTGTTGCCTCCTCCATGTCTGTTCCCCAAGAGGCCATCACTGTAGAAGTACCAAAGAAGCTG
AAGGAATCATCAACTATCTGTAAAAAAATTAGGTTTGTCAATTCTCTAACTTATTTTGTTAATAACGGCT
TATAGATAATGGTAGAGTAAAGAAATTTGTTCGTTAGATTAAACGATTTATAATTTTCCCAAATGTTTAAT
ATATGTTTTTTTTTCAAATGTTGANAGGAAGAATCAGAGCAAAAGGGTGGTGAAAGAGGTGAGAGCA
GAAGAGCTTTTTTCAGATGTGTGGGCGTGGCGTAAATATGGGCAAAAACCCATCAAGGGATCGCCATA
TCCGAGGAGCTATTACAGGTGCAGCAGCTCAAAAGGATGCTTAGCAAGAAAACAAGTGGAAAGGAGC
TGTTCCAATCCTGAAACCTTCATCATAACCTACACTGCAGAACACAACCATGTCCATCCGACTCGTCGA
AACTCTCTTGCCGGAAGCACTAGATCCAAGTTCCCATCATCTAAAAACAAAGGCAAGTGTAAAAGTTC
TTTGAATTCTCCAGCCATGACCAATTTGGCGGCTTCCATTGAAGAAGATCCCGATGTGCAAAGTGCAA
GTACTAATGCTGTAAAAGAGGAGGCACAATTGTTGTTGGAAGAGGATGAAATTAGTCATGAAAATGTC
ATGTTGGATGTGATGTTGAGTGATGAAATGATCCCAAGCTTAGAGAATTTGGATAGAGAGTTGGAACC
AGTTATGGATGGTTGGTTTTTGGATCAATTTTCGGATAATTTTCCAAGTCCTTGGTTCAATATTGGTCAC
TCTAATACTGTAACCGGTGCTTGCTGA
>PmWRKY54
ATGGATTCTAGTAAGAGCTGGGAGCACAAGTCACTGCTAACCGAGATCACTGAAGGGATGGAGCTAG
CAAAACAGTTGAGGCTAAGTCTAAGTGCAGCATCTTCATCAGACACCAGACAATTTTTAGTGCAGAGG
ATATTGTCCTCCTATGAGAAAGCCCTTTTGATACTGAACTTCAGTGGGCCGGCTCAAAGTACTGCGGGT
GCAATAGCCAGTGTGCCGGAGTCTCTTGTGTCAGCTAACGCAGGTCCTTGTTTTGATGACTATAACAAG
AGTCCCAAGGATCATCAGGACCTCACAGATGTCTCCAAGAAGAGGTGAGCTTAGACTATTCATTAAGT
CATAAATATAGAGAATATGATGCCAAGAAATCAAATGCTAATTTGATTTTGCATATTGCAGAAAGATCAT
GGCCAAATGGACAGACCATGTAATGAGAGTTAGCTCTGAGAATGGGATTGAAGGACCTCATGAAGATG
GCCATAGCTGGAGAAAATATGGACAGAAAGACATCCTAGGAACCAAACATCCAAGGTAACTAAAGTG
CCTTTTTACTTAATCTTATCCTGTTGTAAAATCCCATAAGCGAATCCATTCAACCATCCAATCATTTGCAG
TAACAGTCACCGATGCTACATTTCAAAAAGCAACATATAATACTCTACCTTTTACGCGCACGCGCACGC
GCACAAAGCTTTGTCAGGTTGACCCACAAATGGGAAATATAGCAGTCTTCTGCATCATAGAAATATCAC
TTTGTTATGAACATTCTGATGACCAATTTTGTTTAAATTTGCAGAAGCTATTACAGATGCACCTACCGAA
ACACGCAAAGCTGTTATGCTACAAAGCAAGTGCAAAGATCAGATGAAGACCCCACGATATTCGAAATC
ACATACAAAGGAAAGCATACATGTTCTCATGGTAGCAATTCAGTTCTACCACCACCATCACCAGAACA
GCAAGAACAAAAACGAAACAAGCAAAATAATACTTCTCACCAACAGCAGTCTCAAGGAAACCAAATG
AGCTTTCCGACTAATCTGAGAGTCGATACCAAGTTCTTGGAAGACAGAGAGAACATGACATCTCCATT
CTCTTTCACTTCAACTTCATTTGGATGTATGATGGCTGATGATGCCTTTCTATCTTCAATGCTTGATGATA
ACAACACTTTCTTTGACAACTTCAATCATTCATTGCTATCTCCAGCCGCAGGCGAATCAAACTACTATTT
GATGCCACCAAGCCAGATGAGAAACATTGCAGGAAACGAGCAACTTTCAGAATGTGGTCTTACTGAG
ATCATCTCAGCCAACAATTCGTCAACCAATTCTCCAATTCCAGACATGGATTTTCCACTGGAGCCAGTG
GAAATTGAGCCCAATTTCCCATTTGACACCACAGGAATTTTCTCATAA

>PmWRKY55
TTAGGCATTATATGTTTGTAAATGTCTCAGTATCTGGTCAAAGTTTTCTGCGGAAGTTTCATCAGTGGGA
TGTGAATGGATTCCTTCATATGTCGTCACAACAATCTCTTCATCTTTCGAAAGGCGTTGGACTTGCTTTT
TGACAACGCACCCTTGATGCGTGCACCTATAGTAGCTTCTGCATCAAATAGTCAAAAAACCAATTTACT
AGAACGAGCAAGATGACGAAGGCATTGCAGAAACAGAAGGAAATTTCAAGCATTAGGCGAAGCATG
GAACTAGACGAATACGAAGTTACAGAGCAGATATGAGCAATCAAGTGTTTTAGTGCATTTTTTCAGAA
GACTATCTTAGGTATAAACTTTGCAAATCTAATAATTAATTGCTGGCAGTTTTCTAACAGTGTTTGATTTT
GAAATAGAAAACTTCCATTTTGATCCCCATTCTTAGCACTTGAACTGAGAACCAATATATCAAAACTTC
ATCACAAGAATCCAAAACTTCTTGGCCTTTATTGGGGTTTTCTCTCTTGGTTCATTGTGTGTTTCACAGA
AGATTCATTGCAAAATAAAATCAGAACTGTAAAAGGTAGCTATAGGTGATAGATTCGACAAACGAGCC
AGAAATTTGACAAAGTTCAATATAGACAGATCAAAGGAGAATCATGATAACTTCCAGGTGACAGATCA
AAGAGCTTTTCTATATCTATTGTTTACTAGTCAATGAAGATCTTCTATCAAATGGGTGGTTGGTGTTCTTT
TTCCAGGGTTTAAGTAGTTGATAAGTGAACCCTTTTGCTTTCTATCAAAGGCATGCATATAAATATAGAT
ATAATGGTGGTTTAGTCTATGTGATTATTGTGATAAGATGTTCAAACATACCCAAATTAGATCAAAGATC
AATACCATAAATTCACAAGAAAACTATTTGATGAGAGCCAGCAGAACTAACCTAGGAAATTTACTGTTT
TTAACTGTTTTTTGCCCGTATTTTCGCCATCGGTACCCATCATCAAGTATATCAACCTGGCTCCTTGTTTG
AAATGCATATTTGTGCTTCTTGGTCTCTTTGCCACCTTCTTTCTTGCCGGATTTCATCTTGTCCACTGAA
CCATAAGAGGTTCCACAATGCTGATAAATTTTTGCTTCATTCTTCATTAGACCTGCGCTGTCAAAGTGA
AAACGAGGAAACACTTGAGAAATATTTGATGGGTTTGATGGAAGATTAGGAGAAGCTGAATCAGATGA
TTTTGGTAAAGCCAGGAAAAGTATTTGGTTGTGCTCCAT
>PmWRKY56
CTATAAACTTTTGTGATCTCTAGTGTCACCAGGAGACGTAGATGCGCTCTTGGGGGCACTTGCAAATGG
CAATGAAGGTGGCAGAAACATCAAGCTCCCGGGCTGCGAATTCGCAGAAGTCGTTTTGTTCAAATAGC
TTGATGCACATCCAATGGTACTATTGTTCCCTATCTGCGTTTGGAGGTATGGAGAAGAAGAAGTAGTTG
TACTTCCTGCAAATTGGTCGCCCCAGTTGAATTTCTGAGCCATATTGTTATTGTCACCACCACCAGCCTG
ATTGTTGTTGTTGCCTAGCATTCCAGTAGTGCCAGCTGTCCCAACATTACTGTTGCTGCCAATGATTGA
AGAAAGTGCAGCTGCTAAGGCAGATTGGAAACTTGGGTCAGCTGTGATTGCCTTGGTTGCAGCAGCA
ATGGGGTCTGGTTGAAACTGATGAGGAGTTGCTCCTTGAGAAGGATGAGGAGGAGGAGTTGGGTTAA
GATTATTCTTGTGCATGTAGTTTTGGTATATATTGTTTTGTAGAGGCTGCTGCTGTTGCCTAATTCCAAGA
CTACTTAGTGATCCAATTTGGTTCTTGTTGTTGCTATTGTATGGTGGTAATTGAGCCCCATAGGAAAGGA
ACCCATTGCTCCATGACATGTTGGTGTTGGATTCAGAATTAGAACCAAAGTTGAGGCTGGTGGGAGGG
TGTAGTTGGTGAGAAGAATTTGATGAAGAAGAAAATTTATTGAAATGGGACAAGGGATTGGAGGATGA
TGATGGTGGTGGTGGGTTTGAAGTTAGGTCTAAGGTGATTGTTGGAAGAGAAGATGACAATGAAGAGT
TGGGTATGAAGAATCTGGATTTTGAGTTGTCAGAGAGATAGAAATTGTATCCATGAAGATCGTGAGCAG
CAGTTGTGGTGGCCCCAACTGATGGGTTCAGGCCAGACCGGGCTGATGATGATGAGCCGGATAATAGC
ATGGAAGCGGCTGCAGAGGTGGTGGAAGCCATAGCTGTGGCTGACATAGGAAGTGGGTGATTGTGCG
TTCCTTCATATGTGGTGATTAAGATGGACATGTCCTCCGCACATCTTTGTACCTGAAATTAATTACGAAC
CAAAAAAATTAACATATGTACAATACATTTTTAAACTACATGGTGCATCATATTATGTACAGGTGATGTGT
TCATCGCCAAATAAGGTGCATATATATTCACCTGTTTCCGTACAGGACATGACGGTGCGATGGTGCAAC
GGTAGTATGCACGAGGGCATGGATTTCCTTTTGCAATCTTCTGTCCATATTTCCTCCACTGACATCCATC
ATTCATCTGTGTCCAAATACATTATATTTTGTTAGTAAAATCTCACGCCTGGCTACATCTAGCTAGCTATA
TCTTTAATTGATTTCATTTTTGCATGTAGACTAACCGTTGGGGTATCACATCTGGCTCTCACTGAAACCC
TAGCTTTCTTCACTGGGTTCTGTTGGGCAACTTCATCTTCTACACTTCTCGCTGTCTTGAGAACTTTCTT
AGGTGGCCAGGTCTCACCTGCTTCTTCCTTGGGCACCTCTTCAAGGCTGCTTGCAGGGCTTGAATTCG

ATAAAGGCACTTCAGTAGTTGCAGCACTTGATTTAGGTGCTTCATATTTGCAGTCTAGCCCCAAAGACA
AGCTTTCTCTATCTGACTCGTTTTCTTTCCCTTGACTACTGGTACTACGGTTTTTGTTCTTTTCATCCTTT
TTGGCTCTGCTAGAAAACGATCCAAGGCTGAGTGAGACCATCTCGGATTCCTCGTATTGATGGTTATAA
ATATTGTTAGTACTAGCAATTGTAACATCCTTGGACTTGTTTGCTTTTTCTTGAATGTCATACAACTGCAT
TTGAAGTGTCTGGTACTCGTCTGTTATCCGATCTAAGTACTTCTTCAGCCTTTGATTCTCTTCTCTAACC
TCACCCATTTCAGCTCTAGCAGATTCAAGCTGATAATCCTGTTCCTTTCGACTTGATGAAGTAGACGCC
ATTGAATGATCAGGTTCCACCTTCATATTACTCGTCGTTGATATCTGTTCAATTCAGCATGATAAGTTAGA
AAAGTTCATATTGATGAGTTAACAGTATATCGTAATATTCGAACTTGGAATCTCTTTTAGCATTGGAAAG
AGAAGTATTACCATTAAACGAATAGTTAGTTAGTCTTCCTATTCAAATTAAACGTTTAGCATAGACAAAA
GCTTAGCTCATAGGCAGCCATGTAACAAGTCATGCAAGTTCAAACCACTAGCTAAATAGCCAAACATC
ACATATAGAGCTCTCTGGTTTCTGCCCTAATTTTAGGTCCTCTAATATTGAAAATATTTATCTGCTCATGA
AGATTACACCTTTCAAATTATGATCTTAACCTTTCATTAGGAATTATATTCATAATTTTACATTGACACGTT
TGAGTGGGAAGACAAACTTGGGAAGAAAAATCAAATCAGTGATTGAGATGGTTTCAAATCTCCATTAG
TAATGATAGATATTTTGAGATATTTATTTGCTTTTATATGCAAAATTACTTGAAATCCAAACGAACTAGAA
AAATATATAGATCAGTCAGGTATCATTCAAAAACTGTTTATTTTAAGAAAGGGGAAATATTTTCTTGATT
GGAAAGGCTTCAGTTTCTATTACCTGCTTGCTGCTACTCAAATCTTTCTGTGTAGGTGAGGATGATTTCA
TTTCGTTATTTTCCTGGCATGATCCTCCATTTCCAACCTGTAAATTTCATATCATTAAAAGAGAAATAATT
GTTAATTAGCTAGCAATTAAGTACTTATAAAATTTCATGAAGAATAAAACGATAGCCCGATTTTTGTACT
TGTTTCAACCTATACATCAATAAAAAGCTATCGCCTTTGACTAAAAAAAGTACTTATAAAATTTCGTGAA
GAATTAATATGTCAATTTATAACTTAGTTTTGTCAAAAGATGAGGATAAAAACAGCTAGCAATAGCCAC
AGAATCATGGATATGCACCTTATTTTCATCAAACTAATAATATTAGTTATCTAAAACAAGTGATCTTTCAA
AATTTATAGAATTTTTTCCTTTGTGGGTACTCAACGCATAAATTAATCACAAGGTTAATGATATCCACATG
TAATTAAGAGTTTCATCATGTATTATTTTTCTGAATATTGATTTCGAACAAAAAATTAGAAACAAATATGA
CTTACTTTGAGAAGAGTATCTTCTTCGCTACCAGCTGCTTCTTGATCATCATGATCTGCAAGACTGCTAG
ATTCAAGTACCAGTCTCTTCTCTTCCTTTTCTGATCCTTCATGAGAATTGGACTTTCCCAAAGCAGCCTC
CAT
>PmWRKY57
ATGGCTGAGCACGAGAGCTTCGATGCTCTCCAAGTAGAAAAGCTAAAGCCAAACGAACAAGAACAAG
AAGAAGATGAAGAAGGCGATGAAGATGAAGACGAGGACGAAGAAGGAAAGCGACTCGGTGAAATTC
ATCTGGGCGAGTTACAGAACTCTGCACCAGAGTCAAGAGAGACGCAATTAGAAACCCTAGCGGTGCC
TTCTACGCTGGAATTATCAGAGAACGATCAGTGTGCAGGTAAGCTTTGTTCTTCGGAAACGGTGTGTTA
GTTTTTGGAAGAAAAAAAATGTTTTTTTGTTTTTTTATTTTATATAGCTTTAGTGCTTGGTGGGATTATAG
TGAACGGGGTGATCTTGTGTGGTTGATCCCACAGGTTTTCAGGTTAACTCCACGTCACAGTCGATTGAT
GGAGCTCAATTACAGGTTAGAAGCTCATTCATATTCAATTTCTAAACTTTTGATCTTTGGCTAAGTGAAT
TGTGAGCACTTTTATTTGCGTGATATTACTGATTCTTTTGAGAATTTCTGTTTGCTTGACTTTTGTTTTTT
GGGCTGGAGTGAGGAGGGACGTATAAATATATCACGAATTGGGAGAGAAGTCATGTGTGCAAGGGTGC
TGGTGGAAATGGTTTTGCATTCTTGCAGTTTTTTGTTTATGAATTTTCTTTGGAATTGAAATCTATGAGT
GCCTTTTCATGGGGACGAGCAAAAATGTTTGTTAAAGTTCTTGTTTGAGGGTTGAAATCATGAATCCAA
AATCCTTGTGATTATAGCCTCGCAGTTTTTGTGAAGCCCTCTAGGTACTAAGTAGGGAGAGAGCCACAA
GTGAAGAGGAGTGTCAGGTTGCAAAATAATTGTTAGCCTGTATCGCTTTTGGAGTAGTGATAACCAAA
GTAAAGTCTTTTCCTCTTTCCTTTTGAACCTATCTGTCAAAAAGGAAAAAATAAAAAATAAAGAGATAT
TATCTCACATACTGTTTGCTTTCTGGAGAGGTGGATTATATGTCAAAATGAAAAGAATGTTAAAGTATCA
TTTTTCAGAGGATTGGCTGGAAGTTAGAAAACTTGACCACTTTGTGATCAGATAATATCAGAAGACAA
AATGATTGGATTTTATGACTGATGGAACAGGCCAATACATTTAACAATAAAAACTTTCAGTCTATATGTG

TGAGGAACAGGCCAACTTAATTGACCTTACATAATATCATTTAGACCTCTTACAGTCTAACTTGAGGCG
TGGGTCTTCCTTACGACAAATTCTAGATGCACATTCGTGCCAATCTGAATGGGTCTTGGATGCATCTTGT
ATCACGTAATTTGAATCATATGCAACTCCAGAATGTTATACCTCTCATTATCATAAGTCAAATATCTGCTT
CCTTGTGTTTCACTTGTATAATTATTTTCCTTGTTCTCTCTCAGGAGCAACTTGGGGTGAGCCACCAGG
AAATTTTGGCAATTGTAACACACCAGGATTCTCAGATGCAGACACAAAGTCCGGTTCAGTTGGCTGTT
TATCCAACTCCATTGTCAGAACTATCACCAACGTCTGTTACACAATCCATTCAGACTCAGACACAAAGT
CCGCTTCAGTTGACTGTTTATCCAACTCCGTTGTCAGAACTATCACCAACTTCTGTTACGCAATCCATTT
CATCTGCTCCCAGCCCGATTCTGCTAGAACAAAAACTGCCACCAGAAAAGGTTAATACTTTATGTACAC
CAGAGGTGGACAAGCAAAATTCTTCTGACCATAAATTTATATCTTCTGTTCCTCTTGTGAAGACTTCTG
CTTCTGATGGTTACAACTGGAGAAAATATGGTCAGAAGCAAGTGAAGAGCCCTCAAGGTTCTCGAAGT
TATTATAGGTGCACATATTCTGAGTGTTATGCCAAGAAGATTGAATGCTGTGACCACTCGGGCTATGTAA
CAGAAATTGTTTACAAAAGCCAACATACTCATGAGCCCCCTCAAAAAAGTAACTGCACAAAGGAAAG
TAAACTTGCATTATCTGCTGAGTGTGTAAGGAATAGTGTTACAGAACATCCCTGCAGAACATTTAATGA
TTCAGAGGTGTCCACATCCTCAAAAGAACGTATTCAAGAAACACCTTCAATTCCTGAAAGAAAGCGG
CAAAGCCCAAGTGACTCTGATGGTAATGGTGATGTTAAGATCAAGGAGGAGCATGGTGATGGTGATGA
ACCAGAACCCAAACGAAGGCAAGTTTACTCGTAGATTTTGTTTCCTTTTCTCATGCTGTATTTTGTAACA
TACTTGTTCAAAGAAGTAGAAATTTACCTGGTTTTGATATACTCTCTGCTTATTGATAACCAGAGTGAAG
AAAAGCAACTTAGAGTATTCAACTTCTCTCCTAAAACCTGGCAAGAAACCTAAATTTGTTGTGCATGC
GGCGGGGGATGTTGGAATATCAGGTGATGGATACAGATGGCGCAAATACGGACAGAAGATGGTGAAA
GGAAATCCGCATCCCAGGTATGCCCATTTCCTTCATCTTTTTTCTTTGTAGCTTGATATATTGACCCAATT
AATCAGCATCTGGGGTTCAGATTCAGTATTTTATGTTGTTAAAAGATGTTAGGCATATGTTCTCTTTACAT
AAACTTCATCTTGCCTTTGTGCTAGACTTATCTGCTTTAAAACTTCATAGTGCGCTTAATTTTTGAAAGA
TGCAATTGGACGAACTGGAAATTTCAATGATGCAGAGCCTCAGTTATGAATTTTCAAAAGAACAACTT
AAATGTTCCAATTTGCTTTTGATTCTTAAGCAATGTTAGCTGTTTCCATTTTCATTTATTTGCGCTTGCAA
GATGCAAGCAGAGCAGAAATGACATTGACTAAAGAATGCCTTTTACTCGTTTTGTATTTTGTCTGGTGG
CGTCTATAATCAAATGAAGCTTTTGATATAATATCTGCCAGTTGTGAGTATCAATTGACAAGAAATAGAA
AGTGAATGGAATTTATGCAGAGTGTTAAGAGAGCGTTATTGTTTATTATTATTAATCATGACATATGAAC
CTCTCCCTTTGGTTGTAATTTGACTCTTTGGTATTATGTATCTGGGGATATTAATATTAAGCTATTTGTAGC
TTTAAATCAGAAAGTGTCTTTTCTCTGCACAGTAATCTTGAGCTGCAGAATTGCCAGCTAAAAGGTTAT
CGACAGTTTCAAATGATGCAAATAATAGAGAATAATTGTTGGGTATGCTATGCATGCAGTTGCCTTTAGA
CTTAAAGGAGTTTGCTTATTGCATAAATCTCCGTTGCTTATCATGTGAGCTGTTGAAACCCCACTTAGGA
ACTACTACATGTATTCCTTTCCTTATGTTTTGATAATTGTATTTAAATTTTTTAGGAACTACTACAGATGCA
CTTCTGCTGGGTGTCCAGTCCGGAAGCACATTGAAACAGCCATAGATAACACAAGTGCTGTGATTATA
ACATACAAGGGGATACATGACCATGACATGCCTGTTCCAAAGAAACGACATGGCCCTCCGAGTGCTCC
TCTTGTTGCTGCCGCTGCTCCTGCTTCCATGAACAATTTGCATATCAAGAAGACTGATACACACCAAAA
CCAGATTTCTTCAACCCAGTGGTCAGTGGACACCGGGGGAGAATTGACTGGTGAGGCCTTGGACCTT
GGAGGTGAGAAGGCAATGGAATCAGCTCGAACTCTTTTGAGCATTGGATTTGAAATCAAGCCTTGCTG
A
>PmWRKY58
TTAGAACTCCGGAAATCCATAACTTCCTTCACTTTGCAACATTTTGAGGGTATAGGGTGATTGCCTTTCT
GTTGTTGGCTGCCTTGTATTCTGAAGAGAGCTGAGGTAGCTCAAGTTCGAATGATTAGGCAAGGCTAA
GGGCCTCACAGCCATGGACACATTGCTGTTGTTGTTGCTGCCATTATCAGAAGCAGGTCTATTTCCATT
GCTATAACTGCCGCTGCCGCGCGCTGCAGGAACATCATGGTTGTGTTTCCCTTCGTATGTGGTGATCAC
AGCCCTTGTATCATGTGAAGCTCGCTCCACATGTTTCCTCACCGGACAACCTACCGATGTGCATTTGTA

GTAGCTCCTACAGATTGGAAAAACAATTGATTAGGTTAATCAGAAAAGTTCAAAAAGTGTTTGCTAGT
AATTAAACTCAAAAACCCAAGAAAGCTAAACAAATTTATAGGATGGTTACCTTGGATTTGGATTTCCCT
TCACTACTTTCTGTCCATATTTCCTCCATCTGTACCCATCATCCAGAATATCTATTTCGCTTGTTGTCTGC
ACTACAATTCTTGGCTCTTTTACAATTCTACTCCCAGCAGCTGAAAAGGGCTGATCGTTCGCATTTTCTC
CCTTCCTGCAAAATATGAAAAAGTACATTCAATCGTCTGCAGTTTTGGCAATAACATATACACAATTCAC
AATCGAAATCACAAGTCTATGCACTTACCATCTTTTGGCCTCAGGCTCATTTTCATCTTCAGCTCCTCCT
GAATTACTTAGTGGTGAGTTTTGTTCAAACTCATCCTCTCCAATTGAGGCTGAAGAGTCCTCCTGCATC
GTGACAGATTCCATTTTCGGATTGGATATTGTTGGCACAGATTGATCAGAAATGCCATATGAAGAACCC
TGAATTGACTGAGAGGTTGATCTTCTTGTAGACTGAGGCTTGGGATGGTTGTGACTTCCCTTGTATACA
ATTTGAGTGATATGTCCATCCAATGATCTCTCAACCTTTTTTTTTGTTGGGCAATTCGGATATGTGCACTT
GTAATAACTCCGCGGATTTTCGCTGCCCTTCACTTGTTTCTGCCCATATTTTCTCCAATTGAACCCATCAT
CTGATTTTTGTTCTCTGACAAACTGAGACGATTGAGTGCAATGTATTGAATCAGGCCTAGGAGCCCCAC
TAGGTCCATTGGTCTGCATATTTGTTTGGTTTGTGCCAATCTCTGGCAAGATGATCTGTAGTGGCTCAAA
TTCAGACTTAACTCCTGTCTTCTCTGTTGAAGAATCAGCTTGCCTTGAGATTCTGTTGAAATCCCATGG
TTTCTGTTCCATTTTCAACGATTCTTCCTGTTCACATAAGAATGATTAAGTCTCAGAACAATTACAAAAA
ATAAGGCTTCCAACTGCACAACTAACTTGTAAAGATAACAGAATGAAGAGAATTAATTTAGTCTTACCA
CTGAAGCCATGATTGAAGAAGCTTGAATGTTAAAGGATGAGGATGATGTTGCAGCAGGCCTTGTTTCT
GGTTGGAAAGAGAATTCAGAGAACATTTTCTGTTCCCTCTCCATTCCTTGCTGGGTTTCTTTAGAATTA
CTCATCCAGTCAAAGATTTGACTAGAGAAGGCTCCACTTGTTGGAGATTGAAGAGTCTGAAGAACAA
ACACAACACAACACAACACAACACAAAAAACCCATCAGTAAACAAAAACGAAAGAACGAAAGAAC
GAAAGAACCCAACAACATAATAAGTTTATAACTTACATTAGAGGAAGATAGGAACATGGGTGAGCTGA
AAAAATCAGTTGGGCTAAAAGCTGGTGTTGATGCTAAGTACGAAGAAGGGGAAACAGGAGGAGGAG
ACATAGGCAGAGAAGGGGGCTGAAGTGACTTGAATTTTGGGATTTCAATGCCATTTCTGTCCATTAAAC
GGTCTGAAGAATAGTCTGAAAGTCCCCAGTTAGTTCTTTCCTGGTCAATATTGGTGTCCATGTTGTTCAT
GTTACTTGTGAGGAGGTGAGTGAAAGAGGAAGTCAT

Supplementary Data 3. The protein sequences of 58 *P.mume WRKY* genes and 12 *Arabidopsis thaliana WRKY* genes

>PmWRKY01
MASSSGSLDTSANSHPTFTFSTHPFMTTSFSDLLASGTDEDPSTNTTVQHVGHGGLADRIAERTGSGVPKF
KSLPPPSLPISPPSVSPSSYFAIPmGLSPAELLDSPVLLSTSNILPSPTTGSFAAQAFWKANSGNNQQIVKHES
KNYSDFSFQTQTRPFTSSSTMFQSSNGTIQTAQKQGWSNSYFQAQEPQKQDDFSSGKSMVKPEYGSVQSF
SSGMATNIQNNSQANGGFQSEYSNYNHQTSQTLSRKSDDGFNWRKYGQKQVKGSENPRSYYKCTYPNCP
TKKKVERSLDGQITEIVYKGNHNHPKPQNTRRSSSNSSHAIQASNPNTNEIPDQSFANHGNSQMDSIGTPE
NSSISMGDDDFEQSSQKSKSGGGDEFDEDEPNAKRWKKDVDNEGISAPGSRTVREPRVVVQTTSDIDILDD
GYRWRKYGQKVVKGNPNPRSYYKCTNPGCPVRKHVERASHDLRAVITTYEGKHNHDVPmARGSGSHAS
VNRALPNNNNINNSNNNVATAMRPVAHQTNNLRQQTSEGQQAPFTLEMLQSPGSFGFAGFDNSMGSYMN
QAQLNENMFSKTKEEPRDDAFFESLLC

>PmWRKY02
MAGIDDNVAIIGDWVPPSTSPRAFFSSMLVDDIGSRSMLEPPSSNKTAEFFLGSQEEDNNGKNLSQGNASG
EELNEVGSFSEYKSNSRGGLVERIAARAGFNAPRLNTESIRSSDLSLNSDIRSPYLTIPPGLSPTILLDSPVFLS
SSLAQPSPTTGKFPFVSNGHSRSSTLMTEGPDKTNFFEDINTSFAFKPIAESGSFFLGPTSKMGSTSFPQQSFA
SIEVSVQSENSSQSIEPTKVQNQNTNNLQLQADFSRTSTEKDNGANSADPRAFDTVGGSTEHSPPLDEQPD
EEGDQRGSGDSMAAAAGGTPSEDVYNWRKYGQKQVKGSEYPRSYYKCTHPNCQVKKKVERSHEGHITE

IIYKGAHNHPKPPPNRRSAAIGSSNPLNDMRPDIPEQGGPQSGADGDLVWASTQKANVGAPDWKHENLEV
TSSASVGPDYCNQSSSMQAQNGTHLESGDVVDASSTFSNDEDEDDRGTHGSVSLAYDGEGDESESKRRKI
EAYATEMSGATRAIREPRVVVQTTSEVDILDDGYRWRKYGQKVVKGNPNPRSYYKCTNAGCTVRKHVER
ASHDLKSVITTYEGKHNHDVPmARNSSHVNSGPSNTMSGQASSAGIQTHPHRPEPSQVHNSMARFERPSS
LGSFSLPGRQQLGPSHGFSFGMNQPGLANLAMAGLGPGQPKLPVMPVHHPYFAQQRQVNEMGFMLPKG
EPKVEPMSESGLNMSNGSSVYQQLMSRLPLGPQM

>PmWRKY03

MASTTSQAMLSQQQGLFENEEGSNAQMGFFSIPPNLNFSSLGSLKAFGSSSIPSSTNLSETLLPSLTPLKHRE
DHNITTSSDFGGSQLLSLQRSTANLWAWGEVSDDHECLSSKRSNGGDDRHHHHMGVSAMKMKKMKAIR
RKVREPRFCFKTMSEVDVLDDGYKWRKYGQKVVKNTQHPRSYYRCTMDNCRVKKRVERLAEDPRMITT
YEGRHVHSPSHDLDQDSQQASSHLNNFFW

>PmWRKY04

MDCLQNPNPSSAGPYHFGESIDPSIDFDEFSDCFMLDYGVDDHQDSSSLSTVSPEKFMADCFTGSSGGATS
RNSNNNMKCRNEGRRNKTEMGHSRVAFRTKSELEVMDDGFKWRKYGKKSVKNSPYPRNYYKCSSGGC
NVKKRVERDREDSSYVITTYDGVHNHESPCVVYYNQMPPPVDPNNIWTLRASSQSSASS

>PmWRKY05

MAVDFMGYRNSSFSAKLEENAVQEAASGLESVEKLIRLLSQAQQNQHQDKYPSMVMDMDCRAVADVAV
SKFKKVISLLGRTRTGHARFRRAPLTLTSGSSSSSQNQAQTQETFVKQAPLESTKVYHATPIQQIPPPLHHHS
TVLESTKDSSTTINFSYSATTSFMSSLTGDSDSKQPLSSSAFQITNMSQVSSAGKPPLSSASLKRKCSSDNLG
SGKCGAGSSGRCHCSKKRKLRLKRVVRVPmISLKMADIPPDDYSWRKYGQKPIKGSPHPRGYYKCSSVR
GCPmRKHVERALDDPmMLVVTYEGEHNHSLSVAETSNLILESS

>PmWRKY06

MEDAMGATWSDWSEEELVRELLDNETPFFVLPEEALQSQMSVSNEDSVVNRFIPTVYSGPTIKDIETALSV
TTGAIQPQELSPmRLSMLERGLSKVEHKYTLKIKSCDNGAMADDGYKWRKYGQKSIKNSPNPRSYYRCT
NPRCSAKKQVERSSDDPDTLIITYEGLHLHFAYPFFPLNNQAQNTSPPMKKPRNKTSQPQAEDREHEAQES
PRSITPEPDPDLQPGPFPDPHEEFVEKEPSAQGLLQDVVPFMIRNPNPSSASSNSSCSSYRSSPTSPSSLSWAT
SYFDVGFNHSFG

>PmWRKY07

MDIKEAERVVIAKPVASRPTCSSFRSFTELLAGAIDASPSNISSETAVPmIRPKTVRFKPTVNHAVSGLVSSQA
EMSGTAHSNSSEKISKSDSRASVVYKPLAKVVSRATVSVLANMVSSKGNFNTSHQSTQSSVEAGILHQNQ
DKCFRSQLSPNLRHNNPSCAETNQTIEPLKIASQNVEEDPKHIPSTANTDRPSYDGYNWRKYGQKQVKGS
EYPRSYYKCTHPNCPVKKKVERSLDGQIAEIVYKGEHNHSKPQPPKKSSSGTQGLGLASDGTGQDTNNRL
WNSQLNERNEGSEGRVEDQNEVGLPmHSYQSKAPLLYDPLASGGINAGGGTPDNSCGLSGECEEGSKGPE
AEDYEPRSKRRKSENQSNEGGISGEGVPDPRVVVQSSVDSDMTGDGFRWRKYGQKVVKGNPYPRSYYR
CTSPKCSVRKHVERVSDDPKAFITTYEGKHNHDMPLRNTNPGASEKDAQAPTTKEKP

>PmWRKY08

MELSWPESVLSNRERVMEELIQGRELASQLCKVLDDHKSTLVSGCGGGGGGDVGSAEGLVNKILGSFTNT
LLILNGKEADGEIVSGDQIQGNSSGIGSADSSSWDANHAIIKSEDFHEEISCKSASTFKDRRGSYKRRKTSHS
WTRDTPmLTDDGHAWRKYGQKVIHNAKHPRNYFRCTHKFDQSCQATKHVQQILDDPPVFRTTYYGNHT
CRDYLKASELILDCTSPRESSSFIRFGDIKQDHPFFSSFTSVKKEVVIKEEKPQPSDDMMASHHHNHSSSGD
YNVSPHPTEFKSSCPLSGLSSTIDPYDHEGDVMSGLIVGPYYFDDEVLQYEF

>PmWRKY09

MEWSWPESVLSNGERVKEELMQGRELAGKLCRVFDNHKSTLVSGFGGGGGGSAEDLVDKILGSFANTLLI
LNGKESDGEIVSGDQIQGISSGGGSADSSSWDANHTAAVIKSEDFYEESCKTTSTFKDRRGSYKRRKTSHC

WTRDTPmLADDGHGWRKYGQKRILNSKHSRNYYRCTHKFEQSCKATKYVQQIQDHPPMFRTTYHGNHT
CRDYLKASELLLDCTSPRESSNFIRFGDTKQDHPFFSFFTSVRKEELVFKEESIIKEEIPPTSDDRVMTSHDNY
SWLCDYFVSPDLTTFESSGPPSGFSSTLDEFDHEDVISRLMMGSFDLDEVLQYI
>PmWRKY10
MEGVSSNRQSEMEELIQGGEVASGQLSRVINLHNNRSLTVNGDHNHDLGSGDDEGFVNKMLGSFSNTLFI
ANGKEMKEFHHHDDQELISDQIIQVNTGGGGGGGGGGGGGAGASSWDVDHLLDHAIKCEASHEESCK
SASTFSTDRRGSYKKRKTCHSWIKDSPmLTEDGHAWRKYGQKLILNAKHPRNYFRCTHKFDQACQATKH
VQQVEDDPPLFRTTYYGNHTCRVYLKASELILDCTSPRESSKFIRFDDTNPSSKQEHPFFSSFKSLKGEECFK
EETMPSDYMTTQHHNQLALSDYLVSPPDLPmFTSPGLMSGFASANYDDFAFDEFLQYELR
>PmWRKY11
MDEVEEANKAAVESCHGVLSHLCQPKGQVHCKNLLAETEEAVFKFKRVVSLLGGGLGHGRVRKLKKFKP
PPLPQNIFLDGPNYRLDISTKPLQLLPPNSLENRRAEKDSKYGSSLQHTHSQKVFLGNPVVDLEMKIKLPLQ
IPKTKPLQQYHFLEQKHDHQQEIQRLQLQPQQLKYQADTMYSGRNRGINLAFDRSTTRTPSMSSARSFVS
CLSMDGGVANTHCDSFQLIRGVPQPPDRISQQQRRCNGAGENGTVKCGSSGKCQYPKKRKVRVKRSFKV
PmISNKIADIPmDEYSWRKYGQKPIKGSPYPRGYYKCSSMRGCPmRKHVERSLEDPmMLIVTYEGEHKHS
PSDQ
>PmWRKY12
MDQEDDAVSENKTKGCGPGANTISIAERRAAKCGFNAERINTARFRTTSPLPSPmAAARSPCLTIPPGISPTA
LLDSPMMLPNSQALPSPTTGTFPLLPPNDDSSVLKSGTHEDGHRGSDLGSSFTFKRQGDPKYLPGYSSFEN
QGSTVDYQSLVLEQQPIDFEFPMEFPEEANAKNYAVDPSTHVKNLNSGILNANCVDLQRSNSSAASEQNSL
PKEPIHGEDVGSHPFLEGEHRGSYPSAGMVRTSEDGYNWRKYGQKQVKGSEYPRSYYKCTHPNCQVKK
KVERSFDGQITEIIYKGAPHNHAMPQPNRRAGASLGSSFSFDETSEMSEGSRASVKVEGGLVWTNIQSGKD
IKTGYDGRAEGLERTSSTSVVTDLSDPLSTTQGKSMSIFESAETPEFSSTLASHDDDDRGTQGSISLGDDAD
DEESESKRRKKESCLMEPSLASRAVREPRVVVQIESEVDILDDGYRWRKYGQKVVKGNPNPRSYYKCTSA
GCSVRKHVERASHNLKFVITTYEGKHNHEVPmARNSNHINSNGGNAHPSTANAQPmLALPRSSNNPKPET
QVQDLAPHFDRKPEFHEEYLRSSFLGNFNNELKFGVPSIYQMKYPPLQNTMPYSSFGLHANHGVTHQAGS
VVPDFPISLPLNLPPSGNLPLSGFDFNNGKSVCPVQPYFSGQQLQEDDMRFLKPKQEQKDDNLYDACLPII
DQANASLSSSSSSSLLYQRIMGGYPS
>PmWRKY13
MGREKEIGLDIDLSLKIDSKQEKQGEDGDQGEGEEEEREEEEDRNDDDDIEQGAKQEEEDEQAPRAQNNN
QKAAEAAAGEVEDDASVVETSLEENNMKTDQELCVLQMEMSRIKEENKVLRKGVQQTMKDYYDLQMK
FTALHQSNQNKDPQTFLSLDGNDPDAVQGPKTIPKRSPLSPTREDMMKESQLGLSLRLQTSPTTNIQSHGR
EEDDDDKEENKKKEDLTSSSLASSLLQNKLQRTELAGISSHVTSQPNRKARVSVRARCESATMNDGCQWR
KYGQKIAKGNPCPRAYYRCTVAPGCPVRKQVQRCLEDMSILITTYEGTHNHPLPVGATAMASTASAAASF
MLLDSSNHHLLDGTPSNYTQASLPNYYNVPDHRMNPSSHSSSNFRTINPHDPSKGIVLDLTNNFYDHNNTP
QNIPMGSSSSHSSVPPGFSNWMMSTSRSASSYQNPNSITSHLLANSTISRSSGVGVEDQRSWRGEENNMSL
AENVTAIASDPKFRVAVAAAITSLINKENHTANHPFGPRDGGENGSSTSNNWALDHQSFSANGKPIRKSSLE
>PmWRKY14
MAVDLMNFPKMEDQKAIQEAASQGLKSMEHLIRFLSHQQQTNQSSRLDCTDITDHTVSKFKKVISLLNRT
GHARFRRGPVQPDQPVQFPSSSSHPSYSQTLSLAPmLNPRPSPmPmPVTTLPIVPPmPIESSYVQSQPHSMTL
DFTRPNVFASNPKSTEIEFAKESFSVSSSSSFMSSAITGDGSVSNGKQGSSIFLAPmPmVSGPKPPLSTAPFKK
RCHEHDHHSDDASCKYSGSGSASGSGKCHCSKRRKNRVKKTIRVPmISSKIADIPPDEYSWRKYGQKPIKG
SPYPRGYYKCSTVRGCPmRKHVERAPDDPmMLIVTYEGEHRHAPENVGLVFEST
>PmWRKY15

MAVELMMGFIDSFAAQMEENAVKEAASAGIQSVEKFISLISQHHHQFQDSCFSSSSNPEAATEYKAVADMA
VTKFRKVISLLDNGRTGHARFRRAPVTPSPPPPPPPLQETETQIPRPSIQEPHNPKTDQPPGFKTEQSSAFKV
YCPTPSVRLPPLPHNPHLKTTPVVLTKSVLCAERRLDAPTTINFSPSPSISAANSFMSSLTTGEAEGSVQHSM
SSGFQFTNMSQSSSGKPPLSSSSLKRKCNSMDDVAALRCGSSSGRCHCSKKRKSKVKRVVRVPmISMKMA
DIPPDDYSWRKYGQKPIKGSPHPRGYYKCSSQRGCLARKHVERALDDPTMLIVTYEGDHNHSHSVTDPTP
mLVLESS

>PmWRKY16

MSNSNNFRAQESPENDFSDQSNFEFSEYLMIGEWLDEDHRTSMALETVQNSGYQANEVDESRGSSSQLGG
SNSRENESGTVRERQEVRERVAFKTKSEVEILDDGFKWRKYGKKMVKNSPNPRNYYKCSVEGCPVKKRV
ERDRDDPRFVITTYEGIHNHQSL

>PmWRKY17

MISYCPVALEILMNMDAHTPTKRKVDDLQQDLLRLRKENEALRFLLQAMTTKCNTLEQLIREKNIERHDQ
FPVAQKTTQFLVRTDSKDNTLIVKDGYQWRKYGQKVTKDNPSSPRAYFKCSLAPRCPVKKKVQRCMVDK
SVLVATYEGEHNHDAINGSPLGQFCCSSSTADHNNNIIFNPINFPSHGISATINNNDVINIASPSNSSRPVPITL
DLTLSGSMSNQENNGSAGSPQNSSSCAPNINCESRIEDYVAYLTKDHNFTQALAAAVASSITRPPmGTENQ

>PmWRKY18

MDSEWVNTSLGLNVIPFHPmSYDQAPVKKQLELEGDYTKVFEGHASVKQEAAASHVLTEELNRISLENK
KLTEMLASLCENYTNLQSHVKELMITKQQSSDQNDLATNFNKKRKPDSEDYSNMIGLTSTETSSISDEEYG
CKRPKENMNLKISRVYVRTEASDTRLIVKDGYQWRKYGQKVTRDNPSPRAYYKCSFAPSCQVKKKVQKS
AENPCVLVATYEGEHNHMHPETRAEVTLIGSISPNQQLSPLSPSMPKRTSPVPTFSCDKNNNLSPREIEGAPP
mFQQFLVQQMASSLTKDPNFASALAAAMSGRFSDHSRMGNW

>PmWRKY19

MDDDKYNRDPmSTTEFTTQSTWPLDPDSAYFFASHDVRDNTLLTEFGWNFHPDGSRPDGFSELDPIGTRD
MSDLAATSSQLVADCLRPmDSSSSSTAAFRSSDPmPmVGSASTNPSGSSTSSEDPPEKSTGSGGKPPPEIPSK
VKKKGQKRIRQPRFAFMTKSEVDHLEDGYRWRKYGQKAVKNSPFPRSYYRCTNSKCTVKKRVERSSEDP
TIVITTYEGQHCHHTVAFPRGGVIAQESGFAGHHLAQLPPVSSHFMYYPmIQPRERVSPVNLTRTTSHQLAS
PRGDDDDDDDQAAGAGSSHCLNPQTTSVPmDEGLLGDIVPPGMRNR

>PmWRKY20

MAENQEQPPGPPPSTSSSPLKSAAPPPPPPQRPTITLPPRSSFEMLFNSGGANSGPGFGLGFSPGPMTLVSSFL
SDGEDCKSFSQLLAGAMSPmARPPGFPQLEDRGSGDGDDNSDFRFKQNRPSPMFSVPSPGLLDSPGLFSPG
QGPFGMTHQQALAQVTAQAAQSSSYFHIPTEYSSSLSTVPmTSLTQLPmFTSDSTAPQEMPSGAADSGVAI
KESSDISHSDQRSQPSSFTVDKPNDDGYNWRKYGQKQVKGSEFPRSYYKCTHSNCPVKKKVERSIDGQIT
EIIYKGEHNHERPQSKRAKDSGNPIGNIQANPDLASQVHGGHLNKSKKGQESSQATHDHLSGTSDSEEVG
DAETRVDEKDEDQPDPKRRNTEVRAEPmSSHRTLTEPRIVVQTTSEVDLLDDGYRWRKYGQKVVKGNPY
PRSYYKCTFPmCNVRKHVERASTDPKAVITTYEGKHNHDVPmSKTGSHYAANNNASNLRAVNAGTEKIN
KMDLRNNDQQPIARLRLKEEQIT

>PmWRKY21

MEMPKRRSKAMENNNTEENSFLGNTPVEDKTKKSDGHDEEGTSMEMEISKSSSSMDITIQGGRGWLAEP
EQTNTLMPSSSAGKDRALIKHPQEDELQSAKAEMGEVKEENERLKLLLSRIVRNYQSLQMHFHGLLQKD
EEAKKSMDPSSSARDQSNEQDEEADELVSLSLGRSSSIDQPRKDEQMKKTSHFSKNGKGDDEEGLNGAGL
ALELGCRFEPmADQSTEVVMKNSSSDNSCGDPKEDDPTEIWPPGKTSKTTRSGDDEVSQQTHLKKARVSI
RARCDAPTMNDGCQWRKYGQKIAKGNPCPRAYYRCTVSPSCPVRKQVQRCADDMSILITTYEGSHNHPL
PMSATAMASTTSAAASMLLSHSSTSQQGHTATAPISASTNLQGLNFSTSTLSQNSRLPQHFYFPNSSISTTNS
HPTITLDLTAPSPSHFGRFPmAVFSSNPRYPSTCLNFSSSPSSSLDHNNHTLQLQAPWNNNNHTAAGYLNYG

NRVLNQVGSALSMGKQSIFQEPNNLYQSYIQNQKPPPPPPPPSPPPLPPPPHQQLMLTETIASATKAITSNPKF
QSALAAALTSFVGTNNGGSATTGVRENHHHHQHQSSTSTESASGLKLKWAESLTTNPIYPPSPNGIGCASS
YLNKSS
>PmWRKY22
MGNPQGAYSTTDHLQFQNNKSPPmANGFLGLMSDMEVSNNINSSQSKSFGGPEGAVRLGTKKGEKKIRK
PRYAFQTRSQVDILDDGYRWRKYGQKAVKNNKFPRSYYRCTHQGCNVKKQVQRLTKDEGIVVTTYEGM
HSHPIEKSTDNFEHILSQMQIYTSI
>PmWRKY23
MDGRETGELKTTIDQISMANSTVFSDEIIPGSSFASFASSGGNIFDMSDNNQRGSGFMDLLGFQDFNMPPSL
FDFSSSQTTSSSSSMMMVMMPPHHQQQQQPPLASPmSTPTAVALPERESTTSEVLNGSTAPTTPNSSSISSSS
NEAAANKNEDQTTTKAQDEEADEQNQDPEKTQKQLKPKKKNQKRQREPRFAFMTKSEVDNLDDGYRW
RKYGQKAVKNSPYPRSYYRCTTAACGVKKRVERSSDDPSTVVTTYEGQHTHPSPITPRGTMGIAPLPDQPC
AFPSSPFGVQQLLPHHHYQQQQQQQQQPQYSYIYSSAPSLNISSPmYGGGAFNPSSFSTGLLQERYNFGSPS
SSSSAASNLLRDHGLLQDIVPQIRKEAKEEDHLHQ
>PmWRKY24
MDKGWGLTLDSDSFGFFLNKPPmAVKLDHHQNNSKISNFFGGERMFPGIEFPVKLGGREDQLAAPQPSIH
DNNNRVVVDEVDFFSDRKNKHNTTTTDDHHQDMKSKGTISVKKENWTGLDVNTGLHLVTANTGSDQS
MVDDGISSDVDNKRAKNHELAQLQVELQRMNSENLRLKEMLGQVTNNYSALQMHVAAVMQQQQQQQ
NHTAAADQSSQLKHDQNVEAKADQEKKQGLVPRQFLDLGPRATAETDDQVSNSSSEARTRSASPQNINEA
ASSKDHHLKKNDHPIGPLDPENSNNFRDGKRVGREESPESESQGWVPNKAPKLNNSAANKPIDQSTEATM
RKARVSVRARSEAPMITDGCQWRKYGQKMAKGNPCPRAYYRCTMAVGCPVRKQVQRCAEDRTILITTY
EGNHNHPLPPmAMAMASTTTAAASMLLSGSMSSADGIMNPNNLLARAILPCSSSSQFQVPFPGQQPQLPQ
VFGQALYNQSKFSGLQLSQDLMGSNSQQQQQHLPHQTQSASFADTVSAATAAITADPTFTAALAAAITSIIG
GGHPNNNNNNNNNSTSTTSNNSNGGNNNSNSKMSGFPGH
>PmWRKY25
MDSTFFNSSNSNSNSRSKQFMSDQEENDNTTSSTPENSSGSPPPPSTNFSDFSKITSTSSPKKSARRAIQKRV
VSIPIKGDNSNTPPPSDSWAWRKYGQKPIKGSPYPRCSSSKGCPmRKQVERSRVDPSMLVITYSSEHNHPW
PmSRNHHNHHQSNSSSSAAAAATTTKPGSNKTEAPEAQQPEHQDHDPTFADLNDESLLTHDEFGWFADM
ETTSSTVLESPIFAESGCAGGADSADMAAMVFPMGEEDESLFADLGELPECSLVFRHRGVGPQVQIC
>PmWRKY26
MSDHEHKDLYYHDLFQYEDHEHLNGGMISINHNQNLQGIEVPSYMNNTSFAECLKGPMDYNTLATAFGL
ASSTSDEVIFSSANEGDQKPmEHIRYSGGGGDGDGGGGETPLTPNSSVSSSSAEAGAEEDSGKSKKDRQPK
GSSEDGGDSSKKVMSKAKKKGEKKQREPRFAFMTKSEVDHLEDGYRWRKYGQKAVKNSPYPRSYYRCT
TQKCGVKKRVERSFEDPSTVITTYEGQHNHPLPmTLRGNAAASSAFFPHAAAAFNYAPITGSAGPSSNFPQ
ELLFQMPHHYMINNNNQGSSRSVVVNPHHPHHQQQHQLPRADQHGLLQDLLFPSMFLKQEP
>PmWRKY27
MENYQGDLTDILRASSAATSAAGAYHSDHLLHHHHQEPEAAADSWHFPSDHPPPMKFSSASSVMEEEEEE
DSKDNFGDPFSSYMRDPLLHELDISSSSSFFSSPNSSSDMNINITSVDEAGGTSSTFGGAHIVLAPPPPPPHH
HHLHHHQQGGVVDGDPIKRPCNIFSRMLQISPSAKVPVTACDSPLLGAATAVSSPRGISKAVSGSISGAMVG
SDMIINGNSSSGKGCLLENAGVQISSPRNPGIKRRKSQAKKVVCIPmPmAANSRPTGEVVPSDLWAWRKY
GQKPIKGSPYPRGYYRCSSSKGCSARKQVERSRTDPNMLVITYTSEHNHAWPTQRNALAGSTRSQPSKNG
AGSKTSSNSSHQPHQKPmSPTKEEQNETMNDNNHVSPVITAGSASTVKEEFEDIEKQLKLDHHQFNGDHQ
AGFPYRPSMPDDQSNQSHHNEDFFADLGEIEADPLNLLFSQCFTADEQQKGSKAAALDPFNLFDWSGDQN
TNNNNNNNNATSFGEAAKRRL

>PmWRKY28
MESAASDHSIHNCRSIEQPLLSPEFTAVDHDHHGKRVVDELDFFADNKGRLMEMRDQTVEVKEEGAHDH
HGVGQEKQLPDVNTGLNLLTTYTSSDKSSMDDGTSSSHNMEDKHRTNELAVLQAELGRMNVENQRLRV
MISQVNNNYQALQVQIVTLMQRQQNQKADHQTPEQHKMINNGSVVVEEKQMMNGFNHIVPRQFMDMG
RAEKDERSQCPLEGCRSQDCSGSPPRNDIVESMECCKSTSHVLHRDLSGRISTTNGGEDSPDQEFQGWVPK
KVSKMMSPRDVDQASSETMSMIKKARVSVRARSEASMISDGCQWRKYGQKMAKGNPCPRAYYRCTMG
TGCAVRKQVQRCAEDRTILVTTYEGHHNHPLPPmAMAMASTTSAAASMLLSGSMPSADGLISSNSFLARS
ALPNCPPSLATLSASAPFPTVTLDLTRTPTSSEMPLGQPNQLPPSFPQNMMSVPQILGQALSSQSKFSVLDSF
QGLDSATHSLADKVNAATAAITADPNFTAALVAAITSIVGNVHSNNNTNNNITTRNNSDSNT
>PmWRKY29
MESAASDHSIHNCRSIEQPLLSPETAVDHDHHGKRIVDELDFFADNKGRLMEMRDQTVEVQEEGAHDHH
GVGQEKQLPDTGLNLLTTYTSSDKSSMDDGTSSSHNMEDKHRTNELAVLQAELGRMNVENQRLRVMISQ
VNNNYQALQVQIVTLMQRQQNQKVDHQTPEQHKMNNNGSVVVEEKQMMNGFNHIVPRQFMDMGRAE
KDELSQCSLEGCRSQDCSGSSPRNDIVESMECCKSTSHVLHRDLSGRISTTNGGEDSPDQEFQGWVPKKVS
KMMSPRHVDQASSETMSMIKKARVSVRARSEASMISDGCQWRKYGQKMAKGNPCPRAYYRCTMGTGC
PVRKQVQRCAEDRTILVTTYEGHHNHPLPPmAMAMASTTSAAASMLLSGSMPSADGLISSNSFLARSALQ
NCPPSLATLSASAPFPTVTLDLTRTPTSSEMPLGQPNQLPPSFPQNMMPVPQILGQALSSQSTFSVLESFPGL
DSATHSLADKVNAATAAITADPNFTAALVAAITSIVGNVHSNNNTNGNITTRNNSDSNT
>PmWRKY30
MDATTLDHPSGPSDDFDPGLTDFNPGSDPTLFSSTGGGGGAKYKLMSPmKLPISRSPCLTIPPGLSPTSFLES
PVLLSNMKAEPSPTTGSFLKPQMVYGSLSSTTYSANTMCSDFDTFDERNSGSFEFKPHAGSNMVTTDYNH
QRNDQLVQGQAQPQSLVSPPLVKSEMAVSSNELSLSAPVHMVTSGASAPmEGDSDDLSQRGHPNPGVQTS
QFDHKGSGPSVISSDDGYNWRKYGQKHVKGSEFPRSYYKCTHPNCEVKKLFERSHDGQITEIIYKGTHDH
PKPQPSRRYNTGAMMPIQEERSEKASSLIGRDDKPSSIYGQMSSTNEPNSTPERSPVTGNDDSVEGTGSLSN
RMAEEIDDDDPFSKRRRMDVGGVDVTPVVKPIREPRVVVQTLSEVDILDDGYRWRKYGQKVVRGNPNPR
SYYKCTNAGCPVRKHVERASHDPKAVITTYEGKHNHDVPTARNSSHDTSGPTTVNVPSRIRSEESDTISLD
LGVGINSAAENKSNEHLQLHSELMERQSHTSSNFKAIQTTPVSTYYGVLNSGMNQYGSRENPSESRSIEIPP
LNHSSYPYPQNMGRKDLQLCKDKILHQYELTRIHTNTLPLSHSVNQTEMHALWVSLKDNVRCNGNKVAD
VVGRASEICNRRKSSSGKANSGGEIVHGKETPmRELMHRGSYTQAQLYELDIGDPSRNVIEMIVRRATVNP
SEPSNRIKRVLRVQNSIETLERFEKYREMVKKMAKERYMRHPRSTVDGNELLRFYGTTVACCSGESKRVS
ELCKAPNCQVCMIIQSDFYTEYSVSNEIQLSTSSEKFGENSITITRRNKIRRAVIVCRTIAGSMVNMTDKEYE
ESDSIESQKLLSTSEYLVVRNPSAVLPCFVIAFT
>PmWRKY31
MEKQGRRHHIIPKLFPHSVSDCSFNASDDGLVDATNNTSSSNPSIPIPIPIPIKQLFRQPKEIKVMDFFSDNNI
MNDDADDDEEPRRQPQQDCNNNPPmVNTGLNLLTLNSGISSTSASDIHQNSNNKLMTSLQVELERLHEEN
GELKTMLDQMTKSYSQLQAQLLMAMQKQAQNRLREPIKCEANGMLARQFMDPRPSTAAAMDHVRDPS
VAYSSGKTPmDHEAFSSFAPSNLNIEVMSMERDQYQRRLQTNINCAEEALDRSSQCPGSPNYTSKSSDPNL
FDDDNDEPKRSTDQEQVPVADQIPFRKARVSVRARSEAPMISDGCQWRKYGQKMAKGNPCPRAYYRCT
MAIGCPVRKQVQRLAEDKTILVTTYEGNHSHPLPPmATAMAKTTSAAAAMLLSGSTTSKEAHQYHHHHL
ANSGFFSNSQLPFFTSSMATLSASAPFPTITLDLTQSPMQQFHRIPPPSSSTFPLPLHGYHQLMGGLGHPIQAP
MYFPPNYKAPPPPmGVPLGGQRSTSTHDSGMIETVGAAIASDPNFTAALAAAISTIMGAPRPHQGHQGGIN
INDGDIANNNAARGVVASTNHSPPSANIGVPPGSLQPGSPQLPQSCTTFSTN
>PmWRKY32
MDVKKDVVKMEDNSTNIGCGFSSSPFSGIFDFCEGEKSSSLGFMELLGAGQDFCTNSLFDYLPQTPSMLPS

LAPNFPNTSIMAKECSDYSLNQQPmTPNSSSISSASSEALNEEQTDNKGAADQDEEEERDQPKTKKELKAK
KASQKRQREPRFAFMTKSEVDHLEDGYRWRKYGQKAVKNSPFPRSYYRCTSTACNVKKRVERSFNDPSIV
VTTYEGQHTHPSPLIPRPTLTASASAQPNISTTFAMPSMPRTLLSHHYQQQLQPFNFCNYVNGGSPTANASG
TGFHHERRFCTPmTGSAMLTDHGLLQDIVPSHMLKQE
>PmWRKY33
MDYSSAAYDDTSLDLNTKPLRLFDDTPIKKEAQSKILIGFGRQLSPDEESGALLEELQRVSAENKKLTEMLT
VMGESYNGLRNQLLDYMSKNPEKELSPISKKRKSESSNNNNTNSNNNINGAVNGNSESSSSDGESCKKPR
EENIKAKISRAYVRTEASDTTSLVVKDGYQWRKYGQKVTRDNPCPRAYFKCSFAPSCPVKKKVQRSVEDQ
SILVATYEGEHNHSHPSQIEATSGSNRCMTLGSVPCSTSLASSGPTITLDLTKSKSSADTKSTKTKTETPEVR
KFLVEQMASSLTKDPDFTKALAAAISGRILQHNSY
>PmWRKY34
MDPQFYRISPFGTDPmDPDQPMTSENGPGSPSSGEETKVATAPSPKKRRGVQKRVVTVPIGDVEGSKSKGE
GHPPSDSWAWRKYGQKPIKGSPYPRGYYRCSSSKGCPmRKQVERSRVDPTMLLITYACEHNHPKPTTKPH
QTSTTTSPNAEPELRAKTVTPNEEELTIFASQVDLDLSDDSATLLSAFGWFSDVASTAVLESPICAGNSTCAD
YDVATRLGDEEDEYLFADLGELPEGSVIFRHKMVESDEQNRRCSLSVVPCCNSR
>PmWRKY35
MSNEKKNPYQYDPFDYNPHEINRSSFPFFNYGTPSIQDPQNLHGFESDHPNSSFMSFTDCLHGSMDYNTLS
RAFDMSCSSSEVISPQLDHENSKNQQAAAAAGVGDHSVGTSTTENPSTPNSSVSSSSNEGAGSHEHEDSEK
KKKEKQPKVAVCDEAAGDEEDKSKKGSKAKKKEKRQREPRFAFLTKSEVDHLEDGYRWRKYGQKAVKN
SPYPRSYYRCTTQKCVVKKRVERSFQDPSIVITTYEGQHNHQCPmTLRGNLNLNAVGMLSPNSLLTSASLN
GSARFQHEFLTQFLPMNNQLQLQLQSHHHQDDHQASNSMIYSNLVAPRPHHHQQQHQRQHQRQQLHVP
DYGLLQDLVPSFGHKQEP
>PmWRKY36
MISLGEPGTDKIASDIVPKKESSNSEIHAPHQTPDNGICSLQSDHRGNVQSLIPEKSLQLPDDVGTASQSNQE
GSVTSLTSEKAPQTPETSALVLRSGQEGSTPSTARERGLEDGYHWRKYGQKLVKGNAYVRSYYRCTHAKC
PVKRQVERTHNGQITDTVYFGEHQHPKAQVNVPVAVSFLVSIVEERPEELLLTGVEGKSSDVHGHTSNQIE
PVDPmQLSTVADNEGVQRVLSQSNRTRDGDPDSKRQKKEKHNGNSIPVDKPmGEPRVVVQTMSEADIVN
DGYRWRKYGQKLVKGNPNPRSYYRCSNPGCPVKKHVERASHDSKVVIATYEGQHDHDMPPTRTVTHNA
AASNVITTARNGESGTTSEGNAVCHDTSPEHEDKPNKQLNVEPRTKSSDVAGCDMVVDSDLGPERKLNEQ
VVGKACTTEESDAPDIIVPRANELQNGESGIKSEGNNACIDTVIHGNLCPESNSPEQKNPKAEPV
>PmWRKY37
MAKGSGLSIDSDPFGFSLHNPIVLNSFQQDQYNHHQPCRTKKQQQQLHHSSLNMDMDASTIHPRSPPPPPL
PPPTLQFSVNLNCTHEDVDNQHHHHHHQHSPEPPSNEKRKVIDERDFFADNKSHVDQDKSASADPmDKM
DLHGPTDMEFNVNTGLNLLLTNTSSDQSVVDDGISSNIEDKRAKSELAVLQAELERMNAENQRLRGMLN
QVTTNYNALQVHLLTLMQSQKAEQNSSAAEGHGVFDGNNKMVVEEKKLINGNGSPVVVPRQFMDLGL
AANNADADEPSQSSSEERSRERSGSLGENVKVAGHSDDQEKKEFGRGIGREESPDQPSQSWAPNKVPRLN
SPKEVDQTEATMRKARVSVRARSEAPMITDGCQWRKYGQKMAKGNPCPRAYYRCTMAAGCPVRKQVQ
RCAEDRTILITTYEGNHNHPLPPmAMAMASTTSSAARMLLSGSMPSADGLMDSNFLTRTILPCSSSMATISA
SAPFPTVTLDLTQSPNPLQLQRPPGQFNIPFPNPSQNFTNGPVSLLPQIFGQALYNQSKFSGLQMSQDMEGA
QLGHQQQPGHQGQQQNSLADTVTAATAAIAADPNFTAALAAAITSIIGNAHPNNNSNNGTNPmTNSNNNN
GNGNGNATSNNNKLSNSSFPmN
>PmWRKY38
MPNSNSRTAPEKKVDESVGYQEIYSQEESELENARAEMGEVREENARLKLTLQHMEKDYQSLQCRFFDIL
RQEASKKATNVDVGVHRIEEPNQLLSLCLGRSPREPKHDETNTANFKLVQVDHEDLNANLTLGLGNSKLM

ELPMELVRSQKPQETSLEEPKDHSEAGAGESLPPSSKTPKTTRNEDDEVPQQANAKRARVSVRVRCDTPT
MNDGCQWRKYGQKIAKGNPCPRAYYRCTVAPmCPVRKQVQRCYEDMSILITTYEGTHNHPLPFTASSMA
STTAAAASMLLSGSSTSQPGFGSTATLLNGSDFGVFDSSRTNQLYLPKSNPLLPTITLDLTASPSSSPIHLNRL
SSSFASACTFPSGLSFCSSESNISPNSRGNGYLKYGSLPFDKGSFNLDQPYAEKNHQSSSQVSLTESLTKAITS
DPNFKSVIAVALSSMVGGGAATHGNQSERLGHHLKWSEAAHQFTSHNPLIQNGKGCSTPSIFNRLSSSDSQ
K

>PmWRKY39

MAKNQDSERVSVSAPPQPQRPVITLPPRPSAEALFSGGSGASPGPMTLVSSFFPDTYPDSEYRSFSQLLAGA
MGSPMGSTRPIQFNENPVDGSAQLEGGSENGGENKSGFKQSRPMNLMVARSPLFTVPPGLSPSGLLNSPGF
FSPPSPFGISHQQALAQVTAQAALAQSRMHMQAEYQPSSVGAPTEPQAYHPSVMPNEASQQQTLPSTSDH
RSSARQSSEASHSDRKYQPSSVATDRPmDDSYNWRKYGQKQVKGSEYPRSYYKCTHLNCPVKKKVERSP
NGEITEIIYKGQHNHEAPQPKRGKDGGDLNGHLHSQPRPENGLQRLVGDSNGSSENIASHSMLERHQEST
QAAPGQLPGASDSEELRDGEIREEGDADEPNPKRRNIDVGASEVALSHKTVTEPKIIVQTRSEVDLLDDGY
RWRKYGQKVVKGNPHPRSYYKCTYAGCNVRKHVERASTDPKAVITTYEGKHNHDVPmARNSSHNTANN
NASQLKPLAVVREKHPLLKGREFGNNDQRPVLLQLKEEQIFV

>PmWRKY40

MEGDQRGVPSYEVQISFSSTPNPQQSIHEMGFVQFEDHHPHHNQVLSFMAPSSHIQQQQPNNSHHPHHHQ
PmELSSGASAATTTTSNGVTVGFSTHTDLLVARPSWNNSNDQVGTLDPKAISDENGTGNASDCSNSWWRS
SSSEKSKMKVRRKLREPRFCFQTRSDVDVLDDGYKWRKYGQKVVKNSLHPRSYYRCTHSNCRVKKRVE
RLSEDCRMVITTYEGRHNHTPCDDSNSSEHECFSSF

>PmWRKY41

MEGHQEPNYPPPLTPSSLISLPFLLPPQNPLFTPSSASTAPSSSSQLISPPLLEPQQHQVLPDIDWVSLLSGSPm
AGFDGQEINNYKPLVENNNVGASAENINEADREGKGANSKRKGGGRMRKPmSRPRFAFQTRSADDILDD
GYRWRKYGQKAVKNNLYPRSYYRCTHHTCNVKKQVQRLSKDTSIVVTTYEGTHNHPCEKLMETLTPLLK
QMQFLSRF

>PmWRKY42

MPLVVEPLDQRLDFDSLMAALLGTPYSLEGDRNTNSLWTGDTWRYNLRAPLPGTHVPVTSVMAWEGAP
LMKKVGTGRVQKKQRHQNPKRGEQMLLLLLLLFDIEMEFYSPNAQPGLQQQFQVLFSLPDPIKPRNAM
EDLHELYKPFFPKSQPPLSSQITPPPNLSPLTSLTPLTAPKDQTHPIQQHQHQNHYQQQQQQQQQQQSKPS
SHSVSSTTTPRSKKRKNQLKKVCQVPmESLSADIWAWRKYGQKPIKGSPYPRGYYRCSSSKGCMARKQV
ERNRSDPNMFIVTYTAEHNHPmPTHRNSLAGSTRQKPFSPQTVTGSDSTKPTSPmTSASVDEDPVVPQSTT
MESFKEEKGSPMVDDDDDELFGMCDSVVSDDFFVGLEGLAGDYFSDHSPTSFGVPWVSSNAATAAGSI

>PmWRKY43

MEKRKSMEWEQETLTSELTQGKELAKQLMNCLHPSASQEKRDFLISKILFSYEKALSLLKRDVGSDGEYHI
PNTMLESPTSFGNGSPMSEISDQDCKNKNVFKKRKTMPRWTEEVKVYSGTGLDGSLDDGYSWRKYGQK
DILGATYPRGYYRCTHRGTQGCLATKQVQKADADPSTMVVTYRGEHTCSQVLQLARSSVLSLAKQASTG
NQNATREVEKPKASEEMSFSFGAGLRVKTEDLDTREDDIFPSFSFPSTPIEPENVGDHIFCATLMENDLMDG
YSPTFASPmATFEPDYLQATSSFGLGLDYVQTSESGLSEIISAPTSVTNSPIGDFGFSLDDLDFHHFENPESFA
YES

>PmWRKY44

MEGHQEQYYPPPSTSSSLPLWPPHPSYLFPSSSTSMNPPPLPEPHHQQQAQLPDIDWISLLSGHHDHQINTN
EPmAMVEVNNKENNVIEAAQEEKGSNKRKGGEGRKISVMKKASRPRFAFQTRSADDILDDGYRWRKYG
QKAVKNSLYPRSYYRCTHHTCSVKKQVQRLSKDTSIVVTTYEGVHNHPCEKLMETLTPLLKQMQFLSRF

>PmWRKY45

MDETTISLILQACELARDLESNLHNLANQPNLLSNSLDEITKKFVTARERVYGQDPSTSSSLHNMLTLVHQ
QQIGTSHVQEWLRSSYATQLVADQKEAKIGGCIDDDDAEVKGLQAMDIVSASDTNIASSSSQRARRRKDQ
GLISKITVPAPRIGNTEIPPEDGFTWRKYGQKEIMGSRFPRSYYRCTHQKLYNCPmKKQVQRLNNDPLTFEV
MYRGEHTCHMSATAPSIPPPSAEHHNATQESMAQTLATTTTADPPTASLWLSMDFNPIRGGGGSSSRIIGGD
HGGGDGVGTSTTTRYGKEVDFPVVDLADAMFNSGSSSSNSMDFIFHSAENKWESEDKKN
>PmWRKY46
MGSNHKRLIKELVEGKKTAAELQMLLHKPFGDHGSASAEELVVKIMTSFTESLSVLAAEKKNPGDGHED
HQSGAFGEVYQIKPEPSHSHCDDRSSGDSGESRKVQGSKDRRGCYKRRKTSQSWTTISSAIEDGRAWRKY
GQKEILNAPYPRAYFRCTRKYDQGCRATKQVQQVQDNPRLYQTTYIGQHTCKSMVPPQMIIGSSDHWESQ
TVSSESETPNKQNHDFLGSSAIPIVKQEEYKEGTPTPSDLTDNLSSLETNHLWSDFKDDFALCDPmAMCVS
TKMGSDNEDVVSNMYLDMDFVVKSIDFDRDFNFDEVEFPKNSL
>PmWRKY47
MENCNTHGEKMSLINELAQGRELARQLQIHLNVPSSSHGTRESLVQKIIVSYEKALSMLNSSSPmSGGEQQ
LPTGHAAIRMIESPPPSLNGSPRSEDSDREFKDQDNKDSSRKRKTLPRWTQQIRITSGIGLEGPLDDGFSWR
KYGQKDILGAKYPRGYYRCTHRNAQCCSATKQVQRSDEDPTVFEITYRGRHTCTQASTSITSAPPPPPPPP
QNSMDIVDPQQNEQQPQDLLLAIPQGLTVVTEGLDAGDFSPPFMGPmTSGTQYFSASEQGFGGGSQGFQG
AECEIAEILSSSTSAANSPmALGFPFGQADQNLCPNFSFDGPGFFSS
>PmWRKY48
MPINSPRPRKRKCQQKRQVCQVSAENLSADLWAWRKYGQKPIKGSPHPRNYYRCSSSKGCSARKQVERS
TADPNIFVVTYTGDHNHPRPTHRNSLAGSTRNKLSATAGQNQPINNDPGSPPmQADQDAVANNVNSDNSL
DKENEELDEEEERDENEIEDEDVEEDDVLIPNTAMPDEIFLGLKQLGCTSSSGSGGSLAAGASGDTFSDQEP
SSLGSSWAAGATVGGGC
>PmWRKY49
MKPSLQNQTQKFQKNSGQKVRHPKEGRWFMRRLWRKYGQKPIKGSPYPRGYYRCSTSKGCSAKKQVER
SKTDASVLIITYTCSHNHPGPDVSTTNLTQLQPSTEDHNLPmLLPKQEEQEKEEQEKEPEKEKEEKQGKLE
DHPmMKSDHEDHFHYIQSPIRSSQNIMIEQEEDPFLEKTHVSSTLGFLLDEEPLSYSQLMSFSTPKSEENDFF
DELEELPTFSSFPSFMRSNLSLERIPSVPS
>PmWRKY50
MEDVEEANKAAVESCHRVLSLFSQTQDQVQYRNLMVETGKAVSKFKKVVSLLNTGLGHARVRKRKKLQ
IPFPERILLDNPNCITDYPSKTPHFIQSSFPENPVQDLGLNVKNSLCLGNPSLELSTNGKNPLQPmQQAPPTQ
YHFLQQQQQQQQVQQQQQQQQRLFLQQQQQMKHQAEMMYRRSNSGINLNFDSSSCTPTMSSTRSFISSL
SIDGSVANFDGNSFHLIGAPLSSDQNSQHKRKCSARGDDGSVKCGSSGRCHCSKKRKHRVKRSIKVPmISN
KLADIPPDDYSWRKYGQKPIKGSPHPRGYYKCSSMRGCPmRKHVERCLEEPSMLIVTYEGEHNHPRIPS
QSTTT
>PmWRKY51
MDASSGWIKLNFDGLVKNGVATTGFVIRDDNAHTLLAGAKTIGDNSIAVAECLALRDGLAHAVHHDWHNI
IIEGDSKLVIDAINKKCSAPGVLCSFSKMCHTWPPFQAMESHEHLNNNFGAVNSSGTIMENWKRLCAMLQ
NSNEPPVVVGDLQRIMQEESIESKQGAPRLKKNFCQVYSSTKMTGAAESKLVPISEDGYSWRKYGQKGEK
GNEGSTSYYMCTYPYCKRKKKVGRSLDGQITQVAYKGTHNHEERKDMNQSLSRSVWELRGGAPLLSITP
DVLPSIGSSVDNVDHEEPILHEQKHDHDQPSHENTNYGKPRQATKTTSGTEWFFLLTSLGLEILSAAFDQA
SSPSKPHYALFGMLLAFGALLTCICELLHKGIKEGVVLRRWGMLWWFYYPPPRYTVFGTLPDIYGLAGGIS
QCVCSTIQYIYFCRRVNNPIKISLWPTIFLICLAASRLNGNRNETLVHYD
>PmWRKY52
MNPGLDFKIHEAAQSSLKHAHHLFGCVSENKQKRSVQEVSLIAQDAVEEFRKLLALLDGSLLSNQKRIRR

GPLPKSHDINQVELLDSPNPSSQNTTHNWPQPHTLPQPRNCLVRQFFSAVHQSDQATANVIHSNSFSIAREK
KPNPmLQQGQSEAGVVLPNNFIMGLNQFSQKPTSTSLISMDGSSPNTRMIHYSSSELLGSRDCTSMFSSKR
KCGVKSEEATPRCLVSAGGCHCSKRRKLRIKRRIRVPALSNKLADIPPDDYTWRKYGQKPIKGSPYPRSYY
KCSSVRGCPmRKHVERCLEDPTMLVVTYEGDHQHPKITFQAPTVAI
>PmWRKY53
MENSQIPYCFAPLSIEEEDDFFHALPQLFEATAALDELEELYKPFYPVLQPSTPPIIVASSMSVPQEAITVEVP
KKLKESSTICKKIRKNQSKRVVKEVRAEELFSDVWAWRKYGQKPIKGSPYPRSYYRCSSSKGCLARKQVE
RSCSNPETFIITYTAEHNHVHPTRRNSLAGSTRSKFPSSKNKGKCKSSLNSPmMTNLAASIEEDPDVQSAST
NAVKEEAQLLLEEDEISHENVMLDVMLSDEMIPSLENLDRELEPVMDGWFLDQFSDNFPSPWFNIGHSNT
VTGAC
>PmWRKY54
MDSSKSWEHKSLLTEITEGMELAKQLRLSLSAASSSDTRQFLVQRILSSYEKALLILNFSGPmQSTAGAIAS
VPESLVSANAGPCFDDYNKSPKDHQDLTDVSKKRKIMAKWTDHVMRVSSENGIEGPHEDGHSWRKYGQ
KDILGTKHPRSYYRCTYRNTQSCYATKQVQRSDEDPTIFEITYKGKHTCSHGSNSVLPPPSPEQQEQKRNK
QNNTSHQQQSQGNQMSFPTNLRVDTKFLEDRENMTSPFSFTSTSFGCMMADDAFLSSMLDDNNTFFDNF
NHSLLSPmAGESNYYLMPPSQMRNIAGNEQLSECGLTEIISANNSSTNSPIPDMDFPLEPVEIEPNFPFDTTGI
FS
>PmWRKY55
MEHNQILFLALPKSSDSASPNLPSNPSNISQVFPRFHFDSAGLMKNEAKIYQHCGTSYGSVDKMKSGKKEG
GKETKKHKYAFQTRSQVDILDDGYRWRKYGQKTVKNSKFPRSYYRCTHQGCVVKKQVQRLSKDEEIVV
TTYEGIHSHPTDETSAENFDQILRHLQTYNA
>PmWRKY56
MEAALGKSNSHEGSEKEEKRLVLESSSLADHDDQEAAGSEEDTLLKVGNGGSCQENNEMKSSSPTQKDL
SSSKQISTTSNMKVEPDHSMASTSSSRKEQDYQLESARAEMGEVREENQRLKKYLDRITDEYQTLQMQLY
DIQEKANKSKDVTIASTNNIYNHQYEESEMVSLSLGSFSSRAKKDEKNKNRSTSSQGKENESDRESLSLGL
DCKYEAPKSSAATTEVPLSNSSPmSSLEEVPKEEAGETWPPKKVLKTARSVEDEVAQQNPVKKARVSVRA
RCDTPTMNDGCQWRKYGQKIAKGNPCPRAYYRCTIAPSCPVRKQVQRCAEDMSILITTYEGTHNHPLPM
SATAMASTTSAAASMLLSGSSSSARSGLNPSVGATTTAAHDLHGYNFYLSDNSKSRFFIPNSSLSSSLPTITL
DLTSNPPPPSSSSNPLSHFNKFSSSSNSSHQLHPPTSLNFGSNSESNTNMSWSNGFLSYGAQLPPYNSNNKN
QIGSLSSLGIRQQQQPLQNNIYQNYMHKNNLNPTPPPHPSQGATPHQFQPDPIAAATKAITADPSFQSALAA
ALSSIIGSNSNVGTAGTTGMLGNNNNQAGGGDNNNMAQKFNWGDQFAGSTTTSSSPYLQTQIGNNSTIGC
ASSYLNKTTSANSQPGSLMFLPPSLPFASAPKSASTSPGDTRDHKSL
>PmWRKY57
MAEHESFDALQVEKLKPNEQEQEEDEEGDEDEDEDEEGKRLGEIHLGELQNSAPESRETQLETLAVPSTLE
LSENDQCAGFQVNSTSQSIDGAQLQEQLGVSHQEILAIVTHQDSQMQTQSPVQLAVYPTPLSELSPTSVTQ
SIQTQTQSPLQLTVVYPTPLSELSPTSVTQSISSAPSPILLEQKLPPEKVNTLCTPEVDKQNSSDHKFISSVPLVK
TSASDGYNWRKYGQKQVKSPQGSRSYYRCTYSECYAKKIECCDHSGYVTEIVYKSQHTHEPPQKSNCTK
ESKLALSAECVRNSVTEHPCRTFNDSEVSTSSKERIQETPSIPERKRQSPSDSDGNGDVKIKEEHGDGDEPE
PKRRQVYSVKKSNLEYSTSLLKPGKKPKFVVHAAGDVGISGDGYRWRKYGQKMVKGNPHPRNYYRCTS
AGCPVRKHIETAIDNTSAVIITYKGIHDHDMPVPKKRHGPPSAPLVAAAAPmSMNNLHIKKTDTHQNQISST
QWSVDTGGELTGEALDLGGEKAMESARTLLSIGFEIKPC
>PmWRKY58
MTSSFTHLLTSNMNNMDTNIDQERTNWGLSDYSSDRLMDRNGIEIPKFKSLQPPSLPMSPPPVSPSSYLAST
PmFSPTDFFSSPMFLSSSNTLQSPTSGAFSSQIFDWMSNSKETQQGMEREQKMFSEFSFQPETRPmATSSSSF

NIQASSIMASVEESLKMEQKPWDFNRISRQADSSTEKTGVKSEFEPLQIILPEIGTNQTNMQTNGPSGAPRP
DSIHCTQSSQFVREQKSDDGFNWRKYGQKQVKGSENPRSYYKCTYPNCPTKKKVERSLDGHITQIVYKGS
HNHPKPQSTRRSTSQSIQGSSYGISDQSVPTISNPKMESVTMQEDSSASIGEDEFEQNSPLSNSGGAEDENEP
EAKRWKGENANDQPFSAAGSRIVKEPRIVVQTTSEIDILDDGYRWRKYGQKVVKGNPNPRSYYKCTSVG
CPVRKHVERASHDTRAVITTYEGKHNHDVPmARGSGSYSNGNRPmSDNGSNNNSNVSMAVRPLALPNHS
NLSYLSSLQNTRQPTTERQSPYTLKMLQSEGSYGFPEF
